# A new primitive Neornithischian dinosaur from the Jurassic of Patagonia with gut contents

**DOI:** 10.1038/srep42778

**Published:** 2017-02-16

**Authors:** Leonardo Salgado, José I. Canudo, Alberto C. Garrido, Miguel Moreno-Azanza, Leandro C. A. Martínez, Rodolfo A. Coria, José M. Gasca

**Affiliations:** 1 Instituto de Investigación en Paleobiología y Geología, Universidad Nacional de Río Negro-Conicet, Av. Gral. J. A. Roca 1242, General Roca (8332), Río Negro, Argentina; 2 Facultad de Ciencias-IUCA, Universidad de Zaragoza, C/Pedro Cerbuna 12, 50009 Zaragoza, Spain; 3 Museo Provincial de Ciencias Naturales “Profesor Dr. Juan A. Olsacher”, Dirección Provincial de Minería, Etcheluz y Ejército Argentino, Zapala (8340), provincia del Neuquén, Argentina; 4 Departamento Geología y Petróleo, Facultad de Ingeniería, Universidad Nacional del Comahue, Buenos Aires 1400, Neuquén (8300), provincia del Neuquén, Argentina; 5 Departamento de Ciências da Terra, Geobiotec. Departamento de Ciências da Terra. Faculdade de Ciências e Tecnologia, FCT, Universidade Nova de Lisboa, 2829-526. Caparica, Portugal; 6 Facultad de Ciencias Naturales y Museo, División Paleobotánica, Universidad Nacional de La Plata, Paseo del Bosque S/N, La Plata (B1900FWA), Argentina; 7 Consejo Nacional de Investigaciones Científicas y Técnicas, Argentina; 8 Museo “Carmen Funes”. Plaza Huincul (8318), Neuquén, Argentina

## Abstract

We describe a new species of an ornithischian dinosaur, *Isaberrysaura mollensis* gen. et sp. nov. The specimen, consisting in an almost complete skull and incomplete postcranium was collected from the marine-deltaic deposits of the Los Molles Formation (Toarcian-Bajocian), being the first reported dinosaur for this unit, one of the oldest from Neuquén Basin, and the first neornithischian dinosaur known from the Jurassic of South America. Despite showing a general stegosaurian appearance, the extensive phylogenetic analysis carried out depicts *Isaberrysaura mollensis* gen. et sp. nov. as a basal ornithopod, suggesting that both Thyreophora and neornithischians could have achieved significant convergent features. The specimen was preserved articulated and with some of its gut content place in the middle-posterior part of the thoracic cavity. Such stomach content was identified as seeds, most of them belonging to the Cycadales group. This finding reveals a possible and unexpected role of this ornithischian species as seed-dispersal agent.

Dinosaurs were present in most terrestrial ecosystems for 185 million years, interacting with their physical environment and other living organisms, including plants. As in modern ecosystems, there were complex ecological relations between plants and plant-eating organisms. However, there is little evidence of the type and the parts of plants on which different phytophagous dinosaurs fed^1^,^2^,^3^,^4^. We describe a new species of ornithischian dinosaur with indubitable gut content consisting of a great amount of cycad and other plant seeds; this finding reveals that there were interactions between cycads and ornithischians in the dispersion of seeds.

The holotype of *Isaberrysaura mollensis* gen. et sp. nov. is an incomplete articulated skeleton including an almost complete skull and a partial postcranium from a medium-to-large-sized specimen (estimated length 5–6 m). This specimen was found in marine-deltaic levels of the Los Molles Formation from the Middle Jurassic (Neuquén province, Argentina); in fact, these are the first dinosaur remains found in this geological unit.

*Isaberrysaura mollensis* gen. et sp. nov. is the first neornithischian dinosaur from the Jurassic of South America. Up to now, the South American record of Jurassic ornithischian dinosaurs was limited to just a few specimens belonging to Heterodontosauriformes, a clade of small-sized forms that survived in Europe up to the Early Cretaceous^5^,^6^,^7^. By contrast, the other major clade of ornithischians, the Neornithischia, was highly and diversely represented in various Jurassic – above all Late Jurassic – localities in the world. Up to now, however, it has been considered absent from the Jurassic localities of South America.

The material here described was found by Isabel Valdivia and Erico Otilio Berry in the locality of Los Molles (Neuquén Province, Argentina, Fig. 1) and brought, partially prepared, to the ‘Prof. Dr. Juan A. Olsacher’ Natural Sciences Museum. In 2009 more field work was undertaken, and further fragmentary material was collected.

## Results

### **Systematic palaeontology**

Ornithischia Seeley, 1887

Genasauria Sereno, 1986

Neornithischia Sereno, 1986

*Isaberrysaura mollensis* gen. et sp. nov.

### Etymology

In honour of Isabel Valdivia Berry, who reported the finding of the holotype material.

### Holotype

MOZ-Pv 6459. A skeleton comprising a nearly complete skull, and a partial postcranium (still unprepared) consisting of 6 cervical vertebrae, 15 dorsal vertebrae, a sacrum with a partial ilium and an apparently complete pubis, 9 caudal vertebrae, part of a scapula, ribs, and unidentifiable fragments.

### Type locality and horizon

**Figure 1:**
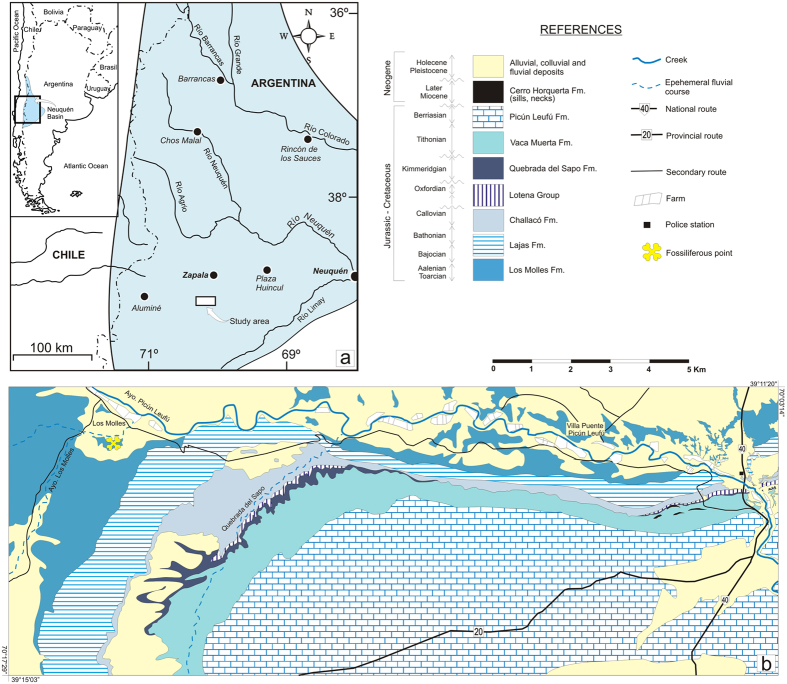
Geological map showing the type locality of *Isaberrysaura mollensis* gen. et sp. nov. The map was made by Alberto C. Garrido on the basis of a LANDSAT satellite image available in the Dirección Provincial de Minería (the institution where A.C.G. works) using Adobe Photoshop CS2 Serial Number: 1045-1412-5685-1654-6343-1431.

The holotype comes from the locality of Los Molles (Neuquén Province, Argentina) (Fig. 1). The specimen was found in the marine-deltaic deposits of the Los Molles Formation (Toarcian-Bajocian), which in this sector reaches a thickness of approximately 1,042 m^8^. The fossil-bearing level is composed of laminated pelites rich in ammonitiferous concretions and vertebrate remains, located some 40 m below the contact with the overlying unit (Lajas Formation, Bajocian-Bathonian). The presence of the ammonite *Sonninia altecostata* allows the fossil-bearing level to be situated biochronologically in the early Bajocian^9^. In palaeoenvironmental terms, the sedimentary succession comprises a large-scale progradational deltaic system, dominated by wave action and the influence of storms^10^,^11^. The dinosaur remains described here, the first reported from this unit, are among the oldest from Neuquén Basin^12^.

### Diagnosis

*Isaberrysaura* differs from all other ornithischian dinosaurs in the following autapomorphies: premaxilla with posterolateral process that does not contact the lacrimal, elongated maxilla and correlated increase in the tooth count and snout length, and the posterior process of the jugal at least as long as the anterior one. The phylogenetic analysis revealed that this dinosaur also differs from all other ornithischians in the following combination of synapomorphies: two supraorbitals incorporated into the orbital margin (char. 32, from 0 to 1, shared with *Agilisaurus, Haya, Thescelosaurus* and Pachycephalosauria), a broad contact between the postorbital and the parietal (char. 51, from 0 to 1, shared with Pachycephalosauria), subcircular supratemporal fenestrae (char. 66, from 0 to 1, shared with most ceratopsians), and an anteriorly downturned dentary row (char. 98, from 0 to 1, shared with Thyreophora).

### Description

**Figure 2:**
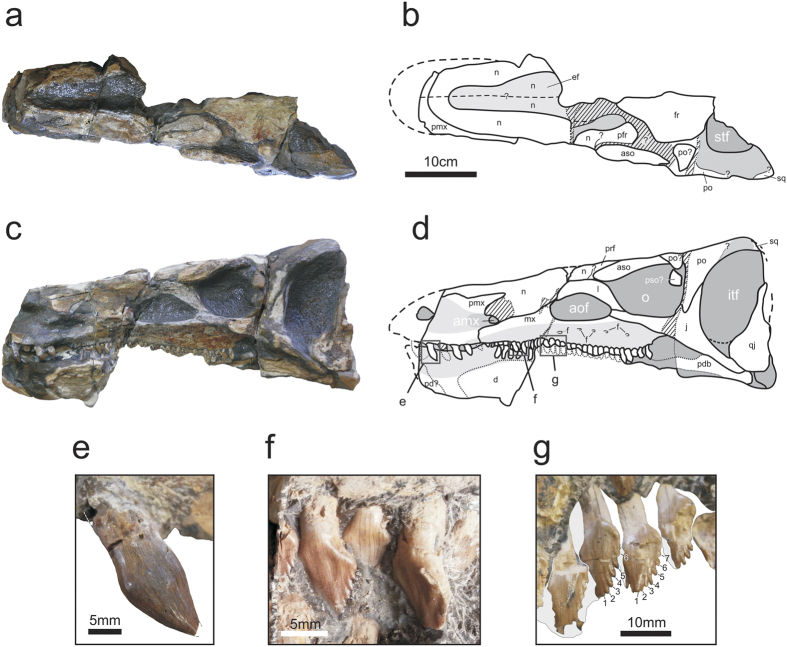
*Isaberrysaura mollensis* gen. et sp. nov. holotype. Skull in dorsal (**a** and **b**, photograph and drawing respectively), and left lateral (**c** and **d**, photograph and drawing respectively) views. (**e**) Premaxillary tooth; (**f,g**) maxillary teeth (**g** inverted). amf, anterior maxillary fossa; aof, antorbital fossa; aso, anterior supraorbital; d, dentary; ef, elliptical fossa; f, foramina; fr, frontal; ift, infratemporal fenestra; j, jugal; mx, maxilla; n, nasals; o, orbit; pd, predentary; pdb, postdentary bones; pmx, premaxilla; po, postorbital; pso: posterior supraorbital; prf, prefrontal; qj, quadratojugal; sq, squamosal; stf, supratemporal fenestra. 1–7 denticles. The drawings were processed using Adobe Photoshop CS2 Serial Number: 1045-1412-5685-1654-6343-1431.

The skull of the new species is estimated to be 52 cm long and 20 cm wide across the orbits, and it is almost as high as wide (Fig. 2a–d). The snout slopes anteroventrally from the posterodorsal corner of the infratemporal fenestra to what is apparently the maxillary-premaxillary contact. The infratemporal fenestra is dorsoventrally deep (dorsoventral length = 13.5 cm, anteroposterior length = 7 cm). In contrast, the orbit is subcircular, not quite as dorsoventrally tall (~6.5 cm) as anteroposteriorly long (~7 cm), smaller than the infratemporal fenestra, and only visible in lateral view (Fig. 2c,d). The anterolateral sector of the left supratemporal fenestra is relatively well preserved (although the bordering bones are mostly missing): it is visible only in dorsal view. The antorbital fossa is roughly triangular, with its base longer than the other two sides. In absolute terms, it is somewhat anteroposteriorly shorter than the orbit, its dorsoventral height being ~3.7 cm.

The jugal is triradiate. Unlike all non-cerapodan neornithischians, *Thescelosaurus, Hypsilophodon*^13^, and many basal iguanodontians, but similar to the condition in basal thyreophorans and stegosaurs, the anterior process of the jugal forms the posteroventral corner of the antorbital fossa, and surpasses anteriorly the base of the lacrimal, as in *Emausaurus, Scelidosaurus* and *Huayangosaurus* (Fig. 2d). The anterior process is almost as long as the posterior one (~7 cm). It is straight in lateral view, as in *Thescelosaurus* and basal thyreophorans and unlike the neornithischians *Agilisaurus* and *Zephyrosaurus*, where the anterior process of the jugal is curved^13^. In some basal thyreophorans (e.g., *Scelidosaurus*) and in stegosaurs (*Huayangosaurus, Stegosaurus*), the posterior process is much shorter than the anterior one^14^. The dorsal process of the jugal is proportionally as long as in *Kulindadromeus* and *Hexinlusaurus*, and shorter than in *Agilisaurus*^15^,^16^. In lateral view, the very broad quadratojugal is observed.

The nasals are ~20 cm long. There is a deep elliptical fossa along the presumed sutural contact of the nasals, although the actual suture line cannot be seen (Fig. 2a,b), a character that is present in a wide variety of dinosaurs (*Herrerasaurus, Changchunsaurus, Jeholosaurus, Haya, Agilisaurus, Stegosaurus, Heterodontosaurus* and *Hypsilophodon*). According to some authors^17^, thyreophorans lack such a fossa apomorphically, although others^16^ described in *Huayangosaurus* a shallow median depression on the dorsal aspect of the nasals.

There are two supraorbital bones in *Isaberrysaura mollensis* gen. et sp. nov. The anterior supraorbital is elongated (~10 cm), as in stegosaurs, and rod-like, as in basal ornithischians^18^ (Fig. 2c,d). The other element interpreted as a posterior supraorbital is located on the posterior margin of the orbit. There is another bone surrounding the orbit that is possibly part of the anterior ramus of the postorbital, broken and displaced (Fig. 2c,d). The postorbital forms most of the posterior margin of the orbit. Its jugal process surpasses ventrally half of the orbit; it has nearly the same width as the postorbital process of the jugal. The lacrimal is anterodorsally projected; it is rather slender, above all in its contact with the jugal. Dorsally, it contacts with the nasal, the prefrontal (mostly visible in dorsal view), and the anterior supraorbital, whereas anteriorly it articulates with the nasals and, apparently, with the maxilla. The lacrimal forms the posterior and posterodorsal margins of the antorbital fossa pretty much all ornithischian which retain an antorbital fenestra.

The premaxilla is incompletely preserved. It is robust, and the lateral surface of the oral margin is everted, as in the neornithischians *Thescelosaurus, Agilisaurus, Changchunsaurus, Orodromeus, Oryctodromeus, Talenkauen* and some basal iguanodontians^13^.

The posterolateral process of the premaxilla does not extend far enough posteriorly to contact the lacrimal, as in basal ornithischians and thyreophorans, and unlike *Heterodontosaurus, Jeholosaurus*, the basal ceratopsians *Liaoceratops* and *Yinlong*, and basal iguanodontians such as *Tenontosaurus*^13^.

It has at least six premaxillary teeth (three complete, two broken, and the mold of a sixth one), as in the basal ornithischian *Lesothosaurus*, the basal thyreophoran *Scutellosaurus*, and the neornithischians *Thescelosaurus neglectus* and *Jeholosaurus*^13^. In the anterior part of the snout, the posterodorsal process of the premaxilla is observed. This part of the premaxilla is broken, but this process seems to wedge into a recess of the nasal, or in between the nasal and maxilla. The premaxillary teeth are conical and slightly asymmetrical, and point posteriorly, somewhat lingually. The labial side is convex whereas the lingual side is only slightly convex. The crown is globose and has a constriction in the neck (Fig. 2e). The surface of the enamel is ornamented with parallel longitudinal crests, many of which are anastomosed. These seem to be more pronounced in the anterior teeth, which are also the most globose. Ornamentation is present (though less developed) in *Thescelosaurus*, and absent in *Changchunsaurus, Jeholosaurus, Zephyrosaurus, Scelidosaurus* and *Emausaurus*^13^. Unlike the maxillary teeth, a pattern of replacement is not observed in these teeth. Unlike *Agilisaurus* and *Huayangosaurus* there are no denticles in the premaxillary teeth^15^.

Unlike all neornithischians except *Agilisaurus*, but similar to the condition in basal thyreophorans like *Emausaurus* and *Scelidosaurus*, there is no diastema between the premaxillary and the maxillary tooth row^13^.

The maxilla of *Isaberrysaura mollensis* gen. et sp. nov. is anteroposteriorly broad. There is an anteroposterior ridge causing the tooth row to be inset medially, as in *Thescelosaurus, Lesothosaurus* and *Scutellosaurus* and other basal thyreophorans and basal neornithischians^13^.

On its lateral surface there are at least five foramina dorsal to the tooth row. The dorsoventral depth of the buccal emargination decreases anteriorly, which is an ornithischian synapomorphy according to character 26 of Butler *et al*.^17^. Anteriorly, it is 0.6 cm in depth (measured from its border to the margin of the alveolus), whereas at the level of the last alveolus it is almost 3.1 cm. In this respect, *Isaberrysaura mollensis* gen. et sp. nov. resembles *Stegosaurus*. There is a small depression in the anterior border of the maxilla, near the suture with the premaxilla, much like that present in *Changchunsaurus, Haya, Hypsilophodon, Jeholosaurus, Orodromeus, Zephyrosaurus*^13^ and *Huayangosaurus*^14^. This depression is here interpreted as the anterior maxillary fossa^13^. As in other genera (*Huayangosaurus*, ZDM7001; *Thescelosaurus*, NCSM 15728), the floor of the fossa seems to be formed by a flange of the premaxilla^13^,^14^.

The maxilla has at least 30 tooth positions. In *Scelidosaurus* (BMNH R1111) there are 19; in *Thescelosaurus* 20; in *Agilisaurus* 14; in *Emausaurus* 21; in *Stegosaurus* 24, and 27–28 in *Huayangosaurus*^14^,^15^,^19^. The maxillary teeth are closely packed, without space between the alveolar margin and the adjacent crown. They are lanceolate, partially imbricate and slightly curved distally, as a result of which they are asymmetrical in labial view (Fig. 2g). They have 5 to 7 large denticles with an angle of 45°. They present a slight eminence at the base of the tooth crown, much less developed than in *Scelidosaurus*. The enamel surface is smooth. The anteriormost maxillary teeth are somewhat smaller than the posterior ones and are slightly twisted anteriorly (Fig. 2f).

### Gut contents

**Figure 3:**
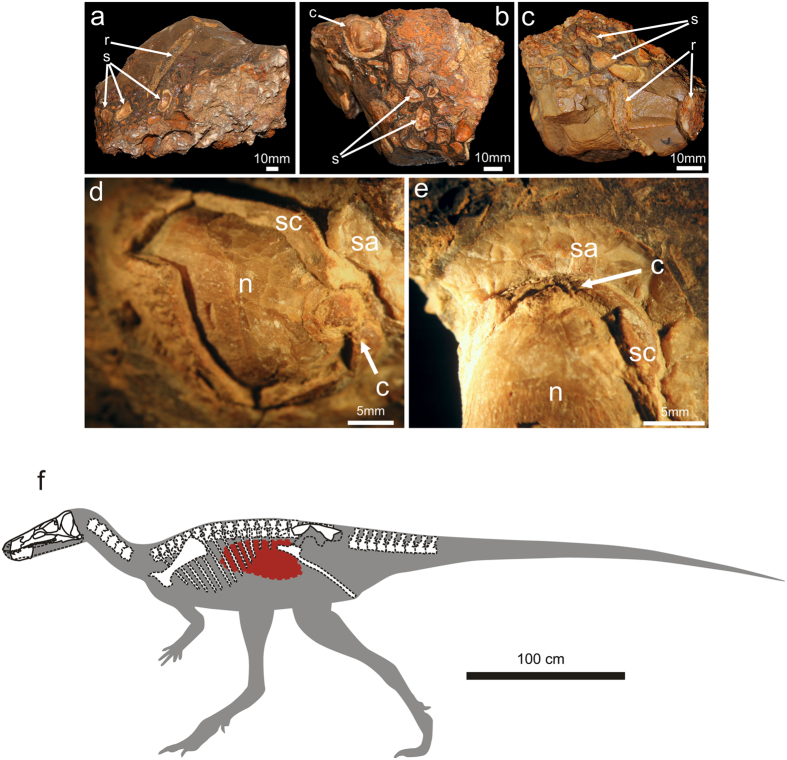
Gut content of *Isaberrysaura mollensis* gen. et sp. nov. (**a**–**c**), seeds of cycads (c), and other seeds (s); rib (r). (**d**,**e**) Detail of seeds of cycads: sarcotesta (sa), sclerotesta (sc), coronula (c), nucellus (n). (**f**) Location of the gut content in the reconstructed skeleton of *Isaberrysaura mollensis* gen. et sp. nov. The drawings were processed using Adobe Photoshop CS2 Serial Number: 1045-1412-5685-1654-6343-1431.

One of the most notable features of the discovery of the skeleton belonging to *Isaberrysaura mollensis* gen. et sp. nov. is that inside the skeleton there was a mass of permineralized seeds in the middle-posterior part of the thoracic cavity (Fig. 3f). There is little direct evidence of the feeding habits of herbivorous dinosaurs that matches the stomach contents preserved within a carcass^1^,^20^. Most unaltered gut contents in plant-eating dinosaurs are found in hadrosaurid ornithopods^2^, whereas until now there has been no known record in basal neornithischians. Two types of seeds were recovered close to the posterior ribs of *Isaberrysaura mollensis* gen. et sp. nov., distinguished according to their size. The largest seeds preserved three layers: an outer fleshy sarcotesta, the sclerotesta, and the inner layer (possibly corresponding to the nucellus). These seeds are assigned to the Cycadales (Zamiineae) on the basis of a well-defined coronula in the micropylar region, whereas the smaller, platyspermic seeds are still indeterminate.

The largest fossil seeds were found with an entire sarcotesta, suggesting that they were gobbled down, and not chewed. This is consistent with the morphology of the maxillary teeth of *Isaberrysaura mollensis* gen. et sp. nov. and analogous with some living tetrapods (e.g. elephants and peccaries), which eat the seeds of cycads but avoid masticating them^3^. The sarcotesta is a soft tissue that is normally digested, but the sclerotesta is a hard tissue that ensures safe passage of the endosperm through the digestive tract; this germinative strategy has been proposed for the seeds eaten by some dinosaurs^3^,^4^. The well-preserved mass of seeds with a sarcotesta, clustered close to the ribs, suggests that the digestion in the holotype specimen of *Isaberrysaura mollensis* gen. et sp. nov. was in its first steps in the gut.

Extant cycads produce harmful toxic compounds (e.g. cycasin), storing them in stems, leaves and seeds. The sarcotesta in these cases contains high levels of toxins^21^; however, the sarcotesta is edible, especially for large-bodied animals such as dinosaurs. The microbial “gut flora” of these reptiles probably contained micro-organisms that produced active enzymes capable of cleaving the cycad molecule cycasin^3^,^4^,^21^. The seeds with a thick sclerotesta would then pass through the digestive system, to be excreted as seed kernels. These findings suggest the hypothesis of interactions (endozoochory) between cycads and dinosaurs, especially in the dispersion of seeds.

*Isaberrysaura mollensis* gen. et sp. nov. shows marked heterodonty. The possession of recurved premaxillary and lanceolate maxillary/dentary teeth in extant iguanid lizards is correlated with diets that include a mixture of animal and plant material^22^. However, the stomach contents of *Isaberrysaura* are composed entirely of seeds, with no evidence of animal remains.

### Phylogenetic analysis

#### Butler dataset, Godefroit et al. version

This analysis resulted in 1740 most parsimonious trees of 603 steps (consistency index 0.421, retention index 0.688).

**Figure 4:**
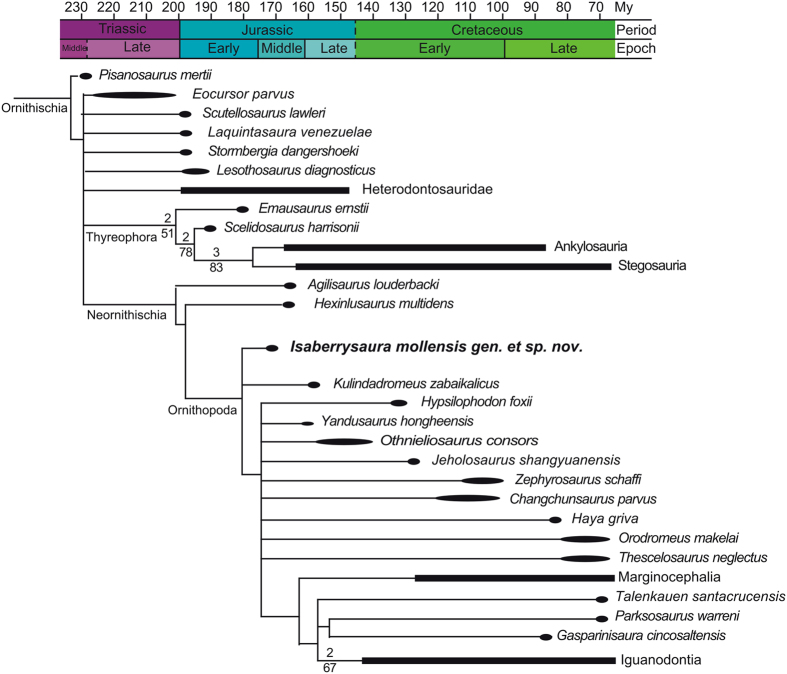
Phylogenetic position of *Isaberrysaura mollensis* gen. et sp. nov. Calibrated reduced strict consensus obtained after including the Argentinian taxon in the current iteration of the Butler *et al*.^17^ dataset. Numbers over branches are Bremer support values over 1. Numbers below branches represent bootstrap support values over 50.

As in most previous analyses of this dataset^17^,^23^,^24^,^25^, the resulting consensus is an uninformative polytomy. Reduced consensus trees were obtained using the “tree-pruning” option of TNT, *a posteriori* removing wildcard taxa, following the original approach in the first iterations of this dataset^17^,^23^,^24^, by contrast with other analyses, where certain taxa were removed *a priori*, on the basis of previous analyses^16^. Given the intricate puzzle that ornithischian phylogeny currently represents, we consider that *a priori* removal of taxa, although it certainly increases the resolution of the consensus, can lead to important omissions in the phylogenetic relations of this clade, and may result in the misidentification of clade synapomorphies. Using the “tree-pruning” option of TNT, we searched for reduced consensus obtained after pruning up to 5 taxa. From the multiple sets of five taxa recovered, we chose to prune *Echinodon, Anabisetia, Koreanosaurus, Yueosaurus* and *Albalophosaurus a posteriori*. The reduced consensus tree gained 10 nodes, and recovered *Isaberrysaura* at the base of Neornithischia, in a trichotomy with *Kulindadromeus* and all more derived neornithischians (Fig. 4). It shares with all ornithopods the presence of more than six sacral vertebrae (char. 137, from 2 to 3), a character also shared with some heterodontosaurs and many stegosaurs and ankylosaurs.

Enforcing *Isaberrysaura* within Thyreophora resulted in 1140 equal-length trees of 607 steps (consistency index 0.418, retention index 0.684). These trees recovered *Isaberrysaura* as the sister group of Ankylosauria + Stegosauria, and are 4 steps longer than the unconstrained most-parsimonious trees. To test the significance of this result, 1000 replications of the Templeton test were used, comparing pairs of unconstrained and constrained trees chosen at random from both tree spaces. All tests produced non-significant results (see Supplementary Information, C5), implying that the hypothesis of *Isaberrysaura* being a basal thyreophoran cannot be rejected with confidence.

#### Butler dataset, Baron et al. version

This analysis resulted in 69 most parsimonious trees of 594 steps (Consistency index 0.428, retention index 0.689).

Again, the strict consensus shows a huge polytomy. To improve resolution, five taxa were *a posteriori* pruned from the consensus (*Echinodon, Anabisetia, Yandusaurus, Yueosaurus* and *Koreanosaurus*). The resulting topology mimics that obtained by Baron *et al*.^26^, with the inclusion of *Isaberrysaura* among the basal neornithischians, more derived than *Hexinlusaurus* but less derived that *Othnieliosaurus* (Supplementary Fig. S1). Despite the addition of *Laquintasaura* and the mergin of *Lesothosaurus* and *Stormbergia* carried by Baron *et al*.^26^, resulting in a better characterization of the Thyreophora clade, *Isaberrysaura* remains immobile in its ornithopod placement. Enforcing *Isaberrysaura* within Thyreophora resulted in a total of 7776 trees of 598 steps (consistency index 0.425, retention index 0.686); four steps longer than the most parsimonious trees. Noticeably, the consensus of the constrained trees does not recover Thyreophora, which is collapsed into a big politomy together with *Isaberrysaura, Lesothosaurus, Emasaurus*, the Ankylosauria plus Stegosauria clade and the Ornithopoda clade. Again, 1000 replications of the Templeton test failed to reject this topology (Supplementary Information, C5).

#### Boyd dataset

A total of 180 most-parsimonious trees of 889 steps were obtained (consistency index 0.359, retention index 0.660). The resulting topology of the strict consensus resembles that published by Boyd^27^, with some important exceptions. *Isaberrysaura* is recovered as belonging to Parksosauridae, the sister group of Cerapoda, but its location within this clade remains uncertain (Supplementary Fig. S2). The inclusion of *Isaberrysaura* results in the collapse of both Orodrominae and Thescelosaurinae subfamilies into a polytomy. *Isaberrysaura* is placed in a variable basal position within Thescelosaurinae or as a sister taxon of the clade containing *Orodromeus* and the unnamed taxon from Kaiparowits. *Isaberrysaura* differs from all Parksosauridae in the absence of a diastema between the premaxillary and maxillary teeth (char. 8, from 1 to 0), and shares with them the everted lateral surface of the oral margins of the premaxilla (char. 5, from 0 to 1, also shared with *Agilisaurus*), the concavity of the posterior end of the premaxilla for receipt of the anterolateral boss of the maxilla (char. 14, from 0 to 1) and the presence of fused premaxillae (char. 255, from 0 to 1).

Enforcing *Isaberrysaura* within Thyreophora resulted in 72 equally parsimonious trees of 894 steps (consistency index 0.357, retention index 0.657), five steps longer than the most parsimonious placement of *Isaberrysaura* within Parksosauridae. *Isaberrysaura* is recorded in a polytomy at the base of Thyreophora, in an unresolved position between *Scutellosaurus, Lesothosaurus* and the clade formed by *Emausaurus* and *Scelidosaurus*. It is important to note that in this topology no synapomorphies support the clade Thyreophora, due to the enforced placement of *Isaberrysaura*, which indeed differs from all other thyreophorans in possessing a ventrally deflected margin of the premaxilla at the level of the maxillary teeth (char. 6, from 0 to 1, shared with Heterodontosauridae, *Orodromeus, Hypsilophodon* and *Zalmoxes*) and in possessing a pubic peduncle of the ilium that tapers distally and is smaller than the ischial peduncle (char. 192, from 0 to 1, shared with all neornithischians but *Agilisaurus*). Again, 1000 replications of the Templeton test confronting the two topologies do not rule out the hypothesis of *Isaberrysaura* being a thyreophoran (see Supplementary Information, C5).

#### Remarks

*Isaberrysaura mollensis* gen. et sp. nov. has been included in three different datasets, all of three recovering it at the base of Ornithopoda. Despite the general stegosaurian appearance of the specimen, and presenting an anteriorly downturned dentary row, a synapomorphy of Thyreophora, the extensive analysis carried out does not allow us to consider the Neuquenian species as a basal member of this clade. Further preparation of the type specimen, findings of additional specimens and, above all, a better and more compressive dataset focused in the basal thyreophorans may alter this results in the near future, and seed light on the *Isaberrysaura* puzzle: was it a stegosaurian mimic ornithischian, with a skull shaped to profit similar vegetal resources as derived thyreophorans or it is a very basal form of the thyreophoran clade?

## Discussion

The discovery of the new basal neornithischian *Isaberrysaura mollensis* reveals the existence of a previously unknown morphotype among basal neornithischians. The cranium of this new species is reminiscent of that of the thyreophorans. Among the characters shared with the latter are their large body size, their elongate and low skull (as occurs in stegosaurs, *Emausaurus*), at least six premaxillary teeth (6–7 being common in many thyreophorans, but infrequent outside Thyreophora), the high maxillary tooth count (as in *Huayangosaurus*), the depression between the premaxilla and maxilla (as in *Huayangosaurus*), the very deep buccal emargination (also found in stegosaurs), and the anteriorly downturned dentary tooth row (a thyreophoran synapomorphy). The interpretation given here is that *Isaberrysaura* and the thyreophorans were convergent forms of ornithischians. Like thyreophorans in general and the basal stegosaurs in particular, *Isaberrysaura* shows weak or non-existent wear facets, indicating a lower degree of oral food processing. This in turn is consistent with the state of the seeds found in the digestive tract.

Although the reason for the many similarities between *Isaberrysaura* and the thyreophorans could have been the diet they had in common, we still lack a clear idea of the diet of these dinosaurs beyond their ingestion of cycad seeds and other seed plants. We conjecture that the summer diet consisted mainly of fructifications, but we remain completely ignorant of what food resources were used in periods when the seeds were unavailable. The palaeobotanical association of conifers (Podocarpaceae, Araucariaceae and Cheirolepidiaceae), Cycadales, Bennettitales and ferns from the Middle Jurassic of Neuquén Basin suggests humid-temperate to warm climates^28^,^29^,^30^,^31^. These plants would belong to forest to open environments; such a diversity of environments with a rich flora would have yielded enough food resources for the development of *Isaberrysaura* and the associated fauna.

The central and southern sector of Neuquén Basin was invaded by the waters of the Pacific Ocean from the Pliensbachian (Lower Jurassic) to the Lower Cretaceous, interrupted only by brief periods of continentalization^32^,^33^,^34^. The deposition of the Los Molles Formation was associated with the first major flooding of the basin, with the development of low-energy marine facies in restricted environments deficient in oxygen, grading towards the far south of the basin into the deltaic, shallow marine and estuarine deposits of the Lajas Formation^10^,^11^,^35^,^36^,^37^,^38^,^39^. To date, no outcrops of a clearly continental origin providing terrestrial vertebrate remains have been found for this period (Pliensbachian-Bathonian) in Neuquén Basin, so the discovery of this new ornithischian certainly contributes to our knowledge of the Jurassic dinosaur faunas of Patagonia, known above all from Late Jurassic forms.

## Methods

To assess the phylogenetic position of *Isaberrysaura*, it was coded in the three largest datasets available in the literature: two different versions of the matrix built by Butler *et al*.^17^, one after Makovicky *et al*.^23^, Ruiz-Omeñaca *et al*.^24^, Barrett *et al*.^25^ and Godefroit *et al*.^16^ (Supplementary Information C1), and the other after Ham *et al*.^40^, and Baron *et al*.^26^, (Supplementary Information C2); and the dataset published by Boyd^27^ (Supplementary Information C3).

### Butler dataset, Godefroit *et al*. version

The Butler dataset includes a total of 57 taxa scored for 227 characters, five of which (112, 135, 137, 138, 174) were treated as ordered (see Supplementary Information). The resulting dataset was analysed with TNT v1.5v^41^. A heuristic search with 1000 replicates, followed by branch swapping by tree-bisection-reconnection (TBR), holding ten trees per replicate, was conducted. Additional rounds of TBR were performed, obtaining increasing numbers of trees (up to two million), but no impacts on the topology of the consensus were observed other than increasing computing times, so all the following analyses were performed with the first set of trees.

### Butler dataset, Baron *et al*. version

The second version of Butler’s dataset includes a total of 55 taxa scored for 227 characters, five of which (112, 135, 137, 138, 174) were treated as ordered (see Supplementary Information). Following Baron *et al*.^26^, 100 replications of new technology searches, including sectorial searches, ratchet, drift and tree fusing algorithms were carried under default settings, followed by and additional round of TBR using the obtained trees as starting seeds.

### Boyd dataset

*Isaberrysaura* was also coded in the dataset by Boyd^27^ for a total of 69 taxa coded for 255 characters (see Supplementary Information). A heuristic search with 1000 replicates, followed by branch swapping by tree-bisection-reconnection (TBR), holding ten trees per replicate, was conducted. This was followed by an additional round of TBR using the obtained trees as starting seeds.

### Constrained searches

To explore the alternative hypothesis of *Isaberrysaura* being a thyreophoran, constrained searches were conducted enforcing the monophyly of the clade that contains *Isaberrysaura* within Thyreophora in both datasets. After enforcing the constraint, the same search settings as those described above were used for the constrained search.

The Templeton test^42^,^43^ was used to test the significance of these results. The test was run using a modified version of the TNT script by Schmidt-Lebuhn (see Supplementary Information, C4, original script can be downloaded at http://www.anbg.gov.au/cpbr/tools/templetontest.tnt). The Templeton test compares topologies in pairs in order to ascertain whether they are both significantly supported by the data or whether one of them can be rejected. This test has been used in previous dinosaur studies, normally comparing the topologies of one of the most parsimonious trees with one or more constrained topologies, both topologies being chosen at random from the results of the normal and constrained tree searches. Due to the high numbers of trees recovered in both the normal and constrained searches performed in our study, we consider that a comparison of one pair of trees may not be representative, so we modified the script from Schmidt-Lebuhn (Schmidt-Lebuhn, n.d.) to run for 1000 iterations, each time choosing pairs of trees at random, and recording the results of each Templeton test in a table that is saved as an output file. The script works in the current version of TNT, and a detailed description of how it works is included with the script in the supplementary information.

## Additional Information

**How to cite this article**: Salgado, L. *et al*. A new primitive Neornithischian dinosaur from the Jurassic of Patagonia with gut contents. *Sci. Rep.* **7**, 42778; doi: 10.1038/srep42778 (2017).

**Publisher's note:** Springer Nature remains neutral with regard to jurisdictional claims in published maps and institutional affiliations.

## Supplementary Material

Supplementary Information
