# Supplementary material for: A new primitive Neornithischian dinosaur from the Jurassic of Patagonia with gut contents
Source: Sci Rep. 2017 Feb 16;7:42778. doi: 10.1038/srep42778 (PMC5311864; doi:10.1038/srep42778)
Supplement: Supplementary Information [file srep42778-s1.pdf]

SUPPLEMENTARY INFORMATION FOR

Salgado, L., Canudo, J.I. Garrido, A.C., Moreno Azanza, M., Martínez, L., Coria, R.A., and Gasca, J.M. 2016.

A new primitive Neornithischia dinosaur from the Jurassic of Patagonia with gut contents.

This PDF file includes:
Institutional abbreviations
Data Matrix
Supplementary figures

Supplementary Information is linked to the online version of the paper at

www.nature.com/nature.

Institutional Abbreviations:

BMNH, Natural History Museum, London, UK.

MOZ-Pv, 'Prof. Dr. Juan A. Olsacher' Natural Sciences Museum, Zapala, Neuquén, Argentina.

NCSM, North Carolina Museum of Natural History, USA.

ZDM, Zigong Dinosaur Museum, Dashanpu, China.

Figure S1. Phylogenetic position of *Isaberrysaura mollensis* gen. et sp. nov. Reduced strict consensus obtained after including the Argentinian taxon in the current iteration of the Butler et al. dataset after Baron et al. Numbers over branches are Bremer support values over 1. Numbers below branches represent bootstrap support values over 50.

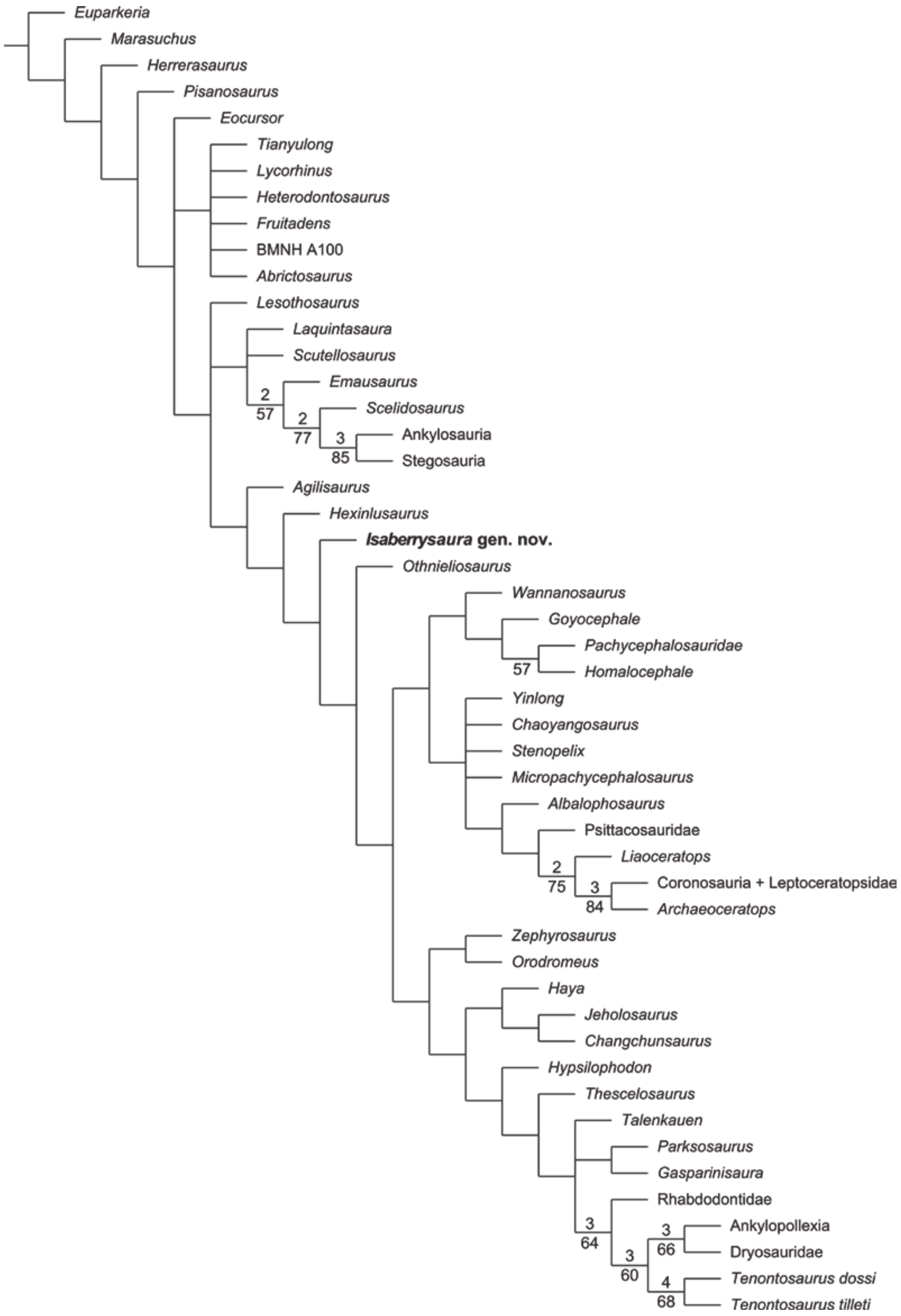

Figure S2. Phylogenetic position of *Isaberrysaura mollensis* gen. et sp. nov. Strict consensus obtained after including the Argentinian taxon in the current iteration of the Boyd et al.[27]. Numbers over branches are Bremer support values over 1. Numbers below branches represent bootstrap support values over 50.

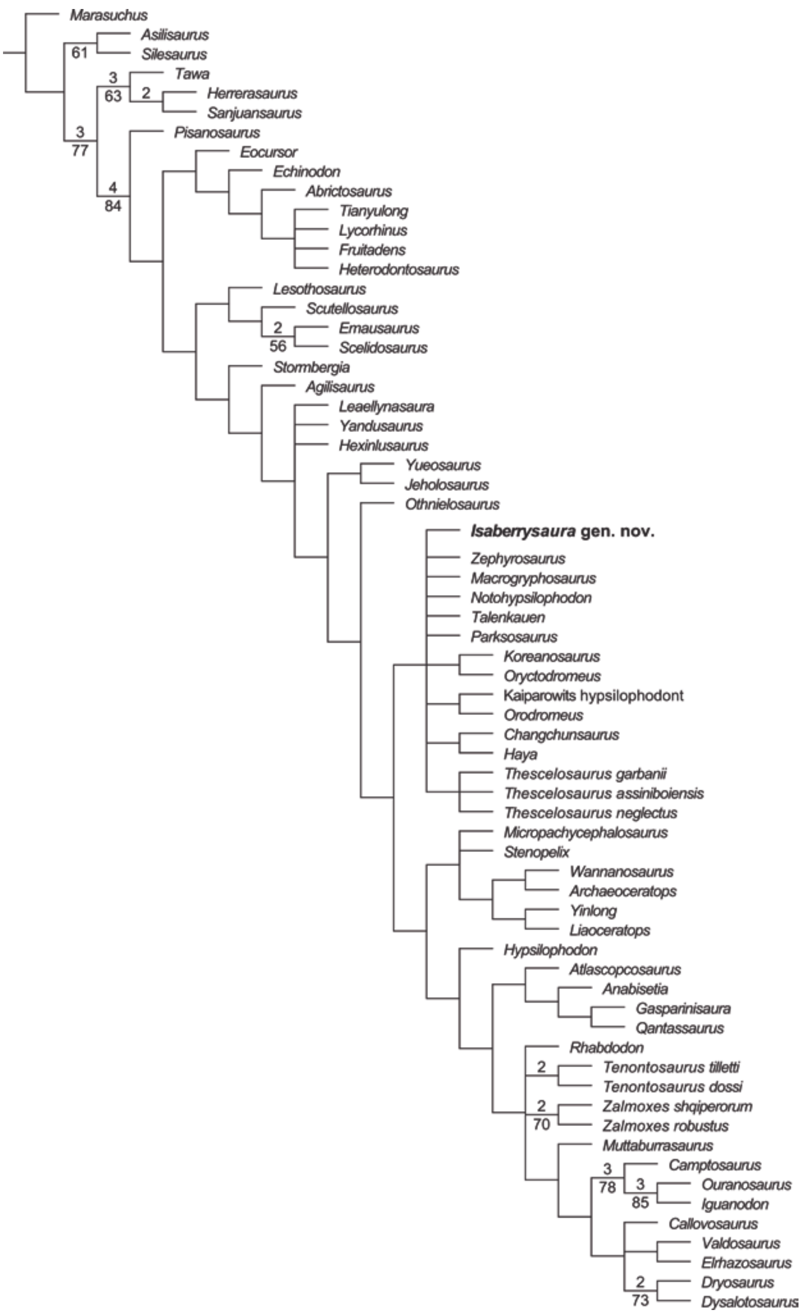

Supp C1. TNT file containing the dataset of Butler et al.[17] after Barrett[15] plus coding for *Isaberrysaura* mollensis gen. et sp. nov.

```
"nstates 8 ; "
xread 'Data from Salgado et al.'
227 57
Euparkeria_capensis 000??000000000?0000000000000????000?000000000000000000000000100000??
000000100000000?0000?????000000000000000002000000?00?1001000000100010000000?100011000?00???0?
10000000??20100000000000?000???0000?0000000000000000?1?000?000
Marasuchus_liloensis_ ?0????????????????????????????????????????????????????????????????????????00?
0?????????????????????????????????????????????010?0?00000?0????????000????????10000010??
20000000000000?000???000101000000101001000?0?000?000
Herrerasaurus_   000??000000000?11010010000000????000?000000001000000000000000000100000??000000100?
000?0?0000?????000000000000000002000000?00?10010000000020100000000????1110001101001100000000?
0100000010000000?000???0001010000000000000000?00001?000
Pisanosaurus_mertii ?0??????????????????????
1???????????????????????????????????????????????????????????????000?10?000000????1??0?00??00?
11110??0???????????????????????????????????????????????0???????????????0?0?0????0???0001??000
Abrictosaurus_consors 000??10011??011000?100??0100100000?0?????????????????
0?????????????????????????????????011000?1000011000?00?0030011?0?0??001?11110????????0?????????0?
10000?10??01?01000000?11?100??????????????????0?2?200??1???010110???0001?11??
BMNH_A100       ??0??1?0??1?01100??100?00100???????0?0??00?00???????????????????000??????
0??????????????0???????0000?????????0030111?0?0??
011111110??????????????????????????????????????????????????????????????????????????????????????
1?????
Echinodon_becklesii ??0????0??1????0????????
01???????????????????????????????????????????????????????????????1??????0000??0????0??0[34]??1100?00?
00?
011110??????????????????????????????????????????????????????????????????????????????????????????????
Fruitadens_haagarorum ??0??1?0??1??11?????0??
001?????????????????????????????????????????????????????????????????0000??0???????03101100?00?
011011110??0???3???????????????0001?????????????????????????????????????????00212000111?????????????
011110
Heterodontosaurus_tucki 000??1101111011000110010010010000000?00100100?0000001000000000200000??
000000000000000?00011000?1000011000000100301111101 1[01]111111011?001?03?00000010?
0110000110100110100000 0?111100000000?110?001000100212000 01??00101101000001111 10
Lycorhinus_angustidens ????????????011????100??
010???????????????????????????????????????????????????????????????????????????????????????11?0?0??0??
111110????????????????????????????????????????????????????????????????????????????????????????1?????
Eocursor_parvus  ?0??????????????????????????????????????????????????????????????????0000?????????????
00?????????????10000100010000????11?0??0?00?01?110??0?0?2??????????0010000??01?01?01?00000?
1110000000100110?0010001002020 0001???0?????1??0000000?1
Lesothosaurus_diagnosticus 000??100011000?000?10000010110000000?00000000100000010000000000000000??
0000000000000?0?000100000010000100010000000101100?00?000011110000?0?20?????????00101000000?
00001000000?010000000001 00110?001000100202000 0111001?110100000100001
Stormbergia_dangershoeki
???????????????????????????????????????????????????????????????????????????????????????????????????
??????????????????????????0?0?0120000??????0010??0????????01000000?0100000000[01]11110?
0010001002020000111?01?1101??000??0001
Scutellosaurus_lawleri ?00????0?11????10????????10???????10?000000000100??????0??0????
1100????????????????????????1????????0000????1?0?000?01100?00?0?00111100?01012??0?1????????1?000?00??
00001000000??1?0??0000000110?0010??10020200001???0????01??110000001
Emausaurus_ernstii 0?0???000?1000?000?100000100101000 10?000000001000000 1000?00000?00000??0000????
00?00?????1?0?????11000100010000011011 00?00?000011110?????????????????????????
01???????????????????????????????????????????????????????0???11?0?????
Scelidosaurus_harrisonii 000??100???0?0????0101000100 11?2?010?000010001000000010000000000100000??
000000?001001?10001?0?????110001010100010110 1100?00?000011110000000 2200000 0010?0010000????????
01001000?0000000000001110?011000100202100011100001111001111 00001
Stegosauria      000??1000110[01]0?0000100000100 11?2?010?0000000000000011000110000100000??
0000[01]0100100001?000100000001100110000 0000[01]?101100?00?000011110110[012]0[012]2000100010?
```

0011000?010100001111000?0000000002001110?0[01]1010110??01000111?121111[01]0011000?001
Ankylosauria   000??100011000?000011?00010011?2??10?00001100000000?1?10010000101?10??100000?
00100001100110000?11100010100000010?101100?00?00001111011000?[12]000000010100?1000?
010100011111000?000000100[02]001111[01]111000110??01000111?121111100111[01]0?0?1
Agilisaurus_louderbacki 000??100011000?00011000?010?10111000?00000000000000?10001000001000000??
000010?00000??0?00010?0000100011010000000110110 0?00?0200111100101012000000010?1010100???????
01000000?010001000011011 0?001010100202000011100101101000000100001
Hexinlusaurus_multidens ?00???0?????????011000??10?10000100?00000000000000?1000?00000?00000??
00001?????????????0???????1000110????000????1101000?0?00111100101012000000010?
1010000000000000001000000?1101010000011110?0011111002020000111101011010000000001
Othnieliosaurus_consors
??
00???????0?0????1110?00?0???1?1?0??0?013?000?011010010000????????01000000?1111110000011110?
00111110131200001111010110 1?0000?00001
Anabisetia_saldiviai ?????????????????????????
1??0101????????0????11?110??????
111????????????????????00100100000??0001100000?1111110001011110?0010111013120101111?01?1101?0000?
00?0?
Gasparinisaura_cincosaltensis 00???????????10????1010??10010000100?0000000000000?01100?000?0??00?0??
000000000?????????0????????00011011000 10????111111??000?111100?0???3100??1?10???00?00????????
01000010?11111100000011110?0011111013120000111102011011 0000?00001
Hypsilophodon_foxii 000??1001110110000110010010010000100?00000000000000110000000111010000??
000010000000100?
00011100001000110110001001101111[01]010000011110010101310000011010010000000000000001000000?
1211111000001111 0?0011111013120000111100011011 0000100001
Orodromeus_makelai 000??100011?10?00011010101001000000 0?0010000000010011000000000000010000??
0000100000000?????01???0?010001101100000011011 00?00?00001111001010131000?0?1010010?
00000000000 1000000?121111000001111 0?001111101312000[01]11110001101?000010001?
Parksosaurus_warreni 00????0??????10???01010??10010?0?100?0000001?00?000?1100?000?00?0000????
00???????0??????0????????000110???0010????111?100?000011110????023?00?0111?10000000????????
01000000????1??000001111??001111101312100?1???0001101100001?0001
Thescelosaurus_neglectus 000??100111011 0000?10101010010010100?00000000000010011000?00?11000000??
0000?00100001?????011100101000110112000000101101101000001111001010231001?
0110000100000000000001000010?1111011000011110?001111101312100011110001101100001 00001
Yandusaurus_hongheensis ????????????????????100000100??????00?0000000000??????????
0??????????????????????????????0?????????????????????????111?10??0???11110??0?0????????????0010?
00??000??????????0??????10????
Zephyrosaurus_schaffi ??0??1?01110?10000?10??1010?10100?00?00100000?0?100??0??100???010000??00?
0100000001?????0?????????????????????0110111?100?00?
011110??1?????
Tenontosaurus_tilleti 000??11110100??001010100110010000100?0000000000100011100000111000000??
0000101100001?0?000110100010101111200001???
211100101000111100012023100110010100000000000001100010000 10?
11111100010111110001011111312111111110001101100001 00001
Tenontosaurus_dossi 000???1110100100010101001100100001 00?0000000000100011100000111000000??
000010110??0??0?0?011010001010111???00000???211100101000111110?0120?3?00110?1010000000000???
0001000010?11111100010111110?0010111?131211111111?00011011000001 00001
Talenkauen_santacrucensis ?00??101??1?010000?10???
0100???01110?10100011?????0?001???1??10???0??
11110?00102????????10?0010010????????01000010?1111?1???????11??0010111?1312????111?000?101?0000??
00??
Rhabdodontidae   000??10010100??0000101000100?????100?000000000110001110000000?0200000??
0000101000?01?????011000101010111??000001???2111101010001111 0?11?0?3?001?0?10??000?00????????
01001000??111011010001????????????11312111?111???????11?000100001
Dryosauridae   000??11110110??0010101001101100010 00?000000000100001110000011?000000??
00001011000010 0?0001101010100011110000001???2111011010001111 0?11101310010001010000000????????
01000010?1111111011011110?0010111013120111111102011010000000100001

Ankylopollexia 000??11110100??00101010011001000000?00000000010000111000000[01]10000000??
000010110000200?00011010101010111110001 01???211101101000111110 11[12]02[23]?
00100010[12]00[01]000000001100010000[01]0?
1111111011011111000101111[23]121111111000011010200010 0001
Micropachycephalosaurus_hongtuy ??00???
2???11?0????0???111?0????????????0??????????????????01?
000??????1?1??????????????????0131?0?0?????????????????0?????
Stenopelix_valdensis
???
??????????????????????????????????3?00??0???????1??????????01001100?111101020000 1111?00121?10131?????
1???000110100000???001
Wannanosaurus_yansiensis_ ?????????????????????????????11????00?000101000000???????????????
0001001101??????????????0???????000010010000 00???11?0??0?0???11110??0??????????????????111?????????
1?111??????1?????????????????????0????01??1???????????????0001?0
Goyocephale_lattimorei ??0???001???01100001?????10?11?1??00?0001?10??????1????????????
0000100111110?????1?1??0?0???????00001?010?0100031111?[01][01]00?01??111?00???1?2??????????2????
111????????0101110101211?????????????????????????????1??1?????011100010????
Homalocephale_calathocercos_ ??0?????????????????11????10?11?1??00?000111000000?
111000000000000001100111110?000110?0??10???????????????????0?????11?110??0???11110????1?3111??0???
2???????????????0101110111211111100001111110131110131 10?0?11?10?????1110001?010?
Pachycephalosauridae 000??100111001[01]000011???010011?1??00?000111000000?11100000000000 1?
1100111110?[01]0011010?[01]10???????000010010001 0003[01][01]11?[01][01]00?0[12]?0[01]1110????1?3?11??
0???20110111????????0101110111211111100001???????????013110?0????????????111000 1001?1
Chaoyangosaurus_youngi 1?1001?00?0?000110?1????011???????210000 1????000????1000?
000002????????????????0??0??0?0?011101?010001101001100041011 10?00?000011110??0????????????????
1???1?????
Psittacosauridae 1110011001000??11001???0011010100?
21010000000100000110000000000200001010000 0[01]000000010?10011[01]00001001111[01][01]000001???
11111110000011110010[01]003100?0001110[01]10000000000000001000000??
211[01]10000001111100121010[01]31201001110000110100000100001
Liaoceratops_yanzigouensis_ 0?1111100100010000 11000?0110?????
0211100010110000101100000100020010111000001?010001?0?
00011001001001111100110003101111111110000 11110???
???1?????
Archaeoceratops_oshimai 011111000100010000 01000?010010100021111011011000010?1011?
01000200101110000 01?01??01?0???011101101000111101110003101 1???1?1000011111????103110??
0??????????????????01000000?1211110?000011111001210 1?131?????1111000110100000 1???0?
Coronosauria_Leptoceratopsidae 011111000?
0001010001010001001010002111100101100001011011001000200101110000 01?01000[12]10?100111011010?
01111011000[01]410111111111000011111?102?03?00?000111011000000000100010 00010?
121111000000111110010001013120100111100011[01]100000100001
Yinlong_downsi 111011?0010??0?00011010??10?1000002101001000000000000 11000?000002001010101 10100?
00112????00101001010001100101100031011?0?0??000?11110???????????????????????????????????001001100?1?
110????????111000121010?2120?????????????0???000??????
Tianyulong 000??1101??0011000?1000??10??????00????00?????????????0????????
2???????????????????????????11?00???010110000?000050?1??0?0??010?10120????????????????????1??
00?????????????????????????00010?1110??????10?????1001???01???01100000?????
Changchunsaurus_parvus 000??1?0011?110000?10???010?10?0??00?00110???100???11000000???0????0??
000????000?0000?0001110110101011111000000 1101101[01]00?0000111100101?1??????????1?010?0?????????
0?????????1?1?1?????????????????????131?????1?1?01???01?000010000?
Jeholosaurus 0?0??110011010?00011000??10?10000100?0001000010000011000000 111010000??0000100?00?
001???00111000010001101000000001011?1100?0000111100001??30000?0?1??1001000????????01000000?
11101100001101 10??01???10131200001111010??01?0000??0001
Haya_griva 0?0??10001?01101101100100100101000000000101001001100000011 1010000??
000010000000 0?001001110?1010001101100000011011011 00?000011110010101 31000?0?1010010000?00???0?
01000000?121100000001?110?001111101312000011?1000??0100000000001
Isaberrysaura 0?????000???000??01?000001101101?0001000000000000?0111?00???????001?00?

000???????????????0????????1?????????????0[01]101100100?0???111????????3??????????????????????????100?
00?????1001??????????????????????????????????????1?00????????
Kulindadromeus_zabaikalicus ??????1????1?0??????001?01?010?001???0000000000000011000??0011?000?00??
000??????????????01??????1000?1?10?00000???11000?0?000011110??????????0??????1010?01????????010?0?
0??121100000??1?110?00111??01??20???1???000??00??000?00??1
Koreanosaurus
??
?????????????????????????????0?????????????10010?01????????0???0??????111?0?????????????????
0131200011?????????0??000?000?1
Yueosaurus
??
????????????????????????????0??????00?0?????000?00?????????????????????????0?0???110???????10???20???
1????????000?000??000?
Albalophosaurus ?????????????????????00???0??1010?????0?00????????????????0?????0?????????000?????
02?????
0???
?????????????????????????????1?????
Laquintasaura   ?????1?????????????1000??0?????????1??000000?????00??????000???110?00?????000?????
0????????????????????????????000?3100000?0??01?1??????0???????0?????000???????????01???00????
0000000011111???01?00??02020001101??????0??????0?0?01
"; "

Ccode
-[/1 0 -[/1 1 -[/1 2 -[/1 3 -[/1 4
-[/1 5 -[/1 6 -[/1 7 -[/1 8 -[/1 9
-[/1 10 -[/1 11 -[/1 12 -[/1 13 -[/1 14
-[/1 15 -[/1 16 -[/1 17 -[/1 18 -[/1 19
-[/1 20 -[/1 21 -[/1 22 -[/1 23 -[/1 24
-[/1 25 -[/1 26 -[/1 27 -[/1 28 -[/1 29
-[/1 30 -[/1 31 -[/1 32 -[/1 33 -[/1 34
-[/1 35 -[/1 36 -[/1 37 -[/1 38 -[/1 39
-[/1 40 -[/1 41 -[/1 42 -[/1 43 -[/1 44
-[/1 45 -[/1 46 -[/1 47 -[/1 48 -[/1 49
-[/1 50 -[/1 51 -[/1 52 -[/1 53 -[/1 54
-[/1 55 -[/1 56 -[/1 57 -[/1 58 -[/1 59
-[/1 60 -[/1 61 -[/1 62 -[/1 63 -[/1 64
-[/1 65 -[/1 66 -[/1 67 -[/1 68 -[/1 69
-[/1 70 -[/1 71 -[/1 72 -[/1 73 -[/1 74
-[/1 75 -[/1 76 -[/1 77 -[/1 78 -[/1 79
-[/1 80 -[/1 81 -[/1 82 -[/1 83 -[/1 84
-[/1 85 -[/1 86 -[/1 87 -[/1 88 -[/1 89
-[/1 90 -[/1 91 -[/1 92 -[/1 93 -[/1 94
-[/1 95 -[/1 96 -[/1 97 -[/1 98 -[/1 99
-[/1 100 -[/1 101 -[/1 102 -[/1 103 -[/1 104
-[/1 105 -[/1 106 -[/1 107 -[/1 108 -[/1 109
-[/1 110 +[/1 111 -[/1 112 -[/1 113 -[/1 114
-[/1 115 -[/1 116 -[/1 117 -[/1 118 -[/1 119
-[/1 120 -[/1 121 -[/1 122 -[/1 123 -[/1 124
-[/1 125 -[/1 126 -[/1 127 -[/1 128 -[/1 129
-[/1 130 -[/1 131 -[/1 132 -[/1 133 +[/1 134
-[/1 135 +[/1 136 +[/1 137 -[/1 138 -[/1 139
-[/1 140 -[/1 141 -[/1 142 -[/1 143 -[/1 144
-[/1 145 -[/1 146 -[/1 147 -[/1 148 -[/1 149
-[/1 150 -[/1 151 -[/1 152 -[/1 153 -[/1 154
-[/1 155 -[/1 156 -[/1 157 -[/1 158 -[/1 159
-[/1 160 -[/1 161 -[/1 162 -[/1 163 -[/1 164
-[/1 165 -[/1 166 -[/1 167 -[/1 168 -[/1 169

```
   -[/1  170      -[/1  171      -[/1  172      +[/1  173      -[/1  174
   -[/1  175      -[/1  176      -[/1  177      -[/1  178      -[/1  179
   -[/1  180      -[/1  181      -[/1  182      -[/1  183      -[/1  184
   -[/1  185      -[/1  186      -[/1  187      -[/1  188      -[/1  189
   -[/1  190      -[/1  191      -[/1  192      -[/1  193      -[/1  194
   -[/1  195      -[/1  196      -[/1  197      -[/1  198      -[/1  199
   -[/1  200      -[/1  201      -[/1  202      -[/1  203      -[/1  204
   -[/1  205      -[/1  206      -[/1  207      -[/1  208      -[/1  209
   -[/1  210      -[/1  211      -[/1  212      -[/1  213      -[/1  214
   -[/1  215      -[/1  216      -[/1  217      -[/1  218      -[/1  219
   -[/1  220      -[/1  221      -[/1  222      -[/1  223      -[/1  224
"   -[/1  225      -[/1  226   ; "

"Hold 50000;"

"proc/;"
```

Supp C2. TNT file containing the dataset of Butler et al., 2008 after Baron et al., 2016 plus coding for *Isaberrysaura mollensis* gen. et sp. nov.

```
"nstates 8 ; "
xread 'Data saved from TNT'
228 55
Euparkeria_capensis 0000??000000000?00000000000000????000?0000000000000000000000000100000??000000100000000?
0000??????0000000000000002000000?00?1001000000100010000000?100011000?00???0?10000000??2010000000000000?
000???0000?000000000000000000?1?000?000
Marasuchus_liloensis_ 0?0?????????????????????????????????????????????????????????????????????????00?
0?????????????????????????????????????????????????010?0?00000?0????????000????????10000010??200000000000000?
000???000101000000101001000?0?000?000
Herrerasaurus_   0000??000000000?11010010000000????000?000000001000000000000000000100000??000000100?000?0?
0000??????0000000000000002000000?00?10010000000201000000000????11100011010011000000000?0100000010000000?
000???00010100000000000000000?00001?000
Pisanosaurus_mertii 0?0??????????????????????
1?????????????????????????????????????????????????????????????????000?10?000000????1??0?00??00?11110??
0?????????????????????????????????????????????????????0???????????????0?0?0????0???0001??000
Abrictosaurus_consors 0000??10011??011000?100??0100100000?0??????????????????0????????????????????????????????
011000?1000011000?00?0030011?0?0??001?11110????????0????????0?10000?10??01?01000000?11?100??????????????????
0?2?200??1???010110???0001?11??
BMNH_A100        0??0??1?0??1?01100??100?00100???????0?0??00?00???????????????????000??????0??????????????
0???????0000?????????0030111?0?0??
011111110???????????????????????????????????????????????????????????????????????????????????1?????
Echinodon_becklesii 0??0????0??1????0????????01??????????????????????????????????????????????????????????
1??????0000??0????0??0[34]??1100?00?00?
011110?????????????????????????????????????????????????????????????????????????????????????
Fruitadens_haagarorum 0??0??1?0??1??11?????0??
001??????????????????????????????????????????????????????????????????0000??0???????03101100?00?011011110??
0???3???????????????0001??????????????????????????????????????????00212000111??????????????011110
Heterodontosaurus_tucki 0000??11011110110001100100100100000000?00100100?0000001000000000200000??
000000000000000?00011000?10000110000001003011111011[01]111111011?001?03?00000010?
0110000110100110100000?111100000000?110?00100010021200001??0010110100000111110
Lycorhinus_angustidens 0????????????011????100??
010????????????????????????????????????????????????????????????????????????????????????11?0?0??0??
111110?????????????????????????????????????????????????????????????????????????????????????1?????
Eocursor_parvus  0?0???????????????????????????????????????????????????????????????????0000??????????????00??????????????
10000100010000????11?0??0?00?01?110??0?0?2???????????0010000??01?01?01?00000?1110000000100110?
0010001002020000001???0?????1??0000000?1
Lesothosaurus_diagnosticus 0000??100011000?000?1000001011000000000?00000000010000001000000000000000??
0000000000000?0?000100000001000010001000000101100?00?000011110000?0120000?????00100000000?00001000000?
010000000011111 0?001000100202000111001?110100000100001
Scutellosaurus_lawleri 0?00????0?11????00????????10???????10?000000000100??????0??0????1100??????????????????????
1????????0000????1?0?000?01100?00?0?00111100?01012??0?1????????1?000?00??00001000000??1?0??0000000110?0010??
10020200001???0????01??110000001
Emausaurus_ernstii 00?0???000?1000?000?1000001001010001 0?0000000010000001000?00000?00000??0000????00?00?????
1?0?????110001000100000 1101100?00?000011110??????????????????????????
01?????????????????????????????????????????????????????????????0???11?0?????
Scelidosaurus_harrisonii 0000??100???0?0????01010001001 1?2?010?000010001000000100000000000100000??000000?
001001?10001?0?????11000101010001011 01100?00?0000111100000022000000010?0010000????????01001000?
00000000000001110?011000100202100011100001111001111 00001
Stegosauria      0000??1000110[01]0?0000100000100 11?2?010?000000000000000110001100001 00000??0000[01]0100100001?
000100000001100110000000 0[01]?101100?00?000011110110[012]0[012]2000100010?0011000?010100001111000?
00000000020 01110?0[01]1010110??01000111?121111[01]0011000?001
Ankylosauria     0000??100011000?000011?00010011?2??10?0000110000000 0?1?10010000101?10??100000?
0010000110011 0000?1110001010000010?101100?00?00001111011000?[12]000000010100?1000?0101000111 11000?
000000100[02]001111[01]111000110??01000111?121111100111[01]0?0?1
Agilisaurus_louderbacki 0000??100011000?00011000?010?10111000?00000000000000?100010000010 0000??000010?
00000??0?00010?0000100011010000000 1101100?00?020011110010101 2000000010?1010100????????01000000?
0100010000110110?001010100202000011100101101 00000100001
Hexinlusaurus_multidens 0?00???0??????????011000??10?10000100?00000000000000?1000?00000?00000??
00001??????????????0???????1000110????000????1101000?0?001111001010120000000 10?1010000000000000001000000?
110101000001 1110?0011111002020000111101011010000000 0001
Othnieliosaurus_consors
0?????????????????????????????????????????????????????????????????????????????????????????????00???????0?0????
1110?00?0???1?1?0??0?013?000?011010010000????????01000000?1111110000011110?00111110131200001111010110 1?
0000?00001
Anabisetia_saldiviai 0?????????????????????????
1?????????????????????????????????????????????????????????????????0101????????0????11?110??????
111????????????????????00100100000??0001100000?11111100010 11110?0010111013120101111?01?1101?0000?00?0?
Gasparinisaura_cincosaltensis 000????????????10????1010??10010000100?0000000000000?01100?000?0??00?0??
```

000000000?????????0????????0001101100010????111111??000?111100?0???3100??1?10???00?00????????01000010?
1111110000011110?001111101312000011110201101100000?00001
Hypsilophodon_foxii 0000??10011101100001100100100100001000?0000000000000001100000111010000??
000010000000100?
0001110000100011011000100110111l[01]01000001111001010131000001101001000000000000001000000?
1211110000011110?0011111013120000111100011011000010001
Orodromeus_makelai 0000??100011?10?00011010101001000000000?0010000000010011000000000010000??
0000100000000?????01???0?010001101100000011011 00?00?00001111001010131000?0?1010010?0000000000001000000?
1211110000011110?001111101312000[01]11110001101?000010001?
Parksosaurus_warreni 000????0??????10???01010??10010?0?100?0000001?00?000?1100?000?00?0000????00???????0??????
0????????000110???0010????111?100?000011110????023?00?0111?10000000????????01000000????1??000001111??
0011111013121 00?1???0001101100001?0001
Thescelosaurus_neglectus 0000??1001110110000?10101010010010100?000000000000010011000?00?11000000??0000?
00100001????011100101000110112000000101101101000001111001010231001?0110000100000000000001000010?
1111011000011110?0011111013121000111100011011000010001
Yandusaurus_hongheensis 0???????????????????100000100??????00?0000000000?????????
0??????????????????????????????0?????????????????????????111?10??0???11110??0?0????????????0010?
00??000?????????0??????10????
Zephyrosaurus_schaffi 0??0??1?01110?10000?10??1010?10100?00?00100000?0?100??0??100???010000??00?
0100000001?????0?????????????????????0110111?100?00?
011110???1?????
Tenontosaurus_tilleti 0000??11110100??0010101001100100001000?00000000000100011100000111000000??0000101100001?
0?0001101000101011111200001???211100101000111100012023100110010100000000000110001000010?
111111000101111100010111113121111111100011011000010001
Tenontosaurus_dossi 0000???1110100100010101001100100001000?00000000000100011100000111000000??000010110??0??0?
0?01101000101011 1???00000???211100101000111110?0120?3?00110?1010000000000???0001000010?1111110001011110?
0010111?13121111111?0001101100001000 01
Talenkauen_santacrucensis 0?00??101??1?010000?10???
0100??01110?10100011?????0?001???1??10???0??11110?
00102????????10?0010010????????01000010?1111?1???????11??0010111?1312????111?000?101?0000??00??
Rhabdodontidae 0000??10010100??0000101000100?????100?0000000001100011100 0000?0200000??0000101000?01?????
011000101010111??000001???21111010100011110?11?0?3?001?0?10??000?00????????01001000??
111011010001??????????11312111?111???????11?000100001
Dryosauridae 0000??11110110??001010100110110001000?000000000100001110000011?000000??000010110000100?
000110101010001111000000 1???2111011010001111 0?1110131001000101 0000000????????0100001 0?1111111011011110?
00101110131201111111020110100000100001
Ankylopollexia 0000??11110100??00101010011001000000000?000000000100001110 0000[01]10000000??000010110000200?
00011010101010111110001 01???2111011010001111 1011[12]02[23]?00100010[12]00[01]000000001100010000[01]0?
11111110110111110001011111[23]121111111100001101020001 00001
Micropachycephalosaurus_hongtuy 0???00???
2??11?0????0???111?0????????????0???????????????????01?000??????
1?1??????????????????0131?0?0?????????????????0?????
Stenopelix_valdensis
0???
??????????????????????3?00??0???????1???????????01001100?11110102000011111?00121?10131?????1???000110100000???
001
Wannanosaurus_yansiensis_ 0?????????????????????????????11????00?000101000000???????????????
0001001101??????????????0???????000010010000 00????11?0??0?0???11110??0???????????????????111?????????1?
111??????1?????????????????????0????01??1???????????????0001?0
Goyocephale_lattimorei 0??0???001???01100001?????10?11?1??00?0001?10??????1????????????0000100111110?????1?1??
0?0???????00001?010?0100031111?[01][01]00?01??111?00???1?2??????????2????111????????
0101110101211?????????????????????????????1??1?????011100010????
Homalocephale_calathocercos_ 0??0?????????????????11????10?11?1??00?000111000000?1110000000000000001100111110?
000110?0??10????????????????????0?????11?110??0???11110????1?3111??0???2????????????????
01011101112111111000011111101311101311 0?0?11?10?????1110001?010?
Pachycephalosauridae 0000??100111001[01]000011???010011?1??00?000111000000?11100000000000 1?1100111110?
[01]0011010?[01]10???????00001001000 10003[01][01]11?[01][01]00?0[12]?0[01]1110????1?3?11??0???20110111????????
010111011121111111 00001???????????013110?0????????????1110001001?1
Chaoyangosaurus_youngi 01?1001?00?0?000110?1????011???????2100001????000????1000?000002????????????????0??0??0?
0?011101?0100011010011000 4101110?00?000011110??0????????????????
1??1?????
Psittacosauridae 01110011001000??11001???0011010100?21010000000100000110000000002000010100000[01]000000010?
10011[01]00001001111[01][01]000001???11111110000011110010[01]003100?0001110[01]1000000000000001000000??
211[01]10000001111100121010[01]3120100111000011010000 0100001
Liaoceratops_yanzigouensis_ 00?1111100100010000 11000?0110?????02111000101100001011000001000200101110 00001?
010001?0?
00011001001001111100110003101111111110000 11110??
????????????????????????????1?????

Archaeoceratops_oshimai 001111100010001000001000?010010100021111011011000010?1011?0100020010111000001?01??
01?0???0111011010001111011100031011???1?1000011111????103110??0??????????????????01000000?1211110?
0000111110012101?131????11110001101000001???0?
Unnamed_taxon__Coronosauria___L 0011111000?
000101000101000100101000211110010110000101101100100020010111000001?01000[12]10?100111011010?
01111011000[01]410111111111000011111?102?03?00?00011101100000000010001000010?
121111000000111110010001013120100111100011[01]100000100001
Yinlong_downsi 0111011?0010??0?00011010??10?100000210100100000000000011000?00000200101010110100?00112????
00101001010001100101100031011?0?0??000?11110????????????????????????????????001001100?1?110????????
111000121010?2120????????????0???000??????
Tianyulong 0000??1101??0011000?1000??10??????00????00????????????0????????2??????????????????????????11?00???
010110000?000050?1??0?0??010?10120???????????????????1??00????????????????????????00010?1110??????10?????
1001???01???01100000?????
Changchunsaurus_parvus 0000??1?0011?110000?10???010?10?0??00?00110???100???11000000???0????0??000????000?
0000?000111011010101111110000001101101[01]00?0000111100101?1??????????1?010?0?????????0????????1?1?
1????????????????????131?????1?1?01???01?000010000?
Jeholosaurus_shangyuanensis 00?0??110011010?00011000??10?10000100?0001000010000011000000111010000??0000100?
00?001???00111000010001101000000001011?1100?0000111100001??30000?0?1??1001000???????01000000?
1110110000110110??01???10131200001111010??01?0000??0001
Albalophosaurus_yamaguchiorum 0????????????????????00???0??1010?????0?00????????????????0?????0?????????000?????
02?????0???????????110????0?0????111111110???111?
0??1?????
Haya_griva 00?0??10001?011011011001001001001010000000001010010011000000111010000??0000100000000?
001001110?101000110110000001101101100?00001111001010131000?0?1010010000?00???0?01000000?121100000001?
110?001111101312000011?1000??0100000000001
Koreanosaurus
0??
?????????????????0?????????????10010?01????????0???0??????111?0?????????????????013120011??????????0??000?000?1
Yueosaurus
0??
?????????????????0??????00?0?????000?00?????????????????????????0?0???110???????10???20???1????????000?000??000?
Laquintasaura 0?????1?????????????1000??0?????????1??000000?????00??????000???110?00?????000?????
0???????????????????????????000?3100000?0??01?1??????0???????0?????000???????????01???00????0000000011111???
01?00??02020001101??????0??????0?0?01
Isaberrysaura 00?????000???000??01?000001101101?0001000000000000?0111?00???????001?00?
000???????????????0????????1?????????????0[01]101100100?0???111?????????3??????????????????????????100?00?????
1001????????????????????????????????????1?00????????
"; "

Ccode
-[/1 0 -[/1 1 -[/1 2 -[/1 3 -[/1 4
-[/1 5 -[/1 6 -[/1 7 -[/1 8 -[/1 9
-[/1 10 -[/1 11 -[/1 12 -[/1 13 -[/1 14
-[/1 15 -[/1 16 -[/1 17 -[/1 18 -[/1 19
-[/1 20 -[/1 21 -[/1 22 -[/1 23 -[/1 24
-[/1 25 -[/1 26 -[/1 27 -[/1 28 -[/1 29
-[/1 30 -[/1 31 -[/1 32 -[/1 33 -[/1 34
-[/1 35 -[/1 36 -[/1 37 -[/1 38 -[/1 39
-[/1 40 -[/1 41 -[/1 42 -[/1 43 -[/1 44
-[/1 45 -[/1 46 -[/1 47 -[/1 48 -[/1 49
-[/1 50 -[/1 51 -[/1 52 -[/1 53 -[/1 54
-[/1 55 -[/1 56 -[/1 57 -[/1 58 -[/1 59
-[/1 60 -[/1 61 -[/1 62 -[/1 63 -[/1 64
-[/1 65 -[/1 66 -[/1 67 -[/1 68 -[/1 69
-[/1 70 -[/1 71 -[/1 72 -[/1 73 -[/1 74
-[/1 75 -[/1 76 -[/1 77 -[/1 78 -[/1 79
-[/1 80 -[/1 81 -[/1 82 -[/1 83 -[/1 84
-[/1 85 -[/1 86 -[/1 87 -[/1 88 -[/1 89
-[/1 90 -[/1 91 -[/1 92 -[/1 93 -[/1 94
-[/1 95 -[/1 96 -[/1 97 -[/1 98 -[/1 99
-[/1 100 -[/1 101 -[/1 102 -[/1 103 -[/1 104
-[/1 105 -[/1 106 -[/1 107 -[/1 108 -[/1 109
-[/1 110 -[/1 111 +[/1 112 -[/1 113 -[/1 114
-[/1 115 -[/1 116 -[/1 117 -[/1 118 -[/1 119
-[/1 120 -[/1 121 -[/1 122 -[/1 123 -[/1 124
-[/1 125 -[/1 126 -[/1 127 -[/1 128 -[/1 129
-[/1 130 -[/1 131 -[/1 132 -[/1 133 -[/1 134
+[/1 135 -[/1 136 +[/1 137 +[/1 138 -[/1 139

-[/1 140	-[/1 141	-[/1 142	-[/1 143	-[/1 144
-[/1 145	-[/1 146	-[/1 147	-[/1 148	-[/1 149
-[/1 150	-[/1 151	-[/1 152	-[/1 153	-[/1 154
-[/1 155	-[/1 156	-[/1 157	-[/1 158	-[/1 159
-[/1 160	-[/1 161	-[/1 162	-[/1 163	-[/1 164
-[/1 165	-[/1 166	-[/1 167	-[/1 168	-[/1 169
-[/1 170	-[/1 171	-[/1 172	-[/1 173	+[/1 174
-[/1 175	-[/1 176	-[/1 177	-[/1 178	-[/1 179
-[/1 180	-[/1 181	-[/1 182	-[/1 183	-[/1 184
-[/1 185	-[/1 186	-[/1 187	-[/1 188	-[/1 189
-[/1 190	-[/1 191	-[/1 192	-[/1 193	-[/1 194
-[/1 195	-[/1 196	-[/1 197	-[/1 198	-[/1 199
-[/1 200	-[/1 201	-[/1 202	-[/1 203	-[/1 204
-[/1 205	-[/1 206	-[/1 207	-[/1 208	-[/1 209
-[/1 210	-[/1 211	-[/1 212	-[/1 213	-[/1 214
-[/1 215	-[/1 216	-[/1 217	-[/1 218	-[/1 219
-[/1 220	-[/1 221	-[/1 222	-[/1 223	-[/1 224

"   -[/1 225   -[/1 226   -[/1 227   ; "

xgroup

"; "
agroup

=0 (Wildcard)   6 20 26 51 52
=1 (Stable)   0 1 2 3 4 5 7 8 9 10 11 12 13 14 15 16 17 18 19 21 22 23 24 25 27 28 29 30 31 32 33 34 35 36 37 38 39 40 41 42 43 44 45 46 47 48 49 50 53 54

"; "

"blocks 0; "

"proc/;"

Supp C3. TNT file containing the dataset of Boyd, 2015 plus coding for *Isaberrysaura mollensis* gen. et sp. nov.

```
"nstates 16 ; "
xread 'Data from Salgado et al.'
255 66
Marasuchus       ?????????????????????????????????????????????????????????????????????????????????????????
0???????????????????0???????????????????????????????????1?0?0?????1????0?????0????????????????001002??
00?02???012?0?0?00??000000001?00?000?230?0?0020?0???002000?00?0??
Silesaurus       ?0000000?00?1?1?000??00????0???0?1??00??????101????100??00????00111??????40??00000?0??
1000???0?0000??0??000?2001001?0?0001??3?0?10030003????00210021001??????0000???0?020?00?????????
0000002?000002???01000?01001?00?000001?00?011?21000?0020?????0011??000?000
Asilisaurus      ?0???????????????0??????????????0??????????????02???????????????????????????
4????????????????????????????????????????0????????????3????0??0003???????100?00?00?????0000????
0???????????????00000???????02???0???0?0110???0?000000?0??01??2100??0020????????1???00?0??
Sanjuansaurus    ?????????????????00?00?????
0???????????????????????????????????????????????????????????0??????????????????????????0??10?0?2?001?
04?0??????00??0?000????????00001??????2??????????????11?????????02???????????????010??????1?00?001?20?
0??0021?????????????00????
Herrerasaurus    10000000?00010?0000?000??00020?0101000??0100102??0??100000100000??2?????010?
0000000010?0100010000??0?0010?????0100?10??10?0?2100100410?21???00?10000???0?????00?0????0???2?
0000?000022011000 2?000002???100?0?100000100000001?00?001120100?0021?10??002100000?000
Tawa             1000000??000???0000?000??0002000000000??00000???????00?100000???????????010?
00000000???100?0100000???0?????????0200?10??10?0?2000100410?2???????10??0?????????00?0?????01????00?
0?011?0010?02???0?02???11????1??00010??00001?0???0??2110??0021?????00?100000?000
Pisanosaurus     ??????????????????011????????????????????????????????????????????????????????0011?
10000????????????????????????????????1??0??????10??100???1?00?????0??????????????????????????????????????
0???????0???????11?????0??????????????????????110???001????????????0???00?
Heterodontosaurus 010001111000 1?010011001010001000000003??000000000?0?00?010000?000110?0100?
00101101000000001000000 0?00?100?0?2002110120?11?110011101?11000?110?100002??0000100001????
0001200000000000001100101?00?2000010000?011000000101212?00?000??2100??????00000??100001?00?
Fruitadens       ????0?111????0??0111????????????????????????????????????????????????????0?0??0??
0?????????????????????????????0210?01??0??0?0?00011?0??0?00??0?????
2????????????????????????????????????????????????????????????????0???0?101212?00?00???210???
0010??????????????????
Echinodon        ?1??0????????????0011????????????????????????????????????????????????????1?000?
10110?????????????????????????????????020??11??0??0?0?00011200?0??
0????????????????????????????????????????????????????????????????????????????????????????????????????????????
??????????0?
Lycorhinus       ???????11???????0011?
0?????????????????????????????????????????????????????????????????????00???????????????????1???1??00???
0?000?13?
1????????????????????????????????????????????????????????????????????????????????????????????????????????????
?????????????????
Tianyulong       010001111?001010??1???????0?20?0???000????????????????????????????????1?0???01?10110110??
200???000?0??????????????0211?11??0????0?00011????0??0???????????????????????????????
102??????????????????????????2??????010?0?0???????????????1000??????????????????0??100011000?
Abrictosaurus    ?10?0111100?1????01100101????0?0??????????????????????????????????????????101?0?0??01101?
0?????00?????0??????????????0200?11??0??1?00000113?1?01?01???????1??????????????????0000????????0?????
1100101?00???????????0????????????0?1112?00???0??2???????????????0001?00???00?
Eocursor         ???????????????????1???????????????????????????????????????????0????????????????0???
000101000???????????0???1????????????0??????????0??????1??0000?00?????????1?????????00?????0?00????????
0????01100101?00?2000010010?00?00000?000102000?0001121000????????????????????00?
Lesothosaurus    010??01101?110??01101010?00010?0?000????000?0000??0000?000000000010???0010?00001??
00010??00???000??01?1000?0100000?011000000010?010??000?0?0????0??????0??1????10????1??000?00?0????
100000100000?2000010010?00000?00000010200????0??2??0?3?010110?????0???1?00?
Scutellosaurus   ?1000??10?0??????110??????0???0110??00???1??00?001?000?000000?00?1???????00000010????
11?0??????0???0??????????00000010?0000?0?0001020000 2000001 1?011??0??????0?1000??0000??00???00?0??
0100100?00?200??11?10?00000000000000102000?000??2100?0?????????0???00??1?10?
Scelidosaurus    ?1???0?0??0?1????1110????????0?1?????????0????????????????00??????????????1????101?????
```

11???0?????????????????????????0??????0??????????0?????0????0?????????0?0????????0???????????????001??
110?00?????0???00??1?????0?0?010??0????1??2??????????????????????1?11?
Emausaurus ?1???0?0?0??1????1110????????0?1????????0????????????????0????????????0?1????101?????
11???0?????????????????????????0??????0??????????0?????
0???
????????10?
Stormbergia
??
???????????????????????????????????0??0?????00??1??001000??1??1??1?????????00100100100?20000??
011001000000000010??00?00003210???001??????0????0?1?0??
Agilisaurus 01001010??0?1?100?1100200111011?010000??0100100??10??0?000000000011???001?0?
1011011000100010000001??000??1?????01000010?00?0???10011200?0200001??1?11??0?1?1?0001000?01100?
0000??000??10100100101?21?2110011000000?100001102?00?0001?2??000????????0001000011000
Hexinlusaurus 01?????????????0??11??1011001100?0?0?0??01??000????????0101?0?0001??????
12010011?????????0?00?00??????????????????0??1??00?000?10011300?0200?00101011??002????0010???0?1000?
000?0000??01000101101?20021100010??0001000000102?00?00012210100001?1111001100000?00?
Yandusaurus ?1????????????????11?????????????????????????000?????
00????????????????????????????????????00??????????????????????1??00?0001100?13?0????0??
0???????????????01000??0?1??0?00??????
1????????????????0??????
Leaellynasaura ??????????????????11??????0??10001?000??????000?0???00????????00?
1???1???11??0?110?13?
0??2003?1000100?0000?0?101202?0??000??210??
00010????0001000??????
Jeholosaurus 0100001101011?1101110110010021100100001??
110000000101001010100000011100001201001111010000010000001??0?0001?1?000001?010?1????0110011200?
010000010?11200001????00??0??010000000????????010001011110200?1100?1?000010110001212?00?
00013210?000011111100??000?1100?
Yueosaurus
??
????????????????????????????????????0010???????0?????00?????0?002?????????????????????????????????0??????
00?11??0121200???????2??????????????0????00??????
Othnielosaurus ???????????????????????????0??????1???????????????????????000??00??????0?1?10?
11???????????????????10????00?1?????1???0000?000?1001?200001?0?0010??120??11???100100??0?100?0?00?
00??0001000101111020021101?100?0001110001212?00?00?0?2101?21011111110001000???00?
Parksosaurus ???????10???1??0?1110?????00??101100?0??1?0??00??????01?????0???????????????0?01101?
0?????01011001?00?????1???0???1??10?0???00?10?11?0?10?00??0?1?0????001???100?00000???1?
00???????????001?0111??2????1120110100101110121201???001?210100001??1??000100011000?
T._neglectus 11001011010111100111012001002110001000?0A1000000011110101010110?
101110000112011011111012?000101000110 0?1011??0010001?010100100011001120000211000111 0?3?0111??
0100?0??20010000000?000000010101011111200211000110100101110011212?
110001022102000011111000010001100 01
T._assiniboiensis ????????????????????????0??????????????????????????????????
010001011?????????????????????????????????1001011110000????????????????????1???????????0?????????1???
2??????????????????????????010?11?1111?21021?????????01011100121?011000102210?00001?11?0000100011?
00?
T._garbanii
??
??
???????????100??02?10?00001?11100001000??????
Talenkauen ?1001?110?0???100?11?0??100011?0?1?
11???????????????????????????????0?01??1???????0?11??1???????1?00?10?2???????????????????0??2??????????????
0?01010??1??20?1???????1??0???????12?2?1????1?????????????????0??1????1????
Notohypsilophodon
??
?????????????????????????????????????01?????00???????0??0?????
120??11?00121???0?00???210??0???????????????0??????

Macrogryphosaurus
??
???????????????????????????????????10?10102?0??????1??????????2?????????????010101?1111120011???0??
111???
Haya 0100001101001110?11101200100?1?0010000??1100000???1??0?010110?0011000001111101 1?1?
00??000?110001??000101?????0101?010??020?0110011?00?01?1000?0???2??0111??000101000?001????
0???????01000121?11220021??00100??000111001212?00?000????????0?1?11???001000011000
Changchunsaurus 01001011010111?00111?1?0??00???001?002??0101??0??1???000?0000??????10001111?
10111100120000?0??001??0????1???1?0100?010?01?0?0?10011200?0?01?0010??1????????000?0?00??
10??????????????1?????????????????????????011?00121?????????3?10???????????0??1000?1?001
Oryctodromeus ?1001?1??10111??01????????0????0?1???0??????0000??01000?10??????????????1??1??11????
1????????00??0000??11?1??0?00???0000??????0?1??00?0?01??021?11310?121?2?101001??0101000????????
1100012?111220021????????001111001212?0100000?210?100??????0001000111001
Zephyrosaurus ?10?0?110101?11?011111101?10?110?1?00300???0000000010011101100001?????????0???
11???0??1???0???00?1100?1011112001???0100001000 1100?12?00??????020?0?????11??0?1?100???????
0??????????????001?????2???????101?1????11?10012??????00?0?2??11002101 1100??1000???001
Orodromeus 010011110?0011?00111?1101100210011?00300A10?00000?0110111011010 00???????
12111011010010 2?001000001100101011111?010??0100000000???0112?000????002010130000 10?0?101000???
1111000???0000?010001211112200 2?1000100?0000111?01212?01000000?1011200101110000 10000??000
Kaiparowits_Oro. ?1??????????????????????????????2??1???3??????????????????1????000????????1?????
11????????????????0???????0????????00??100000??0?0?011?0000?01??021??????????????1??0?1???111???
00000001?????????????2???????????????000111?01212??0?00??2?1001????011110001000???0??
Koreanosaurus
??
???????????????????????????????????????021?0??0???????011101000?1010?????????????1?????111???????11???????
001111001212?00?00013?1011????????????????????0??
Archaeoceratops 0110001100101000?11100101000201010000 1??0111000?010010?0?1010?001011100110?
110111100002000 00?00010???0??1?????0201?01??11?1?0??101141??0??10???0????2?
0011?????????????????????????????0100110111 1?20030????0??????1110?0121???????????2????00110111?0??
1000???000
Liaoceratops 01100011001010?1?11100???0002?10100002??0110000000000?011010?
00001100011011101101 00?0?000????0010?0?0101????10201?01??1??110?10011?00?01?
10???
????????????
Yinlong 011?0011001?1001?11100101100201010000 1??0101111?000?00?0?10?0?
000011000110011011010 01??0001011000??0??101???21020?001??00???010?0?12?
0???01011101??1?20030?????????0??????01212?
0?????????????????????0???000?????0
Stenopelix
??
??2??????????0????????????????????????0100010 1?01?20?3?1?110?
010????1?????1??????????2?????????????000?00001000?
Micropachycephalosaurus ??????????????????????????????????????0????????00????
0?????????????????????????????????????2???0???????????????
0???????????????????????????????????1??01????1??????????????0001110?0121?00??0???3?
10????????????????????????
Wannanosaurus ??????????????????????21??0???10?0???1?0???????????????0?10?0?10??????????0??01101?
000?????????01?????????????????0???1????????????0??00?00?0??0?0????????????????????????10???????????????1???
1?????????????????????00??????1????00???00????????????????????????????
Hypsilophodon 0100011101011010011101101001110011 0000??11000000010000?
0000000000111100012011011011010000011000111?000101100100001?
02001111100100110001010100010101200011110100110000010101 00000000??
01000101111120121100011010001110001 21?00??00003210201001111110011000110000
Atlascopcosaurus ???????????????????11???1???
11??????????????????????????????????????201111?00?111?1?0010?
11???
?????????????

Qantassaurus ??
120110110????????????????????????????????????0?????0?????0?
400100010???
????????????????????
Anabisetia ?????????????????111?0???1???
11???????????????????0????0?????????1??1??1??1?0??11?1??0????0??????????????????00100??0?02???
0?????????01010101??1?20011110010??100?110111212?010000????????????????000100?1?????
Gasparinisaura 01????????????????110?100?0111?0010010??01000001110????000000?0?0???????120?1011?
100100??0101?001??010100??2?0??0???1??11?1?00111?10?0?000?0??1????210?1101??0?100?????10?
00?????????0101010111102002111?01?01?00?111111212?00?0000221010002111211?01?1110?1000?
Z._robustus 110001??010?1?0?01110????00021?0?01?10??011000111110010?0100110011021100110 1?
1011111010201??01?101??000200??1?01?011?201111111?1000110011011??010?0?320101????00?0?????110?
1?????????1110110??1???????11100?1101101110012120110101 1?2101?00210?????1????????000
Z._shqiperorum ?1????????????????????????00021?00010?0??????0??11??011?0?00?10?111??1???1010101111?
0102???????10???0?0200???????01???0?11111?????01100110110?0?0?0?32?1??????000010???110?1??????????
1110110?111?2002111100?1111101110012120111101 1?210??0???0?????????????1000?
T._dossi 11002?110?0?1??11?11?010?????100001000??0100001?1?0??1?010??10?01??111?0121?101111?
01??01010111010?01???1????00201???0111111?0001?11?011001011?0?1121011????000100001?1100100???
10???11010101?11?21?111110100111001100012 12?1111011221030??????11?001100011000?
T._tilletti 1??0???????????1?0????0???001110??1100???1???0??0??00?1001????1001?1?01????101???1?0?12?
11???1?11?0?01???10020?1?01???0111111??001?11?011001011?0?112101110?1000100001?11001000001 01??
110101011110201111100100001001100012120111101022103000211111 00A0100011000?
Rhabdodon ??????????????0??0?????????????????????????????0?????000?0????????????????????111?111??
110?????????????0?????100???????????0?11111??101?11?01??11??0???1??10111????0?000????10101??????????
1??0????1??2??????10?0?1?111?110001???00??1?10???01?012???201?1??1?????00?
Muttaburrasaurus ??????????0?1??0??110?10?001?100000110??0?0??01???1??0???0????1?10??????????????11????
1????1?0????1??0?0??1?????????1??10?11???0?11??1??0????1?011??1?2??????????00?0??????0?10100???????0?
1101?1?1?11?2??111?????????1?011000121201101 01??21?1?0101?1???01?1??0???00?
Elrhazosaurus
??
???
00011000121?101200??????0?????????????????????????
Dysalotosaurus 010020???00?10011111001010011010000110100001001 1?0101010100???000?
111101110110111101 10?1101011001 0000?111102211?0???21????11?1111011?0110010011??112?01121??00?
010?0???11010????10???110101011111211111010101?1010110011212101 1101??2103?31?11??0?00?
1110110000
Dryosaurus 010020???00?100111110010000010100001101000010010?0101000100??1000?
1111011211101111 0100?1101011001 0000?111002011?011?211111111?111011?0111010?111?1?2101120?100?
01?????011000?0??10???110101011110211111 0A0101?1010110011212101010100 2103?310111111?
001110110000
Callovosaurus
??
???
00011000111210 1110??????????????????????????????
Valdosaurus
??
?????????????????????????????????????121???1?????2?12???001?11?
0001100012121012100022113 0???1011?011??1????????
Camptosaurus 1101?????????00?1????????001111??101??010???0??0??1011011?0011101?2?01?1??111???1?
111??11???1?00?0110???0012001?0????1111111??011?11??1?100??0???1??211111?000?110????01101111??
101??1?000???1??0??1???1A?1?1?11001?0001???01111?11???0?0012????0??1??100??1???
Iguanodon 1101?????????0??1?????????0121???100??210???1??1??1011001?0?11111?2?01?1??110???1?
211???1?????0???111???000220??0????1111111??011?11??1?200??1???1??211111?200?210?????1210111??
111??1?010???1??1??????11?1?1?110?1?0101???01131?11???15?012???201?1??111??1???
Ouranosaurus 110???????????0??1?????????0120???1?0??210???1??1??1001?0??0011111?2????0???10?????
111??????????????111???00002???0????1?????1??????1???1???????????1??????????2?0?21????????210?1?????????
1??????????????????1????1?1???0?0?01???0??????1???140012???2???????1?????????

Isaberrysaura 11??11?0????11?0??1100110100202?00?01?????10?01?????????????0?101?
0??????????????????????0101?00??????????????000?001?00010002000111?0?0?
00??0?0001????
1??11001
"; "

"Hold 50000;"
"proc/;"

Supp C4. TNT scripts used to calculate the Templeton tests.

FILE: templeloop.run

```
macro - ;

macro [2000 ;

macro * 15 40000 ;

macro = ;

/****************************************************/

/*This TNT script is am updated version of the script */

/* writen by Alexander N. Schmidt-Lebuhn to           */

/*implement the Templeton Test                        */

/*Templeton AR 1983, Evolution 37(2): 221-244         */

/*This new version of the script allows to chose which*/

/*tree topologies to compare at random from the       */

/*most parsimonious trees                             */

/*                                                    */

/*To run the test, load the matrix on TNT, and conduct*/

/*a normal unconstrained search, saving the resulting */

/*trees to a parenthetical tree file named mpt.tre,   */

/*Then, conduct a constrained search that produces the*/

/*set of topologies to be tested, and save it in a    */

/*parenthetical tree file named cons.tre. Edit the    */

/*templeloop.run script to set the number of          */

/*replications to run (1000 by default) and run the   */

/*script. It will generate an file named results.out  */

/*with the results in the test in csv format. The last*/

/*column of the table will output a 0 if the test is  */

/*non significant or a 1 the test is significant at   */

/*any level an thus the constrained topologies can be */

/*rejected.     M.Moreno-Azanza. Beta0.1 June 2016   */
```

```
/*Here follows the comments of the original script    */
/* This TNT script implements the Templeton Test      */
/* Templeton AR 1983, Evolution 37(2): 221-244        */
/*                                                    */
/* It assumes that the data matrix and two trees      */
/* are in memory, that the first tree (number 0) is   */
/* the better, and that the second tree is tested     */
/* against it. It does not check if there is anything */
/* in memory or if the first tree is the better one,  */
/* that is up to the user. Output is text produced by */
/* the quote command, so you may want to log it into  */
/* a text file.                                       */
/*                                                    */
/* The tables of critical values are hard-coded       */
/* because it appeared to me as if TNT did not have   */
/* the mathematical functions needed to estimate      */
/* them. This means that the script does not produce  */
/* exact P values, and that the test is limited to    */
/* a maximum of 10,000 character differences in the   */
/* data.                                              */
/*                                                    */
/* The script has been tested against PAUP with three */
/* different pairs of trees and seems to produce the  */
/* same results, but it is offered here without any   */
/* guarantee or warranty of any kind whatsoever.      */
/*                                                    */
/* If somebody finds errors in the script I would be  */
/* grateful to be informed. If somebody can change    */
/* the script to test several trees against the first */
/* that would be great, but for some reason whenever  */
/* I added another loop around this script I got an   */
```

```
/* unexpected end of file error and couldn't figure   */
/* out why. I also wouldn't mind being acknowledged   */
/* if somebody used the script for a paper.           */
/*                                                    */
/* Alexander N. Schmidt-Lebuhn                        */
/* alexander.s-l@csiro.au                             */
/****************************************************/



/*generate results table*/
log results.out
quote   Random MPT, Random constrained tree, Sum Negative ranks, Non zero scores, Rank for significance at 0.05, Rank for significance at 0.025, Rank for significance at 0.001, Result,
;
log/;

/*the loop*/

loop 0 1000
p temple.run;

stop

p/;
```

FILE temple.run

/* careful: variables are initiated with value 0 */

```
var:    wilc5[10000]
        wilc2_5[10000]
        wilc1[10000]
        charnumber
        diffs[(nchar+1)]
        nonzerodiffs
        thisdiff
        absdiffs[(nchar+1)]
        diffsrank[(nchar+1)]
        howmanytrees
        swapbuffer
        startrankblock
        rankblocksum
        lookupwilc
        teststat randmpttree randcontree results;
```

/* hardcode Wilcoxon table */

setarray 10000 wilc5 0 0 0 0 0 2 3 5 8 10 13 17 21 25 30 35 41 47 53 60 67 75 83 91 100 110 119 130 140 151 163 175 187 200 213 227 241 256 271 286 302 319 336 353 371 389 407 426 446 466 487 508 530 551 574 596 619 643 667 692 716 742 768 794 821 848 876 904 932 961 991 1021 1051 1082 1113 1145 1177 1210 1243 1277 1311 1346 1381 1416 1452 1488 1525 1563 1600 1639 1677 1717 1756 1796 1837 1878 1919 1961 2004 2047 2090 2134 2178 2223 2268 2314 2360 2406 2453 2501 2549 2597 2646 2696 2746 2796 2847 2898 2950 3002 3055 3108 3161 3215 3270 3325 3380 3436 3493 3550 3607 3665 3723 3782 3841 3901 3961 4022 4083 4144 4206 4269 4332 4395 4459 4523 4588 4654 4719 4786 4853 4920 4987 5056 5124

5193 5263 5333 5403 5474 5546 5618 5690 5763 5836 5910 5985 6059 6135 6210 6287 6363
6440 6518 6596 6675 6754 6833 6913 6993 7074 7156 7238 7320 7403 7486 7570 7654 7739
7824 7910 7996 8082 8169 8257 8345 8434 8523 8612 8702 8792 8883 8975 9066 9159 9251
9345 9438 9533 9627 9722 9818 9914 10011 10108 10205 10303 10402 10501 10600 10700
10801 10901 11003 11105 11207 11310 11413 11517 11621 11725 11831 11936 12042 12149
12256 12364 12472 12580 12689 12798 12908 13019 13130 13241 13353 13465 13578 13691
13805 13919 14034 14149 14265 14381 14497 14614 14732 14850 14969 15088 15207 15327
15447 15568 15690 15812 15934 16057 16180 16304 16428 16553 16678 16804 16930 17056
17184 17311 17439 17568 17697 17826 17956 18087 18218 18349 18481 18614 18746 18880
19014 19148 19283 19418 19554 19690 19827 19964 20102 20240 20378 20517 20657 20797
20938 21079 21220 21362 21505 21647 21791 21935 22079 22224 22369 22515 22661 22808
22956 23103 23251 23400 23549 23699 23849 24000 24151 24302 24455 24607 24760 24914
25068 25222 25377 25532 25688 25845 26002 26159 26317 26475 26634 26793 26953 27113
27274 27435 27597 27759 27921 28085 28248 28412 28577 28742 28907 29073 29240 29407
29574 29742 29911 30080 30249 30419 30589 30760 30931 31103 31275 31448 31621 31795
31969 32144 32319 32495 32671 32847 33024 33202 33380 33558 33737 33917 34097 34277
34458 34640 34822 35004 35187 35370 35554 35738 35923 36108 36294 36480 36667 36854
37042 37230 37419 37608 37798 37988 38178 38369 38561 38753 38945 39138 39332 39526
39720 39915 40111 40306 40503 40700 40897 41095 41293 41492 41691 41891 42091 42292
42493 42695 42897 43100 43303 43506 43710 43915 44120 44326 44532 44738 44945 45153
45361 45569 45778 45987 46197 46408 46618 46830 47042 47254 47467 47680 47894 48108
48323 48538 48754 48970 49186 49404 49621 49839 50058 50277 50497 50717 50937 51158
51380 51602 51824 52047 52270 52494 52719 52944 53169 53395 53621 53848 54075 54303
54531 54760 54989 55219 55449 55680 55911 56143 56375 56607 56841 57074 57308 57543
57778 58013 58249 58486 58723 58960 59198 59437 59676 59915 60155 60395 60636 60877
61119 61362 61604 61848 62091 62336 62580 62826 63071 63317 63564 63811 64059 64307
64556 64805 65054 65304 65555 65806 66057 66309 66562 66815 67068 67322 67577 67832
68087 68343 68599 68856 69113 69371 69629 69888 70148 70407 70667 70928 71189 71451
71713 71976 72239 72503 72767 73031 73297 73562 73828 74095 74362 74629 74897 75166
75435 75704 75974 76244 76515 76787 77059 77331 77604 77877 78151 78425 78700 78975
79251 79527 79804 80081 80359 80637 80916 81195 81475 81755 82035 82316 82598 82880
83163 83446 83729 84013 84298 84583 84868 85154 85441 85728 86015 86303 86591 86880
87169 87459 87750 88040 88332 88623 88916 89208 89502 89795 90090 90384 90680 90975
91271 91568 91865 92163 92461 92759 93058 93358 93658 93959 94260 94561 94863 95166
95468 95772 96076 96380 96685 96991 97296 97603 97910 98217 98525 98833 99142 99451
99761 100071 100382 100693 101005 101317 101630 101943 102256 102571 102885 103200
103516 103832 104149 104466 104783 105101 105420 105739 106058 106378 106699
107019 107341 107663 107985 108308 108631 108955 109280 109605 109930 110256
110582 110909 111236 111564 111892 112221 112550 112880 113210 113540 113872
114203 114535 114868 115201 115535 115869 116203 116538 116874 117210 117546
117883 118221 118559 118897 119236 119576 119916 120256 120597 120938 121280
121622 121965 122309 122652 122997 123342 123687 124033 124379 124726 125073
125420 125769 126117 126466 126816 127166 127517 127868 128220 128572 128924
129277 129631 129985 130339 130694 131050 131406 131762 132119 132477 132835
133193 133552 133911 134271 134632 134992 135354 135716 136078 136441 136804
137168 137532 137897 138262 138628 138994 139361 139728 140096 140464 140832
141202 141571 141941 142312 142683 143054 143427 143799 144172 144546 144920
145294 145669 146045 146421 146797 147174 147551 147929 148308 148687 149066

149446 149826 150207 150588 150970 151352 151735 152118 152502 152886 153271
153656 154042 154428 154815 155202 155590 155978 156366 156756 157145 157535
157926 158317 158708 159101 159493 159886 160280 160674 161068 161463 161858
162254 162651 163048 163445 163843 164241 164640 165040 165440 165840 166241
166642 167044 167446 167849 168252 168656 169060 169465 169870 170276 170682
171089 171496 171903 172312 172720 173129 173539 173949 174360 174771 175182
175594 176007 176420 176833 177247 177662 178077 178492 178908 179325 179741
180159 180577 180995 181414 181833 182253 182674 183094 183516 183937 184360
184783 185206 185630 186054 186479 186904 187329 187756 188182 188609 189037
189465 189894 190323 190753 191183 191613 192044 192476 192908 193341 193774
194207 194641 195076 195511 195946 196382 196818 197255 197693 198131 198569
199008 199447 199887 200328 200768 201210 201652 202094 202537 202980 203424
203868 204313 204758 205204 205650 206097 206544 206991 207440 207888 208337
208787 209237 209688 210139 210590 211042 211495 211948 212402 212856 213310
213765 214221 214677 215133 215590 216047 216505 216964 217423 217882 218342
218802 219263 219725 220186 220649 221112 221575 222039 222503 222968 223433
223899 224365 224832 225299 225767 226235 226703 227173 227642 228112 228583
229054 229526 229998 230471 230944 231417 231891 232366 232841 233316 233792
234269 234746 235223 235701 236180 236659 237138 237618 238098 238579 239061
239543 240025 240508 240991 241475 241959 242444 242929 243415 243901 244388
244875 245363 245851 246340 246829 247319 247809 248300 248791 249283 249775
250268 250761 251254 251748 252243 252738 253234 253730 254226 254723 255221
255719 256217 256716 257216 257716 258216 258717 259218 259720 260223 260726
261229 261733 262237 262742 263248 263753 264260 264767 265274 265782 266290
266799 267308 267818 268328 268839 269350 269862 270374 270886 271400 271913
272427 272942 273457 273973 274489 275005 275523 276040 276558 277077 277596
278115 278635 279156 279677 280198 280720 281243 281766 282289 282813 283337
283862 284388 284914 285440 285967 286494 287022 287550 288079 288609 289138
289669 290199 290731 291262 291795 292327 292861 293394 293929 294463 294999
295534 296070 296607 297144 297682 298220 298759 299298 299837 300377 300918
301459 302000 302542 303085 303628 304172 304716 305260 305805 306350 306896
307443 307990 308537 309085 309633 310182 310732 311282 311832 312383 312934
313486 314038 314591 315144 315698 316252 316807 317362 317918 318474 319031
319588 320146 320704 321263 321822 322382 322942 323503 324064 324625 325187
325750 326313 326877 327441 328005 328570 329136 329702 330268 330835 331403
331971 332539 333108 333678 334248 334818 335389 335960 336532 337105 337678
338251 338825 339399 339974 340549 341125 341701 342278 342856 343433 344012
344590 345170 345749 346330 346910 347491 348073 348655 349238 349821 350405
350989 351574 352159 352744 353330 353917 354504 355092 355680 356268 356857
357447 358037 358627 359218 359810 360402 360994 361587 362181 362775 363369
363964 364559 365155 365752 366349 366946 367544 368142 368741 369340 369940
370541 371141 371743 372344 372947 373549 374153 374756 375361 375965 376571
377176 377782 378389 378996 379604 380212 380821 381430 382039 382650 383260
383871 384483 385095 385707 386321 386934 387548 388163 388778 389393 390009
390626 391243 391860 392478 393096 393715 394335 394955 395575 396196 396817
397439 398062 398684 399308 399932 400556 401181 401806 402432 403058 403685
404312 404940 405568 406197 406826 407456 408086 408717 409348 409979 410612
411244 411877 412511 413145 413780 414415 415051 415687 416323 416960 417598

418236 418874 419513 420153 420793 421433 422074 422716 423358 424000 424643
425287 425931 426575 427220 427865 428511 429158 429804 430452 431100 431748
432397 433046 433696 434346 434997 435648 436300 436952 437605 438258 438912
439566 440221 440876 441532 442188 442845 443502 444160 444818 445476 446136
446795 447455 448116 448777 449439 450101 450763 451426 452090 452754 453418
454083 454749 455415 456081 456748 457416 458084 458752 459421 460091 460760
461431 462102 462773 463445 464117 464790 465464 466137 466812 467487 468162
468838 469514 470191 470868 471546 472224 472903 473582 474262 474942 475623
476304 476986 477668 478350 479034 479717 480401 481086 481771 482457 483143
483829 484517 485204 485892 486581 487270 487959 488649 489340 490031 490722
491414 492107 492800 493493 494187 494882 495577 496272 496968 497664 498361
499059 499757 500455 501154 501853 502553 503253 503954 504655 505357 506060
506762 507466 508169 508874 509578 510284 510989 511696 512402 513109 513817
514525 515234 515943 516653 517363 518074 518785 519496 520208 520921 521634
522348 523062 523776 524491 525207 525923 526640 527357 528074 528792 529511
530230 530949 531669 532389 533110 533832 534554 535276 535999 536722 537446
538171 538896 539621 540347 541073 541800 542527 543255 543983 544712 545441
546171 546902 547632 548364 549095 549827 550560 551293 552027 552761 553496
554231 554967 555703 556440 557177 557914 558653 559391 560130 560870 561610
562351 563092 563833 564575 565318 566061 566804 567548 568293 569038 569783
570529 571276 572023 572770 573518 574267 575015 575765 576515 577265 578016
578767 579519 580272 581025 581778 582532 583286 584041 584796 585552 586308
587065 587822 588580 589338 590097 590856 591616 592376 593137 593898 594660
595422 596185 596948 597711 598476 599240 600005 600771 601537 602304 603071
603838 604606 605375 606144 606914 607684 608454 609225 609997 610769 611541
612314 613088 613862 614636 615411 616186 616962 617739 618516 619293 620071
620849 621628 622408 623188 623968 624749 625530 626312 627094 627877 628660
629444 630228 631013 631798 632584 633370 634157 634944 635732 636520 637309
638098 638888 639678 640469 641260 642051 642844 643636 644429 645223 646017
646812 647607 648402 649198 649995 650792 651590 652388 653186 653985 654785
655585 656385 657186 657988 658790 659592 660395 661198 662002 662807 663612
664417 665223 666029 666836 667644 668451 669260 670069 670878 671688 672498
673309 674120 674932 675744 676557 677370 678184 678998 679813 680628 681444
682260 683077 683894 684712 685530 686349 687168 687987 688808 689628 690449
691271 692093 692916 693739 694562 695386 696211 697036 697862 698688 699514
700341 701169 701997 702825 703654 704484 705314 706144 706975 707806 708638
709471 710304 711137 711971 712805 713640 714476 715312 716148 716985 717822
718660 719498 720337 721176 722016 722857 723697 724539 725380 726223 727065
727909 728752 729597 730441 731286 732132 732978 733825 734672 735520 736368
737217 738066 738915 739766 740616 741467 742319 743171 744023 744877 745730
746584 747439 748294 749149 750005 750862 751719 752576 753434 754293 755152
756011 756871 757732 758593 759454 760316 761178 762041 762905 763768 764633
765498 766363 767229 768095 768962 769829 770697 771566 772434 773304 774173
775044 775914 776786 777657 778530 779402 780276 781149 782024 782898 783773
784649 785525 786402 787279 788157 789035 789914 790793 791672 792552 793433
794314 795196 796078 796960 797843 798727 799611 800495 801380 802266 803152
804038 804925 805813 806701 807589 808478 809368 810258 811148 812039 812931
813822 814715 815608 816501 817395 818289 819184 820080 820975 821872 822769

823666 824564 825462 826361 827260 828160 829060 829961 830862 831764 832666
833569 834472 835376 836280 837185 838090 838995 839902 840808 841715 842623
843531 844440 845349 846259 847169 848079 848990 849902 850814 851727 852640
853553 854467 855382 856297 857212 858128 859045 859962 860879 861797 862716
863635 864554 865474 866394 867315 868237 869159 870081 871004 871927 872851
873776 874700 875626 876552 877478 878405 879332 880260 881188 882117 883046
883976 884906 885837 886768 887700 888632 889565 890498 891432 892366 893301
894236 895172 896108 897045 897982 898919 899857 900796 901735 902675 903615
904556 905497 906438 907380 908323 909266 910209 911153 912098 913043 913989
914935 915881 916828 917776 918723 919672 920621 921570 922520 923471 924422
925373 926325 927277 928230 929184 930138 931092 932047 933002 933958 934914
935871 936828 937786 938745 939703 940663 941622 942583 943544 944505 945467
946429 947392 948355 949318 950283 951247 952213 953178 954144 955111 956078
957046 958014 958983 959952 960921 961892 962862 963833 964805 965777 966750
967723 968696 969670 970645 971620 972595 973571 974548 975525 976502 977480
978459 979438 980417 981397 982378 983359 984340 985322 986304 987287 988271
989255 990239 991224 992209 993195 994181 995168 996156 997143 998132 999121
1000110 1001100 1002090 1003081 1004072 1005064 1006056 1007049 1008042 1009036
1010030 1011025 1012020 1013016 1014012 1015009 1016006 1017003 1018002 1019000
1019999 1020999 1021999 1023000 1024001 1025003 1026005 1027007 1028010 1029014
1030018 1031023 1032028 1033033 1034039 1035046 1036053 1037060 1038068 1039077
1040086 1041095 1042105 1043116 1044127 1045138 1046150 1047162 1048175 1049189
1050203 1051217 1052232 1053247 1054263 1055280 1056297 1057314 1058332 1059350
1060369 1061388 1062408 1063428 1064449 1065471 1066492 1067515 1068537 1069561
1070584 1071609 1072633 1073659 1074684 1075711 1076737 1077765 1078792 1079820
1080849 1081878 1082908 1083938 1084969 1086000 1087032 1088064 1089096 1090129
1091163 1092197 1093232 1094267 1095302 1096338 1097375 1098412 1099450 1100488
1101526 1102565 1103605 1104645 1105685 1106726 1107768 1108810 1109852 1110895
1111939 1112982 1114027 1115072 1116117 1117163 1118210 1119256 1120304 1121352
1122400 1123449 1124498 1125548 1126599 1127649 1128701 1129753 1130805 1131858
1132911 1133965 1135019 1136074 1137129 1138185 1139241 1140298 1141355 1142413
1143471 1144530 1145589 1146649 1147709 1148769 1149831 1150892 1151955 1153017
1154080 1155144 1156208 1157273 1158338 1159403 1160470 1161536 1162603 1163671
1164739 1165807 1166876 1167946 1169016 1170087 1171158 1172229 1173301 1174374
1175447 1176520 1177594 1178669 1179744 1180819 1181895 1182971 1184048 1185126
1186204 1187282 1188361 1189440 1190520 1191601 1192682 1193763 1194845 1195927
1197010 1198093 1199177 1200261 1201346 1202432 1203517 1204604 1205690 1206778
1207865 1208954 1210042 1211132 1212221 1213312 1214402 1215494 1216585 1217678
1218770 1219863 1220957 1222051 1223146 1224241 1225337 1226433 1227529 1228627
1229724 1230822 1231921 1233020 1234120 1235220 1236320 1237421 1238523 1239625
1240727 1241831 1242934 1244038 1245143 1246248 1247353 1248459 1249566 1250673
1251780 1252888 1253996 1255105 1256215 1257325 1258435 1259546 1260658 1261769
1262882 1263995 1265108 1266222 1267336 1268451 1269567 1270682 1271799 1272916
1274033 1275151 1276269 1277388 1278507 1279627 1280747 1281868 1282989 1284111
1285233 1286356 1287479 1288603 1289727 1290852 1291977 1293103 1294229 1295355
1296483 1297610 1298738 1299867 1300996 1302126 1303256 1304387 1305518 1306649
1307781 1308914 1310047 1311181 1312315 1313449 1314584 1315720 1316856 1317993
1319130 1320267 1321405 1322544 1323683 1324822 1325962 1327103 1328243 1329385

1330527 1331669 1332812 1333956 1335100 1336244 1337389 1338534 1339680 1340827
1341973 1343121 1344269 1345417 1346566 1347715 1348865 1350015 1351166 1352318
1353469 1354622 1355774 1356928 1358082 1359236 1360391 1361546 1362702 1363858
1365015 1366172 1367329 1368488 1369646 1370806 1371965 1373125 1374286 1375447
1376609 1377771 1378934 1380097 1381260 1382425 1383589 1384754 1385920 1387086
1388253 1389420 1390587 1391755 1392924 1394093 1395262 1396432 1397603 1398774
1399946 1401118 1402290 1403463 1404637 1405811 1406985 1408160 1409335 1410511
1411688 1412865 1414042 1415220 1416398 1417577 1418757 1419937 1421117 1422298
1423479 1424661 1425844 1427026 1428210 1429394 1430578 1431763 1432948 1434134
1435320 1436507 1437694 1438882 1440070 1441259 1442448 1443638 1444828 1446019
1447210 1448402 1449594 1450787 1451980 1453174 1454368 1455563 1456758 1457953
1459149 1460346 1461543 1462741 1463939 1465138 1466337 1467536 1468736 1469937
1471138 1472340 1473542 1474744 1475947 1477151 1478355 1479559 1480765 1481970
1483176 1484383 1485590 1486797 1488005 1489213 1490422 1491632 1492842 1494052
1495263 1496475 1497687 1498899 1500112 1501325 1502539 1503754 1504968 1506184
1507400 1508616 1509833 1511050 1512268 1513486 1514705 1515925 1517144 1518365
1519585 1520807 1522029 1523251 1524474 1525697 1526921 1528145 1529370 1530595
1531821 1533047 1534273 1535501 1536728 1537957 1539185 1540414 1541644 1542874
1544105 1545336 1546568 1547800 1549032 1550266 1551499 1552733 1553968 1555203
1556439 1557675 1558911 1560148 1561386 1562624 1563862 1565101 1566341 1567581
1568822 1570063 1571304 1572546 1573788 1575031 1576275 1577519 1578763 1580008
1581254 1582500 1583746 1584993 1586240 1587488 1588737 1589986 1591235 1592485
1593735 1594986 1596237 1597489 1598742 1599995 1601248 1602502 1603756 1605011
1606266 1607522 1608778 1610035 1611292 1612550 1613808 1615067 1616326 1617586
1618846 1620107 1621368 1622630 1623892 1625155 1626418 1627682 1628946 1630211
1631476 1632741 1634007 1635274 1636541 1637809 1639077 1640346 1641615 1642884
1644154 1645425 1646696 1647968 1649240 1650512 1651785 1653059 1654333 1655607
1656882 1658158 1659434 1660710 1661987 1663265 1664543 1665821 1667100 1668380
1669660 1670940 1672221 1673503 1674785 1676067 1677350 1678633 1679917 1681202
1682486 1683772 1685058 1686344 1687631 1688918 1690206 1691494 1692783 1694073
1695362 1696653 1697943 1699235 1700527 1701819 1703112 1704405 1705699 1706993
1708288 1709583 1710879 1712175 1713472 1714769 1716066 1717365 1718663 1719963
1721262 1722562 1723863 1725164 1726466 1727768 1729071 1730374 1731677 1732981
1734286 1735591 1736897 1738203 1739509 1740817 1742124 1743432 1744741 1746050
1747359 1748669 1749980 1751291 1752602 1753914 1755227 1756540 1757853 1759167
1760482 1761797 1763112 1764428 1765745 1767062 1768379 1769697 1771015 1772334
1773654 1774974 1776294 1777615 1778936 1780258 1781580 1782903 1784227 1785550
1786875 1788200 1789525 1790851 1792177 1793504 1794831 1796159 1797487 1798816
1800145 1801475 1802805 1804136 1805467 1806799 1808131 1809464 1810797 1812131
1813465 1814800 1816135 1817471 1818807 1820143 1821481 1822818 1824156 1825495
1826834 1828174 1829514 1830854 1832196 1833537 1834879 1836222 1837565 1838908
1840253 1841597 1842942 1844288 1845634 1846980 1848327 1849675 1851023 1852371
1853720 1855070 1856420 1857770 1859121 1860473 1861825 1863177 1864530 1865883
1867237 1868592 1869947 1871302 1872658 1874014 1875371 1876729 1878087 1879445
1880804 1882163 1883523 1884883 1886244 1887605 1888967 1890330 1891692 1893056
1894419 1895784 1897149 1898514 1899880 1901246 1902613 1903980 1905348 1906716
1908084 1909454 1910823 1912194 1913564 1914936 1916307 1917679 1919052 1920425
1921799 1923173 1924548 1925923 1927298 1928675 1930051 1931428 1932806 1934184

1935563 1936942 1938321 1939701 1941082 1942463 1943844 1945227 1946609 1947992
1949376 1950760 1952144 1953529 1954915 1956301 1957687 1959074 1960462 1961850
1963238 1964627 1966016 1967406 1968797 1970188 1971579 1972971 1974363 1975756
1977150 1978544 1979938 1981333 1982728 1984124 1985520 1986917 1988315 1989712
1991111 1992510 1993909 1995309 1996709 1998110 1999511 2000913 2002315 2003718
2005121 2006525 2007929 2009334 2010739 2012145 2013551 2014958 2016365 2017773
2019181 2020589 2021999 2023408 2024818 2026229 2027640 2029052 2030464 2031877
2033290 2034703 2036118 2037532 2038947 2040363 2041779 2043196 2044613 2046030
2047448 2048867 2050286 2051705 2053125 2054546 2055967 2057388 2058810 2060233
2061656 2063079 2064503 2065928 2067353 2068778 2070204 2071631 2073058 2074485
2075913 2077341 2078770 2080200 2081630 2083060 2084491 2085922 2087354 2088787
2090219 2091653 2093087 2094521 2095956 2097391 2098827 2100263 2101700 2103137
2104575 2106013 2107452 2108892 2110331 2111772 2113212 2114654 2116095 2117538
2118980 2120423 2121867 2123311 2124756 2126201 2127647 2129093 2130540 2131987
2133435 2134883 2136332 2137781 2139230 2140680 2142131 2143582 2145034 2146486
2147938 2149392 2150845 2152299 2153754 2155209 2156664 2158121 2159577 2161034
2162492 2163950 2165408 2166867 2168327 2169787 2171247 2172708 2174170 2175632
2177094 2178557 2180021 2181484 2182949 2184414 2185879 2187345 2188812 2190279
2191746 2193214 2194682 2196151 2197621 2199090 2200561 2202032 2203503 2204975
2206447 2207920 2209393 2210867 2212341 2213816 2215292 2216767 2218244 2219720
2221198 2222675 2224154 2225632 2227112 2228591 2230072 2231552 2233034 2234515
2235998 2237480 2238963 2240447 2241931 2243416 2244901 2246387 2247873 2249360
2250847 2252334 2253823 2255311 2256800 2258290 2259780 2261271 2262762 2264253
2265745 2267238 2268731 2270225 2271719 2273213 2274708 2276204 2277700 2279196
2280693 2282191 2283689 2285187 2286686 2288186 2289686 2291186 2292687 2294189
2295691 2297193 2298696 2300200 2301703 2303208 2304713 2306218 2307724 2309230
2310737 2312245 2313753 2315261 2316770 2318279 2319789 2321299 2322810 2324322
2325833 2327346 2328859 2330372 2331886 2333400 2334915 2336430 2337946 2339462
2340979 2342496 2344014 2345532 2347051 2348570 2350090 2351610 2353131 2354652
2356174 2357696 2359218 2360742 2362265 2363789 2365314 2366839 2368365 2369891
2371418 2372945 2374472 2376001 2377529 2379058 2380588 2382118 2383649 2385180
2386711 2388243 2389776 2391309 2392842 2394376 2395911 2397446 2398982 2400518
2402054 2403591 2405129 2406667 2408205 2409744 2411283 2412823 2414364 2415905
2417446 2418988 2420531 2422074 2423617 2425161 2426705 2428250 2429796 2431341
2432888 2434435 2435982 2437530 2439078 2440627 2442176 2443726 2445277 2446827
2448379 2449931 2451483 2453036 2454589 2456143 2457697 2459252 2460807 2462363
2463919 2465476 2467033 2468591 2470149 2471708 2473267 2474827 2476387 2477948
2479509 2481070 2482633 2484195 2485758 2487322 2488886 2490451 2492016 2493582
2495148 2496714 2498282 2499849 2501417 2502986 2504555 2506125 2507695 2509265
2510836 2512408 2513980 2515552 2517125 2518699 2520273 2521848 2523423 2524998
2526574 2528151 2529728 2531305 2532883 2534461 2536040 2537620 2539200 2540780
2542361 2543943 2545525 2547107 2548690 2550273 2551857 2553442 2555027 2556612
2558198 2559784 2561371 2562958 2564546 2566135 2567723 2569313 2570903 2572493
2574084 2575675 2577267 2578859 2580452 2582045 2583639 2585233 2586828 2588423
2590019 2591615 2593212 2594809 2596407 2598005 2599604 2601203 2602803 2604403
2606004 2607605 2609207 2610809 2612411 2614015 2615618 2617222 2618827 2620432
2622038 2623644 2625251 2626858 2628465 2630073 2631682 2633291 2634901 2636511
2638121 2639732 2641344 2642956 2644568 2646181 2647795 2649409 2651024 2652639

2654254 2655870 2657487 2659104 2660721 2662339 2663957 2665576 2667196 2668816
2670436 2672057 2673679 2675301 2676923 2678546 2680169 2681793 2683418 2685042
2686668 2688294 2689920 2691547 2693174 2694802 2696430 2698059 2699689 2701318
2702949 2704580 2706211 2707843 2709475 2711108 2712741 2714375 2716009 2717644
2719279 2720915 2722551 2724188 2725825 2727463 2729101 2730740 2732379 2734019
2735659 2737300 2738941 2740582 2742225 2743867 2745510 2747154 2748798 2750443
2752088 2753734 2755380 2757026 2758673 2760321 2761969 2763618 2765267 2766916
2768567 2770217 2771868 2773520 2775172 2776824 2778477 2780131 2781785 2783439
2785094 2786750 2788406 2790062 2791719 2793377 2795035 2796693 2798352 2800012
2801672 2803332 2804993 2806655 2808316 2809979 2811642 2813305 2814969 2816634
2818298 2819964 2821630 2823296 2824963 2826630 2828298 2829966 2831635 2833305
2834974 2836645 2838316 2839987 2841659 2843331 2845004 2846677 2848351 2850025
2851700 2853375 2855051 2856727 2858404 2860081 2861759 2863437 2865116 2866795
2868475 2870155 2871836 2873517 2875199 2876881 2878563 2880247 2881930 2883614
2885299 2886984 2888670 2890356 2892043 2893730 2895417 2897105 2898794 2900483
2902173 2903863 2905553 2907244 2908936 2910628 2912321 2914014 2915707 2917401
2919096 2920791 2922486 2924182 2925879 2927576 2929273 2930971 2932670 2934369
2936068 2937768 2939469 2941169 2942871 2944573 2946275 2947978 2949682 2951386
2953090 2954795 2956500 2958206 2959912 2961619 2963327 2965035 2966743 2968452
2970161 2971871 2973581 2975292 2977003 2978715 2980428 2982140 2983854 2985567
2987282 2988997 2990712 2992428 2994144 2995861 2997578 2999296 3001014 3002733
3004452 3006172 3007892 3009612 3011334 3013055 3014778 3016500 3018223 3019947
3021671 3023396 3025121 3026847 3028573 3030299 3032027 3033754 3035482 3037211
3038940 3040670 3042400 3044130 3045861 3047593 3049325 3051058 3052791 3054524
3056258 3057993 3059728 3061463 3063199 3064936 3066673 3068410 3070148 3071887
3073626 3075365 3077105 3078846 3080587 3082328 3084070 3085813 3087556 3089299
3091043 3092787 3094532 3096278 3098024 3099770 3101517 3103264 3105012 3106760
3108509 3110259 3112009 3113759 3115510 3117261 3119013 3120765 3122518 3124271
3126025 3127779 3129534 3131290 3133045 3134802 3136558 3138316 3140073 3141832
3143590 3145350 3147109 3148870 3150630 3152392 3154153 3155915 3157678 3159441
3161205 3162969 3164734 3166499 3168265 3170031 3171798 3173565 3175332 3177101
3178869 3180638 3182408 3184178 3185949 3187720 3189491 3191263 3193036 3194809
3196583 3198357 3200131 3201906 3203682 3205458 3207234 3209011 3210789 3212567
3214345 3216124 3217904 3219684 3221464 3223245 3225027 3226809 3228591 3230374
3232158 3233942 3235726 3237511 3239297 3241082 3242869 3244656 3246443 3248231
3250019 3251808 3253598 3255388 3257178 3258969 3260760 3262552 3264344 3266137
3267931 3269724 3271519 3273314 3275109 3276905 3278701 3280498 3282295 3284093
3285891 3287690 3289489 3291289 3293089 3294890 3296691 3298493 3300295 3302098
3303901 3305705 3307509 3309314 3311119 3312925 3314731 3316537 3318345 3320152
3321960 3323769 3325578 3327388 3329198 3331009 3332820 3334631 3336443 3338256
3340069 3341883 3343697 3345511 3347326 3349142 3350958 3352775 3354592 3356409
3358227 3360046 3361865 3363684 3365504 3367325 3369146 3370967 3372789 3374612
3376435 3378258 3380082 3381907 3383732 3385557 3387383 3389210 3391037 3392864
3394692 3396520 3398349 3400179 3402009 3403839 3405670 3407501 3409333 3411165
3412998 3414832 3416666 3418500 3420335 3422170 3424006 3425842 3427679 3429516
3431354 3433192 3435031 3436871 3438710 3440551 3442391 3444233 3446074 3447917
3449759 3451603 3453446 3455291 3457135 3458981 3460826 3462673 3464519 3466366
3468214 3470062 3471911 3473760 3475610 3477460 3479311 3481162 3483013 3484866

3486718 3488571 3490425 3492279 3494134 3495989 3497844 3499701 3501557 3503414
3505272 3507130 3508988 3510847 3512707 3514567 3516428 3518289 3520150 3522012
3523875 3525738 3527601 3529465 3531330 3533195 3535060 3536926 3538793 3540660
3542527 3544395 3546264 3548132 3550002 3551872 3553742 3555613 3557485 3559357
3561229 3563102 3564975 3566849 3568723 3570598 3572474 3574350 3576226 3578103
3579980 3581858 3583736 3585615 3587494 3589374 3591255 3593135 3595017 3596899
3598781 3600664 3602547 3604431 3606315 3608200 3610085 3611971 3613857 3615744
3617631 3619519 3621407 3623296 3625185 3627074 3628965 3630855 3632747 3634638
3636530 3638423 3640316 3642210 3644104 3645999 3647894 3649789 3651686 3653582
3655479 3657377 3659275 3661174 3663073 3664972 3666872 3668773 3670674 3672576
3674478 3676380 3678283 3680187 3682091 3683995 3685900 3687806 3689712 3691618
3693525 3695433 3697341 3699249 3701158 3703068 3704978 3706888 3708799 3710711
3712623 3714535 3716448 3718361 3720275 3722190 3724105 3726020 3727936 3729852
3731769 3733687 3735605 3737523 3739442 3741361 3743281 3745201 3747122 3749044
3750965 3752888 3754811 3756734 3758658 3760582 3762507 3764432 3766358 3768284
3770211 3772138 3774066 3775994 3777923 3779852 3781782 3783712 3785643 3787574
3789506 3791438 3793371 3795304 3797238 3799172 3801107 3803042 3804977 3806914
3808850 3810787 3812725 3814663 3816602 3818541 3820481 3822421 3824361 3826302
3828244 3830186 3832129 3834072 3836015 3837960 3839904 3841849 3843795 3845741
3847687 3849634 3851582 3853530 3855478 3857427 3859377 3861327 3863278 3865229
3867180 3869132 3871084 3873037 3874991 3876945 3878899 3880854 3882810 3884766
3886722 3888679 3890636 3892594 3894553 3896512 3898471 3900431 3902391 3904352
3906314 3908276 3910238 3912201 3914164 3916128 3918092 3920057 3922023 3923988
3925955 3927922 3929889 3931857 3933825 3935794 3937763 3939733 3941703 3943674
3945645 3947617 3949589 3951562 3953535 3955509 3957483 3959458 3961433 3963409
3965385 3967362 3969339 3971317 3973295 3975273 3977253 3979232 3981212 3983193
3985174 3987156 3989138 3991121 3993104 3995088 3997072 3999056 4001041 4003027
4005013 4007000 4008987 4010974 4012962 4014951 4016940 4018930 4020920 4022910
4024901 4026893 4028885 4030877 4032870 4034864 4036858 4038852 4040847 4042843
4044839 4046835 4048832 4050830 4052828 4054826 4056825 4058825 4060825 4062825
4064826 4066827 4068829 4070832 4072835 4074838 4076842 4078846 4080851 4082857
4084863 4086869 4088876 4090883 4092891 4094899 4096908 4098918 4100927 4102938
4104948 4106960 4108972 4110984 4112997 4115010 4117024 4119038 4121053 4123068
4125084 4127100 4129117 4131134 4133152 4135170 4137189 4139208 4141228 4143248
4145269 4147290 4149312 4151334 4153357 4155380 4157403 4159428 4161452 4163477
4165503 4167529 4169556 4171583 4173611 4175639 4177667 4179697 4181726 4183756
4185787 4187818 4189850 4191882 4193914 4195947 4197981 4200015 4202049 4204084
4206120 4208156 4210193 4212230 4214267 4216305 4218344 4220383 4222422 4224462
4226503 4228544 4230585 4232627 4234670 4236713 4238756 4240800 4242844 4244889
4246935 4248981 4251027 4253074 4255121 4257169 4259218 4261267 4263316 4265366
4267416 4269467 4271519 4273570 4275623 4277676 4279729 4281783 4283837 4285892
4287947 4290003 4292059 4294116 4296174 4298231 4300290 4302348 4304408 4306467
4308528 4310589 4312650 4314712 4316774 4318837 4320900 4322963 4325028 4327092
4329158 4331223 4333290 4335356 4337423 4339491 4341559 4343628 4345697 4347767
4349837 4351908 4353979 4356050 4358123 4360195 4362268 4364342 4366416 4368491
4370566 4372641 4374718 4376794 4378871 4380949 4383027 4385105 4387184 4389264
4391344 4393425 4395506 4397587 4399669 4401752 4403835 4405918 4408002 4410087
4412172 4414257 4416343 4418430 4420517 4422604 4424692 4426781 4428870 4430959

4433049 4435139 4437230 4439322 4441414 4443506 4445599 4447692 4449786 4451881
4453976 4456071 4458167 4460263 4462360 4464457 4466555 4468654 4470752 4472852
4474952 4477052 4479153 4481254 4483356 4485458 4487561 4489664 4491768 4493872
4495977 4498082 4500188 4502294 4504401 4506508 4508616 4510724 4512833 4514942
4517052 4519162 4521273 4523384 4525496 4527608 4529721 4531834 4533948 4536062
4538177 4540292 4542407 4544524 4546640 4548757 4550875 4552993 4555112 4557231
4559351 4561471 4563591 4565712 4567834 4569956 4572079 4574202 4576325 4578449
4580574 4582699 4584824 4586951 4589077 4591204 4593332 4595460 4597588 4599717
4601847 4603977 4606107 4608238 4610370 4612502 4614634 4616767 4618901 4621035
4623169 4625304 4627439 4629575 4631712 4633849 4635986 4638124 4640262 4642401
4644541 4646681 4648821 4650962 4653103 4655245 4657388 4659530 4661674 4663818
4665962 4668107 4670252 4672398 4674544 4676691 4678838 4680986 4683135 4685283
4687433 4689582 4691733 4693883 4696035 4698186 4700339 4702492 4704645 4706799
4708953 4711107 4713263 4715418 4717575 4719731 4721889 4724046 4726204 4728363
4730522 4732682 4734842 4737003 4739164 4741326 4743488 4745650 4747814 4749977
4752141 4754306 4756471 4758637 4760803 4762969 4765137 4767304 4769472 4771641
4773810 4775979 4778150 4780320 4782491 4784663 4786835 4789007 4791180 4793354
4795528 4797702 4799877 4802053 4804229 4806405 4808582 4810760 4812938 4815116
4817295 4819475 4821655 4823835 4826016 4828198 4830380 4832562 4834745 4836928
4839112 4841297 4843482 4845667 4847853 4850039 4852226 4854414 4856601 4858790
4860979 4863168 4865358 4867548 4869739 4871930 4874122 4876315 4878507 4880701
4882895 4885089 4887284 4889479 4891675 4893871 4896068 4898265 4900463 4902661
4904860 4907059 4909259 4911459 4913660 4915861 4918063 4920265 4922468 4924671
4926875 4929079 4931284 4933489 4935694 4937901 4940107 4942314 4944522 4946730
4948939 4951148 4953358 4955568 4957778 4959990 4962201 4964413 4966626 4968839
4971053 4973267 4975481 4977696 4979912 4982128 4984345 4986562 4988779 4990997
4993216 4995435 4997654 4999874 5002095 5004316 5006538 5008760 5010982 5013205
5015429 5017653 5019877 5022102 5024327 5026553 5028780 5031007 5033234 5035462
5037691 5039920 5042149 5044379 5046609 5048840 5051072 5053304 5055536 5057769
5060002 5062236 5064471 5066705 5068941 5071177 5073413 5075650 5077887 5080125
5082363 5084602 5086842 5089081 5091322 5093562 5095804 5098046 5100288 5102531
5104774 5107018 5109262 5111507 5113752 5115998 5118244 5120491 5122738 5124986
5127234 5129483 5131732 5133982 5136232 5138483 5140734 5142985 5145238 5147490
5149743 5151997 5154251 5156506 5158761 5161017 5163273 5165530 5167787 5170044
5172302 5174561 5176820 5179080 5181340 5183601 5185862 5188123 5190385 5192648
5194911 5197175 5199439 5201703 5203968 5206234 5208500 5210766 5213033 5215301
5217569 5219837 5222106 5224376 5226646 5228916 5231187 5233459 5235731 5238003
5240276 5242550 5244824 5247098 5249373 5251649 5253924 5256201 5258478 5260755
5263033 5265312 5267591 5269870 5272150 5274430 5276711 5278992 5281274 5283557
5285840 5288123 5290407 5292691 5294976 5297261 5299547 5301833 5304120 5306408
5308695 5310984 5313273 5315562 5317852 5320142 5322433 5324724 5327016 5329308
5331601 5333894 5336188 5338482 5340777 5343072 5345368 5347664 5349961 5352258
5354556 5356854 5359152 5361452 5363751 5366052 5368352 5370653 5372955 5375257
5377560 5379863 5382167 5384471 5386775 5389081 5391386 5393692 5395999 5398306
5400614 5402922 5405230 5407539 5409849 5412159 5414470 5416781 5419092 5421404
5423717 5426030 5428343 5430657 5432972 5435287 5437603 5439919 5442235 5444552
5446870 5449188 5451506 5453825 5456145 5458465 5460785 5463106 5465427 5467749
5470072 5472395 5474718 5477042 5479367 5481691 5484017 5486343 5488669 5490996

5493324 5495651 5497980 5500309 5502638 5504968 5507298 5509629 5511960 5514292
5516625 5518958 5521291 5523625 5525959 5528294 5530629 5532965 5535301 5537638
5539975 5542313 5544651 5546990 5549329 5551669 5554010 5556350 5558692 5561033
5563376 5565718 5568061 5570405 5572749 5575094 5577439 5579785 5582131 5584478
5586825 5589173 5591521 5593870 5596219 5598569 5600919 5603269 5605620 5607972
5610324 5612677 5615030 5617384 5619738 5622092 5624447 5626803 5629159 5631516
5633873 5636230 5638589 5640947 5643306 5645666 5648026 5650386 5652747 5655109
5657471 5659833 5662197 5664560 5666924 5669289 5671654 5674019 5676385 5678752
5681119 5683486 5685854 5688222 5690591 5692961 5695331 5697701 5700072 5702444
5704816 5707188 5709561 5711934 5714308 5716683 5719058 5721433 5723809 5726185
5728562 5730940 5733317 5735696 5738075 5740454 5742834 5745214 5747595 5749976
5752358 5754741 5757123 5759507 5761890 5764275 5766660 5769045 5771431 5773817
5776204 5778591 5780979 5783367 5785756 5788145 5790535 5792925 5795316 5797707
5800099 5802491 5804884 5807277 5809671 5812065 5814460 5816855 5819250 5821647
5824043 5826440 5828838 5831236 5833635 5836034 5838434 5840834 5843235 5845636
5848037 5850439 5852842 5855245 5857649 5860053 5862457 5864863 5867268 5869674
5872081 5874488 5876895 5879303 5881712 5884121 5886531 5888941 5891351 5893762
5896174 5898586 5900998 5903411 5905825 5908239 5910653 5913068 5915484 5917900
5920316 5922733 5925151 5927569 5929987 5932406 5934826 5937245 5939666 5942087
5944508 5946930 5949353 5951775 5954199 5956623 5959047 5961472 5963897 5966323
5968750 5971177 5973604 5976032 5978460 5980889 5983318 5985748 5988179 5990609
5993041 5995472 5997905 6000338 6002771 6005205 6007639 6010074 6012509 6014945
6017381 6019818 6022255 6024693 6027131 6029570 6032009 6034449 6036889 6039330
6041771 6044213 6046655 6049098 6051541 6053985 6056429 6058874 6061319 6063764
6066211 6068657 6071104 6073552 6076000 6078449 6080898 6083348 6085798 6088248
6090699 6093151 6095603 6098056 6100509 6102963 6105417 6107871 6110326 6112782
6115238 6117694 6120152 6122609 6125067 6127526 6129985 6132444 6134904 6137365
6139826 6142287 6144749 6147212 6149675 6152138 6154602 6157067 6159532 6161997
6164463 6166930 6169397 6171864 6174332 6176801 6179270 6181739 6184209 6186679
6189150 6191622 6194094 6196566 6199039 6201512 6203986 6206461 6208935 6211411
6213887 6216363 6218840 6221317 6223795 6226273 6228752 6231232 6233711 6236192
6238673 6241154 6243636 6246118 6248601 6251084 6253568 6256052 6258537 6261022
6263508 6265994 6268481 6270968 6273456 6275944 6278433 6280922 6283412 6285902
6288393 6290884 6293376 6295868 6298361 6300854 6303348 6305842 6308337 6310832
6313327 6315824 6318320 6320817 6323315 6325813 6328312 6330811 6333311 6335811
6338311 6340812 6343314 6345816 6348319 6350822 6353325 6355830 6358334 6360839
6363345 6365851 6368357 6370864 6373372 6375880 6378389 6380898 6383407 6385917
6388428 6390939 6393450 6395962 6398475 6400988 6403501 6406015 6408530 6411045
6413560 6416076 6418593 6421110 6423627 6426145 6428663 6431182 6433702 6436222
6438742 6441263 6443784 6446306 6448829 6451352 6453875 6456399 6458923 6461448
6463974 6466499 6469026 6471553 6474080 6476608 6479136 6481665 6484194 6486724
6489254 6491785 6494317 6496848 6499381 6501913 6504447 6506981 6509515 6512050
6514585 6517121 6519657 6522194 6524731 6527269 6529807 6532346 6534885 6537425
6539965 6542506 6545047 6547589 6550131 6552673 6555217 6557760 6560304 6562849
6565394 6567940 6570486 6573033 6575580 6578128 6580676 6583224 6585774 6588323
6590873 6593424 6595975 6598527 6601079 6603631 6606184 6608738 6611292 6613847
6616402 6618957 6621513 6624070 6626627 6629185 6631743 6634301 6636860 6639420
6641980 6644540 6647101 6649663 6652225 6654787 6657350 6659914 6662478 6665042

6667607 6670173 6672739 6675305 6677872 6680440 6683007 6685576 6688145 6690714
6693284 6695855 6698426 6700997 6703569 6706141 6708714 6711288 6713861 6716436
6719011 6721586 6724162 6726738 6729315 6731893 6734470 6737049 6739628 6742207
6744787 6747367 6749948 6752529 6755111 6757693 6760276 6762859 6765443 6768027
6770612 6773198 6775783 6778370 6780956 6783544 6786131 6788720 6791308 6793898
6796487 6799078 6801668 6804259 6806851 6809443 6812036 6814629 6817223 6819817
6822412 6825007 6827603 6830199 6832796 6835393 6837990 6840588 6843187 6845786
6848386 6850986 6853587 6856188 6858789 6861391 6863994 6866597 6869201 6871805
6874409 6877015 6879620 6882226 6884833 6887440 6890047 6892655 6895264 6897873
6900483 6903093 6905703 6908314 6910926 6913538 6916150 6918763 6921377 6923991
6926605 6929220 6931836 6934452 6937068 6939685 6942302 6944920 6947539 6950158
6952777 6955397 6958018 6960639 6963260 6965882 6968504 6971127 6973751 6976374
6978999 6981624 6984249 6986875 6989501 6992128 6994756 6997383 7000012 7002641
7005270 7007900 7010530 7013161 7015792 7018424 7021056 7023689 7026323 7028956
7031591 7034225 7036861 7039496 7042133 7044769 7047407 7050045 7052683 7055322
7057961 7060601 7063241 7065881 7068523 7071164 7073807 7076449 7079093 7081736
7084380 7087025 7089670 7092316 7094962 7097609 7100256 7102904 7105552 7108200
7110850 7113499 7116149 7118800 7121451 7124103 7126755 7129408 7132061 7134714
7137368 7140023 7142678 7145334 7147990 7150646 7153303 7155961 7158619 7161277
7163937 7166596 7169256 7171917 7174578 7177239 7179901 7182564 7185227 7187890
7190554 7193219 7195884 7198549 7201215 7203882 7206549 7209216 7211884 7214553
7217222 7219891 7222561 7225231 7227902 7230574 7233246 7235918 7238591 7241264
7243938 7246613 7249288 7251963 7254639 7257315 7259992 7262669 7265347 7268026
7270705 7273384 7276064 7278744 7281425 7284106 7286788 7289470 7292153 7294837
7297520 7300205 7302889 7305575 7308261 7310947 7313634 7316321 7319009 7321697
7324386 7327075 7329765 7332455 7335146 7337837 7340529 7343221 7345914 7348607
7351301 7353995 7356689 7359385 7362080 7364777 7367473 7370170 7372868 7375566
7378265 7380964 7383664 7386364 7389064 7391766 7394467 7397169 7399872 7402575
7405279 7407983 7410687 7413393 7416098 7418804 7421511 7424218 7426926 7429634
7432342 7435051 7437761 7440471 7443181 7445892 7448604 7451316 7454029 7456742
7459455 7462169 7464884 7467599 7470314 7473030 7475747 7478464 7481181 7483899
7486618 7489336 7492056 7494776 7497496 7500217 7502939 7505661 7508383 7511106
7513829 7516553 7519278 7522003 7524728 7527454 7530180 7532907 7535635 7538363
7541091 7543820 7546549 7549279 7552009 7554740 7557472 7560203 7562936 7565669
7568402 7571136 7573870 7576605 7579340 7582076 7584812 7587549 7590286 7593024
7595763 7598501 7601241 7603980 7606721 7609461 7612203 7614944 7617687 7620429
7623173 7625916 7628661 7631405 7634150 7636896 7639642 7642389 7645136 7647884
7650632 7653381 7656130 7658880 7661630 7664380 7667132 7669883 7672635 7675388
7678141 7680895 7683649 7686404 7689159 7691914 7694670 7697427 7700184 7702942
7705700 7708458 7711218 7713977 7716737 7719498 7722259 7725020 7727782 7730545
7733308 7736072 7738836 7741600 7744365 7747131 7749897 7752663 7755430 7758198
7760966 7763734 7766503 7769273 7772043 7774813 7777584 7780356 7783127 7785900
7788673 7791446 7794220 7796995 7799770 7802545 7805321 7808097 7810874 7813652
7816430 7819208 7821987 7824766 7827546 7830327 7833107 7835889 7838671 7841453
7844236 7847019 7849803 7852587 7855372 7858158 7860943 7863730 7866517 7869304
7872092 7874880 7877669 7880458 7883248 7886038 7888829 7891620 7894412 7897204
7899997 7902790 7905584 7908378 7911173 7913968 7916764 7919560 7922357 7925154
7927952 7930750 7933549 7936348 7939148 7941948 7944748 7947550 7950351 7953154

7955956 7958759 7961563 7964367 7967172 7969977 7972783 7975589 7978395 7981202
7984010 7986818 7989627 7992436 7995246 7998056 8000866 8003677 8006489 8009301
8012114 8014927 8017740 8020554 8023369 8026184 8028999 8031815 8034632 8037449
8040266 8043084 8045903 8048722 8051542 8054362 8057182 8060003 8062824 8065646
8068469 8071292 8074115 8076939 8079764 8082589 8085414 8088240 8091067 8093894
8096721 8099549 8102377 8105206 8108036 8110866 8113696 8116527 8119358 8122190
8125023 8127855 8130689 8133523 8136357 8139192 8142027 8144863 8147699 8150536
8153374 8156211 8159050 8161889 8164728 8167568 8170408 8173249 8176090 8178932
8181774 8184617 8187460 8190304 8193148 8195993 8198838 8201684 8204530 8207377
8210224 8213072 8215920 8218769 8221618 8224468 8227318 8230169 8233020 8235872
8238724 8241577 8244430 8247284 8250138 8252992 8255848 8258703 8261559 8264416
8267273 8270131 8272989 8275848 8278707 8281566 8284427 8287287 8290148 8293010
8295872 8298735 8301598 8304461 8307325 8310190 8313055 8315921 8318787 8321653
8324520 8327388 8330256 8333124 8335993 8338863 8341733 8344604 8347475 8350346
8353218 8356091 8358964 8361837 8364711 8367586 8370461 8373336 8376212 8379088
8381965 8384843 8387721 8390599 8393478 8396358 8399238 8402118 8404999 8407880
8410762 8413645 8416528 8419411 8422295 8425179 8428064 8430950 8433835 8436722
8439609 8442496 8445384 8448272 8451161 8454050 8456940 8459831 8462721 8465613
8468505 8471397 8474290 8477183 8480077 8482971 8485866 8488761 8491657 8494553
8497450 8500347 8503245 8506143 8509042 8511941 8514841 8517741 8520642 8523543
8526445 8529347 8532250 8535153 8538057 8540961 8543866 8546771 8549677 8552583
8555490 8558397 8561305 8564213 8567121 8570031 8572940 8575850 8578761 8581672
8584584 8587496 8590409 8593322 8596235 8599149 8602064 8604979 8607895 8610811
8613727 8616645 8619562 8622480 8625399 8628318 8631237 8634157 8637078 8639999
8642921 8645843 8648765 8651688 8654612 8657536 8660460 8663385 8666311 8669237
8672163 8675090 8678018 8680946 8683874 8686803 8689733 8692663 8695593 8698524
8701456 8704388 8707320 8710253 8713186 8716120 8719055 8721990 8724925 8727861
8730797 8733734 8736672 8739610 8742548 8745487 8748426 8751366 8754306 8757247
8760189 8763131 8766073 8769016 8771959 8774903 8777847 8780792 8783737 8786683
8789630 8792576 8795524 8798471 8801420 8804369 8807318 8810268 8813218 8816169
8819120 8822072 8825024 8827977 8830930 8833884 8836838 8839793 8842748 8845704
8848660 8851617 8854574 8857532 8860490 8863448 8866408 8869367 8872328 8875288
8878249 8881211 8884173 8887136 8890099 8893063 8896027 8898992 8901957 8904922
8907888 8910855 8913822 8916790 8919758 8922727 8925696 8928665 8931635 8934606
8937577 8940549 8943521 8946493 8949466 8952440 8955414 8958388 8961364 8964339
8967315 8970292 8973269 8976246 8979224 8982203 8985182 8988161 8991141 8994122
8997103 9000084 9003066 9006049 9009032 9012015 9014999 9017984 9020969 9023954
9026940 9029926 9032913 9035901 9038889 9041877 9044866 9047856 9050845 9053836
9056827 9059818 9062810 9065802 9068795 9071789 9074783 9077777 9080772 9083767
9086763 9089759 9092756 9095754 9098751 9101750 9104749 9107748 9110748 9113748
9116749 9119750 9122752 9125754 9128757 9131760 9134764 9137768 9140773 9143778
9146784 9149790 9152797 9155805 9158812 9161821 9164829 9167838 9170848 9173858
9176869 9179880 9182892 9185904 9188917 9191930 9194944 9197958 9200973 9203988
9207004 9210020 9213036 9216054 9219071 9222089 9225108 9228127 9231147 9234167
9237188 9240209 9243230 9246253 9249275 9252298 9255322 9258346 9261371 9264396
9267421 9270447 9273474 9276501 9279528 9282557 9285585 9288614 9291644 9294674
9297704 9300735 9303767 9306799 9309831 9312864 9315898 9318932 9321966 9325001
9328037 9331073 9334109 9337146 9340184 9343222 9346260 9349299 9352339 9355379

9358419 9361460 9364502 9367543 9370586 9373629 9376672 9379716 9382761 9385806
9388851 9391897 9394943 9397990 9401037 9404085 9407134 9410183 9413232 9416282
9419332 9422383 9425434 9428486 9431539 9434591 9437645 9440699 9443753 9446808
9449863 9452919 9455975 9459032 9462089 9465147 9468205 9471264 9474323 9477383
9480443 9483504 9486565 9489627 9492689 9495752 9498815 9501879 9504943 9508008
9511073 9514139 9517205 9520272 9523339 9526407 9529475 9532544 9535613 9538683
9541753 9544824 9547895 9550966 9554039 9557111 9560184 9563258 9566332 9569407
9572482 9575558 9578634 9581710 9584787 9587865 9590943 9594022 9597101 9600180
9603260 9606341 9609422 9612504 9615586 9618668 9621751 9624835 9627919 9631004
9634089 9637174 9640260 9643347 9646434 9649521 9652609 9655698 9658787 9661876
9664966 9668057 9671148 9674239 9677331 9680424 9683517 9686610 9689704 9692799
9695894 9698989 9702085 9705181 9708278 9711376 9714474 9717572 9720671 9723770
9726870 9729971 9733072 9736173 9739275 9742377 9745480 9748584 9751687 9754792
9757897 9761002 9764108 9767214 9770321 9773428 9776536 9779645 9782753 9785863
9788973 9792083 9795194 9798305 9801417 9804529 9807642 9810755 9813869 9816983
9820098 9823213 9826329 9829445 9832562 9835679 9838797 9841915 9845034 9848153
9851273 9854393 9857514 9860635 9863757 9866879 9870002 9873125 9876249 9879373
9882498 9885623 9888748 9891875 9895001 9898128 9901256 9904384 9907513 9910642
9913772 9916902 9920032 9923164 9926295 9929427 9932560 9935693 9938827 9941961
9945095 9948230 9951366 9954502 9957639 9960776 9963913 9967051 9970190 9973329
9976469 9979609 9982749 9985890 9989032 9992174 9995316 9998459 10001603 10004747
10007891 10011036 10014182 10017328 10020474 10023621 10026769 10029917 10033065
10036214 10039364 10042514 10045664 10048815 10051967 10055118 10058271 10061424
10064577 10067731 10070886 10074041 10077196 10080352 10083508 10086665 10089823
10092980 10096139 10099298 10102457 10105617 10108777 10111938 10115099 10118261
10121424 10124587 10127750 10130914 10134078 10137243 10140408 10143574 10146740
10149907 10153074 10156242 10159411 10162579 10165749 10168918 10172089 10175259
10178431 10181602 10184775 10187947 10191121 10194294 10197469 10200643 10203819
10206994 10210171 10213347 10216525 10219702 10222880 10226059 10229238 10232418
10235598 10238779 10241960 10245142 10248324 10251507 10254690 10257873 10261058
10264242 10267427 10270613 10273799 10276986 10280173 10283361 10286549 10289737
10292926 10296116 10299306 10302497 10305688 10308879 10312071 10315264 10318457
10321651 10324845 10328039 10331234 10334430 10337626 10340822 10344019 10347217
10350415 10353614 10356813 10360012 10363212 10366413 10369614 10372815 10376017
10379220 10382423 10385626 10388830 10392035 10395239 10398445 10401651 10404857
10408064 10411272 10414480 10417688 10420897 10424106 10427316 10430527 10433738
10436949 10440161 10443373 10446586 10449800 10453014 10456228 10459443 10462658
10465874 10469091 10472307 10475525 10478743 10481961 10485180 10488399 10491619
10494839 10498060 10501281 10504503 10507726 10510948 10514172 10517395 10520620
10523845 10527070 10530296 10533522 10536749 10539976 10543204 10546432 10549661
10552890 10556120 10559350 10562581 10565812 10569044 10572276 10575509 10578742
10581976 10585210 10588444 10591680 10594915 10598152 10601388 10604625 10607863
10611101 10614340 10617579 10620819 10624059 10627300 10630541 10633782 10637024
10640267 10643510 10646754 10649998 10653243 10656488 10659733 10662979 10666226
10669473 10672721 10675969 10679217 10682466 10685716 10688966 10692217 10695468
10698719 10701971 10705224 10708477 10711730 10714984 10718239 10721494 10724750
10728006 10731262 10734519 10737777 10741035 10744293 10747552 10750811 10754071
10757332 10760593 10763854 10767116 10770379 10773642 10776905 10780169 10783434

10786699 10789964 10793230 10796496 10799763 10803031 10806299 10809567 10812836
10816105 10819375 10822646 10825916 10829188 10832460 10835732 10839005 10842278
10845552 10848826 10852101 10855377 10858653 10861929 10865206 10868483 10871761
10875039 10878318 10881597 10884877 10888157 10891438 10894719 10898001 10901284
10904566 10907850 10911133 10914418 10917702 10920988 10924273 10927560 10930846
10934134 10937421 10940710 10943998 10947288 10950577 10953867 10957158 10960449
10963741 10967033 10970326 10973619 10976913 10980207 10983502 10986797 10990093
10993389 10996685 10999983 11003280 11006578 11009877 11013176 11016476 11019776
11023076 11026378 11029679 11032981 11036284 11039587 11042891 11046195 11049499
11052804 11056110 11059416 11062723 11066030 11069337 11072645 11075954 11079263
11082572 11085882 11089193 11092504 11095816 11099128 11102440 11105753 11109067
11112381 11115695 11119010 11122326 11125642 11128958 11132275 11135593 11138910
11142229 11145548 11148867 11152187 11155508 11158829 11162150 11165472 11168795
11172117 11175441 11178765 11182089 11185414 11188740 11192065 11195392 11198719
11202046 11205374 11208702 11212031 11215361 11218690 11222021 11225352 11228683
11232015 11235347 11238680 11242013 11245347 11248682 11252016 11255352 11258688
11262024 11265361 11268698 11272036 11275374 11278713 11282052 11285392 11288732
11292073 11295414 11298756 11302098 11305441 11308784 11312128 11315472 11318817
11322162 11325508 11328854 11332201 11335548 11338896 11342244 11345593 11348942
11352292 11355642 11358992 11362344 11365695 11369047 11372400 11375753 11379107
11382461 11385816 11389171 11392527 11395883 11399239 11402596 11405954 11409312
11412671 11416030 11419390 11422750 11426110 11429471 11432833 11436195 11439558
11442921 11446284 11449648 11453013 11456378 11459744 11463110 11466476 11469843
11473211 11476579 11479947 11483316 11486686 11490056 11493426 11496797 11500169
11503541 11506913 11510286 11513660 11517034 11520408 11523783 11527159 11530535
11533911 11537288 11540666 11544044 11547422 11550801 11554180 11557560 11560941
11564322 11567703 11571085 11574467 11577850 11581234 11584618 11588002 11591387
11594772 11598158 11601544 11604931 11608319 11611707 11615095 11618484 11621873
11625263 11628653 11632044 11635435 11638827 11642220 11645612 11649006 11652400
11655794 11659189 11662584 11665980 11669376 11672773 11676170 11679568 11682966
11686365 11689764 11693164 11696564 11699965 11703366 11706768 11710170 11713573
11716976 11720380 11723784 11727189 11730594 11734000 11737406 11740813 11744220
11747628 11751036 11754444 11757854 11761263 11764673 11768084 11771495 11774907
11778319 11781732 11785145 11788559 11791973 11795387 11798802 11802218 11805634
11809051 11812468 11815885 11819304 11822722 11826141 11829561 11832981 11836402
11839823 11843244 11846666 11850089 11853512 11856935 11860359 11863784 11867209
11870635 11874061 11877487 11880914 11884342 11887770 11891198 11894627 11898057
11901487 11904917 11908348 11911780 11915212 11918644 11922077 11925511 11928945
11932379 11935814 11939249 11942685 11946122 11949559 11952996 11956434 11959873
11963311 11966751 11970191 11973631 11977072 11980514 11983955 11987398 11990841
11994284 11997728 12001172 12004617 12008063 12011508 12014955 12018402 12021849
12025297 12028745 12032194 12035643 12039093 12042544 12045994 12049446 12052897
12056350 12059803 12063256 12066710 12070164 12073619 12077074 12080530 12083986
12087443 12090900 12094358 12097816 12101275 12104734 12108194 12111654 12115115
12118576 12122038 12125500 12128963 12132426 12135890 12139354 12142819 12146284
12149749 12153216 12156682 12160150 12163617 12167085 12170554 12174023 12177493
12180963 12184434 12187905 12191376 12194849 12198321 12201794 12205268 12208742
12212217 12215692 12219167 12222644 12226120 12229597 12233075 12236553 12240032

12243511 12246990 12250470 12253951 12257432 12260913 12264395 12267878 12271361
12274845 12278329 12281813 12285298 12288784 12292270 12295756 12299243 12302731
12306219 12309707 12313196 12316686 12320176 12323666 12327157 12330649 12334141
12337633 12341126 12344620 12348113 12351608 12355103 12358598 12362094 12365591
12369088 12372585 12376083 12379581 12383080 12386580 12390080 12393580 12397081
12400582 12404084 12407586 12411089 12414593 12418097 12421601 12425106 12428611
12432117 12435623 12439130 12442638 12446145 12449654 12453163 12456672 12460182
12463692 12467203 12470714 12474226 12477738 12481251 12484764 12488278 12491792
12495307 12498822 12502338 12505854 12509371 12512888 12516406 12519924 12523443
12526962 12530482 12534002 12537523 12541044 12544566 12548088 12551611 12555134
12558658 12562182 12565707 12569232 12572758 12576284 12579810 12583338 12586865
12590393 12593922 12597451 12600981 12604511 12608042 12611573 12615104 12618637
12622169 12625702 12629236 12632770 12636305 12639840 12643375 12646911 12650448
12653985 12657523 12661061 12664599 12668138 12671678 12675218 12678759 12682300
12685841 12689383 12692926 12696469 12700012 12703556 12707101 12710646 12714191
12717737 12721284 12724831 12728378 12731926 12735475 12739024 12742573 12746123
12749674 12753225 12756776 12760328 12763881 12767434 12770987 12774541 12778095
12781650 12785206 12788762 12792318 12795875 12799433 12802990 12806549 12810108
12813667 12817227 12820787 12824348 12827910 12831472 12835034 12838597 12842160
12845724 12849288 12852853 12856419 12859984 12863551 12867118 12870685 12874253
12877821 12881390 12884959 12888529 12892099 12895670 12899241 12902813 12906385
12909958 12913532 12917105 12920680 12924254 12927830 12931405 12934982 12938558
12942136 12945713 12949292 12952870 12956450 12960029 12963609 12967190 12970771
12974353 12977935 12981518 12985101 12988685 12992269 12995854 12999439 13003025
13006611 13010197 13013785 13017372 13020960 13024549 13028138 13031728 13035318
13038909 13042500 13046091 13049684 13053276 13056869 13060463 13064057 13067652
13071247 13074842 13078438 13082035 13085632 13089230 13092828 13096426 13100025
13103625 13107225 13110825 13114427 13118028 13121630 13125233 13128836 13132439
13136043 13139648 13143253 13146858 13150464 13154071 13157678 13161285 13164893
13168502 13172111 13175720 13179330 13182941 13186552 13190163 13193775 13197387
13201000 13204614 13208228 13211842 13215457 13219073 13222688 13226305 13229922
13233539 13237157 13240776 13244394 13248014 13251634 13255254 13258875 13262496
13266118 13269741 13273363 13276987 13280611 13284235 13287860 13291485 13295111
13298737 13302364 13305992 13309619 13313248 13316876 13320506 13324136 13327766
13331397 13335028 13338660 13342292 13345925 13349558 13353192 13356826 13360461
13364096 13367732 13371368 13375005 13378642 13382280 13385918 13389557 13393196
13396836 13400476 13404117 13407758 13411400 13415042 13418685 13422328 13425971
13429616 13433260 13436905 13440551 13444197 13447844 13451491 13455139 13458787
13462436 13466085 13469734 13473385 13477035 13480686 13484338 13487990 13491643
13495296 13498949 13502603 13506258 13509913 13513569 13517225 13520881 13524538
13528196 13531854 13535513 13539172 13542831 13546491 13550152 13553813 13557474
13561137 13564799 13568462 13572126 13575790 13579454 13583119 13586785 13590451
13594117 13597784 13601452 13605120 13608788 13612457 13616127 13619797 13623467
13627138 13630809 13634481 13638154 13641827 13645500 13649174 13652849 13656524
13660199 13663875 13667551 13671228 13674906 13678584 13682262 13685941 13689620
13693300 13696980 13700661 13704343 13708025 13711707 13715390 13719073 13722757
13726441 13730126 13733812 13737497 13741184 13744871 13748558 13752246 13755934
13759623 13763312 13767002 13770692 13774383 13778074 13781766 13785458 13789151

13792845 13796538 13800233 13803927 13807623 13811318 13815015 13818711 13822409
13826106 13829805 13833503 13837203 13840902 13844602 13848303 13852004 13855706
13859408 13863111 13866814 13870518 13874222 13877927 13881632 13885338 13889044
13892750 13896458 13900165 13903873 13907582 13911291 13915001 13918711 13922422
13926133 13929844 13933556 13937269 13940982 13944696 13948410 13952125 13955840
13959555 13963271 13966988 13970705 13974423 13978141 13981859 13985578 13989298
13993018 13996738 14000459 14004181 14007903 14011626 14015349 14019072 14022796
14026521 14030246 14033971 14037697 14041424 14045151 14048878 14052606 14056335
14060064 14063793 14067523 14071254 14074985 14078716 14082448 14086180 14089913
14093647 14097381 14101115 14104850 14108586 14112321 14116058 14119795 14123532
14127270 14131009 14134747 14138487 14142227 14145967 14149708 14153449 14157191
14160934 14164676 14168420 14172164 14175908 14179653 14183398 14187144 14190890
14194637 14198385 14202132 14205881 14209630 14213379 14217129 14220879 14224630
14228381 14232133 14235885 14239638 14243391 14247145 14250899 14254654 14258409
14262165 14265921 14269678 14273435 14277193 14280951 14284710 14288469 14292229
14295989 14299750 14303511 14307273 14311035 14314798 14318561 14322325 14326089
14329854 14333619 14337384 14341151 14344917 14348684 14352452 14356220 14359989
14363758 14367528 14371298 14375069 14378840 14382611 14386383 14390156 14393929
14397703 14401477 14405252 14409027 14412802 14416578 14420355 14424132 14427910
14431688 14435466 14439245 14443025 14446805 14450585 14454367 14458148 14461930
14465713 14469496 14473279 14477063 14480848 14484633 14488418 14492204 14495991
14499778 14503565 14507353 14511142 14514931 14518720 14522510 14526301 14530092
14533883 14537675 14541468 14545261 14549054 14552848 14556642 14560437 14564233
14568029 14571825 14575622 14579420 14583217 14587016 14590815 14594614 14598414
14602214 14606015 14609817 14613619 14617421 14621224 14625027 14628831 14632635
14636440 14640246 14644052 14647858 14651665 14655472 14659280 14663088 14666897
14670706 14674516 14678327 14682137 14685949 14689760 14693573 14697386 14701199
14705013 14708827 14712642 14716457 14720273 14724089 14727906 14731723 14735541
14739359 14743178 14746997 14750817 14754637 14758458 14762279 14766101 14769923
14773746 14777569 14781392 14785217 14789041 14792867 14796692 14800518 14804345
14808172 14812000 14815828 14819657 14823486 14827316 14831146 14834976 14838808
14842639 14846471 14850304 14854137 14857971 14861805 14865639 14869475 14873310
14877146 14880983 14884820 14888658 14892496 14896334 14900173 14904013 14907853
14911694 14915535 14919376 14923218 14927061 14930904 14934747 14938591 14942436
14946281 14950127 14953973 14957819 14961666 14965514 14969362 14973210 14977059
14980909 14984759 14988609 14992460 14996312 15000164 15004016 15007869 15011722
15015576 15019431 15023286 15027141 15030997 15034854 15038711 15042568 15046426
15050284 15054143 15058003 15061863 15065723 15069584 15073445 15077307 15081170
15085032 15088896 15092760 15096624 15100489 15104354 15108220 15112087 15115953
15119821 15123689 15127557 15131426 15135295 15139165 15143035 15146906 15150777
15154649 15158522 15162394 15166268 15170141 15174016 15177891 15181766 15185642
15189518 15193395 15197272 15201150 15205028 15208907 15212786 15216666 15220546
15224427 15228308 15232190 15236072 15239955 15243838 15247722 15251606 15255491
15259376 15263261 15267148 15271034 15274922 15278809 15282697 15286586 15290475
15294365 15298255 15302146 15306037 15309929 15313821 15317713 15321606 15325500
15329394 15333289 15337184 15341080 15344976 15348872 15352769 15356667 15360565
15364464 15368363 15372262 15376163 15380063 15383964 15387866 15391768 15395670
15399574 15403477 15407381 15411286 15415191 15419096 15423002 15426909 15430816

15434723 15438631 15442540 15446449 15450358 15454268 15458179 15462090 15466001
15469913 15473826 15477739 15481652 15485566 15489481 15493396 15497311 15501227
15505144 15509061 15512978 15516896 15520814 15524733 15528653 15532573 15536493
15540414 15544336 15548257 15552180 15556103 15560026 15563950 15567874 15571799
15575725 15579651 15583577 15587504 15591431 15595359 15599287 15603216 15607146
15611075 15615006 15618937 15622868 15626800 15630732 15634665 15638598 15642532
15646466 15650401 15654336 15658272 15662209 15666145 15670083 15674020 15677959
15681898 15685837 15689777 15693717 15697658 15701599 15705541 15709483 15713426
15717369 15721313 15725257 15729202 15733147 15737092 15741039 15744985 15748933
15752880 15756828 15760777 15764726 15768676 15772626 15776577 15780528 15784480
15788432 15792385 15796338 15800291 15804246 15808200 15812155 15816111 15820067
15824024 15827981 15831939 15835897 15839855 15843814 15847774 15851734 15855695
15859656 15863617 15867579 15871542 15875505 15879469 15883433 15887397 15891362
15895328 15899294 15903261 15907228 15911195 15915163 15919132 15923101 15927070
15931040 15935011 15938982 15942953 15946925 15950898 15954871 15958844 15962818
15966793 15970768 15974743 15978719 15982696 15986673 15990650 15994628 15998607
16002586 16006565 16010545 16014525 16018506 16022488 16026470 16030452 16034435
16038419 16042402 16046387 16050372 16054357 16058343 16062329 16066316 16070304
16074292 16078280 16082269 16086258 16090248 16094238 16098229 16102221 16106213
16110205 16114198 16118191 16122185 16126179 16130174 16134169 16138165 16142161
16146158 16150156 16154153 16158152 16162151 16166150 16170150 16174150 16178151
16182152 16186154 16190156 16194159 16198162 16202166 16206170 16210175 16214180
16218186 16222192 16226199 16230206 16234214 16238222 16242231 16246240 16250250
16254260 16258270 16262282 16266293 16270305 16274318 16278331 16282345 16286359
16290374 16294389 16298405 16302421 16306438 16310455 16314472 16318490 16322509
16326528 16330548 16334568 16338589 16342610 16346631 16350653 16354676 16358699
16362723 16366747 16370771 16374797 16378822 16382848 16386875 16390902 16394929
16398957 16402986 16407015 16411045 16415075 16419105 16423136 16427168 16431200
16435232 16439265 16443299 16447333 16451367 16455402 16459438 16463474 16467510
16471547 16475585 16479623 16483661 16487700 16491740 16495780 16499820 16503861
16507903 16511945 16515987 16520030 16524073 16528117 16532162 16536207 16540252
16544298 16548345 16552391 16556439 16560487 16564535 16568584 16572633 16576683
16580734 16584785 16588836 16592888 16596940 16600993 16605046 16609100 16613155
16617209 16621265 16625321 16629377 16633434 16637491 16641549 16645607 16649666
16653725 16657785 16661846 16665906 16669968 16674029 16678092 16682155 16686218
16690282 16694346 16698411 16702476 16706542 16710608 16714675 16718742 16722810
16726878 16730947 16735016 16739086 16743156 16747227 16751298 16755370 16759442
16763515 16767588 16771661 16775736 16779810 16783885 16787961 16792037 16796114
16800191 16804269 16808347 16812426 16816505 16820585 16824665 16828745 16832826
16836908 16840990 16845073 16849156 16853240 16857324 16861408 16865493 16869579
16873665 16877752 16881839 16885926 16890015 16894103 16898192 16902282 16906372
16910462 16914553 16918645 16922737 16926830 16930923 16935016 16939110 16943205
16947300 16951395 16955491 16959588 16963685 16967782 16971880 16975979 16980078
16984178 16988277 16992378 16996479 17000580 17004682 17008785 17012888 17016991
17021095 17025200 17029305 17033410 17037516 17041623 17045730 17049837 17053945
17058053 17062162 17066272 17070382 17074492 17078603 17082714 17086826 17090939
17095051 17099165 17103279 17107393 17111508 17115623 17119739 17123856 17127972
17132090 17136208 17140326 17144445 17148564 17152684 17156804 17160925 17165047

17169168 17173291 17177414 17181537 17185661 17189785 17193910 17198035 17202161
17206287 17210414 17214541 17218669 17222797 17226926 17231055 17235185 17239315
17243446 17247577 17251709 17255841 17259974 17264107 17268241 17272375 17276510
17280645 17284781 17288917 17293054 17297191 17301329 17305467 17309606 17313745
17317884 17322025 17326165 17330306 17334448 17338590 17342733 17346876 17351020
17355164 17359309 17363454 17367599 17371745 17375892 17380039 17384187 17388335
17392484 17396633 17400782 17404932 17409083 17413234 17417386 17421538 17425690
17429843 17433997 17438151 17442306 17446461 17450616 17454772 17458929 17463086
17467243 17471401 17475560 17479719 17483879 17488039 17492199 17496360 17500522
17504684 17508846 17513009 17517173 17521337 17525501 17529666 17533832 17537998
17542164 17546331 17550498 17554666 17558835 17563004 17567173 17571343 17575514
17579685 17583856 17588028 17592200 17596373 17600547 17604721 17608895 17613070
17617245 17621421 17625598 17629775 17633952 17638130 17642308 17646487 17650667
17654846 17659027 17663208 17667389 17671571 17675753 17679936 17684119 17688303
17692488 17696672 17700858 17705044 17709230 17713417 17717604 17721792 17725980
17730169 17734358 17738548 17742738 17746929 17751120 17755312 17759504 17763697
17767890 17772084 17776278 17780473 17784668 17788864 17793060 17797257 17801454
17805652 17809850 17814049 17818248 17822448 17826648 17830849 17835050 17839252
17843454 17847657 17851860 17856064 17860268 17864472 17868678 17872883 17877089
17881296 17885503 17889711 17893919 17898128 17902337 17906546 17910757 17914967
17919178 17923390 17927602 17931815 17936028 17940241 17944456 17948670 17952885
17957101 17961317 17965533 17969751 17973968 17978186 17982405 17986624 17990843
17995063 17999284 18003505 18007727 18011949 18016171 18020394 18024618 18028842
18033066 18037291 18041517 18045743 18049969 18054196 18058424 18062652 18066880
18071109 18075339 18079569 18083799 18088030 18092262 18096494 18100726 18104959
18109193 18113427 18117661 18121896 18126131 18130367 18134604 18138841 18143078
18147316 18151554 18155793 18160033 18164273 18168513 18172754 18176995 18181237
18185480 18189722 18193966 18198210 18202454 18206699 18210944 18215190 18219436
18223683 18227931 18232179 18236427 18240676 18244925 18249175 18253425 18257676
18261927 18266179 18270431 18274684 18278938 18283191 18287446 18291700 18295956
18300211 18304468 18308725 18312982 18317240 18321498 18325757 18330016 18334276
18338536 18342797 18347058 18351320 18355582 18359845 18364108 18368371 18372636
18376900 18381166 18385431 18389697 18393964 18398231 18402499 18406767 18411036
18415305 18419575 18423845 18428116 18432387 18436658 18440931 18445203 18449476
18453750 18458024 18462299 18466574 18470850 18475126 18479402 18483679 18487957
18492235 18496514 18500793 18505072 18509353 18513633 18517914 18522196 18526478
18530760 18535044 18539327 18543611 18547896 18552181 18556466 18560752 18565039
18569326 18573613 18577902 18582190 18586479 18590769 18595059 18599349 18603640
18607932 18612224 18616516 18620809 18625103 18629397 18633691 18637986 18642282
18646578 18650874 18655171 18659468 18663766 18668065 18672364 18676663 18680963
18685264 18689565 18693866 18698168 18702470 18706773 18711077 18715381 18719685
18723990 18728295 18732601 18736908 18741214 18745522 18749830 18754138 18758447
18762756 18767066 18771377 18775687 18779999 18784311 18788623 18792936 18797249
18801563 18805877 18810192 18814507 18818823 18823140 18827456 18831774 18836091
18840410 18844729 18849048 18853368 18857688 18862009 18866330 18870652 18874974
18879297 18883620 18887944 18892268 18896593 18900918 18905244 18909570 18913897
18918224 18922552 18926880 18931209 18935538 18939868 18944198 18948529 18952860
18957191 18961524 18965856 18970189 18974523 18978857 18983192 18987527 18991863

18996199 19000536 19004873 19009210 19013549 19017887 19022226 19026566 19030906
19035247 19039588 19043930 19048272 19052614 19056957 19061301 19065645 19069990
19074335 19078680 19083026 19087373 19091720 19096068 19100416 19104764 19109113
19113463 19117813 19122164 19126515 19130866 19135218 19139571 19143924 19148277
19152631 19156986 19161341 19165696 19170052 19174409 19178766 19183123 19187481
19191840 19196199 19200558 19204918 19209279 19213640 19218001 19222363 19226726
19231089 19235452 19239816 19244181 19248545 19252911 19257277 19261643 19266010
19270378 19274746 19279114 19283483 19287852 19292222 19296593 19300964 19305335
19309707 19314079 19318452 19322825 19327199 19331574 19335949 19340324 19344700
19349076 19353453 19357830 19362208 19366587 19370965 19375345 19379725 19384105
19388486 19392867 19397249 19401631 19406014 19410397 19414781 19419165 19423550
19427935 19432321 19436707 19441094 19445482 19449869 19454258 19458646 19463036
19467425 19471816 19476206 19480598 19484989 19489382 19493774 19498168 19502561
19506955 19511350 19515745 19520141 19524537 19528934 19533331 19537729 19542127
19546526 19550925 19555325 19559725 19564125 19568527 19572928 19577330 19581733
19586136 19590540 19594944 19599349 19603754 19608159 19612566 19616972 19621379
19625787 19630195 19634604 19639013 19643422 19647833 19652243 19656654 19661066
19665478 19669890 19674304 19678717 19683131 19687546 19691961 19696376 19700792
19705209 19709626 19714044 19718462 19722880 19727299 19731719 19736139 19740559
19744980 19749402 19753824 19758246 19762669 19767093 19771517 19775941 19780366
19784792 19789218 19793644 19798071 19802499 19806927 19811355 19815784 19820213
19824643 19829074 19833505 19837936 19842368 19846801 19851234 19855667 19860101
19864535 19868970 19873406 19877842 19882278 19886715 19891152 19895590 19900028
19904467 19908907 19913347 19917787 19922228 19926669 19931111 19935553 19939996
19944440 19948883 19953328 19957773 19962218 19966664 19971110 19975557 19980004
19984452 19988900 19993349 19997799 20002248 20006699 20011149 20015601 20020052
20024505 20028958 20033411 20037865 20042319 20046774 20051229 20055685 20060141
20064598 20069055 20073513 20077971 20082430 20086889 20091349 20095809 20100269
20104731 20109192 20113655 20118117 20122581 20127044 20131508 20135973 20140438
20144904 20149370 20153837 20158304 20162772 20167240 20171708 20176178 20180647
20185117 20189588 20194059 20198531 20203003 20207476 20211949 20216422 20220896
20225371 20229846 20234322 20238798 20243274 20247752 20252229 20256707 20261186
20265665 20270144 20274625 20279105 20283586 20288068 20292550 20297032 20301515
20305999 20310483 20314967 20319453 20323938 20328424 20332911 20337398 20341885
20346373 20350862 20355351 20359840 20364330 20368821 20373312 20377803 20382295
20386788 20391281 20395774 20400268 20404762 20409257 20413753 20418249 20422745
20427242 20431740 20436238 20440736 20445235 20449734 20454234 20458735 20463236
20467737 20472239 20476741 20481244 20485748 20490252 20494756 20499261 20503766
20508272 20512778 20517285 20521793 20526301 20530809 20535318 20539827 20544337
20548847 20553358 20557869 20562381 20566894 20571406 20575920 20580434 20584948
20589463 20593978 20598494 20603010 20607527 20612044 20616562 20621080 20625599
20630118 20634638 20639158 20643679 20648200 20652722 20657244 20661767 20666290
20670814 20675338 20679863 20684388 20688914 20693440 20697967 20702494 20707022
20711550 20716079 20720608 20725138 20729668 20734199 20738730 20743262 20747794
20752326 20756860 20761393 20765927 20770462 20774997 20779533 20784069 20788606
20793143 20797681 20802219 20806757 20811296 20815836 20820376 20824917 20829458
20833999 20838542 20843084 20847627 20852171 20856715 20861260 20865805 20870350
20874896 20879443 20883990 20888538 20893086 20897634 20902183 20906733 20911283

20915833 20920384 20924936 20929488 20934040 20938594 20943147 20947701 20952256
20956811 20961366 20965922 20970479 20975036 20979593 20984151 20988710 20993269
20997828 21002388 21006948 21011509 21016071 21020633 21025195 21029758 21034322
21038886 21043450 21048015 21052580 21057146 21061713 21066280 21070847 21075415
21079983 21084552 21089122 21093692 21098262 21102833 21107404 21111976 21116549
21121121 21125695 21130269 21134843 21139418 21143993 21148569 21153145 21157722
21162300 21166878 21171456 21176035 21180614 21185194 21189774 21194355 21198936
21203518 21208100 21212683 21217267 21221850 21226435 21231019 21235605 21240190
21244777 21249363 21253951 21258539 21263127 21267716 21272305 21276895 21281485
21286076 21290667 21295259 21299851 21304443 21309037 21313630 21318225 21322819
21327415 21332010 21336606 21341203 21345800 21350398 21354996 21359595 21364194
21368794 21373394 21377995 21382596 21387197 21391800 21396402 21401005 21405609
21410213 21414818 21419423 21424029 21428635 21433241 21437849 21442456 21447064
21451673 21456282 21460892 21465502 21470112 21474723 21479335 21483947 21488560
21493173 21497786 21502400 21507015 21511630 21516246 21520862 21525478 21530095
21534713 21539331 21543949 21548568 21553188 21557808 21562428 21567049 21571671
21576293 21580915 21585538 21590162 21594786 21599410 21604035 21608661 21613287
21617913 21622540 21627168 21631796 21636424 21641053 21645683 21650313 21654943
21659574 21664205 21668837 21673470 21678103 21682736 21687370 21692005 21696639
21701275 21705911 21710547 21715184 21719822 21724459 21729098 21733737 21738376
21743016 21747656 21752297 21756939 21761580 21766223 21770866 21775509 21780153
21784797 21789442 21794087 21798733 21803380 21808026 21812674 21817322 21821970
21826619 21831268 21835918 21840568 21845219 21849870 21854522 21859174 21863827
21868480 21873134 21877788 21882443 21887098 21891754 21896410 21901067 21905724
21910382 21915040 21919699 21924358 21929018 21933678 21938339 21943000 21947662
21952324 21956987 21961650 21966314 21970978 21975643 21980308 21984974 21989640
21994306 21998974 22003641 22008310 22012978 22017647 22022317 22026987 22031658
22036329 22041001 22045673 22050345 22055019 22059692 22064366 22069041 22073716
22078392 22083068 22087744 22092421 22097099 22101777 22106456 22111135 22115814
22120495 22125175 22129856 22134538 22139220 22143902 22148585 22153269 22157953
22162638 22167323 22172008 22176694 22181381 22186068 22190755 22195443 22200132
22204821 22209510 22214200 22218891 22223582 22228273 22232965 22237658 22242351
22247044 22251738 22256433 22261128 22265823 22270519 22275216 22279913 22284610
22289308 22294007 22298705 22303405 22308105 22312805 22317506 22322208 22326910
22331612 22336315 22341018 22345722 22350427 22355131 22359837 22364543 22369249
22373956 22378663 22383371 22388080 22392789 22397498 22402208 22406918 22411629
22416340 22421052 22425765 22430477 22435191 22439905 22444619 22449334 22454049
22458765 22463481 22468198 22472915 22477633 22482351 22487070 22491790 22496509
22501230 22505950 22510672 22515393 22520116 22524838 22529562 22534286 22539010
22543735 22548460 22553186 22557912 22562639 22567366 22572093 22576822 22581550
22586280 22591009 22595740 22600470 22605202 22609933 22614665 22619398 22624131
22628865 22633599 22638334 22643069 22647805 22652541 22657278 22662015 22666752
22671491 22676229 22680968 22685708 22690448 22695189 22699930 22704672 22709414
22714156 22718899 22723643 22728387 22733132 22737877 22742622 22747369 22752115
22756862 22761610 22766358 22771106 22775856 22780605 22785355 22790106 22794857
22799608 22804360 22809113 22813866 22818620 22823374 22828128 22832883 22837639
22842395 22847151 22851908 22856666 22861424 22866182 22870941 22875701 22880461
22885221 22889982 22894744 22899506 22904268 22909031 22913795 22918559 22923323

22928088 22932854 22937620 22942386 22947153 22951920 22956688 22961457 22966226 22970995 22975765 22980535 22985306 22990078 22994850 22999622 23004395 23009168 23013942 23018717 23023492 23028267 23033043 23037819 23042596 23047373 23052151 23056930 23061709 23066488 23071268 23076048 23080829 23085610 23090392 23095174 23099957 23104741 23109524 23114309 23119094 23123879 23128665 23133451 23138238 23143025 23147813 23152601 23157390 23162179 23166969 23171759 23176550 23181341 23186133 23190925 23195718 23200511 23205305 23210099 23214894 23219689 23224485 23229281 23234078 23238875 23243673 23248471 23253270 23258069 23262869 23267669 23272470 23277271 23282073 23286875 23291678 23296481 23301284 23306089 23310893 23315698 23320504 23325310 23330117 23334924 23339732 23344540 23349348 23354158 23358967 23363777 23368588 23373399 23378211 23383023 23387836 23392649 23397462 23402276 23407091 23411906 23416722 23421538 23426354 23431171 23435989 23440807 23445626 23450445 23455264 23460084 23464905 23469726 23474547 23479369 23484192 23489015 23493839 23498663 23503487 23508312 23513138 23517964 23522790 23527617 23532445 23537273 23542101 23546930 23551760 23556590 23561420 23566251 23571083 23575915 23580747 23585580 23590414 23595248 23600082 23604917 23609752 23614588 23619425 23624262 23629099 23633937 23638775 23643614 23648454 23653294 23658134 23662975 23667816 23672658 23677501 23682344 23687187 23692031 23696875 23701720 23706566 23711411 23716258 23721105 23725952 23730800 23735648 23740497 23745346 23750196 23755047 23759897 23764749 23769601 23774453 23779306 23784159 23789013 23793867 23798722 23803577 23808433 23813289 23818146 23823004 23827861 23832720 23837578 23842438 23847297 23852158 23857018 23861880 23866741 23871604 23876466 23881330 23886193 23891058 23895922 23900788 23905653 23910520 23915386 23920254 23925121 23929990 23934858 23939727 23944597 23949467 23954338 23959209 23964081 23968953 23973826 23978699 23983573 23988447 23993321 23998197 24003072 24007948 24012825 24017702 24022580 24027458 24032337 24037216 24042095 24046976 24051856 24056737 24061619 24066501 24071384 24076267 24081150 24086034 24090919 24095804 24100690 24105576 24110462 24115349 24120237 24125125 24130014 24134903 24139792 24144682 24149573 24154464 24159355 24164247 24169140 24174033 24178927 24183821 24188715 24193610 24198506 24203402 24208298 24213195 24218093 24222991 24227889 24232788 24237688 24242588 24247488 24252389 24257291 24262193 24267095 24271998 24276901 24281805 24286710 24291615 24296520 24301426 24306333 24311239 24316147 24321055 24325963 24330872 24335782 24340692 24345602 24350513 24355424 24360336 24365249 24370161 24375075 24379989 24384903 24389818 24394733 24399649 24404566 24409482 24414400 24419318 24424236 24429155 24434074 24438994 24443914 24448835 24453756 24458678 24463601 24468523 24473447 24478371 24483295 24488220 24493145 24498071 24502997 24507924 24512851 24517779 24522707 24527636 ;

setarray 10000 wilc2_5 0 0 0 0 0 0 2 3 5 8 10 13 17 21 25 29 34 40 46 52 58 65 73 81 89 98 107 116 126 137 147 159 170 182 195 208 221 235 249 264 279 294 310 327 343 361 378 396 415 434 454 474 494 515 536 558 580 602 625 649 673 697 722 747 773 799 825 852 880 908 936 965 994 1024 1054 1084 1115 1147 1179 1211 1244 1277 1311 1345 1380 1415 1451 1487 1523 1560 1598 1636 1674 1713 1752 1792 1832 1872 1913 1955 1997 2039 2082 2126 2169 2214 2258 2304 2349 2395 2442 2489 2536 2584 2633 2682 2731 2781 2831 2882 2933 2984 3036 3089 3142 3195 3249 3304 3359 3414 3470 3526 3583 3640 3698 3756 3814 3873 3933 3993 4053 4114 4175 4237 4299 4362 4425 4489 4553 4618 4683 4748 4814 4881 4948 5015 5083 5151 5220 5289 5359 5429 5500 5571 5643 5715 5787 5860 5934 6008 6082 6157 6232 6308 6385 6461 6538 6616 6694 6773 6852 6932 7012 7092 7173 7254 7336 7419 7502 7585

7669 7753 7838 7923 8008 8094 8181 8268 8356 8444 8532 8621 8710 8800 8891 8982 9073
9165 9257 9349 9443 9536 9630 9725 9820 9916 10012 10108 10205 10302 10400 10499
10597 10697 10797 10897 10997 11099 11200 11302 11405 11508 11612 11716 11820 11925
12031 12136 12243 12350 12457 12565 12673 12782 12891 13001 13111 13222 13333 13444
13556 13669 13782 13895 14009 14124 14239 14354 14470 14586 14703 14820 14938 15056
15175 15294 15414 15534 15654 15775 15897 16019 16141 16264 16388 16512 16636 16761
16886 17012 17138 17265 17392 17520 17648 17777 17906 18035 18165 18296 18427 18559
18691 18823 18956 19089 19223 19358 19492 19628 19763 19900 20036 20174 20311 20449
20588 20727 20867 21007 21147 21288 21430 21572 21714 21857 22001 22144 22289 22434
22579 22725 22871 23018 23165 23312 23461 23609 23758 23908 24058 24209 24360 24511
24663 24815 24968 25122 25276 25430 25585 25740 25896 26052 26209 26366 26524 26682
26841 27000 27159 27319 27480 27641 27803 27965 28127 28290 28453 28617 28782 28946
29112 29278 29444 29611 29778 29946 30114 30282 30452 30621 30791 30962 31133 31305
31477 31649 31822 31996 32169 32344 32519 32694 32870 33046 33223 33400 33578 33756
33935 34114 34294 34474 34655 34836 35017 35200 35382 35565 35749 35933 36117 36302
36487 36673 36860 37047 37234 37422 37610 37799 37988 38178 38368 38559 38750 38941
39134 39326 39519 39713 39907 40101 40296 40492 40688 40884 41081 41279 41477 41675
41874 42073 42273 42473 42674 42875 43077 43279 43482 43685 43889 44093 44297 44502
44708 44914 45120 45327 45535 45743 45951 46160 46370 46579 46790 47001 47212 47424
47636 47849 48062 48276 48490 48705 48920 49135 49352 49568 49785 50003 50221 50439
50658 50878 51098 51318 51539 51761 51983 52205 52428 52651 52875 53099 53324 53549
53775 54001 54228 54455 54683 54911 55139 55369 55598 55828 56059 56290 56521 56753
56986 57219 57452 57686 57920 58155 58391 58626 58863 59100 59337 59575 59813 60052
60291 60530 60771 61011 61252 61494 61736 61979 62222 62465 62709 62954 63199 63444
63690 63937 64183 64431 64679 64927 65176 65425 65675 65925 66176 66427 66679 66931
67184 67437 67691 67945 68199 68455 68710 68966 69223 69480 69737 69995 70254 70513
70772 71032 71292 71553 71815 72076 72339 72602 72865 73129 73393 73658 73923 74189
74455 74721 74989 75256 75524 75793 76062 76332 76602 76872 77143 77415 77687 77959
78232 78505 78779 79054 79329 79604 79880 80156 80433 80710 80988 81266 81545 81824
82104 82384 82665 82946 83227 83510 83792 84075 84359 84643 84927 85212 85498 85784
86070 86357 86645 86932 87221 87510 87799 88089 88379 88670 88961 89253 89545 89838
90131 90425 90719 91014 91309 91605 91901 92197 92494 92792 93090 93389 93688 93987
94287 94587 94888 95190 95492 95794 96097 96400 96704 97009 97313 97619 97924 98231
98537 98845 99152 99461 99769 100078 100388 100698 101009 101320 101632 101944
102256 102569 102883 103197 103511 103826 104142 104458 104774 105091 105408
105726 106044 106363 106683 107002 107323 107643 107965 108286 108609 108931
109255 109578 109902 110227 110552 110878 111204 111530 111858 112185 112513
112842 113171 113500 113830 114161 114491 114823 115155 115487 115820 116153
116487 116822 117156 117492 117827 118164 118500 118838 119175 119514 119852
120191 120531 120871 121212 121553 121894 122237 122579 122922 123266 123610
123954 124299 124645 124991 125337 125684 126031 126379 126728 127076 127426
127776 128126 128477 128828 129180 129532 129885 130238 130592 130946 131300
131656 132011 132367 132724 133081 133439 133797 134155 134514 134874 135234
135594 135955 136317 136679 137041 137404 137767 138131 138495 138860 139226
139591 139958 140325 140692 141060 141428 141797 142166 142535 142906 143276
143647 144019 144391 144764 145137 145510 145884 146259 146634 147010 147386
147762 148139 148516 148894 149273 149652 150031 150411 150791 151172 151553
151935 152318 152700 153084 153467 153852 154236 154622 155007 155393 155780

156167 156555 156943 157332 157721 158110 158500 158891 159282 159674 160066
160458 160851 161244 161638 162033 162428 162823 163219 163615 164012 164410
164807 165206 165605 166004 166404 166804 167205 167606 168008 168410 168812
169216 169619 170023 170428 170833 171239 171645 172051 172458 172866 173274
173683 174091 174501 174911 175321 175732 176144 176556 176968 177381 177794
178208 178623 179037 179453 179868 180285 180701 181119 181536 181955 182373
182793 183212 183632 184053 184474 184896 185318 185741 186164 186587 187011
187436 187861 188286 188712 189139 189566 189993 190421 190850 191279 191708
192138 192568 192999 193430 193862 194295 194727 195161 195594 196029 196463
196899 197334 197771 198207 198644 199082 199520 199959 200398 200838 201278
201718 202159 202601 203043 203485 203928 204372 204816 205260 205705 206151
206597 207043 207490 207937 208385 208834 209282 209732 210182 210632 211083
211534 211986 212438 212891 213344 213798 214252 214706 215162 215617 216073
216530 216987 217445 217903 218361 218820 219280 219740 220200 220661 221123
221585 222047 222510 222974 223438 223902 224367 224832 225298 225765 226231
226699 227167 227635 228104 228573 229043 229513 229984 230455 230927 231399
231871 232345 232818 233292 233767 234242 234718 235194 235670 236147 236625
237103 237581 238060 238540 239020 239500 239981 240463 240945 241427 241910
242393 242877 243362 243846 244332 244818 245304 245791 246278 246766 247254
247743 248232 248722 249212 249703 250194 250685 251178 251670 252163 252657
253151 253646 254141 254636 255132 255629 256126 256623 257121 257620 258119
258618 259118 259618 260119 260621 261123 261625 262128 262631 263135 263639
264144 264649 265155 265661 266168 266675 267183 267691 268200 268709 269219
269729 270240 270751 271263 271775 272287 272800 273314 273828 274342 274858
275373 275889 276406 276923 277440 277958 278476 278995 279515 280035 280555
281076 281597 282119 282641 283164 283688 284211 284736 285260 285786 286311
286838 287364 287892 288419 288948 289476 290005 290535 291065 291596 292127
292658 293191 293723 294256 294790 295324 295858 296393 296929 297465 298001
298538 299076 299614 300152 300691 301230 301770 302311 302851 303393 303935
304477 305020 305563 306107 306651 307196 307741 308287 308833 309380 309927
310475 311023 311571 312121 312670 313220 313771 314322 314874 315426 315978
316531 317085 317639 318193 318748 319304 319860 320416 320973 321531 322089
322647 323206 323765 324325 324885 325446 326008 326569 327132 327695 328258
328822 329386 329951 330516 331082 331648 332215 332782 333349 333918 334486
335055 335625 336195 336766 337337 337908 338480 339053 339626 340200 340774
341348 341923 342499 343074 343651 344228 344805 345383 345962 346541 347120
347700 348280 348861 349442 350024 350606 351189 351773 352356 352941 353525
354111 354696 355282 355869 356456 357044 357632 358221 358810 359400 359990
360580 361171 361763 362355 362948 363541 364134 364728 365323 365918 366513
367109 367706 368303 368900 369498 370096 370695 371295 371894 372495 373096
373697 374299 374901 375504 376107 376711 377315 377920 378525 379131 379737
380344 380951 381559 382167 382776 383385 383994 384605 385215 385826 386438
387050 387663 388276 388889 389503 390118 390733 391348 391964 392581 393198
393815 394433 395051 395670 396290 396910 397530 398151 398772 399394 400016
400639 401262 401886 402511 403135 403761 404386 405013 405639 406267 406894
407522 408151 408780 409410 410040 410671 411302 411933 412565 413198 413831
414465 415099 415733 416368 417004 417640 418276 418913 419551 420189 420827
421466 422106 422745 423386 424027 424668 425310 425952 426595 427238 427882

428527 429171 429817 430462 431109 431755 432403 433050 433699 434347 434997
435646 436296 436947 437598 438250 438902 439555 440208 440861 441515 442170
442825 443480 444136 444793 445450 446108 446765 447424 448083 448742 449402
450063 450724 451385 452047 452709 453372 454036 454699 455364 456029 456694
457360 458026 458693 459360 460028 460696 461365 462034 462704 463374 464045
464716 465387 466060 466732 467405 468079 468753 469428 470103 470778 471454
472131 472808 473486 474164 474842 475521 476201 476881 477561 478242 478923
479605 480288 480971 481654 482338 483022 483707 484392 485078 485765 486451
487139 487827 488515 489204 489893 490583 491273 491964 492655 493347 494039
494731 495425 496118 496812 497507 498202 498898 499594 500290 500988 501685
502383 503082 503781 504480 505180 505881 506582 507283 507985 508688 509391
510094 510798 511502 512207 512913 513619 514325 515032 515739 516447 517155
517864 518574 519283 519994 520704 521416 522127 522840 523552 524266 524979
525693 526408 527123 527839 528555 529272 529989 530707 531425 532143 532862
533582 534302 535023 535744 536465 537187 537910 538633 539356 540080 540805
541530 542255 542981 543707 544434 545162 545890 546618 547347 548076 548806
549536 550267 550998 551730 552463 553195 553929 554662 555397 556131 556867
557602 558338 559075 559812 560550 561288 562027 562766 563506 564246 564986
565727 566469 567211 567954 568697 569440 570184 570929 571674 572419 573165
573912 574659 575406 576154 576903 577652 578401 579151 579901 580652 581404
582155 582908 583661 584414 585168 585922 586677 587432 588188 588944 589701
590458 591216 591974 592733 593492 594252 595012 595773 596534 597295 598058
598820 599583 600347 601111 601876 602641 603406 604172 604939 605706 606473
607241 608010 608779 609548 610318 611089 611860 612631 613403 614175 614948
615722 616496 617270 618045 618820 619596 620372 621149 621927 622704 623483
624261 625041 625820 626601 627381 628163 628944 629727 630509 631292 632076
632860 633645 634430 635216 636002 636788 637575 638363 639151 639940 640729
641518 642308 643099 643890 644681 645473 646266 647059 647852 648646 649441
650236 651031 651827 652623 653420 654218 655016 655814 656613 657412 658212
659012 659813 660614 661416 662218 663021 663824 664628 665432 666237 667042
667848 668654 669461 670268 671076 671884 672693 673502 674311 675121 675932
676743 677555 678367 679179 679992 680806 681620 682434 683249 684065 684881
685697 686514 687332 688150 688968 689787 690607 691426 692247 693068 693889
694711 695533 696356 697180 698003 698828 699652 700478 701304 702130 702957
703784 704612 705440 706268 707098 707927 708758 709588 710419 711251 712083
712916 713749 714583 715417 716251 717086 717922 718758 719595 720432 721269
722107 722946 723785 724624 725464 726305 727146 727987 728829 729672 730514
731358 732202 733046 733891 734736 735582 736429 737276 738123 738971 739819
740668 741517 742367 743217 744068 744919 745771 746623 747476 748329 749183
750037 750892 751747 752602 753459 754315 755172 756030 756888 757747 758606
759465 760326 761186 762047 762909 763771 764633 765496 766360 767224 768088
768953 769819 770685 771551 772418 773285 774153 775022 775891 776760 777630
778500 779371 780242 781114 781987 782859 783733 784607 785481 786356 787231
788107 788983 789860 790737 791615 792493 793371 794251 795130 796010 796891
797772 798654 799536 800419 801302 802185 803069 803954 804839 805725 806611
807497 808384 809272 810160 811048 811937 812827 813717 814607 815498 816389
817281 818174 819067 819960 820854 821748 822643 823539 824434 825331 826228
827125 828023 828921 829820 830719 831619 832519 833420 834321 835223 836125

837028 837931 838835 839739 840644 841549 842454 843361 844267 845174 846082
846990 847899 848808 849717 850627 851538 852449 853360 854272 855185 856098
857012 857925 858840 859755 860670 861586 862503 863420 864337 865255 866173
867092 868012 868932 869852 870773 871694 872616 873538 874461 875384 876308
877232 878157 879083 880008 880935 881861 882788 883716 884644 885573 886502
887432 888362 889293 890224 891156 892088 893020 893953 894887 895821 896756
897691 898626 899562 900499 901436 902373 903311 904250 905189 906128 907068
908009 908950 909891 910833 911776 912719 913662 914606 915550 916495 917441
918386 919333 920280 921227 922175 923123 924072 925021 925971 926921 927872
928823 929775 930727 931680 932633 933587 934541 935496 936451 937407 938363
939319 940277 941234 942192 943151 944110 945070 946030 946990 947951 948913
949875 950837 951800 952764 953728 954693 955657 956623 957589 958555 959522
960490 961458 962426 963395 964364 965334 966305 967276 968247 969219 970191
971164 972137 973111 974085 975060 976036 977011 977988 978964 979942 980919
981898 982876 983856 984835 985815 986796 987777 988759 989741 990724 991707
992691 993675 994659 995644 996630 997616 998603 999590 1000577 1001565 1002554
1003543 1004532 1005522 1006513 1007504 1008495 1009487 1010480 1011473 1012466
1013460 1014455 1015449 1016445 1017441 1018437 1019434 1020431 1021429 1022428
1023426 1024426 1025426 1026426 1027427 1028428 1029430 1030432 1031435 1032438
1033442 1034446 1035451 1036456 1037462 1038468 1039475 1040482 1041489 1042498
1043506 1044515 1045525 1046535 1047546 1048557 1049569 1050581 1051593 1052606
1053620 1054634 1055648 1056663 1057679 1058695 1059712 1060729 1061746 1062764
1063782 1064801 1065821 1066841 1067861 1068882 1069904 1070925 1071948 1072971
1073994 1075018 1076042 1077067 1078093 1079118 1080145 1081172 1082199 1083227
1084255 1085284 1086313 1087343 1088373 1089404 1090435 1091467 1092499 1093532
1094565 1095598 1096633 1097667 1098702 1099738 1100774 1101811 1102848 1103886
1104924 1105962 1107001 1108041 1109081 1110122 1111163 1112204 1113246 1114289
1115332 1116375 1117419 1118464 1119509 1120554 1121600 1122647 1123693 1124741
1125789 1126837 1127886 1128936 1129985 1131036 1132087 1133138 1134190 1135242
1136295 1137348 1138402 1139456 1140511 1141567 1142622 1143679 1144735 1145793
1146850 1147909 1148967 1150026 1151086 1152146 1153207 1154268 1155330 1156392
1157455 1158518 1159581 1160646 1161710 1162775 1163841 1164907 1165974 1167041
1168108 1169176 1170245 1171314 1172383 1173453 1174524 1175595 1176666 1177738
1178811 1179884 1180957 1182031 1183106 1184180 1185256 1186332 1187408 1188485
1189562 1190640 1191719 1192798 1193877 1194957 1196037 1197118 1198199 1199281
1200363 1201446 1202529 1203613 1204697 1205782 1206867 1207953 1209039 1210126
1211213 1212301 1213389 1214478 1215567 1216656 1217747 1218837 1219928 1221020
1222112 1223205 1224298 1225391 1226485 1227580 1228675 1229771 1230867 1231963
1233060 1234158 1235256 1236354 1237453 1238553 1239653 1240753 1241854 1242955
1244057 1245160 1246263 1247366 1248470 1249574 1250679 1251785 1252891 1253997
1255104 1256211 1257319 1258427 1259536 1260645 1261755 1262865 1263976 1265087
1266199 1267311 1268424 1269537 1270651 1271765 1272880 1273995 1275111 1276227
1277344 1278461 1279579 1280697 1281815 1282934 1284054 1285174 1286295 1287416
1288537 1289659 1290782 1291905 1293029 1294153 1295277 1296402 1297528 1298654
1299780 1300907 1302035 1303163 1304291 1305420 1306549 1307679 1308810 1309941
1311072 1312204 1313336 1314469 1315603 1316736 1317871 1319006 1320141 1321277
1322413 1323550 1324687 1325825 1326963 1328102 1329241 1330381 1331521 1332662
1333803 1334945 1336087 1337230 1338373 1339517 1340661 1341806 1342951 1344096

1345243 1346389 1347536 1348684 1349832 1350981 1352130 1353279 1354429 1355580
1356731 1357883 1359035 1360187 1361340 1362494 1363648 1364802 1365957 1367113
1368269 1369425 1370582 1371739 1372897 1374056 1375215 1376374 1377534 1378694
1379855 1381017 1382178 1383341 1384504 1385667 1386831 1387995 1389160 1390325
1391491 1392657 1393824 1394991 1396159 1397327 1398496 1399665 1400835 1402005
1403176 1404347 1405519 1406691 1407864 1409037 1410211 1411385 1412559 1413735
1414910 1416086 1417263 1418440 1419618 1420796 1421974 1423153 1424333 1425513
1426693 1427874 1429056 1430238 1431420 1432603 1433787 1434971 1436155 1437340
1438526 1439712 1440898 1442085 1443272 1444460 1445649 1446838 1448027 1449217
1450407 1451598 1452789 1453981 1455174 1456366 1457560 1458754 1459948 1461143
1462338 1463534 1464730 1465927 1467124 1468322 1469520 1470719 1471918 1473118
1474318 1475518 1476720 1477921 1479123 1480326 1481529 1482733 1483937 1485142
1486347 1487552 1488758 1489965 1491172 1492380 1493588 1494796 1496005 1497215
1498425 1499635 1500846 1502058 1503270 1504482 1505695 1506909 1508123 1509337
1510552 1511768 1512983 1514200 1515417 1516634 1517852 1519070 1520289 1521509
1522729 1523949 1525170 1526391 1527613 1528835 1530058 1531281 1532505 1533729
1534954 1536179 1537405 1538631 1539858 1541085 1542313 1543541 1544770 1545999
1547229 1548459 1549690 1550921 1552152 1553385 1554617 1555850 1557084 1558318
1559553 1560788 1562023 1563259 1564496 1565733 1566971 1568209 1569447 1570686
1571926 1573166 1574406 1575647 1576889 1578131 1579373 1580616 1581860 1583104
1584348 1585593 1586838 1588084 1589331 1590578 1591825 1593073 1594321 1595570
1596819 1598069 1599320 1600570 1601822 1603073 1604326 1605579 1606832 1608086
1609340 1610595 1611850 1613106 1614362 1615619 1616876 1618134 1619392 1620650
1621910 1623169 1624429 1625690 1626951 1628213 1629475 1630738 1632001 1633264
1634528 1635793 1637058 1638324 1639590 1640856 1642123 1643391 1644659 1645928
1647197 1648466 1649736 1651007 1652278 1653549 1654821 1656093 1657366 1658640
1659914 1661188 1662463 1663739 1665014 1666291 1667568 1668845 1670123 1671401
1672680 1673959 1675239 1676520 1677800 1679082 1680364 1681646 1682929 1684212
1685496 1686780 1688065 1689350 1690636 1691922 1693209 1694496 1695784 1697072
1698360 1699650 1700939 1702229 1703520 1704811 1706103 1707395 1708688 1709981
1711274 1712568 1713863 1715158 1716454 1717750 1719046 1720343 1721641 1722939
1724237 1725537 1726836 1728136 1729437 1730737 1732039 1733341 1734643 1735946
1737250 1738554 1739858 1741163 1742468 1743774 1745081 1746388 1747695 1749003
1750311 1751620 1752929 1754239 1755550 1756860 1758172 1759484 1760796 1762109
1763422 1764736 1766050 1767365 1768680 1769996 1771312 1772629 1773946 1775264
1776582 1777900 1779220 1780539 1781859 1783180 1784501 1785823 1787145 1788468
1789791 1791114 1792438 1793763 1795088 1796414 1797740 1799066 1800393 1801721
1803049 1804377 1805707 1807036 1808366 1809697 1811028 1812359 1813691 1815023
1816356 1817690 1819024 1820358 1821693 1823029 1824364 1825701 1827038 1828375
1829713 1831051 1832390 1833730 1835069 1836410 1837751 1839092 1840434 1841776
1843119 1844462 1845806 1847150 1848495 1849840 1851186 1852532 1853879 1855226
1856574 1857922 1859271 1860620 1861970 1863320 1864670 1866021 1867373 1868725
1870078 1871431 1872785 1874139 1875493 1876848 1878204 1879560 1880917 1882274
1883631 1884989 1886348 1887707 1889066 1890426 1891787 1893148 1894509 1895871
1897234 1898597 1899960 1901324 1902689 1904054 1905419 1906785 1908151 1909518
1910886 1912254 1913622 1914991 1916360 1917730 1919100 1920471 1921842 1923214
1924587 1925959 1927333 1928707 1930081 1931456 1932831 1934207 1935583 1936960
1938337 1939715 1941093 1942471 1943851 1945230 1946611 1947991 1949372 1950754

1952136 1953519 1954902 1956286 1957670 1959054 1960439 1961825 1963211 1964598
1965985 1967372 1968760 1970149 1971538 1972928 1974318 1975708 1977099 1978491
1979883 1981275 1982668 1984062 1985455 1986850 1988245 1989640 1991036 1992433
1993830 1995227 1996625 1998023 1999422 2000822 2002221 2003622 2005023 2006424
2007826 2009228 2010631 2012034 2013438 2014842 2016247 2017652 2019058 2020464
2021871 2023278 2024686 2026094 2027503 2028912 2030322 2031732 2033143 2034554
2035966 2037378 2038791 2040204 2041617 2043032 2044446 2045861 2047277 2048693
2050110 2051527 2052944 2054362 2055781 2057200 2058620 2060040 2061460 2062881
2064303 2065725 2067147 2068570 2069994 2071418 2072842 2074267 2075693 2077119
2078545 2079972 2081399 2082827 2084256 2085685 2087114 2088544 2089974 2091405
2092837 2094268 2095701 2097134 2098567 2100001 2101435 2102870 2104305 2105741
2107177 2108614 2110051 2111489 2112927 2114366 2115805 2117245 2118685 2120126
2121567 2123009 2124451 2125894 2127337 2128781 2130225 2131669 2133115 2134560
2136006 2137453 2138900 2140348 2141796 2143244 2144694 2146143 2147593 2149044
2150495 2151946 2153398 2154851 2156304 2157757 2159211 2160666 2162121 2163576
2165032 2166489 2167946 2169403 2170861 2172320 2173779 2175238 2176698 2178158
2179619 2181081 2182543 2184005 2185468 2186931 2188395 2189859 2191324 2192790
2194255 2195722 2197188 2198656 2200124 2201592 2203061 2204530 2206000 2207470
2208941 2210412 2211884 2213356 2214829 2216302 2217775 2219250 2220724 2222199
2223675 2225151 2226628 2228105 2229583 2231061 2232539 2234019 2235498 2236978
2238459 2239940 2241422 2242904 2244386 2245869 2247353 2248837 2250321 2251806
2253292 2254778 2256264 2257751 2259239 2260727 2262215 2263704 2265194 2266684
2268174 2269665 2271156 2272648 2274141 2275634 2277127 2278621 2280115 2281610
2283106 2284601 2286098 2287595 2289092 2290590 2292088 2293587 2295086 2296586
2298086 2299587 2301088 2302590 2304092 2305595 2307098 2308602 2310106 2311611
2313116 2314622 2316128 2317635 2319142 2320650 2322158 2323666 2325176 2326685
2328195 2329706 2331217 2332729 2334241 2335753 2337267 2338780 2340294 2341809
2343324 2344839 2346355 2347872 2349389 2350906 2352424 2353943 2355462 2356981
2358501 2360022 2361543 2363064 2364586 2366109 2367632 2369155 2370679 2372203
2373728 2375254 2376780 2378306 2379833 2381360 2382888 2384416 2385945 2387475
2389004 2390535 2392066 2393597 2395129 2396661 2398194 2399727 2401261 2402795
2404330 2405865 2407401 2408937 2410474 2412011 2413549 2415087 2416626 2418165
2419704 2421245 2422785 2424326 2425868 2427410 2428953 2430496 2432040 2433584
2435128 2436673 2438219 2439765 2441312 2442859 2444406 2445954 2447503 2449052
2450601 2452151 2453702 2455253 2456805 2458356 2459909 2461462 2463015 2464569
2466124 2467679 2469234 2470790 2472347 2473903 2475461 2477019 2478577 2480136
2481695 2483255 2484816 2486377 2487938 2489500 2491062 2492625 2494188 2495752
2497316 2498881 2500446 2502012 2503578 2505145 2506712 2508280 2509848 2511417
2512986 2514556 2516126 2517697 2519268 2520840 2522412 2523985 2525558 2527132
2528706 2530280 2531856 2533431 2535007 2536584 2538161 2539739 2541317 2542895
2544474 2546054 2547634 2549215 2550796 2552377 2553959 2555542 2557125 2558708
2560292 2561877 2563462 2565047 2566633 2568220 2569807 2571394 2572982 2574571
2576160 2577749 2579339 2580929 2582520 2584112 2585703 2587296 2588889 2590482
2592076 2593670 2595265 2596860 2598456 2600053 2601649 2603247 2604845 2606443
2608042 2609641 2611241 2612841 2614442 2616043 2617645 2619247 2620850 2622453
2624057 2625661 2627265 2628871 2630476 2632082 2633689 2635296 2636904 2638512
2640121 2641730 2643339 2644950 2646560 2648171 2649783 2651395 2653008 2654621
2656234 2657848 2659463 2661078 2662693 2664309 2665926 2667543 2669160 2670778

2672397 2674016 2675635 2677255 2678876 2680497 2682118 2683740 2685363 2686985
2688609 2690233 2691857 2693482 2695107 2696733 2698360 2699987 2701614 2703242
2704870 2706499 2708128 2709758 2711388 2713019 2714651 2716282 2717915 2719547
2721181 2722814 2724449 2726083 2727719 2729354 2730991 2732627 2734265 2735902
2737540 2739179 2740818 2742458 2744098 2745739 2747380 2749022 2750664 2752306
2753949 2755593 2757237 2758882 2760527 2762172 2763819 2765465 2767112 2768760
2770408 2772056 2773705 2775355 2777005 2778655 2780306 2781958 2783610 2785262
2786915 2788569 2790223 2791877 2793532 2795188 2796844 2798500 2800157 2801814
2803472 2805131 2806790 2808449 2810109 2811769 2813430 2815091 2816753 2818416
2820078 2821742 2823406 2825070 2826735 2828400 2830066 2831732 2833399 2835066
2836734 2838402 2840071 2841740 2843410 2845080 2846751 2848422 2850094 2851766
2853439 2855112 2856785 2858460 2860134 2861809 2863485 2865161 2866838 2868515
2870192 2871871 2873549 2875228 2876908 2878588 2880269 2881950 2883631 2885313
2886996 2888679 2890362 2892046 2893731 2895416 2897101 2898787 2900474 2902161
2903848 2905536 2907225 2908914 2910603 2912293 2913983 2915674 2917366 2919058
2920750 2922443 2924136 2925830 2927525 2929219 2930915 2932611 2934307 2936004
2937701 2939399 2941097 2942796 2944495 2946195 2947895 2949596 2951298 2952999
2954702 2956404 2958108 2959811 2961515 2963220 2964925 2966631 2968337 2970044
2971751 2973459 2975167 2976876 2978585 2980295 2982005 2983715 2985426 2987138
2988850 2990563 2992276 2993989 2995704 2997418 2999133 3000849 3002565 3004281
3005998 3007716 3009434 3011152 3012871 3014591 3016311 3018031 3019752 3021474
3023196 3024918 3026641 3028365 3030089 3031813 3033538 3035263 3036989 3038716
3040443 3042170 3043898 3045626 3047355 3049085 3050814 3052545 3054276 3056007
3057739 3059471 3061204 3062937 3064671 3066405 3068140 3069875 3071611 3073347
3075084 3076821 3078559 3080297 3082036 3083775 3085515 3087255 3088996 3090737
3092479 3094221 3095964 3097707 3099450 3101195 3102939 3104684 3106430 3108176
3109923 3111670 3113417 3115166 3116914 3118663 3120413 3122163 3123913 3125664
3127416 3129168 3130921 3132674 3134427 3136181 3137936 3139691 3141446 3143202
3144959 3146716 3148473 3150231 3151989 3153748 3155508 3157268 3159028 3160789
3162550 3164312 3166075 3167838 3169601 3171365 3173129 3174894 3176659 3178425
3180192 3181958 3183726 3185493 3187262 3189031 3190800 3192570 3194340 3196111
3197882 3199654 3201426 3203199 3204972 3206746 3208520 3210295 3212070 3213846
3215622 3217398 3219176 3220953 3222731 3224510 3226289 3228069 3229849 3231630
3233411 3235192 3236974 3238757 3240540 3242324 3244108 3245892 3247677 3249463
3251249 3253036 3254823 3256610 3258398 3260187 3261976 3263765 3265555 3267346
3269137 3270928 3272720 3274513 3276306 3278099 3279893 3281687 3283482 3285278
3287074 3288870 3290667 3292464 3294262 3296061 3297859 3299659 3301459 3303259
3305060 3306861 3308663 3310465 3312268 3314071 3315875 3317680 3319484 3321290
3323095 3324902 3326708 3328516 3330323 3332132 3333940 3335750 3337559 3339369
3341180 3342991 3344803 3346615 3348428 3350241 3352055 3353869 3355684 3357499
3359314 3361130 3362947 3364764 3366582 3368400 3370218 3372038 3373857 3375677
3377498 3379319 3381140 3382962 3384785 3386608 3388432 3390256 3392080 3393905
3395731 3397557 3399383 3401210 3403037 3404865 3406694 3408523 3410352 3412182
3414013 3415843 3417675 3419507 3421339 3423172 3425005 3426839 3428674 3430509
3432344 3434180 3436016 3437853 3439690 3441528 3443366 3445205 3447045 3448884
3450725 3452565 3454407 3456248 3458091 3459933 3461777 3463620 3465465 3467309
3469155 3471000 3472847 3474693 3476540 3478388 3480236 3482085 3483934 3485784
3487634 3489485 3491336 3493187 3495039 3496892 3498745 3500599 3502453 3504307

3506163 3508018 3509874 3511731 3513588 3515445 3517303 3519162 3521021 3522880
3524740 3526601 3528462 3530323 3532185 3534048 3535911 3537774 3539638 3541503
3543368 3545233 3547099 3548965 3550832 3552700 3554568 3556436 3558305 3560174
3562044 3563915 3565785 3567657 3569529 3571401 3573274 3575147 3577021 3578895
3580770 3582645 3584521 3586397 3588274 3590152 3592029 3593908 3595786 3597666
3599545 3601426 3603306 3605187 3607069 3608951 3610834 3612717 3614601 3616485
3618370 3620255 3622141 3624027 3625913 3627801 3629688 3631576 3633465 3635354
3637244 3639134 3641024 3642915 3644807 3646699 3648592 3650485 3652378 3654272
3656167 3658062 3659957 3661853 3663750 3665647 3667544 3669442 3671341 3673240
3675139 3677039 3678940 3680841 3682742 3684644 3686546 3688449 3690353 3692257
3694161 3696066 3697971 3699877 3701784 3703690 3705598 3707506 3709414 3711323
3713232 3715142 3717052 3718963 3720874 3722786 3724698 3726611 3728524 3730438
3732353 3734267 3736183 3738098 3740014 3741931 3743848 3745766 3747684 3749603
3751522 3753442 3755362 3757283 3759204 3761126 3763048 3764970 3766894 3768817
3770741 3772666 3774591 3776517 3778443 3780369 3782296 3784224 3786152 3788081
3790010 3791939 3793869 3795800 3797731 3799662 3801594 3803527 3805460 3807393
3809327 3811262 3813197 3815132 3817068 3819005 3820942 3822879 3824817 3826756
3828695 3830634 3832574 3834514 3836455 3838397 3840338 3842281 3844224 3846167
3848111 3850055 3852000 3853945 3855891 3857838 3859784 3861732 3863680 3865628
3867577 3869526 3871476 3873426 3875377 3877328 3879280 3881232 3883185 3885138
3887092 3889046 3891001 3892956 3894911 3896868 3898824 3900782 3902739 3904697
3906656 3908615 3910575 3912535 3914496 3916457 3918418 3920380 3922343 3924306
3926270 3928234 3930198 3932163 3934129 3936095 3938062 3940029 3941996 3943964
3945933 3947902 3949871 3951841 3953812 3955783 3957754 3959726 3961699 3963672
3965645 3967619 3969593 3971568 3973544 3975520 3977496 3979473 3981451 3983428
3985407 3987386 3989365 3991345 3993325 3995306 3997287 3999269 4001252 4003235
4005218 4007202 4009186 4011171 4013156 4015142 4017128 4019115 4021102 4023090
4025078 4027067 4029056 4031046 4033036 4035027 4037018 4039010 4041002 4042995
4044988 4046982 4048976 4050970 4052966 4054961 4056957 4058954 4060951 4062949
4064947 4066946 4068945 4070944 4072944 4074945 4076946 4078948 4080950 4082952
4084955 4086959 4088963 4090967 4092972 4094978 4096984 4098991 4100998 4103005
4105013 4107022 4109031 4111040 4113050 4115060 4117071 4119083 4121095 4123107
4125120 4127134 4129147 4131162 4133177 4135192 4137208 4139224 4141241 4143259
4145276 4147295 4149314 4151333 4153353 4155373 4157394 4159415 4161437 4163459
4165482 4167506 4169529 4171554 4173578 4175604 4177629 4179656 4181682 4183710
4185737 4187766 4189794 4191823 4193853 4195883 4197914 4199945 4201977 4204009
4206042 4208075 4210109 4212143 4214177 4216212 4218248 4220284 4222321 4224358
4226395 4228434 4230472 4232511 4234551 4236591 4238631 4240672 4242714 4244756
4246799 4248842 4250885 4252929 4254974 4257019 4259064 4261110 4263157 4265204
4267251 4269299 4271347 4273396 4275446 4277496 4279546 4281597 4283649 4285700
4287753 4289806 4291859 4293913 4295967 4298022 4300078 4302133 4304190 4306247
4308304 4310362 4312420 4314479 4316538 4318598 4320658 4322719 4324780 4326842
4328904 4330967 4333031 4335094 4337159 4339223 4341288 4343354 4345420 4347487
4349554 4351622 4353690 4355759 4357828 4359898 4361968 4364039 4366110 4368181
4370254 4372326 4374399 4376473 4378547 4380622 4382697 4384772 4386848 4388925
4391002 4393080 4395158 4397236 4399315 4401395 4403475 4405555 4407636 4409718
4411800 4413882 4415965 4418049 4420133 4422217 4424302 4426388 4428474 4430560
4432647 4434735 4436823 4438911 4441000 4443089 4445179 4447270 4449361 4451452

4453544 4455636 4457729 4459822 4461916 4464011 4466105 4468201 4470297 4472393
4474490 4476587 4478685 4480783 4482882 4484981 4487081 4489181 4491282 4493383
4495485 4497587 4499690 4501793 4503897 4506001 4508106 4510211 4512317 4514423
4516530 4518637 4520745 4522853 4524961 4527071 4529180 4531290 4533401 4535512
4537624 4539736 4541848 4543962 4546075 4548189 4550304 4552419 4554534 4556650
4558767 4560884 4563002 4565120 4567238 4569357 4571477 4573597 4575717 4577838
4579960 4582082 4584204 4586327 4588451 4590575 4592699 4594824 4596949 4599075
4601202 4603329 4605456 4607584 4609712 4611841 4613971 4616101 4618231 4620362
4622493 4624625 4626757 4628890 4631024 4633158 4635292 4637427 4639562 4641698
4643834 4645971 4648108 4650246 4652384 4654523 4656662 4658802 4660942 4663083
4665224 4667366 4669508 4671651 4673794 4675938 4678082 4680227 4682372 4684518
4686664 4688810 4690958 4693105 4695253 4697402 4699551 4701701 4703851 4706001
4708153 4710304 4712456 4714609 4716762 4718916 4721070 4723224 4725379 4727535
4729691 4731847 4734004 4736162 4738320 4740478 4742637 4744797 4746957 4749117
4751278 4753440 4755602 4757764 4759927 4762091 4764255 4766419 4768584 4770749
4772915 4775082 4777249 4779416 4781584 4783752 4785921 4788091 4790261 4792431
4794602 4796773 4798945 4801117 4803290 4805463 4807637 4809812 4811986 4814162
4816337 4818514 4820690 4822868 4825045 4827224 4829403 4831582 4833761 4835942
4838122 4840304 4842485 4844668 4846850 4849033 4851217 4853401 4855586 4857771
4859957 4862143 4864330 4866517 4868704 4870893 4873081 4875270 4877460 4879650
4881841 4884032 4886223 4888415 4890608 4892801 4894995 4897189 4899383 4901578
4903774 4905970 4908166 4910363 4912561 4914759 4916957 4919156 4921356 4923556
4925756 4927957 4930159 4932361 4934563 4936766 4938970 4941173 4943378 4945583
4947788 4949994 4952201 4954407 4956615 4958823 4961031 4963240 4965449 4967659
4969869 4972080 4974292 4976503 4978716 4980929 4983142 4985356 4987570 4989785
4992000 4994216 4996432 4998649 5000866 5003084 5005302 5007521 5009740 5011960
5014180 5016401 5018622 5020844 5023066 5025289 5027512 5029735 5031960 5034184
5036409 5038635 5040861 5043088 5045315 5047543 5049771 5051999 5054228 5056458
5058688 5060919 5063150 5065381 5067614 5069846 5072079 5074313 5076547 5078781
5081016 5083252 5085488 5087724 5089961 5092199 5094437 5096675 5098914 5101154
5103394 5105634 5107875 5110117 5112359 5114601 5116844 5119087 5121331 5123576
5125821 5128066 5130312 5132558 5134805 5137053 5139300 5141549 5143798 5146047
5148297 5150547 5152798 5155049 5157301 5159554 5161806 5164060 5166313 5168568
5170822 5173078 5175334 5177590 5179847 5182104 5184362 5186620 5188879 5191138
5193397 5195658 5197918 5200180 5202441 5204703 5206966 5209229 5211493 5213757
5216022 5218287 5220553 5222819 5225085 5227353 5229620 5231888 5234157 5236426
5238696 5240966 5243236 5245507 5247779 5250051 5252324 5254597 5256870 5259144
5261419 5263694 5265969 5268245 5270522 5272799 5275076 5277354 5279633 5281912
5284191 5286471 5288752 5291033 5293314 5295596 5297878 5300161 5302445 5304729
5307013 5309298 5311583 5313869 5316156 5318443 5320730 5323018 5325306 5327595
5329884 5332174 5334465 5336755 5339047 5341338 5343631 5345924 5348217 5350511
5352805 5355100 5357395 5359691 5361987 5364284 5366581 5368879 5371177 5373476
5375775 5378075 5380375 5382676 5384977 5387279 5389581 5391883 5394187 5396490
5398794 5401099 5403404 5405710 5408016 5410323 5412630 5414937 5417245 5419554
5421863 5424173 5426483 5428793 5431105 5433416 5435728 5438041 5440354 5442667
5444981 5447296 5449611 5451926 5454242 5456559 5458876 5461193 5463511 5465830
5468149 5470468 5472788 5475109 5477430 5479751 5482073 5484396 5486718 5489042
5491366 5493690 5496015 5498341 5500666 5502993 5505320 5507647 5509975 5512303

5514632 5516961 5519291 5521622 5523952 5526284 5528616 5530948 5533281 5535614
5537948 5540282 5542617 5544952 5547288 5549624 5551961 5554298 5556636 5558974
5561313 5563652 5565992 5568332 5570673 5573014 5575356 5577698 5580041 5582384
5584727 5587072 5589416 5591761 5594107 5596453 5598800 5601147 5603495 5605843
5608191 5610540 5612890 5615240 5617591 5619942 5622293 5624645 5626998 5629351
5631705 5634059 5636413 5638768 5641124 5643480 5645836 5648193 5650551 5652909
5655267 5657626 5659986 5662346 5664706 5667067 5669428 5671790 5674153 5676516
5678879 5681243 5683608 5685972 5688338 5690704 5693070 5695437 5697804 5700172
5702541 5704910 5707279 5709649 5712019 5714390 5716761 5719133 5721505 5723878
5726251 5728625 5731000 5733374 5735750 5738125 5740502 5742878 5745256 5747633
5750012 5752390 5754770 5757149 5759530 5761910 5764292 5766673 5769055 5771438
5773821 5776205 5778589 5780974 5783359 5785745 5788131 5790517 5792905 5795292
5797680 5800069 5802458 5804848 5807238 5809628 5812019 5814411 5816803 5819196
5821589 5823982 5826376 5828771 5831166 5833561 5835957 5838354 5840751 5843148
5845546 5847945 5850344 5852743 5855143 5857544 5859945 5862346 5864748 5867151
5869554 5871957 5874361 5876765 5879170 5881576 5883982 5886388 5888795 5891202
5893610 5896019 5898427 5900837 5903247 5905657 5908068 5910479 5912891 5915303
5917716 5920129 5922543 5924958 5927372 5929788 5932203 5934620 5937036 5939454
5941872 5944290 5946709 5949128 5951547 5953968 5956388 5958810 5961231 5963654
5966076 5968499 5970923 5973347 5975772 5978197 5980623 5983049 5985476 5987903
5990330 5992759 5995187 5997616 6000046 6002476 6004907 6007338 6009769 6012201
6014634 6017067 6019501 6021935 6024369 6026804 6029240 6031676 6034112 6036549
6038987 6041425 6043863 6046302 6048742 6051182 6053622 6056063 6058505 6060947
6063389 6065832 6068276 6070720 6073164 6075609 6078054 6080500 6082947 6085394
6087841 6090289 6092737 6095186 6097636 6100085 6102536 6104987 6107438 6109890
6112342 6114795 6117248 6119702 6122157 6124611 6127067 6129522 6131979 6134436
6136893 6139351 6141809 6144268 6146727 6149187 6151647 6154108 6156569 6159031
6161493 6163955 6166419 6168882 6171347 6173811 6176276 6178742 6181208 6183675
6186142 6188610 6191078 6193547 6196016 6198485 6200955 6203426 6205897 6208369
6210841 6213313 6215787 6218260 6220734 6223209 6225684 6228159 6230635 6233112
6235589 6238067 6240545 6243023 6245502 6247982 6250462 6252942 6255423 6257905
6260387 6262869 6265352 6267836 6270319 6272804 6275289 6277774 6280260 6282747
6285234 6287721 6290209 6292697 6295186 6297676 6300165 6302656 6305147 6307638
6310130 6312622 6315115 6317609 6320102 6322597 6325092 6327587 6330083 6332579
6335076 6337573 6340071 6342569 6345068 6347567 6350067 6352567 6355068 6357569
6360071 6362573 6365076 6367579 6370083 6372587 6375092 6377597 6380103 6382609
6385116 6387623 6390130 6392639 6395147 6397656 6400166 6402676 6405187 6407698
6410210 6412722 6415234 6417747 6420261 6422775 6425290 6427805 6430320 6432836
6435353 6437870 6440388 6442906 6445424 6447943 6450463 6452983 6455503 6458024
6460546 6463068 6465590 6468113 6470637 6473161 6475685 6478210 6480736 6483262
6485788 6488315 6490842 6493370 6495899 6498428 6500957 6503487 6506017 6508548
6511080 6513612 6516144 6518677 6521210 6523744 6526278 6528813 6531349 6533884
6536421 6538958 6541495 6544033 6546571 6549110 6551649 6554189 6556729 6559270
6561811 6564353 6566895 6569438 6571981 6574525 6577069 6579614 6582159 6584705
6587251 6589798 6592345 6594893 6597441 6599990 6602539 6605088 6607639 6610189
6612740 6615292 6617844 6620397 6622950 6625504 6628058 6630612 6633167 6635723
6638279 6640836 6643393 6645950 6648508 6651067 6653626 6656186 6658746 6661306
6663867 6666429 6668991 6671553 6674116 6676680 6679244 6681808 6684373 6686939

6689505 6692071 6694638 6697206 6699774 6702342 6704911 6707480 6710050 6712621
6715192 6717763 6720335 6722907 6725480 6728054 6730628 6733202 6735777 6738352
6740928 6743504 6746081 6748659 6751237 6753815 6756394 6758973 6761553 6764133
6766714 6769295 6771877 6774459 6777042 6779625 6782209 6784793 6787378 6789963
6792549 6795135 6797722 6800309 6802897 6805485 6808074 6810663 6813253 6815843
6818434 6821025 6823617 6826209 6828801 6831395 6833988 6836582 6839177 6841772
6844368 6846964 6849560 6852158 6854755 6857353 6859952 6862551 6865151 6867751
6870351 6872952 6875554 6878156 6880758 6883361 6885965 6888569 6891174 6893779
6896384 6898990 6901597 6904204 6906811 6909419 6912028 6914636 6917246 6919856
6922466 6925077 6927689 6930301 6932913 6935526 6938139 6940753 6943368 6945983
6948598 6951214 6953830 6956447 6959065 6961682 6964301 6966920 6969539 6972159
6974779 6977400 6980021 6982643 6985265 6987888 6990512 6993135 6995760 6998384
7001010 7003636 7006262 7008889 7011516 7014144 7016772 7019401 7022030 7024660
7027290 7029920 7032552 7035183 7037816 7040448 7043081 7045715 7048349 7050984
7053619 7056255 7058891 7061528 7064165 7066802 7069440 7072079 7074718 7077358
7079998 7082639 7085280 7087921 7090563 7093206 7095849 7098493 7101137 7103781
7106426 7109072 7111718 7114364 7117011 7119659 7122307 7124955 7127604 7130254
7132904 7135554 7138205 7140857 7143509 7146161 7148814 7151468 7154122 7156776
7159431 7162086 7164742 7167399 7170056 7172713 7175371 7178029 7180688 7183348
7186007 7188668 7191329 7193990 7196652 7199314 7201977 7204640 7207304 7209969
7212633 7215299 7217964 7220631 7223298 7225965 7228633 7231301 7233970 7236639
7239309 7241979 7244650 7247321 7249993 7252665 7255338 7258011 7260685 7263359
7266034 7268709 7271384 7274061 7276737 7279414 7282092 7284770 7287449 7290128
7292808 7295488 7298168 7300850 7303531 7306213 7308896 7311579 7314263 7316947
7319631 7322316 7325002 7327688 7330375 7333062 7335749 7338437 7341126 7343815
7346504 7349194 7351885 7354576 7357267 7359959 7362652 7365345 7368038 7370732
7373427 7376122 7378817 7381513 7384210 7386907 7389604 7392302 7395000 7397699
7400399 7403099 7405799 7408500 7411201 7413903 7416606 7419309 7422012 7424716
7427420 7430125 7432830 7435536 7438243 7440949 7443657 7446365 7449073 7451782
7454491 7457201 7459911 7462622 7465333 7468045 7470757 7473470 7476183 7478897
7481611 7484326 7487041 7489757 7492473 7495190 7497907 7500625 7503343 7506062
7508781 7511501 7514221 7516941 7519663 7522384 7525106 7527829 7530552 7533276
7536000 7538725 7541450 7544175 7546901 7549628 7552355 7555083 7557811 7560539
7563268 7565998 7568728 7571459 7574190 7576921 7579653 7582386 7585119 7587852
7590586 7593321 7596056 7598791 7601527 7604264 7607001 7609738 7612476 7615215
7617954 7620693 7623433 7626174 7628915 7631656 7634398 7637140 7639883 7642627
7645371 7648115 7650860 7653605 7656351 7659097 7661844 7664592 7667340 7670088
7672837 7675586 7678336 7681086 7683837 7686588 7689340 7692093 7694845 7697599
7700352 7703107 7705861 7708617 7711373 7714129 7716886 7719643 7722401 7725159
7727918 7730677 7733436 7736197 7738957 7741719 7744480 7747243 7750005 7752768
7755532 7758296 7761061 7763826 7766592 7769358 7772125 7774892 7777659 7780427
7783196 7785965 7788735 7791505 7794275 7797047 7799818 7802590 7805363 7808136
7810909 7813684 7816458 7819233 7822009 7824785 7827561 7830338 7833116 7835894
7838672 7841451 7844231 7847011 7849791 7852572 7855354 7858136 7860918 7863701
7866485 7869269 7872053 7874838 7877623 7880409 7883196 7885983 7888770 7891558
7894346 7897135 7899925 7902714 7905505 7908296 7911087 7913879 7916671 7919464
7922257 7925051 7927846 7930640 7933436 7936232 7939028 7941825 7944622 7947420
7950218 7953017 7955816 7958616 7961416 7964217 7967018 7969820 7972622 7975425

7978228 7981032 7983836 7986641 7989446 7992252 7995058 7997865 8000672 8003479
8006288 8009096 8011905 8014715 8017525 8020336 8023147 8025959 8028771 8031583
8034397 8037210 8040024 8042839 8045654 8048470 8051286 8054102 8056919 8059737
8062555 8065373 8068193 8071012 8073832 8076653 8079474 8082295 8085117 8087940
8090763 8093586 8096410 8099235 8102060 8104885 8107711 8110538 8113365 8116192
8119020 8121848 8124677 8127507 8130337 8133167 8135998 8138829 8141661 8144494
8147327 8150160 8152994 8155828 8158663 8161499 8164334 8167171 8170008 8172845
8175683 8178521 8181360 8184199 8187039 8189879 8192720 8195561 8198403 8201246
8204088 8206932 8209775 8212620 8215464 8218310 8221155 8224002 8226848 8229696
8232543 8235392 8238240 8241090 8243939 8246789 8249640 8252491 8255343 8258195
8261048 8263901 8266755 8269609 8272463 8275319 8278174 8281030 8283887 8286744
8289602 8292460 8295318 8298178 8301037 8303897 8306758 8309619 8312480 8315343
8318205 8321068 8323932 8326796 8329660 8332525 8335391 8338257 8341123 8343990
8346858 8349726 8352594 8355463 8358333 8361203 8364073 8366944 8369816 8372688
8375560 8378433 8381306 8384180 8387055 8389930 8392805 8395681 8398558 8401434
8404312 8407190 8410068 8412947 8415826 8418706 8421587 8424467 8427349 8430231
8433113 8435996 8438879 8441763 8444647 8447532 8450417 8453303 8456189 8459076
8461964 8464851 8467740 8470628 8473518 8476407 8479298 8482188 8485080 8487971
8490864 8493756 8496649 8499543 8502437 8505332 8508227 8511123 8514019 8516916
8519813 8522711 8525609 8528508 8531407 8534306 8537206 8540107 8543008 8545910
8548812 8551715 8554618 8557521 8560425 8563330 8566235 8569141 8572047 8574953
8577860 8580768 8583676 8586584 8589494 8592403 8595313 8598224 8601135 8604046
8606958 8609871 8612784 8615697 8618611 8621526 8624441 8627356 8630272 8633188
8636105 8639023 8641941 8644859 8647778 8650698 8653618 8656538 8659459 8662380
8665302 8668225 8671147 8674071 8676995 8679919 8682844 8685769 8688695 8691621
8694548 8697476 8700404 8703332 8706261 8709190 8712120 8715050 8717981 8720912
8723844 8726776 8729709 8732642 8735576 8738510 8741445 8744380 8747316 8750252
8753189 8756126 8759064 8762002 8764941 8767880 8770820 8773760 8776701 8779642
8782584 8785526 8788468 8791412 8794355 8797299 8800244 8803189 8806135 8809081
8812028 8814975 8817922 8820870 8823819 8826768 8829718 8832668 8835618 8838569
8841521 8844473 8847426 8850379 8853332 8856286 8859241 8862196 8865151 8868107
8871064 8874021 8876978 8879936 8882895 8885854 8888813 8891773 8894734 8897695
8900656 8903618 8906581 8909544 8912507 8915471 8918435 8921400 8924366 8927332
8930298 8933265 8936232 8939200 8942169 8945137 8948107 8951077 8954047 8957018
8959989 8962961 8965933 8968906 8971880 8974853 8977828 8980803 8983778 8986754
8989730 8992707 8995684 8998662 9001640 9004619 9007598 9010578 9013558 9016539
9019520 9022502 9025484 9028467 9031450 9034434 9037418 9040403 9043388 9046374
9049360 9052346 9055334 9058321 9061310 9064298 9067287 9070277 9073267 9076258
9079249 9082241 9085233 9088225 9091218 9094212 9097206 9100201 9103196 9106191
9109187 9112184 9115181 9118179 9121177 9124175 9127174 9130174 9133174 9136174
9139175 9142177 9145179 9148181 9151184 9154188 9157192 9160196 9163201 9166207
9169213 9172219 9175226 9178234 9181242 9184250 9187259 9190269 9193278 9196289
9199300 9202311 9205323 9208336 9211348 9214362 9217376 9220390 9223405 9226420
9229436 9232453 9235469 9238487 9241505 9244523 9247542 9250561 9253581 9256601
9259622 9262643 9265665 9268687 9271710 9274733 9277757 9280782 9283806 9286832
9289857 9292884 9295910 9298938 9301965 9304993 9308022 9311051 9314081 9317111
9320142 9323173 9326205 9329237 9332270 9335303 9338337 9341371 9344405 9347441
9350476 9353512 9356549 9359586 9362624 9365662 9368701 9371740 9374779 9377819

9380860 9383901 9386943 9389985 9393027 9396070 9399114 9402158 9405202 9408248
9411293 9414339 9417386 9420433 9423480 9426528 9429577 9432626 9435675 9438725
9441776 9444827 9447878 9450930 9453983 9457036 9460089 9463143 9466197 9469252
9472308 9475364 9478420 9481477 9484534 9487592 9490651 9493710 9496769 9499829
9502889 9505950 9509012 9512073 9515136 9518199 9521262 9524326 9527390 9530455
9533520 9536586 9539652 9542719 9545786 9548854 9551923 9554991 9558061 9561130
9564201 9567271 9570343 9573414 9576487 9579559 9582633 9585706 9588781 9591855
9594931 9598006 9601083 9604159 9607236 9610314 9613392 9616471 9619550 9622630
9625710 9628791 9631872 9634953 9638036 9641118 9644201 9647285 9650369 9653454
9656539 9659624 9662711 9665797 9668884 9671972 9675060 9678148 9681238 9684327
9687417 9690508 9693599 9696690 9699782 9702875 9705968 9709061 9712155 9715250
9718345 9721440 9724536 9727633 9730730 9733827 9736925 9740024 9743123 9746222
9749322 9752423 9755524 9758625 9761727 9764829 9767932 9771036 9774140 9777244
9780349 9783454 9786560 9789667 9792773 9795881 9798989 9802097 9805206 9808315
9811425 9814535 9817646 9820758 9823869 9826982 9830095 9833208 9836322 9839436
9842551 9845666 9848782 9851898 9855015 9858132 9861250 9864368 9867487 9870606
9873726 9876846 9879967 9883088 9886210 9889332 9892455 9895578 9898702 9901826
9904950 9908076 9911201 9914327 9917454 9920581 9923709 9926837 9929966 9933095
9936224 9939355 9942485 9945616 9948748 9951880 9955013 9958146 9961279 9964413
9967548 9970683 9973819 9976955 9980091 9983228 9986366 9989504 9992642 9995781
9998921 10002061 10005202 10008343 10011484 10014626 10017769 10020912 10024055
10027199 10030343 10033488 10036634 10039780 10042926 10046073 10049221 10052369
10055517 10058666 10061815 10064965 10068116 10071267 10074418 10077570 10080722
10083875 10087029 10090182 10093337 10096492 10099647 10102803 10105959 10109116
10112273 10115431 10118590 10121748 10124908 10128067 10131228 10134389 10137550
10140712 10143874 10147037 10150200 10153364 10156528 10159693 10162858 10166024
10169190 10172357 10175524 10178692 10181860 10185029 10188198 10191368 10194538
10197708 10200880 10204051 10207223 10210396 10213569 10216743 10219917 10223092
10226267 10229443 10232619 10235795 10238972 10242150 10245328 10248507 10251686
10254865 10258046 10261226 10264407 10267589 10270771 10273953 10277137 10280320
10283504 10286689 10289874 10293059 10296245 10299432 10302619 10305806 10308994
10312183 10315372 10318561 10321751 10324942 10328133 10331324 10334516 10337709
10340902 10344095 10347289 10350483 10353678 10356874 10360070 10363266 10366463
10369660 10372858 10376057 10379256 10382455 10385655 10388855 10392056 10395258
10398459 10401662 10404865 10408068 10411272 10414476 10417681 10420886 10424092
10427298 10430505 10433713 10436920 10440129 10443337 10446547 10449756 10452967
10456178 10459389 10462601 10465813 10469026 10472239 10475453 10478667 10481881
10485097 10488312 10491529 10494745 10497963 10501180 10504398 10507617 10510836
10514056 10517276 10520497 10523718 10526940 10530162 10533384 10536607 10539831
10543055 10546280 10549505 10552731 10555957 10559183 10562410 10565638 10568866
10572095 10575324 10578553 10581783 10585014 10588245 10591477 10594709 10597941
10601174 10604408 10607642 10610876 10614111 10617347 10620583 10623819 10627056
10630294 10633532 10636770 10640009 10643249 10646489 10649729 10652970 10656211
10659453 10662696 10665939 10669182 10672426 10675670 10678915 10682161 10685407
10688653 10691900 10695147 10698395 10701643 10704892 10708142 10711392 10714642
10717893 10721144 10724396 10727648 10730901 10734154 10737408 10740663 10743917
10747173 10750428 10753685 10756941 10760199 10763457 10766715 10769974 10773233
10776493 10779753 10783014 10786275 10789537 10792799 10796061 10799325 10802588

10805853 10809117 10812382 10815648 10818914 10822181 10825448 10828716 10831984
10835253 10838522 10841791 10845062 10848332 10851603 10854875 10858147 10861420
10864693 10867966 10871241 10874515 10877790 10881066 10884342 10887618 10890896
10894173 10897451 10900730 10904009 10907288 10910568 10913849 10917130 10920411
10923693 10926976 10930259 10933542 10936826 10940111 10943396 10946681 10949967
10953254 10956541 10959828 10963116 10966405 10969694 10972983 10976273 10979563
10982854 10986146 10989438 10992730 10996023 10999316 11002610 11005905 11009200
11012495 11015791 11019087 11022384 11025681 11028979 11032278 11035576 11038876
11042176 11045476 11048777 11052078 11055380 11058682 11061985 11065288 11068592
11071896 11075201 11078506 11081812 11085118 11088425 11091732 11095040 11098348
11101657 11104966 11108276 11111586 11114897 11118208 11121520 11124832 11128145
11131458 11134772 11138086 11141401 11144716 11148031 11151348 11154664 11157981
11161299 11164617 11167936 11171255 11174575 11177895 11181215 11184536 11187858
11191180 11194503 11197826 11201149 11204473 11207798 11211123 11214449 11217775
11221101 11224428 11227756 11231084 11234412 11237741 11241071 11244401 11247731
11251062 11254394 11257726 11261058 11264391 11267725 11271059 11274393 11277728
11281064 11284400 11287736 11291073 11294411 11297748 11301087 11304426 11307765
11311105 11314446 11317786 11321128 11324470 11327812 11331155 11334498 11337842
11341187 11344531 11347877 11351223 11354569 11357916 11361263 11364611 11367959
11371308 11374658 11378007 11381358 11384708 11388060 11391412 11394764 11398117
11401470 11404824 11408178 11411533 11414888 11418244 11421600 11424957 11428314
11431672 11435030 11438389 11441748 11445107 11448468 11451828 11455190 11458551
11461913 11465276 11468639 11472003 11475367 11478732 11482097 11485463 11488829
11492195 11495562 11498930 11502298 11505667 11509036 11512406 11515776 11519146
11522517 11525889 11529261 11532634 11536007 11539380 11542754 11546129 11549504
11552879 11556255 11559632 11563009 11566386 11569765 11573143 11576522 11579902
11583282 11586662 11590043 11593424 11596806 11600189 11603572 11606955 11610339
11613724 11617109 11620494 11623880 11627267 11630653 11634041 11637429 11640817
11644206 11647596 11650985 11654376 11657767 11661158 11664550 11667942 11671335
11674729 11678122 11681517 11684912 11688307 11691703 11695099 11698496 11701893
11705291 11708689 11712088 11715487 11718887 11722288 11725688 11729090 11732491
11735894 11739296 11742700 11746103 11749508 11752912 11756318 11759723 11763130
11766536 11769944 11773351 11776759 11780168 11783577 11786987 11790397 11793808
11797219 11800631 11804043 11807456 11810869 11814282 11817697 11821111 11824526
11827942 11831358 11834775 11838192 11841609 11845028 11848446 11851865 11855285
11858705 11862125 11865547 11868968 11872390 11875813 11879236 11882659 11886083
11889508 11892933 11896358 11899785 11903211 11906638 11910066 11913494 11916922
11920351 11923781 11927211 11930641 11934072 11937503 11940935 11944368 11947801
11951234 11954668 11958103 11961538 11964973 11968409 11971845 11975282 11978720
11982158 11985596 11989035 11992474 11995914 11999355 12002795 12006237 12009679
12013121 12016564 12020007 12023451 12026895 12030340 12033785 12037231 12040678
12044124 12047572 12051020 12054468 12057917 12061366 12064816 12068266 12071717
12075168 12078620 12082072 12085525 12088978 12092432 12095886 12099341 12102796
12106252 12109708 12113165 12116622 12120080 12123538 12126996 12130456 12133915
12137375 12140836 12144297 12147759 12151221 12154684 12158147 12161611 12165075
12168539 12172004 12175470 12178936 12182403 12185870 12189337 12192806 12196274
12199743 12203213 12206683 12210153 12213625 12217096 12220568 12224041 12227514
12230987 12234461 12237936 12241411 12244886 12248362 12251839 12255316 12258793

12262271 12265750 12269229 12272708 12276188 12279669 12283150 12286631 12290113
12293596 12297079 12300562 12304046 12307530 12311015 12314501 12317987 12321473
12324960 12328447 12331935 12335424 12338912 12342402 12345892 12349382 12352873
12356364 12359856 12363348 12366841 12370335 12373828 12377323 12380818 12384313
12387809 12391305 12394802 12398299 12401797 12405295 12408794 12412293 12415793
12419293 12422794 12426295 12429797 12433299 12436802 12440305 12443809 12447313
12450818 12454323 12457829 12461335 12464842 12468349 12471857 12475365 12478874
12482383 12485892 12489403 12492913 12496424 12499936 12503448 12506961 12510474
12513988 12517502 12521016 12524532 12528047 12531563 12535080 12538597 12542115
12545633 12549151 12552670 12556190 12559710 12563230 12566752 12570273 12573795
12577318 12580841 12584364 12587888 12591413 12594938 12598463 12601989 12605516
12609043 12612570 12616098 12619627 12623156 12626685 12630215 12633746 12637277
12640808 12644340 12647872 12651405 12654939 12658473 12662007 12665542 12669077
12672613 12676150 12679687 12683224 12686762 12690300 12693839 12697379 12700918
12704459 12708000 12711541 12715083 12718625 12722168 12725711 12729255 12732800
12736344 12739890 12743435 12746982 12750529 12754076 12757624 12761172 12764721
12768270 12771820 12775370 12778921 12782472 12786024 12789576 12793129 12796682
12800236 12803790 12807345 12810900 12814456 12818012 12821568 12825126 12828683
12832242 12835800 12839359 12842919 12846479 12850040 12853601 12857163 12860725
12864287 12867851 12871414 12874978 12878543 12882108 12885674 12889240 12892806
12896373 12899941 12903509 12907078 12910647 12914216 12917786 12921357 12924928
12928500 12932072 12935644 12939217 12942791 12946365 12949939 12953514 12957090
12960666 12964242 12967819 12971397 12974975 12978553 12982132 12985712 12989292
12992872 12996453 13000034 13003616 13007199 13010782 13014365 13017949 13021534
13025118 13028704 13032290 13035876 13039463 13043050 13046638 13050227 13053816
13057405 13060995 13064585 13068176 13071767 13075359 13078952 13082544 13086138
13089732 13093326 13096921 13100516 13104112 13107708 13111305 13114902 13118500
13122098 13125697 13129296 13132896 13136496 13140097 13143698 13147300 13150902
13154505 13158108 13161712 13165316 13168921 13172526 13176132 13179738 13183345
13186952 13190560 13194168 13197776 13201386 13204995 13208605 13212216 13215827
13219439 13223051 13226664 13230277 13233890 13237505 13241119 13244734 13248350
13251966 13255583 13259200 13262817 13266435 13270054 13273673 13277293 13280913
13284533 13288154 13291776 13295398 13299020 13302643 13306267 13309891 13313515
13317141 13320766 13324392 13328019 13331646 13335273 13338901 13342529 13346158
13349788 13353418 13357048 13360679 13364311 13367943 13371575 13375208 13378841
13382475 13386110 13389745 13393380 13397016 13400652 13404289 13407927 13411565
13415203 13418842 13422481 13426121 13429761 13433402 13437044 13440686 13444328
13447971 13451614 13455258 13458902 13462547 13466192 13469838 13473484 13477131
13480778 13484426 13488075 13491723 13495373 13499022 13502673 13506323 13509975
13513626 13517279 13520931 13524585 13528238 13531893 13535547 13539202 13542858
13546514 13550171 13553828 13557486 13561144 13564803 13568462 13572122 13575782
13579442 13583104 13586765 13590427 13594090 13597753 13601417 13605081 13608746
13612411 13616076 13619742 13623409 13627076 13630744 13634412 13638080 13641749
13645419 13649089 13652760 13656431 13660102 13663774 13667447 13671120 13674793
13678467 13682142 13685817 13689492 13693168 13696845 13700522 13704199 13707877
13711556 13715235 13718914 13722594 13726275 13729956 13733637 13737319 13741002
13744685 13748368 13752052 13755736 13759421 13763107 13766793 13770479 13774166
13777853 13781541 13785230 13788918 13792608 13796298 13799988 13803679 13807370

13811062 13814754 13818447 13822141 13825834 13829529 13833224 13836919 13840615
13844311 13848008 13851705 13855403 13859101 13862800 13866499 13870199 13873899
13877600 13881301 13885003 13888705 13892408 13896111 13899815 13903519 13907224
13910929 13914635 13918341 13922048 13925755 13929463 13933171 13936880 13940589
13944298 13948009 13951719 13955430 13959142 13962854 13966567 13970280 13973994
13977708 13981422 13985137 13988853 13992569 13996286 14000003 14003721 14007439
14011157 14014876 14018596 14022316 14026036 14029758 14033479 14037201 14040924
14044647 14048370 14052094 14055819 14059544 14063269 14066995 14070722 14074449
14078176 14081904 14085633 14089362 14093091 14096821 14100552 14104283 14108014
14111746 14115479 14119212 14122945 14126679 14130413 14134148 14137884 14141620
14145356 14149093 14152830 14156568 14160307 14164046 14167785 14171525 14175265
14179006 14182747 14186489 14190232 14193974 14197718 14201462 14205206 14208951
14212696 14216442 14220188 14223935 14227682 14231430 14235178 14238927 14242677
14246426 14250177 14253927 14257679 14261430 14265183 14268935 14272689 14276442
14280197 14283951 14287707 14291462 14295218 14298975 14302732 14306490 14310248
14314007 14317766 14321526 14325286 14329047 14332808 14336570 14340332 14344094
14347858 14351621 14355385 14359150 14362915 14366681 14370447 14374213 14377981
14381748 14385516 14389285 14393054 14396824 14400594 14404364 14408135 14411907
14415679 14419451 14423224 14426998 14430772 14434547 14438322 14442097 14445873
14449650 14453427 14457204 14460982 14464761 14468540 14472319 14476099 14479880
14483661 14487442 14491224 14495007 14498790 14502573 14506357 14510141 14513926
14517712 14521498 14525284 14529071 14532858 14536646 14540435 14544224 14548013
14551803 14555593 14559384 14563176 14566967 14570760 14574553 14578346 14582140
14585934 14589729 14593524 14597320 14601116 14604913 14608711 14612508 14616307
14620106 14623905 14627705 14631505 14635306 14639107 14642909 14646711 14650514
14654317 14658121 14661925 14665730 14669535 14673341 14677147 14680954 14684761
14688569 14692377 14696186 14699995 14703804 14707615 14711425 14715236 14719048
14722860 14726673 14730486 14734300 14738114 14741929 14745744 14749559 14753376
14757192 14761009 14764827 14768645 14772464 14776283 14780102 14783922 14787743
14791564 14795386 14799208 14803030 14806853 14810677 14814501 14818326 14822151
14825976 14829802 14833629 14837456 14841283 14845111 14848940 14852769 14856598
14860428 14864259 14868090 14871921 14875753 14879586 14883419 14887252 14891086
14894921 14898755 14902591 14906427 14910263 14914100 14917938 14921776 14925614
14929453 14933292 14937132 14940973 14944814 14948655 14952497 14956339 14960182
14964025 14967869 14971714 14975558 14979404 14983250 14987096 14990943 14994790
14998638 15002486 15006335 15010184 15014034 15017885 15021735 15025587 15029438
15033291 15037143 15040997 15044851 15048705 15052560 15056415 15060271 15064127
15067984 15071841 15075698 15079557 15083415 15087275 15091134 15094995 15098855
15102716 15106578 15110440 15114303 15118166 15122030 15125894 15129759 15133624
15137490 15141356 15145222 15149089 15152957 15156825 15160694 15164563 15168433
15172303 15176173 15180044 15183916 15187788 15191661 15195534 15199407 15203281
15207156 15211031 15214907 15218783 15222659 15226536 15230414 15234292 15238170
15242049 15245929 15249809 15253689 15257570 15261452 15265334 15269216 15273099
15276983 15280867 15284751 15288636 15292522 15296408 15300294 15304181 15308069
15311956 15315845 15319734 15323623 15327513 15331404 15335294 15339186 15343078
15346970 15350863 15354756 15358650 15362545 15366440 15370335 15374231 15378127
15382024 15385921 15389819 15393718 15397616 15401516 15405416 15409316 15413217
15417118 15421020 15424922 15428825 15432728 15436632 15440536 15444441 15448346

15452252 15456158 15460065 15463972 15467880 15471788 15475697 15479606 15483516 15487426 15491337 15495248 15499160 15503072 15506985 15510898 15514812 15518726 15522641 15526556 15530471 15534388 15538304 15542221 15546139 15550057 15553976 15557895 15561815 15565735 15569655 15573576 15577498 15581420 15585343 15589266 15593189 15597114 15601038 15604963 15608889 15612815 15616741 15620669 15624596 15628524 15632453 15636382 15640311 15644241 15648172 15652103 15656034 15659966 15663899 15667832 15671765 15675699 15679634 15683569 15687504 15691440 15695377 15699314 15703251 15707189 15711128 15715067 15719006 15722946 15726886 15730827 15734769 15738711 15742653 15746596 15750539 15754483 15758428 15762373 15766318 15770264 15774210 15778157 15782105 15786052 15790001 15793950 15797899 15801849 15805799 15809750 15813701 15817653 15821605 15825558 15829512 15833465 15837420 15841374 15845330 15849286 15853242 15857199 15861156 15865114 15869072 15873031 15876990 15880950 15884910 15888871 15892832 15896794 15900756 15904719 15908682 15912646 15916610 15920574 15924540 15928505 15932471 15936438 15940405 15944373 15948341 15952310 15956279 15960249 15964219 15968189 15972161 15976132 15980104 15984077 15988050 15992024 15995998 15999972 16003948 16007923 16011899 16015876 16019853 16023831 16027809 16031787 16035766 16039746 16043726 16047706 16051687 16055669 16059651 16063634 16067617 16071600 16075584 16079569 16083554 16087539 16091525 16095512 16099499 16103486 16107474 16111463 16115452 16119441 16123431 16127421 16131412 16135404 16139396 16143388 16147381 16151375 16155369 16159363 16163358 16167353 16171349 16175346 16179342 16183340 16187338 16191336 16195335 16199334 16203334 16207335 16211335 16215337 16219339 16223341 16227344 16231347 16235351 16239355 16243360 16247366 16251371 16255378 16259384 16263392 16267400 16271408 16275417 16279426 16283436 16287446 16291457 16295468 16299480 16303492 16307505 16311518 16315532 16319546 16323561 16327576 16331592 16335608 16339625 16343642 16347659 16351678 16355696 16359715 16363735 16367755 16371776 16375797 16379819 16383841 16387864 16391887 16395910 16399935 16403959 16407984 16412010 16416036 16420063 16424090 16428117 16432146 16436174 16440203 16444233 16448263 16452293 16456325 16460356 16464388 16468421 16472454 16476487 16480521 16484556 16488591 16492626 16496662 16500699 16504736 16508774 16512812 16516850 16520889 16524929 16528969 16533009 16537050 16541091 16545133 16549176 16553219 16557262 16561306 16565351 16569396 16573441 16577487 16581533 16585580 16589628 16593676 16597724 16601773 16605822 16609872 16613923 16617974 16622025 16626077 16630129 16634182 16638235 16642289 16646344 16650399 16654454 16658510 16662566 16666623 16670680 16674738 16678796 16682855 16686914 16690974 16695035 16699095 16703157 16707218 16711281 16715343 16719407 16723471 16727535 16731600 16735665 16739731 16743797 16747863 16751931 16755998 16760067 16764135 16768205 16772274 16776345 16780415 16784486 16788558 16792630 16796703 16800776 16804850 16808924 16812999 16817074 16821149 16825226 16829302 16833379 16837457 16841535 16845614 16849693 16853773 16857853 16861933 16866014 16870096 16874178 16878261 16882344 16886427 16890511 16894596 16898681 16902767 16906853 16910939 16915026 16919114 16923202 16927290 16931379 16935469 16939559 16943649 16947740 16951832 16955924 16960016 16964109 16968203 16972297 16976391 16980486 16984582 16988678 16992774 16996871 17000969 17005067 17009165 17013264 17017363 17021463 17025564 17029665 17033766 17037868 17041970 17046073 17050177 17054280 17058385 17062490 17066595 17070701 17074807 17078914 17083022 17087129 17091238 17095347 17099456 17103566 17107676 17111787 17115898 17120010 17124122 17128235 17132348 17136462 17140577 17144691 17148807 17152922 17157039 17161155 17165273 17169390 17173509 17177627 17181747

17185866 17189987 17194107 17198229 17202350 17206473 17210595 17214718 17218842
17222966 17227091 17231216 17235342 17239468 17243595 17247722 17251850 17255978
17260106 17264236 17268365 17272495 17276626 17280757 17284889 17289021 17293154
17297287 17301420 17305554 17309689 17313824 17317960 17322096 17326232 17330369
17334507 17338645 17342784 17346923 17351062 17355202 17359343 17363484 17367625
17371767 17375910 17380053 17384196 17388341 17392485 17396630 17400776 17404922
17409068 17413215 17417362 17421510 17425659 17429808 17433957 17438107 17442258
17446409 17450560 17454712 17458865 17463017 17467171 17471325 17475479 17479634
17483790 17487945 17492102 17496259 17500416 17504574 17508732 17512891 17517051
17521210 17525371 17529532 17533693 17537855 17542017 17546180 17550343 17554507
17558672 17562836 17567002 17571168 17575334 17579501 17583668 17587836 17592004
17596173 17600342 17604512 17608682 17612853 17617024 17621196 17625368 17629541
17633714 17637888 17642062 17646237 17650412 17654588 17658764 17662941 17667118
17671296 17675474 17679652 17683832 17688011 17692191 17696372 17700553 17704735
17708917 17713100 17717283 17721467 17725651 17729835 17734021 17738206 17742392
17746579 17750766 17754954 17759142 17763330 17767519 17771709 17775899 17780090
17784281 17788472 17792664 17796857 17801050 17805244 17809438 17813632 17817827
17822023 17826219 17830415 17834612 17838810 17843008 17847206 17851405 17855605
17859805 17864005 17868206 17872408 17876610 17880812 17885015 17889219 17893423
17897627 17901832 17906037 17910243 17914450 17918657 17922864 17927072 17931280
17935489 17939699 17943909 17948119 17952330 17956541 17960753 17964966 17969178
17973392 17977606 17981820 17986035 17990250 17994466 17998682 18002899 18007117
18011334 18015553 18019772 18023991 18028211 18032431 18036652 18040873 18045095
18049317 18053540 18057763 18061987 18066211 18070436 18074661 18078887 18083113
18087340 18091567 18095795 18100023 18104252 18108481 18112711 18116941 18121172
18125403 18129635 18133867 18138100 18142333 18146567 18150801 18155035 18159271
18163506 18167742 18171979 18176216 18180454 18184692 18188931 18193170 18197409
18201649 18205890 18210131 18214373 18218615 18222858 18227101 18231344 18235588
18239833 18244078 18248324 18252570 18256816 18261063 18265311 18269559 18273807
18278056 18282306 18286556 18290807 18295058 18299309 18303561 18307814 18312067
18316320 18320574 18324829 18329084 18333339 18337595 18341851 18346108 18350366
18354624 18358882 18363141 18367401 18371661 18375921 18380182 18384443 18388705
18392968 18397231 18401494 18405758 18410022 18414287 18418553 18422819 18427085
18431352 18435619 18439887 18444155 18448424 18452694 18456963 18461234 18465505
18469776 18474048 18478320 18482593 18486866 18491140 18495414 18499689 18503965
18508240 18512517 18516793 18521071 18525348 18529627 18533905 18538185 18542464
18546745 18551025 18555307 18559588 18563871 18568153 18572437 18576720 18581005
18585289 18589575 18593860 18598146 18602433 18606720 18611008 18615296 18619585
18623874 18628164 18632454 18636745 18641036 18645327 18649619 18653912 18658205
18662499 18666793 18671088 18675383 18679678 18683974 18688271 18692568 18696866
18701164 18705462 18709761 18714061 18718361 18722662 18726963 18731264 18735566
18739869 18744172 18748476 18752780 18757084 18761389 18765695 18770001 18774307
18778614 18782922 18787230 18791538 18795847 18800157 18804467 18808777 18813088
18817400 18821712 18826024 18830337 18834650 18838964 18843279 18847594 18851909
18856225 18860541 18864858 18869176 18873494 18877812 18882131 18886450 18890770
18895091 18899412 18903733 18908055 18912377 18916700 18921023 18925347 18929672
18933996 18938322 18942648 18946974 18951301 18955628 18959956 18964284 18968613
18972942 18977272 18981602 18985933 18990265 18994596 18998929 19003261 19007595

19011928 19016263 19020597 19024933 19029268 19033605 19037941 19042279 19046616
19050955 19055293 19059632 19063972 19068312 19072653 19076994 19081336 19085678
19090021 19094364 19098708 19103052 19107397 19111742 19116087 19120434 19124780
19129127 19133475 19137823 19142172 19146521 19150870 19155221 19159571 19163922
19168274 19172626 19176979 19181332 19185685 19190039 19194394 19198749 19203105
19207461 19211817 19216174 19220532 19224890 19229248 19233607 19237967 19242327
19246687 19251048 19255410 19259772 19264134 19268497 19272861 19277225 19281589
19285954 19290320 19294686 19299052 19303419 19307787 19312155 19316523 19320892
19325262 19329631 19334002 19338373 19342744 19347116 19351489 19355861 19360235
19364609 19368983 19373358 19377733 19382109 19386486 19390863 19395240 19399618
19403996 19408375 19412754 19417134 19421515 19425895 19430277 19434659 19439041
19443424 19447807 19452191 19456575 19460960 19465345 19469731 19474117 19478504
19482891 19487279 19491667 19496056 19500445 19504835 19509225 19513616 19518007
19522399 19526791 19531184 19535577 19539971 19544365 19548760 19553155 19557551
19561947 19566344 19570741 19575139 19579537 19583935 19588335 19592734 19597134
19601535 19605936 19610338 19614740 19619143 19623546 19627949 19632354 19636758
19641163 19645569 19649975 19654382 19658789 19663196 19667604 19672013 19676422
19680832 19685242 19689652 19694063 19698475 19702887 19707300 19711713 19716126
19720540 19724955 19729370 19733785 19738201 19742618 19747035 19751452 19755870
19760289 19764708 19769127 19773547 19777968 19782389 19786810 19791232 19795655
19800078 19804501 19808925 19813349 19817774 19822200 19826626 19831052 19835479
19839906 19844334 19848763 19853192 19857621 19862051 19866481 19870912 19875344
19879775 19884208 19888641 19893074 19897508 19901942 19906377 19910812 19915248
19919685 19924121 19928559 19932997 19937435 19941874 19946313 19950753 19955193
19959634 19964075 19968517 19972959 19977402 19981845 19986289 19990733 19995178
19999623 20004069 20008515 20012962 20017409 20021857 20026305 20030754 20035203
20039653 20044103 20048554 20053005 20057457 20061909 20066362 20070815 20075269
20079723 20084177 20088633 20093088 20097544 20102001 20106458 20110916 20115374
20119833 20124292 20128751 20133211 20137672 20142133 20146595 20151057 20155519
20159983 20164446 20168910 20173375 20177840 20182305 20186772 20191238 20195705
20200173 20204641 20209109 20213578 20218048 20222518 20226988 20231459 20235931
20240403 20244876 20249349 20253822 20258296 20262771 20267245 20271721 20276197
20280673 20285150 20289628 20294106 20298584 20303063 20307543 20312023 20316503
20320984 20325465 20329947 20334430 20338913 20343396 20347880 20352364 20356849
20361335 20365821 20370307 20374794 20379281 20383769 20388257 20392746 20397236
20401726 20406216 20410707 20415198 20419690 20424182 20428675 20433168 20437662
20442157 20446651 20451147 20455642 20460139 20464636 20469133 20473631 20478129
20482628 20487127 20491627 20496127 20500628 20505129 20509631 20514133 20518636
20523139 20527643 20532147 20536652 20541157 20545663 20550169 20554675 20559183
20563690 20568198 20572707 20577216 20581726 20586236 20590747 20595258 20599770
20604282 20608795 20613308 20617821 20622335 20626850 20631365 20635881 20640397
20644914 20649431 20653948 20658466 20662985 20667504 20672024 20676544 20681064
20685585 20690107 20694629 20699152 20703675 20708198 20712722 20717247 20721772
20726297 20730823 20735350 20739877 20744404 20748932 20753461 20757990 20762519
20767049 20771580 20776111 20780642 20785174 20789707 20794240 20798773 20803307
20807842 20812377 20816912 20821448 20825984 20830521 20835059 20839597 20844135
20848674 20853214 20857753 20862294 20866835 20871376 20875918 20880460 20885003
20889547 20894091 20898635 20903180 20907725 20912271 20916817 20921364 20925911

20930459 20935008 20939556 20944106 20948656 20953206 20957757 20962308 20966860
20971412 20975965 20980518 20985072 20989626 20994181 20998736 21003292 21007848
21012405 21016962 21021520 21026078 21030637 21035196 21039756 21044316 21048877
21053438 21058000 21062562 21067125 21071688 21076252 21080816 21085381 21089946
21094511 21099078 21103644 21108211 21112779 21117347 21121916 21126485 21131055
21135625 21140196 21144767 21149338 21153910 21158483 21163056 21167630 21172204
21176778 21181354 21185929 21190505 21195082 21199659 21204236 21208814 21213393
21217972 21222552 21227132 21231712 21236293 21240875 21245457 21250039 21254622
21259206 21263790 21268374 21272959 21277545 21282131 21286717 21291304 21295892
21300480 21305068 21309657 21314247 21318837 21323427 21328018 21332610 21337202
21341794 21346387 21350980 21355574 21360169 21364764 21369359 21373955 21378551
21383148 21387746 21392344 21396942 21401541 21406140 21410740 21415340 21419941
21424543 21429145 21433747 21438350 21442953 21447557 21452161 21456766 21461371
21465977 21470584 21475190 21479798 21484406 21489014 21493623 21498232 21502842
21507452 21512063 21516674 21521286 21525898 21530511 21535124 21539738 21544352
21548967 21553582 21558198 21562814 21567431 21572048 21576666 21581284 21585903
21590522 21595141 21599762 21604382 21609004 21613625 21618247 21622870 21627493
21632117 21636741 21641366 21645991 21650617 21655243 21659869 21664496 21669124
21673752 21678381 21683010 21687640 21692270 21696900 21701531 21706163 21710795
21715428 21720061 21724694 21729328 21733963 21738598 21743234 21747870 21752506
21757143 21761781 21766419 21771057 21775696 21780336 21784976 21789616 21794257
21798899 21803541 21808183 21812826 21817470 21822114 21826758 21831403 21836049
21840695 21845341 21849988 21854636 21859284 21863932 21868581 21873230 21877880
21882531 21887182 21891833 21896485 21901137 21905790 21910444 21915098 21919752
21924407 21929062 21933718 21938375 21943031 21947689 21952347 21957005 21961664
21966323 21970983 21975643 21980304 21984966 21989627 21994290 21998953 22003616
22008280 22012944 22017609 22022274 22026940 22031606 22036273 22040940 22045608
22050276 22054945 22059614 22064284 22068954 22073625 22078296 22082968 22087640
22092313 22096986 22101660 22106334 22111009 22115684 22120360 22125036 22129713
22134390 22139068 22143746 22148424 22153104 22157783 22162464 22167144 22171825
22176507 22181189 22185872 22190555 22195239 22199923 22204607 22209292 22213978
22218664 22223351 22228038 22232725 22237414 22242102 22246791 22251481 22256171
22260862 22265553 22270244 22274936 22279629 22284322 22289015 22293709 22298404
22303099 22307795 22312491 22317187 22321884 22326582 22331280 22335978 22340677
22345377 22350077 22354777 22359478 22364180 22368882 22373584 22378287 22382990
22387694 22392399 22397104 22401809 22406515 22411222 22415929 22420636 22425344
22430052 22434761 22439471 22444181 22448891 22453602 22458313 22463025 22467738
22472450 22477164 22481878 22486592 22491307 22496022 22500738 22505454 22510171
22514889 22519606 22524325 22529044 22533763 22538483 22543203 22547924 22552645
22557367 22562089 22566812 22571535 22576259 22580983 22585708 22590434 22595159
22599886 22604612 22609340 22614067 22618796 22623524 22628254 22632983 22637714
22642444 22647175 22651907 22656639 22661372 22666105 22670839 22675573 22680308
22685043 22689779 22694515 22699252 22703989 22708726 22713465 22718203 22722942
22727682 22732422 22737163 22741904 22746646 22751388 22756130 22760873 22765617
22770361 22775106 22779851 22784596 22789343 22794089 22798836 22803584 22808332
22813080 22817830 22822579 22827329 22832080 22836831 22841582 22846334 22851087
22855840 22860593 22865347 22870102 22874857 22879612 22884368 22889125 22893882
22898639 22903397 22908156 22912915 22917674 22922434 22927195 22931956 22936717

22941479 22946242 22951005 22955768 22960532 22965296 22970061 22974827 22979593 22984359 22989126 22993893 22998661 23003430 23008199 23012968 23017738 23022508 23027279 23032050 23036822 23041595 23046368 23051141 23055915 23060689 23065464 23070239 23075015 23079791 23084568 23089346 23094123 23098902 23103680 23108460 23113240 23118020 23122801 23127582 23132364 23137146 23141929 23146712 23151496 23156280 23161065 23165850 23170636 23175422 23180209 23184996 23189784 23194572 23199361 23204150 23208940 23213730 23218521 23223312 23228103 23232896 23237688 23242482 23247275 23252069 23256864 23261659 23266455 23271251 23276048 23280845 23285642 23290441 23295239 23300038 23304838 23309638 23314439 23319240 23324041 23328844 23333646 23338449 23343253 23348057 23352861 23357667 23362472 23367278 23372085 23376892 23381699 23386507 23391316 23396125 23400935 23405745 23410555 23415366 23420178 23424990 23429802 23434615 23439429 23444243 23449057 23453872 23458688 23463504 23468320 23473137 23477955 23482773 23487591 23492410 23497229 23502049 23506870 23511691 23516512 23521334 23526157 23530980 23535803 23540627 23545451 23550276 23555102 23559927 23564754 23569581 23574408 23579236 23584064 23588893 23593723 23598553 23603383 23608214 23613045 23617877 23622709 23627542 23632375 23637209 23642044 23646878 23651714 23656550 23661386 23666223 23671060 23675898 23680736 23685575 23690414 23695254 23700094 23704935 23709776 23714618 23719460 23724303 23729146 23733990 23738834 23743679 23748524 23753370 23758216 23763063 23767910 23772758 23777606 23782455 23787304 23792154 23797004 23801854 23806706 23811557 23816409 23821262 23826115 23830969 23835823 23840678 23845533 23850389 23855245 23860101 23864958 23869816 23874674 23879533 23884392 23889252 23894112 23898972 23903833 23908695 23913557 23918420 23923283 23928146 23933010 23937875 23942740 23947606 23952472 23957338 23962205 23967073 23971941 23976809 23981678 23986548 23991418 23996288 24001159 24006031 24010903 24015775 24020648 24025522 24030396 24035270 24040145 24045021 24049897 24054773 24059650 24064528 24069406 24074284 24079163 24084042 24088922 24093803 24098684 24103565 24108447 24113329 24118212 24123096 24127980 24132864 24137749 24142634 24147520 24152407 24157293 24162181 24167069 24171957 24176846 24181735 24186625 24191515 24196406 24201298 24206189 24211082 24215975 24220868 24225762 24230656 24235551 24240446 24245342 24250238 24255135 24260032 24264930 24269828 24274727 24279626 24284526 24289426 24294327 24299228 24304130 24309032 24313935 24318838 24323742 24328646 24333551 24338456 24343362 24348268 24353175 24358082 24362990 24367898 24372807 24377716 24382625 24387536 24392446 24397357 24402269 24407181 24412094 24417007 24421921 24426835 24431750 24436665 ;

setarray 10000 wilc1 0 0 0 0 0 0 0 0 1 3 5 7 9 12 15 19 23 27 32 37 43 49 55 62 69 76 84 92 101 110 120 130 140 151 162 173 185 198 211 224 238 252 266 280 296 312 328 345 362 379 397 415 434 453 472 492 513 534 555 577 599 622 645 668 692 716 741 767 792 818 845 872 899 927 956 984 1014 1043 1073 1104 1135 1166 1198 1231 1263 1297 1330 1364 1399 1434 1469 1505 1542 1578 1616 1653 1691 1730 1769 1808 1848 1889 1930 1971 2013 2055 2097 2140 2184 2228 2272 2317 2363 2408 2455 2501 2549 2596 2644 2693 2742 2791 2841 2891 2942 2993 3045 3097 3150 3203 3256 3310 3365 3420 3475 3531 3587 3644 3701 3758 3817 3875 3934 3994 4053 4114 4175 4236 4298 4360 4423 4486 4549 4613 4678 4743 4808 4874 4940 5007 5074 5142 5210 5279 5348 5418 5488 5558 5629 5700 5772 5845 5917 5991 6064 6139 6213 6288 6364 6440 6516 6593 6671 6749 6827 6906 6985 7065 7145 7226 7307 7388 7470 7553 7636 7719 7803 7888 7972 8058 8143 8230 8316 8403 8491 8579 8668 8757 8846 8936 9026 9117 9209 9300 9393 9485 9579 9672 9766 9861 9956 10052 10148 10244 10341

10438 10536 10635 10733 10833 10932 11033 11133 11234 11336 11438 11540 11643 11747
11851 11955 12060 12165 12271 12378 12484 12591 12699 12807 12916 13025 13134 13244
13355 13466 13577 13689 13802 13914 14028 14141 14256 14370 14485 14601 14717 14834
14951 15068 15186 15305 15424 15543 15663 15783 15904 16025 16147 16269 16392 16515
16639 16763 16887 17012 17138 17264 17390 17517 17645 17772 17901 18029 18159 18288
18419 18549 18680 18812 18944 19077 19210 19343 19477 19612 19747 19882 20018 20154
20291 20428 20566 20704 20843 20982 21122 21262 21402 21543 21685 21827 21969 22112
22255 22399 22544 22689 22834 22980 23126 23272 23420 23567 23715 23864 24013 24163
24313 24463 24614 24765 24917 25070 25223 25376 25530 25684 25839 25994 26150 26306
26462 26619 26777 26935 27094 27253 27412 27572 27732 27893 28055 28216 28379 28542
28705 28869 29033 29197 29363 29528 29694 29861 30028 30195 30363 30532 30701 30870
31040 31211 31381 31553 31724 31897 32069 32243 32416 32590 32765 32940 33116 33292
33468 33645 33823 34001 34179 34358 34538 34717 34898 35079 35260 35442 35624 35806
35990 36173 36357 36542 36727 36913 37099 37285 37472 37660 37848 38036 38225 38414
38604 38794 38985 39176 39368 39560 39753 39946 40140 40334 40529 40724 40919 41115
41312 41509 41706 41904 42102 42301 42501 42700 42901 43101 43303 43504 43707 43909
44112 44316 44520 44725 44930 45135 45341 45548 45755 45962 46170 46379 46587 46797
47007 47217 47428 47639 47851 48063 48276 48489 48702 48917 49131 49346 49562 49778
49994 50211 50429 50647 50865 51084 51303 51523 51743 51964 52185 52407 52629 52852
53075 53299 53523 53748 53973 54198 54424 54651 54878 55105 55333 55562 55791 56020
56250 56480 56711 56942 57174 57406 57639 57872 58106 58340 58575 58810 59046 59282
59518 59755 59993 60231 60469 60708 60948 61187 61428 61669 61910 62152 62394 62637
62880 63124 63368 63613 63858 64104 64350 64596 64843 65091 65339 65587 65836 66086
66336 66586 66837 67088 67340 67593 67845 68099 68352 68607 68861 69117 69372 69629
69885 70142 70400 70658 70917 71176 71435 71695 71956 72216 72478 72740 73002 73265
73528 73792 74057 74321 74587 74852 75119 75385 75652 75920 76188 76457 76726 76995
77266 77536 77807 78079 78351 78623 78896 79169 79443 79718 79992 80268 80544 80820
81097 81374 81652 81930 82208 82488 82767 83047 83328 83609 83891 84173 84455 84738
85022 85306 85590 85875 86160 86446 86733 87019 87307 87595 87883 88172 88461 88751
89041 89331 89623 89914 90206 90499 90792 91086 91380 91674 91969 92265 92561 92857
93154 93451 93749 94048 94346 94646 94945 95246 95546 95848 96149 96452 96754 97057
97361 97665 97970 98275 98581 98887 99193 99500 99808 100116 100424 100733 101042
101352 101663 101973 102285 102597 102909 103222 103535 103849 104163 104477
104793 105108 105424 105741 106058 106376 106694 107012 107331 107651 107971
108291 108612 108934 109256 109578 109901 110224 110548 110872 111197 111522
111848 112174 112501 112828 113156 113484 113813 114142 114471 114802 115132
115463 115795 116127 116459 116792 117126 117460 117794 118129 118464 118800
119136 119473 119811 120148 120487 120825 121165 121504 121844 122185 122526
122868 123210 123553 123896 124239 124583 124928 125273 125618 125964 126311
126658 127005 127353 127701 128050 128400 128749 129100 129450 129802 130154
130506 130858 131212 131565 131919 132274 132629 132985 133341 133697 134055
134412 134770 135129 135488 135847 136207 136567 136928 137290 137652 138014
138377 138740 139104 139468 139833 140198 140564 140930 141297 141664 142031
142400 142768 143137 143507 143877 144248 144619 144990 145362 145735 146107
146481 146855 147229 147604 147979 148355 148732 149108 149486 149864 150242
150621 151000 151379 151760 152140 152522 152903 153285 153668 154051 154435
154819 155203 155588 155974 156360 156746 157133 157520 157908 158297 158686
159075 159465 159855 160246 160637 161029 161421 161814 162207 162601 162995

163390 163785 164181 164577 164973 165370 165768 166166 166564 166963 167363
167763 168163 168564 168966 169368 169770 170173 170576 170980 171384 171789
172195 172600 173007 173413 173820 174228 174636 175045 175454 175864 176274
176685 177096 177507 177919 178332 178745 179158 179572 179987 180402 180817
181233 181649 182066 182484 182902 183320 183739 184158 184578 184998 185419
185840 186262 186684 187107 187530 187953 188378 188802 189227 189653 190079
190505 190932 191360 191788 192216 192645 193075 193505 193935 194366 194797
195229 195662 196094 196528 196962 197396 197831 198266 198702 199138 199575
200012 200449 200888 201326 201765 202205 202645 203086 203527 203968 204410
204853 205296 205739 206183 206627 207072 207518 207964 208410 208857 209304
209752 210200 210649 211098 211548 211998 212449 212900 213352 213804 214257
214710 215164 215618 216072 216527 216983 217439 217896 218353 218810 219268
219727 220185 220645 221105 221565 222026 222487 222949 223412 223874 224338
224802 225266 225731 226196 226662 227128 227594 228062 228529 228997 229466
229935 230405 230875 231345 231817 232288 232760 233233 233706 234179 234653
235127 235602 236078 236554 237030 237507 237984 238462 238940 239419 239898
240378 240858 241339 241820 242302 242784 243267 243750 244234 244718 245202
245688 246173 246659 247146 247633 248120 248608 249097 249586 250075 250565
251056 251546 252038 252530 253022 253515 254008 254502 254996 255491 255986
256482 256978 257475 257972 258470 258968 259467 259966 260466 260966 261466
261967 262469 262971 263474 263977 264480 264984 265489 265993 266499 267005
267511 268018 268526 269033 269542 270051 270560 271070 271580 272091 272602
273114 273626 274139 274652 275166 275680 276194 276710 277225 277741 278258
278775 279292 279811 280329 280848 281368 281888 282408 282929 283450 283972
284495 285018 285541 286065 286589 287114 287639 288165 288691 289218 289746
290273 290801 291330 291859 292389 292919 293450 293981 294513 295045 295577
296110 296644 297178 297713 298248 298783 299319 299856 300393 300930 301468
302006 302545 303085 303624 304165 304706 305247 305789 306331 306874 307417
307961 308505 309050 309595 310141 310687 311234 311781 312328 312876 313425
313974 314524 315074 315624 316175 316727 317279 317831 318384 318938 319492
320046 320601 321156 321712 322269 322826 323383 323941 324499 325058 325617
326177 326737 327298 327859 328421 328983 329546 330109 330672 331237 331801
332366 332932 333498 334065 334632 335199 335767 336336 336905 337474 338044
338615 339185 339757 340329 340901 341474 342047 342621 343196 343770 344346
344921 345498 346074 346652 347229 347808 348386 348966 349545 350125 350706
351287 351869 352451 353033 353617 354200 354784 355369 355954 356539 357125
357712 358299 358886 359474 360062 360651 361241 361831 362421 363012 363603
364195 364787 365380 365973 366567 367161 367756 368351 368947 369543 370140
370737 371335 371933 372531 373130 373730 374330 374931 375532 376133 376735
377338 377941 378544 379148 379753 380357 380963 381569 382175 382782 383389
383997 384606 385214 385824 386434 387044 387655 388266 388878 389490 390103
390716 391329 391944 392558 393173 393789 394405 395022 395639 396256 396874
397493 398112 398731 399351 399972 400593 401214 401836 402459 403082 403705
404329 404953 405578 406204 406829 407456 408082 408710 409338 409966 410595
411224 411854 412484 413114 413746 414377 415009 415642 416275 416909 417543
418177 418813 419448 420084 420721 421358 421995 422633 423272 423911 424550
425190 425830 426471 427113 427755 428397 429040 429683 430327 430971 431616
432261 432907 433553 434200 434847 435495 436143 436792 437441 438091 438741

439392 440043 440694 441346 441999 442652 443306 443960 444614 445269 445925
446581 447237 447894 448552 449209 449868 450527 451186 451846 452506 453167
453829 454490 455153 455815 456479 457143 457807 458472 459137 459802 460469
461135 461803 462470 463138 463807 464476 465146 465816 466486 467157 467829
468501 469174 469847 470520 471194 471869 472544 473219 473895 474571 475248
475926 476604 477282 477961 478640 479320 480000 480681 481362 482044 482726
483409 484092 484776 485460 486145 486830 487516 488202 488889 489576 490263
490951 491640 492329 493019 493709 494399 495090 495782 496474 497166 497859
498553 499247 499941 500636 501331 502027 502724 503421 504118 504816 505514
506213 506912 507612 508312 509013 509714 510416 511118 511821 512524 513228
513932 514637 515342 516047 516753 517460 518167 518875 519583 520291 521000
521710 522420 523130 523841 524553 525265 525977 526690 527404 528118 528832
529547 530262 530978 531694 532411 533129 533846 534565 535283 536003 536722
537443 538163 538885 539606 540329 541051 541774 542498 543222 543947 544672
545397 546124 546850 547577 548305 549033 549761 550490 551220 551950 552680
553411 554143 554875 555607 556340 557073 557807 558542 559276 560012 560748
561484 562221 562958 563696 564434 565173 565912 566652 567392 568133 568874
569615 570358 571100 571843 572587 573331 574076 574821 575566 576312 577059
577806 578554 579302 580050 580799 581549 582299 583049 583800 584551 585303
586056 586809 587562 588316 589070 589825 590580 591336 592092 592849 593606
594364 595122 595881 596640 597400 598160 598921 599682 600444 601206 601969
602732 603495 604259 605024 605789 606555 607321 608087 608854 609622 610390
611158 611927 612697 613466 614237 615008 615779 616551 617324 618096 618870
619644 620418 621193 621968 622744 623520 624297 625074 625852 626630 627409
628188 628968 629748 630529 631310 632091 632873 633656 634439 635223 636007
636791 637577 638362 639148 639935 640722 641509 642297 643086 643875 644664
645454 646244 647035 647827 648619 649411 650204 650997 651791 652585 653380
654175 654971 655767 656564 657361 658159 658957 659756 660555 661355 662155
662956 663757 664559 665361 666163 666966 667770 668574 669379 670184 670989
671795 672602 673409 674216 675024 675833 676642 677451 678261 679071 679882
680694 681506 682318 683131 683944 684758 685572 686387 687202 688018 688835
689651 690469 691286 692105 692923 693742 694562 695382 696203 697024 697846
698668 699490 700314 701137 701961 702786 703611 704436 705262 706089 706916
707743 708571 709400 710229 711058 711888 712719 713550 714381 715213 716045
716878 717711 718545 719380 720214 721050 721885 722722 723559 724396 725234
726072 726910 727750 728589 729430 730270 731111 731953 732795 733638 734481
735324 736169 737013 737858 738704 739550 740396 741243 742091 742939 743787
744636 745486 746336 747186 748037 748888 749740 750593 751446 752299 753153
754007 754862 755717 756573 757429 758286 759143 760001 760859 761718 762577
763437 764297 765158 766019 766881 767743 768605 769469 770332 771196 772061
772926 773791 774657 775524 776391 777259 778127 778995 779864 780734 781603
782474 783345 784216 785088 785961 786833 787707 788581 789455 790330 791205
792081 792957 793834 794711 795589 796467 797346 798225 799105 799985 800866
801747 802629 803511 804394 805277 806160 807045 807929 808814 809700 810586
811472 812359 813247 814135 815024 815913 816802 817692 818582 819473 820365
821257 822149 823042 823935 824829 825724 826619 827514 828410 829306 830203
831100 831998 832896 833795 834694 835594 836494 837395 838296 839198 840100
841003 841906 842810 843714 844618 845523 846429 847335 848242 849149 850056

850964 851873 852782 853691 854601 855512 856423 857334 858246 859159 860072
860985 861899 862813 863728 864644 865560 866476 867393 868310 869228 870146
871065 871984 872904 873824 874745 875666 876588 877510 878433 879356 880280
881204 882129 883054 883979 884905 885832 886759 887687 888615 889543 890472
891402 892332 893263 894194 895125 896057 896989 897922 898856 899790 900724
901659 902595 903530 904467 905404 906341 907279 908217 909156 910095 911035
911975 912916 913858 914799 915742 916684 917627 918571 919515 920460 921405
922351 923297 924244 925191 926138 927087 928035 928984 929934 930884 931834
932785 933737 934689 935642 936595 937548 938502 939456 940411 941367 942323
943279 944236 945193 946151 947110 948069 949028 949988 950948 951909 952870
953832 954794 955757 956720 957684 958648 959613 960578 961544 962510 963477
964444 965412 966380 967348 968318 969287 970257 971228 972199 973171 974143
975115 976088 977062 978036 979010 979985 980961 981937 982913 983890 984867
985845 986824 987803 988782 989762 990742 991723 992705 993686 994669 995652
996635 997619 998603 999588 1000573 1001559 1002545 1003532 1004519 1005507
1006495 1007483 1008473 1009462 1010452 1011443 1012434 1013426 1014418 1015410
1016404 1017397 1018391 1019386 1020381 1021376 1022372 1023369 1024366 1025363
1026361 1027360 1028359 1029358 1030358 1031358 1032359 1033361 1034363 1035365
1036368 1037371 1038375 1039379 1040384 1041389 1042395 1043402 1044408 1045416
1046423 1047432 1048440 1049450 1050459 1051469 1052480 1053491 1054503 1055515
1056528 1057541 1058554 1059569 1060583 1061598 1062614 1063630 1064646 1065664
1066681 1067699 1068718 1069737 1070756 1071776 1072796 1073817 1074839 1075861
1076883 1077906 1078930 1079953 1080978 1082003 1083028 1084054 1085080 1086107
1087134 1088162 1089190 1090219 1091249 1092278 1093309 1094339 1095370 1096402
1097434 1098467 1099500 1100534 1101568 1102603 1103638 1104674 1105710 1106746
1107783 1108821 1109859 1110898 1111937 1112976 1114016 1115057 1116098 1117139
1118181 1119224 1120267 1121310 1122354 1123399 1124443 1125489 1126535 1127581
1128628 1129675 1130723 1131772 1132820 1133870 1134920 1135970 1137021 1138072
1139124 1140176 1141229 1142282 1143336 1144390 1145445 1146500 1147556 1148612
1149668 1150726 1151783 1152841 1153900 1154959 1156019 1157079 1158139 1159200
1160262 1161324 1162387 1163450 1164513 1165577 1166642 1167707 1168772 1169838
1170904 1171971 1173039 1174107 1175175 1176244 1177313 1178383 1179454 1180525
1181596 1182668 1183740 1184813 1185886 1186960 1188034 1189109 1190184 1191260
1192336 1193413 1194490 1195568 1196646 1197725 1198804 1199884 1200964 1202044
1203126 1204207 1205289 1206372 1207455 1208539 1209623 1210707 1211792 1212878
1213964 1215050 1216137 1217225 1218313 1219401 1220490 1221580 1222670 1223760
1224851 1225942 1227034 1228127 1229220 1230313 1231407 1232501 1233596 1234691
1235787 1236883 1237980 1239078 1240175 1241274 1242372 1243472 1244571 1245671
1246772 1247873 1248975 1250077 1251180 1252283 1253387 1254491 1255596 1256701
1257806 1258912 1260019 1261126 1262234 1263342 1264450 1265559 1266669 1267779
1268889 1270000 1271112 1272224 1273336 1274449 1275563 1276676 1277791 1278906
1280021 1281137 1282253 1283370 1284488 1285605 1286724 1287843 1288962 1290082
1291202 1292323 1293444 1294566 1295688 1296811 1297934 1299058 1300182 1301307
1302432 1303558 1304684 1305810 1306938 1308065 1309193 1310322 1311451 1312581
1313711 1314841 1315972 1317104 1318236 1319368 1320501 1321635 1322769 1323903
1325038 1326174 1327310 1328446 1329583 1330721 1331859 1332997 1334136 1335275
1336415 1337556 1338696 1339838 1340980 1342122 1343265 1344408 1345552 1346696
1347841 1348986 1350132 1351278 1352425 1353572 1354720 1355868 1357017 1358166

1359316 1360466 1361616 1362768 1363919 1365071 1366224 1367377 1368531 1369685
1370839 1371994 1373150 1374306 1375462 1376619 1377777 1378935 1380093 1381252
1382412 1383572 1384732 1385893 1387055 1388217 1389379 1390542 1391705 1392869
1394033 1395198 1396364 1397529 1398696 1399863 1401030 1402198 1403366 1404535
1405704 1406874 1408044 1409215 1410386 1411558 1412730 1413903 1415076 1416249
1417424 1418598 1419773 1420949 1422125 1423302 1424479 1425656 1426835 1428013
1429192 1430372 1431552 1432732 1433913 1435095 1436277 1437459 1438642 1439826
1441010 1442194 1443379 1444564 1445750 1446937 1448124 1449311 1450499 1451687
1452876 1454066 1455255 1456446 1457636 1458828 1460020 1461212 1462405 1463598
1464792 1465986 1467181 1468376 1469572 1470768 1471964 1473162 1474359 1475557
1476756 1477955 1479155 1480355 1481556 1482757 1483958 1485160 1486363 1487566
1488770 1489974 1491178 1492383 1493589 1494795 1496001 1497208 1498416 1499624
1500832 1502041 1503250 1504460 1505671 1506882 1508093 1509305 1510517 1511730
1512944 1514157 1515372 1516587 1517802 1519018 1520234 1521451 1522668 1523886
1525104 1526323 1527542 1528762 1529982 1531203 1532424 1533646 1534868 1536090
1537313 1538537 1539761 1540986 1542211 1543437 1544663 1545889 1547116 1548344
1549572 1550801 1552030 1553259 1554489 1555720 1556951 1558182 1559414 1560646
1561879 1563113 1564347 1565581 1566816 1568052 1569287 1570524 1571761 1572998
1574236 1575474 1576713 1577952 1579192 1580433 1581673 1582915 1584156 1585399
1586641 1587885 1589128 1590373 1591617 1592863 1594108 1595354 1596601 1597848
1599096 1600344 1601593 1602842 1604091 1605342 1606592 1607843 1609095 1610347
1611599 1612852 1614106 1615360 1616615 1617870 1619125 1620381 1621638 1622894
1624152 1625410 1626668 1627927 1629187 1630447 1631707 1632968 1634229 1635491
1636753 1638016 1639280 1640544 1641808 1643073 1644338 1645604 1646870 1648137
1649404 1650672 1651940 1653209 1654478 1655748 1657018 1658289 1659560 1660831
1662104 1663376 1664649 1665923 1667197 1668472 1669747 1671022 1672299 1673575
1674852 1676130 1677408 1678686 1679965 1681245 1682525 1683805 1685086 1686368
1687650 1688932 1690215 1691499 1692783 1694067 1695352 1696637 1697923 1699210
1700496 1701784 1703072 1704360 1705649 1706938 1708228 1709518 1710809 1712100
1713392 1714684 1715977 1717270 1718564 1719858 1721153 1722448 1723744 1725040
1726337 1727634 1728932 1730230 1731528 1732828 1734127 1735427 1736728 1738029
1739331 1740633 1741935 1743238 1744542 1745846 1747150 1748455 1749761 1751067
1752373 1753680 1754988 1756296 1757604 1758913 1760223 1761532 1762843 1764154
1765465 1766777 1768089 1769402 1770716 1772029 1773344 1774659 1775974 1777290
1778606 1779923 1781240 1782558 1783876 1785195 1786514 1787834 1789154 1790475
1791796 1793118 1794440 1795762 1797086 1798409 1799733 1801058 1802383 1803709
1805035 1806361 1807688 1809016 1810344 1811673 1813002 1814331 1815661 1816992
1818323 1819654 1820986 1822319 1823652 1824985 1826319 1827653 1828988 1830324
1831660 1832996 1834333 1835670 1837008 1838347 1839685 1841025 1842365 1843705
1845046 1846387 1847729 1849071 1850414 1851757 1853101 1854445 1855790 1857135
1858481 1859827 1861174 1862521 1863869 1865217 1866565 1867915 1869264 1870614
1871965 1873316 1874668 1876020 1877372 1878725 1880079 1881433 1882788 1884143
1885498 1886854 1888211 1889568 1890925 1892283 1893642 1895001 1896360 1897720
1899080 1900441 1901803 1903165 1904527 1905890 1907253 1908617 1909982 1911346
1912712 1914078 1915444 1916811 1918178 1919546 1920914 1922283 1923652 1925022
1926392 1927763 1929134 1930506 1931878 1933251 1934624 1935997 1937372 1938746
1940121 1941497 1942873 1944250 1945627 1947005 1948383 1949761 1951140 1952520
1953900 1955280 1956661 1958043 1959425 1960808 1962191 1963574 1964958 1966342

1967727 1969113 1970499 1971885 1973272 1974660 1976047 1977436 1978825 1980214
1981604 1982994 1984385 1985777 1987168 1988561 1989953 1991347 1992741 1994135
1995530 1996925 1998321 1999717 2001114 2002511 2003909 2005307 2006705 2008105
2009504 2010904 2012305 2013706 2015108 2016510 2017913 2019316 2020719 2022124
2023528 2024933 2026339 2027745 2029151 2030558 2031966 2033374 2034782 2036191
2037601 2039011 2040421 2041832 2043244 2044656 2046068 2047481 2048895 2050308
2051723 2053138 2054553 2055969 2057385 2058802 2060220 2061637 2063056 2064475
2065894 2067314 2068734 2070155 2071576 2072998 2074420 2075843 2077266 2078690
2080114 2081539 2082964 2084389 2085816 2087242 2088669 2090097 2091525 2092954
2094383 2095813 2097243 2098673 2100104 2101536 2102968 2104401 2105834 2107267
2108701 2110136 2111571 2113006 2114442 2115879 2117316 2118753 2120191 2121630
2123068 2124508 2125948 2127388 2128829 2130271 2131712 2133155 2134598 2136041
2137485 2138929 2140374 2141819 2143265 2144711 2146158 2147605 2149053 2150502
2151950 2153400 2154849 2156299 2157750 2159201 2160653 2162105 2163558 2165011
2166465 2167919 2169374 2170829 2172284 2173740 2175197 2176654 2178112 2179570
2181028 2182487 2183947 2185407 2186868 2188329 2189790 2191252 2192715 2194178
2195641 2197105 2198570 2200034 2201500 2202966 2204432 2205899 2207367 2208834
2210303 2211772 2213241 2214711 2216181 2217652 2219123 2220595 2222068 2223540
2225014 2226487 2227962 2229437 2230912 2232388 2233864 2235341 2236818 2238295
2239774 2241252 2242732 2244211 2245691 2247172 2248653 2250135 2251617 2253100
2254583 2256066 2257550 2259035 2260520 2262006 2263492 2264978 2266465 2267953
2269441 2270929 2272418 2273908 2275398 2276888 2278379 2279871 2281363 2282855
2284348 2285841 2287335 2288830 2290325 2291820 2293316 2294812 2296309 2297807
2299304 2300803 2302302 2303801 2305301 2306801 2308302 2309803 2311305 2312807
2314310 2315813 2317317 2318821 2320326 2321831 2323337 2324843 2326349 2327857
2329364 2330872 2332381 2333890 2335400 2336910 2338421 2339932 2341443 2342955
2344468 2345981 2347495 2349009 2350523 2352038 2353554 2355070 2356586 2358103
2359621 2361139 2362657 2364176 2365695 2367215 2368736 2370257 2371778 2373300
2374822 2376345 2377869 2379393 2380917 2382442 2383967 2385493 2387019 2388546
2390073 2391601 2393129 2394658 2396188 2397717 2399248 2400778 2402309 2403841
2405373 2406906 2408439 2409973 2411507 2413042 2414577 2416113 2417649 2419185
2420723 2422260 2423798 2425337 2426876 2428416 2429956 2431496 2433037 2434579
2436121 2437663 2439206 2440750 2442294 2443838 2445383 2446929 2448475 2450021
2451568 2453116 2454664 2456212 2457761 2459310 2460860 2462411 2463961 2465513
2467065 2468617 2470170 2471723 2473277 2474831 2476386 2477941 2479497 2481053
2482610 2484167 2485725 2487283 2488842 2490401 2491961 2493521 2495082 2496643
2498205 2499767 2501330 2502893 2504456 2506021 2507585 2509150 2510716 2512282
2513849 2515416 2516983 2518551 2520120 2521689 2523259 2524829 2526399 2527970
2529542 2531114 2532686 2534259 2535833 2537406 2538981 2540556 2542131 2543707
2545284 2546861 2548438 2550016 2551594 2553173 2554752 2556332 2557913 2559493
2561075 2562657 2564239 2565822 2567405 2568989 2570573 2572158 2573743 2575329
2576915 2578502 2580089 2581677 2583265 2584854 2586443 2588032 2589623 2591213
2592804 2594396 2595988 2597581 2599174 2600768 2602362 2603956 2605551 2607147
2608743 2610340 2611937 2613534 2615132 2616731 2618330 2619929 2621529 2623130
2624731 2626332 2627934 2629537 2631139 2632743 2634347 2635951 2637556 2639162
2640767 2642374 2643981 2645588 2647196 2648804 2650413 2652022 2653632 2655242
2656853 2658465 2660076 2661689 2663301 2664915 2666528 2668143 2669757 2671373
2672988 2674605 2676221 2677838 2679456 2681074 2682693 2684312 2685932 2687552

2689172 2690794 2692415 2694037 2695660 2697283 2698907 2700531 2702155 2703780
2705406 2707032 2708658 2710285 2711913 2713541 2715169 2716798 2718428 2720058
2721688 2723319 2724951 2726582 2728215 2729848 2731481 2733115 2734750 2736384
2738020 2739656 2741292 2742929 2744566 2746204 2747842 2749481 2751120 2752760
2754400 2756041 2757682 2759324 2760966 2762609 2764252 2765896 2767540 2769185
2770830 2772476 2774122 2775769 2777416 2779064 2780712 2782360 2784009 2785659
2787309 2788960 2790611 2792262 2793915 2795567 2797220 2798874 2800528 2802182
2803837 2805493 2807149 2808805 2810462 2812120 2813778 2815436 2817095 2818755
2820415 2822075 2823736 2825397 2827059 2828722 2830384 2832048 2833712 2835376
2837041 2838706 2840372 2842038 2843705 2845373 2847040 2848709 2850377 2852047
2853716 2855387 2857057 2858729 2860400 2862073 2863745 2865419 2867092 2868766
2870441 2872116 2873792 2875468 2877145 2878822 2880500 2882178 2883856 2885535
2887215 2888895 2890576 2892257 2893938 2895620 2897303 2898986 2900669 2902353
2904038 2905723 2907408 2909094 2910781 2912468 2914155 2915843 2917532 2919221
2920910 2922600 2924290 2925981 2927673 2929364 2931057 2932750 2934443 2936137
2937831 2939526 2941221 2942917 2944614 2946310 2948008 2949705 2951404 2953102
2954802 2956501 2958202 2959902 2961604 2963305 2965007 2966710 2968413 2970117
2971821 2973526 2975231 2976937 2978643 2980349 2982057 2983764 2985472 2987181
2988890 2990599 2992310 2994020 2995731 2997443 2999155 3000867 3002580 3004294
3006008 3007722 3009437 3011153 3012869 3014585 3016302 3018020 3019737 3021456
3023175 3024894 3026614 3028334 3030055 3031777 3033499 3035221 3036944 3038667
3040391 3042115 3043840 3045565 3047291 3049017 3050744 3052472 3054199 3055928
3057656 3059386 3061115 3062845 3064576 3066307 3068039 3069771 3071504 3073237
3074971 3076705 3078440 3080175 3081910 3083646 3085383 3087120 3088858 3090596
3092334 3094073 3095813 3097553 3099294 3101035 3102776 3104518 3106261 3108004
3109747 3111491 3113236 3114981 3116726 3118472 3120218 3121965 3123713 3125461
3127209 3128958 3130707 3132457 3134208 3135959 3137710 3139462 3141214 3142967
3144720 3146474 3148228 3149983 3151739 3153494 3155251 3157007 3158765 3160522
3162281 3164039 3165799 3167558 3169318 3171079 3172840 3174602 3176364 3178127
3179890 3181654 3183418 3185182 3186948 3188713 3190479 3192246 3194013 3195781
3197549 3199317 3201086 3202856 3204626 3206396 3208167 3209939 3211711 3213483
3215256 3217030 3218804 3220578 3222353 3224129 3225905 3227681 3229458 3231236
3233013 3234792 3236571 3238350 3240130 3241910 3243691 3245473 3247254 3249037
3250820 3252603 3254387 3256171 3257956 3259741 3261527 3263313 3265100 3266887
3268675 3270463 3272252 3274041 3275831 3277621 3279412 3281203 3282994 3284787
3286579 3288372 3290166 3291960 3293755 3295550 3297346 3299142 3300938 3302735
3304533 3306331 3308130 3309929 3311728 3313528 3315329 3317130 3318931 3320733
3322536 3324339 3326142 3327946 3329751 3331556 3333361 3335167 3336973 3338780
3340588 3342396 3344204 3346013 3347822 3349632 3351443 3353253 3355065 3356877
3358689 3360502 3362315 3364129 3365943 3367758 3369573 3371389 3373205 3375022
3376839 3378657 3380475 3382294 3384113 3385933 3387753 3389574 3391395 3393217
3395039 3396861 3398685 3400508 3402332 3404157 3405982 3407808 3409634 3411460
3413288 3415115 3416943 3418772 3420601 3422430 3424260 3426091 3427922 3429753
3431585 3433418 3435251 3437084 3438918 3440753 3442588 3444423 3446259 3448096
3449932 3451770 3453608 3455446 3457285 3459124 3460964 3462805 3464646 3466487
3468329 3470171 3472014 3473857 3475701 3477545 3479390 3481235 3483081 3484927
3486774 3488621 3490469 3492317 3494166 3496015 3497865 3499715 3501566 3503417
3505269 3507121 3508974 3510827 3512680 3514535 3516389 3518244 3520100 3521956

3523813 3525670 3527527 3529385 3531244 3533103 3534962 3536822 3538683 3540544
3542406 3544268 3546130 3547993 3549857 3551721 3553585 3555450 3557315 3559181
3561048 3562915 3564782 3566650 3568518 3570387 3572257 3574127 3575997 3577868
3579739 3581611 3583483 3585356 3587229 3589103 3590978 3592852 3594728 3596603
3598480 3600356 3602234 3604111 3605990 3607868 3609748 3611627 3613508 3615388
3617269 3619151 3621033 3622916 3624799 3626683 3628567 3630452 3632337 3634222
3636108 3637995 3639882 3641770 3643658 3645546 3647435 3649325 3651215 3653105
3654996 3656888 3658780 3660673 3662566 3664459 3666353 3668247 3670142 3672038
3673934 3675830 3677727 3679625 3681523 3683421 3685320 3687219 3689119 3691019
3692920 3694822 3696724 3698626 3700529 3702432 3704336 3706240 3708145 3710050
3711956 3713862 3715769 3717676 3719584 3721492 3723401 3725310 3727220 3729130
3731041 3732952 3734864 3736776 3738689 3740602 3742516 3744430 3746344 3748259
3750175 3752091 3754008 3755925 3757843 3759761 3761679 3763598 3765518 3767438
3769358 3771279 3773201 3775123 3777046 3778969 3780892 3782816 3784740 3786665
3788591 3790517 3792443 3794370 3796298 3798226 3800154 3802083 3804012 3805942
3807873 3809804 3811735 3813667 3815599 3817532 3819465 3821399 3823333 3825268
3827204 3829139 3831076 3833012 3834950 3836887 3838826 3840765 3842704 3844643
3846584 3848524 3850466 3852407 3854350 3856292 3858235 3860179 3862123 3864068
3866013 3867959 3869905 3871851 3873799 3875746 3877694 3879643 3881592 3883542
3885492 3887442 3889393 3891345 3893297 3895249 3897202 3899156 3901110 3903064
3905019 3906975 3908931 3910887 3912844 3914802 3916760 3918718 3920677 3922636
3924596 3926557 3928518 3930479 3932441 3934403 3936366 3938329 3940293 3942258
3944222 3946188 3948154 3950120 3952087 3954054 3956022 3957990 3959959 3961928
3963898 3965868 3967839 3969810 3971782 3973754 3975727 3977700 3979674 3981648
3983622 3985598 3987573 3989549 3991526 3993503 3995481 3997459 3999437 4001417
4003396 4005376 4007357 4009338 4011319 4013301 4015284 4017267 4019251 4021235
4023219 4025204 4027190 4029176 4031162 4033149 4035137 4037125 4039113 4041102
4043091 4045081 4047072 4049063 4051054 4053046 4055038 4057031 4059025 4061018
4063013 4065008 4067003 4068999 4070995 4072992 4074989 4076987 4078985 4080984
4082983 4084983 4086983 4088984 4090985 4092987 4094989 4096992 4098995 4100999
4103003 4105008 4107013 4109019 4111025 4113032 4115039 4117046 4119055 4121063
4123072 4125082 4127092 4129103 4131114 4133125 4135137 4137150 4139163 4141177
4143191 4145205 4147220 4149236 4151252 4153268 4155285 4157303 4159321 4161339
4163358 4165378 4167398 4169418 4171439 4173460 4175482 4177505 4179528 4181551
4183575 4185599 4187624 4189650 4191675 4193702 4195729 4197756 4199784 4201812
4203841 4205870 4207900 4209930 4211961 4213992 4216024 4218056 4220089 4222122
4224156 4226190 4228225 4230260 4232296 4234332 4236369 4238406 4240444 4242482
4244520 4246560 4248599 4250639 4252680 4254721 4256763 4258805 4260847 4262890
4264934 4266978 4269023 4271068 4273113 4275159 4277206 4279253 4281300 4283348
4285397 4287446 4289495 4291545 4293596 4295647 4297698 4299750 4301803 4303856
4305909 4307963 4310017 4312072 4314128 4316183 4318240 4320297 4322354 4324412
4326470 4328529 4330588 4332648 4334709 4336769 4338831 4340892 4342955 4345018
4347081 4349145 4351209 4353274 4355339 4357405 4359471 4361537 4363605 4365672
4367741 4369809 4371878 4373948 4376018 4378089 4380160 4382232 4384304 4386376
4388450 4390523 4392597 4394672 4396747 4398822 4400899 4402975 4405052 4407130
4409208 4411286 4413365 4415445 4417525 4419605 4421686 4423768 4425850 4427932
4430015 4432099 4434183 4436267 4438352 4440437 4442523 4444610 4446697 4448784
4450872 4452960 4455049 4457138 4459228 4461319 4463410 4465501 4467593 4469685

4471778 4473871 4475965 4478059 4480154 4482249 4484345 4486441 4488538 4490635
4492733 4494831 4496930 4499029 4501129 4503229 4505330 4507431 4509532 4511635
4513737 4515840 4517944 4520048 4522153 4524258 4526364 4528470 4530576 4532683
4534791 4536899 4539008 4541117 4543226 4545336 4547447 4549558 4551669 4553781
4555894 4558007 4560120 4562234 4564349 4566464 4568579 4570695 4572811 4574928
4577046 4579164 4581282 4583401 4585520 4587640 4589761 4591882 4594003 4596125
4598247 4600370 4602493 4604617 4606741 4608866 4610992 4613117 4615244 4617370
4619498 4621625 4623754 4625882 4628012 4630141 4632272 4634402 4636534 4638665
4640798 4642930 4645063 4647197 4649331 4651466 4653601 4655737 4657873 4660009
4662147 4664284 4666422 4668561 4670700 4672840 4674980 4677120 4679261 4681403
4683545 4685687 4687830 4689974 4692118 4694262 4696407 4698553 4700699 4702845
4704992 4707140 4709288 4711436 4713585 4715735 4717885 4720035 4722186 4724337
4726489 4728642 4730794 4732948 4735102 4737256 4739411 4741566 4743722 4745878
4748035 4750192 4752350 4754509 4756667 4758827 4760986 4763147 4765307 4767469
4769630 4771793 4773955 4776119 4778282 4780447 4782611 4784776 4786942 4789108
4791275 4793442 4795610 4797778 4799947 4802116 4804285 4806455 4808626 4810797
4812969 4815141 4817313 4819486 4821660 4823834 4826009 4828184 4830359 4832535
4834712 4836889 4839066 4841244 4843423 4845602 4847781 4849961 4852142 4854322
4856504 4858686 4860868 4863051 4865235 4867418 4869603 4871788 4873973 4876159
4878345 4880532 4882720 4884907 4887096 4889285 4891474 4893664 4895854 4898045
4900236 4902428 4904620 4906813 4909006 4911200 4913394 4915589 4917784 4919980
4922176 4924373 4926570 4928768 4930966 4933165 4935364 4937563 4939764 4941964
4944165 4946367 4948569 4950772 4952975 4955178 4957383 4959587 4961792 4963998
4966204 4968410 4970617 4972825 4975033 4977242 4979451 4981660 4983870 4986081
4988292 4990503 4992715 4994928 4997141 4999354 5001568 5003782 5005997 5008213
5010429 5012645 5014862 5017079 5019297 5021516 5023734 5025954 5028174 5030394
5032615 5034836 5037058 5039280 5041503 5043727 5045950 5048175 5050399 5052625
5054850 5057077 5059303 5061531 5063758 5065987 5068215 5070445 5072674 5074904
5077135 5079366 5081598 5083830 5086063 5088296 5090530 5092764 5094998 5097234
5099469 5101705 5103942 5106179 5108417 5110655 5112893 5115132 5117372 5119612
5121853 5124094 5126335 5128577 5130820 5133063 5135306 5137550 5139795 5142040
5144285 5146531 5148777 5151024 5153272 5155520 5157768 5160017 5162267 5164516
5166767 5169018 5171269 5173521 5175773 5178026 5180280 5182533 5184788 5187043
5189298 5191554 5193810 5196067 5198324 5200582 5202840 5205099 5207358 5209618
5211878 5214139 5216400 5218662 5220924 5223187 5225450 5227714 5229978 5232243
5234508 5236774 5239040 5241306 5243573 5245841 5248109 5250378 5252647 5254917
5257187 5259457 5261728 5264000 5266272 5268545 5270818 5273091 5275365 5277640
5279915 5282190 5284466 5286743 5289020 5291297 5293575 5295854 5298133 5300412
5302692 5304973 5307254 5309535 5311817 5314099 5316382 5318666 5320950 5323234
5325519 5327804 5330090 5332376 5334663 5336951 5339238 5341527 5343816 5346105
5348395 5350685 5352976 5355267 5357559 5359851 5362144 5364437 5366731 5369025
5371320 5373615 5375911 5378207 5380504 5382801 5385099 5387397 5389696 5391995
5394294 5396595 5398895 5401196 5403498 5405800 5408103 5410406 5412709 5415014
5417318 5419623 5421929 5424235 5426541 5428848 5431156 5433464 5435773 5438082
5440391 5442701 5445012 5447323 5449634 5451946 5454259 5456571 5458885 5461199
5463513 5465828 5468144 5470460 5472776 5475093 5477410 5479728 5482047 5484365
5486685 5489005 5491325 5493646 5495967 5498289 5500611 5502934 5505258 5507581
5509906 5512230 5514556 5516882 5519208 5521535 5523862 5526190 5528518 5530847

5533176 5535505 5537836 5540166 5542498 5544829 5547161 5549494 5551827 5554161
5556495 5558830 5561165 5563500 5565837 5568173 5570510 5572848 5575186 5577525
5579864 5582203 5584543 5586884 5589225 5591566 5593909 5596251 5598594 5600938
5603282 5605626 5607971 5610317 5612663 5615009 5617356 5619703 5622051 5624400
5626749 5629098 5631448 5633799 5636149 5638501 5640853 5643205 5645558 5647911
5650265 5652620 5654974 5657330 5659686 5662042 5664399 5666756 5669114 5671472
5673831 5676190 5678550 5680910 5683271 5685632 5687994 5690356 5692719 5695082
5697446 5699810 5702175 5704540 5706906 5709272 5711639 5714006 5716373 5718742
5721110 5723479 5725849 5728219 5730590 5732961 5735332 5737704 5740077 5742450
5744824 5747198 5749572 5751947 5754323 5756699 5759076 5761453 5763830 5766208
5768587 5770966 5773345 5775725 5778105 5780486 5782868 5785250 5787632 5790015
5792399 5794783 5797167 5799552 5801937 5804323 5806710 5809096 5811484 5813872
5816260 5818649 5821038 5823428 5825818 5828209 5830601 5832992 5835385 5837777
5840171 5842565 5844959 5847354 5849749 5852145 5854541 5856938 5859335 5861732
5864131 5866529 5868929 5871328 5873728 5876129 5878530 5880932 5883334 5885737
5888140 5890544 5892948 5895352 5897757 5900163 5902569 5904976 5907383 5909790
5912198 5914607 5917016 5919426 5921836 5924246 5926657 5929069 5931481 5933893
5936306 5938720 5941134 5943548 5945963 5948379 5950795 5953211 5955628 5958045
5960463 5962882 5965301 5967720 5970140 5972560 5974981 5977403 5979825 5982247
5984670 5987093 5989517 5991941 5994366 5996791 5999217 6001644 6004070 6006498
6008925 6011354 6013782 6016212 6018641 6021072 6023502 6025934 6028365 6030798
6033230 6035664 6038097 6040531 6042966 6045401 6047837 6050273 6052710 6055147
6057585 6060023 6062461 6064900 6067340 6069780 6072221 6074662 6077103 6079546
6081988 6084431 6086875 6089319 6091763 6094208 6096654 6099100 6101546 6103993
6106441 6108889 6111337 6113786 6116236 6118686 6121136 6123587 6126039 6128491
6130943 6133396 6135849 6138303 6140758 6143213 6145668 6148124 6150580 6153037
6155494 6157952 6160411 6162870 6165329 6167789 6170249 6172710 6175171 6177633
6180095 6182558 6185021 6187485 6189949 6192414 6194879 6197345 6199811 6202278
6204745 6207213 6209681 6212150 6214619 6217089 6219559 6222029 6224501 6226972
6229444 6231917 6234390 6236864 6239338 6241813 6244288 6246763 6249239 6251716
6254193 6256671 6259149 6261627 6264106 6266586 6269066 6271546 6274027 6276509
6278991 6281473 6283956 6286440 6288924 6291408 6293893 6296379 6298865 6301351
6303838 6306325 6308813 6311302 6313791 6316280 6318770 6321260 6323751 6326243
6328735 6331227 6333720 6336213 6338707 6341201 6343696 6346191 6348687 6351183
6353680 6356178 6358675 6361174 6363672 6366172 6368671 6371172 6373672 6376174
6378675 6381178 6383680 6386183 6388687 6391191 6393696 6396201 6398707 6401213
6403720 6406227 6408734 6411243 6413751 6416260 6418770 6421280 6423791 6426302
6428813 6431325 6433838 6436351 6438865 6441379 6443893 6446408 6448924 6451440
6453956 6456473 6458991 6461509 6464027 6466546 6469066 6471586 6474106 6476627
6479149 6481670 6484193 6486716 6489239 6491763 6494288 6496812 6499338 6501864
6504390 6506917 6509444 6511972 6514501 6517029 6519559 6522089 6524619 6527150
6529681 6532213 6534745 6537278 6539811 6542345 6544879 6547414 6549949 6552485
6555021 6557558 6560095 6562633 6565171 6567710 6570249 6572789 6575329 6577869
6580411 6582952 6585494 6588037 6590580 6593124 6595668 6598213 6600758 6603303
6605849 6608396 6610943 6613491 6616039 6618587 6621136 6623686 6626236 6628786
6631337 6633889 6636441 6638993 6641546 6644100 6646654 6649208 6651763 6654319
6656875 6659431 6661988 6664546 6667104 6669662 6672221 6674780 6677340 6679901
6682461 6685023 6687585 6690147 6692710 6695273 6697837 6700401 6702966 6705532

6708097 6710664 6713231 6715798 6718366 6720934 6723503 6726072 6728642 6731212
6733783 6736354 6738926 6741498 6744071 6746644 6749218 6751792 6754366 6756942
6759517 6762093 6764670 6767247 6769825 6772403 6774982 6777561 6780140 6782721
6785301 6787882 6790464 6793046 6795629 6798212 6800795 6803379 6805964 6808549
6811134 6813720 6816307 6818894 6821482 6824070 6826658 6829247 6831836 6834426
6837017 6839608 6842199 6844791 6847384 6849977 6852570 6855164 6857758 6860353
6862949 6865545 6868141 6870738 6873335 6875933 6878532 6881130 6883730 6886330
6888930 6891531 6894132 6896734 6899336 6901939 6904542 6907146 6909750 6912355
6914960 6917566 6920172 6922779 6925386 6927994 6930602 6933211 6935820 6938430
6941040 6943651 6946262 6948874 6951486 6954099 6956712 6959325 6961939 6964554
6967169 6969785 6972401 6975018 6977635 6980252 6982870 6985489 6988108 6990728
6993348 6995968 6998589 7001211 7003833 7006455 7009078 7011702 7014326 7016950
7019575 7022201 7024827 7027453 7030080 7032708 7035336 7037964 7040593 7043222
7045852 7048483 7051114 7053745 7056377 7059009 7061642 7064276 7066909 7069544
7072179 7074814 7077450 7080086 7082723 7085360 7087998 7090636 7093275 7095915
7098554 7101195 7103835 7106477 7109118 7111761 7114403 7117047 7119690 7122335
7124979 7127624 7130270 7132916 7135563 7138210 7140858 7143506 7146155 7148804
7151454 7154104 7156754 7159405 7162057 7164709 7167362 7170015 7172669 7175323
7177977 7180632 7183288 7185944 7188600 7191257 7193915 7196573 7199232 7201891
7204550 7207210 7209871 7212532 7215193 7217855 7220518 7223180 7225844 7228508
7231172 7233837 7236503 7239169 7241835 7244502 7247169 7249837 7252506 7255175
7257844 7260514 7263184 7265855 7268526 7271198 7273871 7276543 7279217 7281890
7284565 7287240 7289915 7292591 7295267 7297944 7300621 7303299 7305977 7308656
7311335 7314015 7316695 7319376 7322057 7324739 7327421 7330103 7332787 7335470
7338154 7340839 7343524 7346210 7348896 7351583 7354270 7356958 7359646 7362334
7365023 7367713 7370403 7373094 7375785 7378476 7381168 7383861 7386554 7389247
7391942 7394636 7397331 7400027 7402723 7405419 7408116 7410814 7413512 7416210
7418909 7421608 7424308 7427009 7429710 7432411 7435113 7437816 7440519 7443222
7445926 7448630 7451335 7454040 7456746 7459453 7462160 7464867 7467575 7470283
7472992 7475701 7478411 7481121 7483832 7486544 7489255 7491968 7494680 7497394
7500107 7502822 7505536 7508252 7510967 7513684 7516400 7519118 7521835 7524553
7527272 7529991 7532711 7535431 7538152 7540873 7543595 7546317 7549040 7551763
7554486 7557210 7559935 7562660 7565386 7568112 7570838 7573566 7576293 7579021
7581750 7584479 7587208 7589938 7592669 7595400 7598131 7600864 7603596 7606329
7609063 7611797 7614531 7617266 7620001 7622737 7625474 7628211 7630948 7633686
7636425 7639164 7641903 7644643 7647383 7650124 7652866 7655607 7658350 7661093
7663836 7666580 7669324 7672069 7674814 7677560 7680307 7683053 7685801 7688549
7691297 7694046 7696795 7699545 7702295 7705046 7707797 7710549 7713301 7716054
7718807 7721561 7724315 7727070 7729825 7732580 7735337 7738093 7740850 7743608
7746366 7749125 7751884 7754644 7757404 7760165 7762926 7765687 7768449 7771212
7773975 7776739 7779503 7782267 7785032 7787798 7790564 7793331 7796098 7798865
7801633 7804402 7807171 7809940 7812710 7815481 7818252 7821023 7823795 7826568
7829341 7832114 7834888 7837662 7840437 7843213 7845989 7848765 7851542 7854319
7857097 7859876 7862655 7865434 7868214 7870994 7873775 7876556 7879338 7882121
7884903 7887687 7890470 7893255 7896040 7898825 7901611 7904397 7907184 7909971
7912759 7915547 7918336 7921125 7923915 7926705 7929496 7932287 7935079 7937871
7940664 7943457 7946250 7949045 7951839 7954634 7957430 7960226 7963023 7965820
7968618 7971416 7974214 7977013 7979813 7982613 7985414 7988215 7991016 7993818

7996621 7999424 8002228 8005032 8007836 8010641 8013447 8016253 8019059 8021866
8024674 8027482 8030290 8033099 8035909 8038719 8041529 8044340 8047151 8049963
8052776 8055589 8058402 8061216 8064030 8066845 8069661 8072477 8075293 8078110
8080927 8083745 8086563 8089382 8092202 8095021 8097842 8100663 8103484 8106306
8109128 8111951 8114774 8117598 8120422 8123247 8126072 8128898 8131724 8134551
8137378 8140206 8143034 8145863 8148692 8151522 8154352 8157183 8160014 8162845
8165678 8168510 8171343 8174177 8177011 8179846 8182681 8185517 8188353 8191189
8194026 8196864 8199702 8202541 8205380 8208219 8211059 8213900 8216741 8219582
8222424 8225267 8228110 8230954 8233798 8236642 8239487 8242333 8245179 8248025
8250872 8253719 8256567 8259416 8262265 8265114 8267964 8270815 8273665 8276517
8279369 8282221 8285074 8287927 8290781 8293636 8296491 8299346 8302202 8305058
8307915 8310772 8313630 8316488 8319347 8322206 8325066 8327927 8330787 8333649
8336510 8339373 8342235 8345099 8347962 8350827 8353691 8356557 8359422 8362289
8365155 8368022 8370890 8373758 8376627 8379496 8382366 8385236 8388107 8390978
8393849 8396722 8399594 8402467 8405341 8408215 8411090 8413965 8416840 8419716
8422593 8425470 8428348 8431226 8434104 8436983 8439863 8442743 8445623 8448504
8451386 8454268 8457150 8460033 8462917 8465801 8468685 8471570 8474456 8477342
8480228 8483115 8486002 8488890 8491779 8494668 8497557 8500447 8503337 8506228
8509120 8512011 8514904 8517797 8520690 8523584 8526478 8529373 8532268 8535164
8538060 8540957 8543855 8546752 8549651 8552549 8555449 8558348 8561249 8564149
8567051 8569952 8572855 8575757 8578661 8581564 8584469 8587373 8590278 8593184
8596090 8598997 8601904 8604812 8607720 8610629 8613538 8616447 8619357 8622268
8625179 8628091 8631003 8633916 8636829 8639742 8642656 8645571 8648486 8651401
8654318 8657234 8660151 8663069 8665987 8668905 8671824 8674744 8677664 8680584
8683505 8686427 8689349 8692271 8695194 8698117 8701041 8703966 8706891 8709816
8712742 8715669 8718595 8721523 8724451 8727379 8730308 8733237 8736167 8739098
8742029 8744960 8747892 8750824 8753757 8756690 8759624 8762558 8765493 8768428
8771364 8774300 8777237 8780175 8783112 8786051 8788989 8791929 8794868 8797809
8800749 8803690 8806632 8809574 8812517 8815460 8818404 8821348 8824293 8827238
8830184 8833130 8836076 8839024 8841971 8844919 8847868 8850817 8853767 8856717
8859667 8862618 8865570 8868522 8871475 8874428 8877381 8880335 8883290 8886245
8889200 8892157 8895113 8898070 8901028 8903986 8906944 8909903 8912862 8915822
8918783 8921744 8924705 8927667 8930630 8933593 8936556 8939520 8942484 8945449
8948415 8951381 8954347 8957314 8960281 8963249 8966218 8969186 8972156 8975126
8978096 8981067 8984038 8987010 8989982 8992955 8995928 8998902 9001876 9004851
9007826 9010802 9013778 9016755 9019732 9022710 9025688 9028667 9031646 9034626
9037606 9040587 9043568 9046550 9049532 9052515 9055498 9058482 9061466 9064450
9067436 9070421 9073407 9076394 9079381 9082369 9085357 9088345 9091334 9094324
9097314 9100305 9103296 9106287 9109279 9112272 9115265 9118258 9121253 9124247
9127242 9130238 9133234 9136230 9139227 9142225 9145223 9148221 9151220 9154219
9157219 9160220 9163221 9166222 9169224 9172227 9175229 9178233 9181237 9184241
9187246 9190251 9193257 9196264 9199271 9202278 9205286 9208294 9211303 9214312
9217322 9220332 9223343 9226354 9229366 9232379 9235391 9238405 9241418 9244433
9247447 9250463 9253478 9256495 9259511 9262529 9265546 9268565 9271583 9274602
9277622 9280642 9283663 9286684 9289706 9292728 9295751 9298774 9301797 9304822
9307846 9310871 9313897 9316923 9319950 9322977 9326004 9329032 9332061 9335090
9338120 9341150 9344180 9347211 9350243 9353275 9356307 9359340 9362374 9365408
9368443 9371478 9374513 9377549 9380585 9383622 9386660 9389698 9392736 9395775

9398815 9401855 9404895 9407936 9410977 9414019 9417062 9420105 9423148 9426192
9429236 9432281 9435326 9438372 9441419 9444466 9447513 9450561 9453609 9456658
9459707 9462757 9465807 9468858 9471909 9474961 9478013 9481066 9484119 9487173
9490227 9493282 9496337 9499393 9502449 9505506 9508563 9511621 9514679 9517738
9520797 9523857 9526917 9529978 9533039 9536100 9539163 9542225 9545288 9548352
9551416 9554481 9557546 9560611 9563677 9566744 9569811 9572879 9575947 9579015
9582084 9585154 9588224 9591295 9594366 9597437 9600509 9603582 9606655 9609728
9612802 9615877 9618952 9622027 9625103 9628180 9631256 9634334 9637412 9640490
9643569 9646649 9649729 9652809 9655890 9658971 9662053 9665136 9668218 9671302
9674386 9677470 9680555 9683640 9686726 9689812 9692899 9695987 9699074 9702163
9705252 9708341 9711431 9714521 9717612 9720703 9723795 9726887 9729980 9733073
9736167 9739261 9742356 9745451 9748547 9751643 9754740 9757837 9760934 9764033
9767131 9770230 9773330 9776430 9779531 9782632 9785734 9788836 9791938 9795042
9798145 9801249 9804354 9807459 9810565 9813671 9816777 9819884 9822992 9826100
9829209 9832318 9835427 9838537 9841648 9844759 9847870 9850982 9854095 9857208
9860321 9863435 9866550 9869665 9872780 9875896 9879012 9882129 9885247 9888365
9891483 9894602 9897722 9900841 9903962 9907083 9910204 9913326 9916448 9919571
9922695 9925818 9928943 9932068 9935193 9938319 9941445 9944572 9947699 9950827
9953955 9957084 9960213 9963343 9966474 9969604 9972736 9975867 9979000 9982132
9985266 9988399 9991534 9994668 9997803 10000939 10004075 10007212 10010349
10013487 10016625 10019764 10022903 10026043 10029183 10032323 10035465 10038606
10041748 10044891 10048034 10051178 10054322 10057466 10060611 10063757 10066903
10070050 10073197 10076344 10079492 10082641 10085790 10088939 10092089 10095240
10098391 10101542 10104694 10107847 10111000 10114153 10117307 10120462 10123617
10126772 10129928 10133085 10136242 10139399 10142557 10145715 10148874 10152034
10155194 10158354 10161515 10164676 10167838 10171000 10174163 10177327 10180491
10183655 10186820 10189985 10193151 10196317 10199484 10202651 10205819 10208987
10212156 10215325 10218495 10221665 10224836 10228007 10231179 10234351 10237524
10240697 10243871 10247045 10250220 10253395 10256571 10259747 10262924 10266101
10269279 10272457 10275636 10278815 10281994 10285174 10288355 10291536 10294718
10297900 10301083 10304266 10307449 10310633 10313818 10317003 10320189 10323375
10326561 10329748 10332936 10336124 10339312 10342501 10345691 10348881 10352072
10355263 10358454 10361646 10364839 10368032 10371225 10374419 10377613 10380808
10384004 10387200 10390396 10393593 10396791 10399988 10403187 10406386 10409585
10412785 10415985 10419186 10422388 10425590 10428792 10431995 10435198 10438402
10441606 10444811 10448016 10451222 10454429 10457635 10460843 10464051 10467259
10470468 10473677 10476887 10480097 10483308 10486519 10489731 10492943 10496156
10499369 10502583 10505797 10509011 10512227 10515442 10518659 10521875 10525092
10528310 10531528 10534747 10537966 10541186 10544406 10547626 10550848 10554069
10557291 10560514 10563737 10566961 10570185 10573409 10576634 10579860 10583086
10586313 10589540 10592767 10595995 10599224 10602453 10605682 10608912 10612143
10615374 10618605 10621837 10625070 10628303 10631536 10634770 10638005 10641240
10644475 10647711 10650947 10654184 10657422 10660660 10663898 10667137 10670376
10673616 10676857 10680098 10683339 10686581 10689823 10693066 10696309 10699553
10702798 10706042 10709288 10712534 10715780 10719027 10722274 10725522 10728770
10732019 10735268 10738518 10741768 10745019 10748270 10751522 10754774 10758027
10761280 10764534 10767788 10771043 10774298 10777554 10780810 10784066 10787324
10790581 10793839 10797098 10800357 10803617 10806877 10810138 10813399 10816660

10819923 10823185 10826448 10829712 10832976 10836241 10839506 10842771 10846037
10849304 10852571 10855838 10859106 10862375 10865644 10868913 10872184 10875454
10878725 10881997 10885269 10888541 10891814 10895087 10898361 10901636 10904911
10908186 10911462 10914739 10918016 10921293 10924571 10927849 10931128 10934408
10937688 10940968 10944249 10947530 10950812 10954094 10957377 10960661 10963944
10967229 10970514 10973799 10977085 10980371 10983658 10986945 10990233 10993521
10996810 11000099 11003389 11006679 11009970 11013261 11016553 11019845 11023138
11026431 11029725 11033019 11036314 11039609 11042905 11046201 11049498 11052795
11056093 11059391 11062689 11065989 11069288 11072588 11075889 11079190 11082492
11085794 11089096 11092400 11095703 11099007 11102312 11105617 11108922 11112229
11115535 11118842 11122150 11125458 11128766 11132075 11135385 11138695 11142005
11145316 11148628 11151940 11155252 11158565 11161879 11165193 11168507 11171822
11175138 11178454 11181770 11185087 11188404 11191722 11195041 11198360 11201679
11204999 11208319 11211640 11214962 11218283 11221606 11224929 11228252 11231576
11234900 11238225 11241550 11244876 11248203 11251529 11254857 11258184 11261513
11264842 11268171 11271501 11274831 11278162 11281493 11284825 11288157 11291490
11294823 11298157 11301491 11304825 11308161 11311496 11314833 11318169 11321506
11324844 11328182 11331521 11334860 11338200 11341540 11344881 11348222 11351563
11354905 11358248 11361591 11364935 11368279 11371623 11374969 11378314 11381660
11385007 11388354 11391701 11395049 11398398 11401747 11405097 11408447 11411797
11415148 11418500 11421852 11425204 11428557 11431911 11435265 11438619 11441974
11445329 11448685 11452042 11455399 11458756 11462114 11465473 11468831 11472191
11475551 11478911 11482272 11485633 11488995 11492358 11495721 11499084 11502448
11505812 11509177 11512542 11515908 11519274 11522641 11526009 11529376 11532745
11536113 11539483 11542853 11546223 11549594 11552965 11556337 11559709 11563082
11566455 11569829 11573203 11576578 11579953 11583328 11586705 11590081 11593459
11596836 11600214 11603593 11606972 11610352 11613732 11617113 11620494 11623876
11627258 11630640 11634023 11637407 11640791 11644176 11647561 11650946 11654332
11657719 11661106 11664494 11667882 11671270 11674659 11678049 11681439 11684829
11688220 11691612 11695004 11698396 11701789 11705183 11708577 11711971 11715366
11718762 11722158 11725554 11728951 11732349 11735747 11739145 11742544 11745943
11749343 11752744 11756145 11759546 11762948 11766350 11769753 11773157 11776560
11779965 11783370 11786775 11790181 11793587 11796994 11800401 11803809 11807217
11810626 11814036 11817445 11820856 11824266 11827678 11831089 11834502 11837914
11841328 11844741 11848156 11851570 11854986 11858401 11861818 11865234 11868651
11872069 11875487 11878906 11882325 11885745 11889165 11892586 11896007 11899429
11902851 11906273 11909696 11913120 11916544 11919969 11923394 11926819 11930246
11933672 11937099 11940527 11943955 11947383 11950813 11954242 11957672 11961103
11964534 11967965 11971397 11974830 11978263 11981696 11985130 11988565 11992000
11995435 11998871 12002308 12005745 12009182 12012620 12016058 12019497 12022937
12026377 12029817 12033258 12036699 12040141 12043584 12047027 12050470 12053914
12057358 12060803 12064249 12067694 12071141 12074588 12078035 12081483 12084931
12088380 12091829 12095279 12098729 12102180 12105631 12109083 12112535 12115988
12119441 12122895 12126349 12129804 12133259 12136715 12140171 12143628 12147085
12150543 12154001 12157460 12160919 12164379 12167839 12171300 12174761 12178223
12181685 12185147 12188610 12192074 12195538 12199003 12202468 12205934 12209400
12212866 12216333 12219801 12223269 12226738 12230207 12233676 12237146 12240617
12244088 12247560 12251032 12254504 12257977 12261451 12264925 12268399 12271874

12275350 12278826 12282302 12285779 12289257 12292735 12296213 12299692 12303171
12306651 12310132 12313613 12317094 12320576 12324058 12327541 12331025 12334509
12337993 12341478 12344963 12348449 12351936 12355422 12358910 12362398 12365886
12369375 12372864 12376354 12379844 12383335 12386826 12390318 12393810 12397303
12400796 12404290 12407785 12411279 12414775 12418270 12421767 12425263 12428761
12432258 12435756 12439255 12442754 12446254 12449754 12453255 12456756 12460258
12463760 12467263 12470766 12474270 12477774 12481278 12484783 12488289 12491795
12495302 12498809 12502317 12505825 12509333 12512842 12516352 12519862 12523373
12526884 12530395 12533907 12537420 12540933 12544446 12547960 12551475 12554990
12558505 12562021 12565538 12569055 12572572 12576090 12579609 12583128 12586647
12590167 12593688 12597209 12600730 12604252 12607774 12611297 12614821 12618345
12621869 12625394 12628919 12632445 12635972 12639499 12643026 12646554 12650082
12653611 12657140 12660670 12664201 12667731 12671263 12674795 12678327 12681860
12685393 12688927 12692461 12695996 12699531 12703067 12706603 12710140 12713677
12717215 12720753 12724292 12727831 12731371 12734911 12738452 12741993 12745535
12749077 12752620 12756163 12759706 12763251 12766795 12770340 12773886 12777432
12780979 12784526 12788074 12791622 12795170 12798719 12802269 12805819 12809370
12812921 12816472 12820024 12823577 12827130 12830684 12834238 12837792 12841347
12844903 12848459 12852015 12855572 12859130 12862688 12866246 12869805 12873365
12876925 12880485 12884046 12887608 12891170 12894732 12898295 12901859 12905422
12908987 12912552 12916117 12919683 12923250 12926816 12930384 12933952 12937520
12941089 12944658 12948228 12951799 12955369 12958941 12962513 12966085 12969658
12973231 12976805 12980379 12983954 12987529 12991105 12994681 12998258 13001835
13005413 13008991 13012570 13016149 13019729 13023309 13026890 13030471 13034053
13037635 13041218 13044801 13048385 13051969 13055554 13059139 13062725 13066311
13069898 13073485 13077072 13080660 13084249 13087838 13091428 13095018 13098609
13102200 13105791 13109383 13112976 13116569 13120163 13123757 13127351 13130946
13134542 13138138 13141735 13145332 13148929 13152527 13156126 13159725 13163324
13166924 13170525 13174126 13177727 13181329 13184932 13188535 13192138 13195742
13199346 13202951 13206557 13210163 13213769 13217376 13220984 13224591 13228200
13231809 13235418 13239028 13242638 13246249 13249861 13253473 13257085 13260698
13264311 13267925 13271539 13275154 13278769 13282385 13286002 13289618 13293236
13296853 13300472 13304090 13307710 13311330 13314950 13318571 13322192 13325814
13329436 13333059 13336682 13340305 13343930 13347554 13351180 13354805 13358431
13362058 13365685 13369313 13372941 13376570 13380199 13383829 13387459 13391089
13394721 13398352 13401984 13405617 13409250 13412884 13416518 13420152 13423787
13427423 13431059 13434696 13438333 13441970 13445608 13449247 13452886 13456525
13460166 13463806 13467447 13471089 13474731 13478373 13482016 13485660 13489304
13492948 13496593 13500238 13503884 13507531 13511178 13514825 13518473 13522122
13525771 13529420 13533070 13536720 13540371 13544023 13547674 13551327 13554980
13558633 13562287 13565941 13569596 13573252 13576907 13580564 13584221 13587878
13591536 13595194 13598853 13602512 13606172 13609832 13613493 13617154 13620816
13624478 13628141 13631804 13635468 13639132 13642797 13646462 13650128 13653794
13657461 13661128 13664796 13668464 13672133 13675802 13679472 13683142 13686813
13690484 13694155 13697827 13701500 13705173 13708847 13712521 13716196 13719871
13723546 13727222 13730899 13734576 13738254 13741932 13745610 13749289 13752969
13756649 13760330 13764011 13767692 13771374 13775057 13778740 13782423 13786107
13789792 13793477 13797162 13800848 13804535 13808222 13811909 13815597 13819286

13822975 13826664 13830354 13834045 13837735 13841427 13845119 13848811 13852504
13856198 13859891 13863586 13867281 13870976 13874672 13878368 13882065 13885763
13889460 13893159 13896858 13900557 13904257 13907957 13911658 13915359 13919061
13922763 13926466 13930169 13933873 13937577 13941282 13944987 13948693 13952399
13956106 13959813 13963521 13967229 13970938 13974647 13978357 13982067 13985778
13989489 13993201 13996913 14000626 14004339 14008053 14011767 14015481 14019196
14022912 14026628 14030345 14034062 14037780 14041498 14045216 14048935 14052655
14056375 14060096 14063817 14067538 14071260 14074983 14078706 14082430 14086154
14089878 14093603 14097329 14101055 14104781 14108508 14112236 14115964 14119692
14123421 14127151 14130881 14134611 14138342 14142074 14145806 14149538 14153271
14157004 14160738 14164473 14168208 14171943 14175679 14179415 14183152 14186890
14190628 14194366 14198105 14201844 14205584 14209324 14213065 14216807 14220548
14224291 14228034 14231777 14235521 14239265 14243010 14246755 14250501 14254247
14257994 14261741 14265489 14269237 14272986 14276735 14280485 14284235 14287986
14291737 14295489 14299241 14302994 14306747 14310501 14314255 14318010 14321765
14325520 14329277 14333033 14336790 14340548 14344306 14348065 14351824 14355584
14359344 14363104 14366866 14370627 14374389 14378152 14381915 14385678 14389443
14393207 14396972 14400738 14404504 14408270 14412037 14415805 14419573 14423342
14427111 14430880 14434650 14438421 14442192 14445963 14449735 14453508 14457280
14461054 14464828 14468602 14472377 14476153 14479929 14483705 14487482 14491259
14495037 14498816 14502595 14506374 14510154 14513934 14517715 14521497 14525279
14529061 14532844 14536627 14540411 14544195 14547980 14551765 14555551 14559338
14563124 14566912 14570700 14574488 14578277 14582066 14585856 14589646 14593437
14597228 14601020 14604812 14608605 14612398 14616192 14619986 14623781 14627576
14631372 14635168 14638965 14642762 14646559 14650358 14654156 14657955 14661755
14665555 14669356 14673157 14676959 14680761 14684563 14688367 14692170 14695974
14699779 14703584 14707390 14711196 14715002 14718809 14722617 14726425 14730234
14734043 14737852 14741662 14745473 14749284 14753095 14756907 14760720 14764533
14768346 14772160 14775975 14779790 14783605 14787421 14791238 14795055 14798872
14802690 14806509 14810328 14814147 14817967 14821787 14825608 14829430 14833252
14837074 14840897 14844720 14848544 14852369 14856193 14860019 14863845 14867671
14871498 14875325 14879153 14882981 14886810 14890640 14894469 14898300 14902131
14905962 14909794 14913626 14917459 14921292 14925126 14928960 14932795 14936630
14940466 14944302 14948139 14951976 14955814 14959652 14963491 14967330 14971170
14975010 14978850 14982692 14986533 14990376 14994218 14998061 15001905 15005749
15009594 15013439 15017285 15021131 15024977 15028824 15032672 15036520 15040369
15044218 15048067 15051918 15055768 15059619 15063471 15067323 15071175 15075028
15078882 15082736 15086591 15090446 15094301 15098157 15102014 15105871 15109728
15113586 15117445 15121304 15125163 15129023 15132884 15136744 15140606 15144468
15148330 15152193 15156057 15159921 15163785 15167650 15171515 15175381 15179248
15183115 15186982 15190850 15194718 15198587 15202456 15206326 15210197 15214067
15217939 15221811 15225683 15229556 15233429 15237303 15241177 15245052 15248927
15252803 15256679 15260556 15264433 15268311 15272190 15276068 15279948 15283827
15287708 15291588 15295469 15299351 15303233 15307116 15310999 15314883 15318767
15322652 15326537 15330423 15334309 15338196 15342083 15345970 15349859 15353747
15357636 15361526 15365416 15369307 15373198 15377089 15380982 15384874 15388767
15392661 15396555 15400450 15404345 15408240 15412136 15416033 15419930 15423827
15427725 15431624 15435523 15439422 15443322 15447223 15451124 15455025 15458927

15462830 15466733 15470636 15474540 15478445 15482350 15486255 15490161 15494068
15497975 15501882 15505790 15509698 15513607 15517517 15521427 15525337 15529248
15533159 15537071 15540983 15544896 15548810 15552724 15556638 15560553 15564468
15568384 15572300 15576217 15580134 15584052 15587971 15591889 15595809 15599728
15603649 15607569 15611491 15615412 15619335 15623257 15627181 15631104 15635029
15638953 15642879 15646804 15650731 15654657 15658585 15662512 15666440 15670369
15674298 15678228 15682158 15686089 15690020 15693952 15697884 15701816 15705750
15709683 15713617 15717552 15721487 15725423 15729359 15733295 15737232 15741170
15745108 15749047 15752986 15756925 15760865 15764806 15768747 15772689 15776631
15780573 15784516 15788460 15792404 15796348 15800293 15804239 15808185 15812131
15816078 15820026 15823974 15827922 15831871 15835820 15839770 15843721 15847672
15851623 15855575 15859528 15863480 15867434 15871388 15875342 15879297 15883252
15887208 15891165 15895122 15899079 15903037 15906995 15910954 15914913 15918873
15922833 15926794 15930756 15934717 15938680 15942642 15946606 15950570 15954534
15958499 15962464 15966430 15970396 15974363 15978330 15982298 15986266 15990235
15994204 15998174 16002144 16006115 16010086 16014057 16018030 16022002 16025976
16029949 16033923 16037898 16041873 16045849 16049825 16053802 16057779 16061756
16065735 16069713 16073692 16077672 16081652 16085633 16089614 16093595 16097577
16101560 16105543 16109527 16113511 16117495 16121480 16125466 16129452 16133438
16137425 16141413 16145401 16149389 16153378 16157368 16161358 16165348 16169339
16173331 16177323 16181315 16185308 16189302 16193296 16197290 16201285 16205280
16209276 16213273 16217270 16221267 16225265 16229263 16233262 16237262 16241262
16245262 16249263 16253264 16257266 16261268 16265271 16269275 16273278 16277283
16281288 16285293 16289299 16293305 16297312 16301319 16305327 16309335 16313344
16317353 16321363 16325373 16329384 16333395 16337407 16341419 16345432 16349445
16353459 16357473 16361488 16365503 16369519 16373535 16377552 16381569 16385587
16389605 16393623 16397643 16401662 16405682 16409703 16413724 16417746 16421768
16425791 16429814 16433837 16437861 16441886 16445911 16449937 16453963 16457989
16462017 16466044 16470072 16474101 16478130 16482159 16486189 16490220 16494251
16498282 16502314 16506347 16510380 16514414 16518448 16522482 16526517 16530552
16534588 16538625 16542662 16546699 16550737 16554776 16558815 16562854 16566894
16570934 16574975 16579017 16583059 16587101 16591144 16595187 16599231 16603276
16607321 16611366 16615412 16619458 16623505 16627552 16631600 16635649 16639697
16643747 16647797 16651847 16655898 16659949 16664001 16668053 16672106 16676159
16680213 16684267 16688322 16692377 16696433 16700489 16704546 16708603 16712661
16716719 16720778 16724837 16728897 16732957 16737018 16741079 16745141 16749203
16753265 16757329 16761392 16765456 16769521 16773586 16777652 16781718 16785785
16789852 16793919 16797988 16802056 16806125 16810195 16814265 16818336 16822407
16826478 16830550 16834623 16838696 16842769 16846844 16850918 16854993 16859069
16863145 16867221 16871298 16875376 16879454 16883532 16887611 16891691 16895771
16899851 16903932 16908014 16912096 16916178 16920261 16924344 16928428 16932513
16936598 16940683 16944769 16948855 16952942 16957030 16961118 16965206 16969295
16973384 16977474 16981565 16985655 16989747 16993839 16997931 17002024 17006117
17010211 17014305 17018400 17022495 17026591 17030688 17034784 17038882 17042979
17047078 17051176 17055276 17059376 17063476 17067577 17071678 17075780 17079882
17083984 17088088 17092191 17096296 17100400 17104506 17108611 17112717 17116824
17120931 17125039 17129147 17133256 17137365 17141475 17145585 17149695 17153807
17157918 17162030 17166143 17170256 17174370 17178484 17182598 17186713 17190829

17194945 17199062 17203179 17207296 17211414 17215533 17219652 17223772 17227892
17232012 17236133 17240255 17244377 17248499 17252622 17256746 17260870 17264994
17269119 17273245 17277371 17281497 17285624 17289752 17293879 17298008 17302137
17306266 17310396 17314527 17318658 17322789 17326921 17331053 17335186 17339320
17343453 17347588 17351723 17355858 17359994 17364130 17368267 17372405 17376542
17380681 17384820 17388959 17393099 17397239 17401380 17405521 17409663 17413805
17417948 17422091 17426235 17430379 17434524 17438669 17442815 17446961 17451108
17455255 17459403 17463551 17467700 17471849 17475999 17480149 17484300 17488451
17492603 17496755 17500908 17505061 17509214 17513369 17517523 17521678 17525834
17529990 17534147 17538304 17542462 17546620 17550778 17554937 17559097 17563257
17567418 17571579 17575740 17579902 17584065 17588228 17592392 17596556 17600720
17604885 17609051 17613217 17617383 17621550 17625718 17629886 17634054 17638223
17642393 17646563 17650733 17654904 17659076 17663248 17667420 17671593 17675767
17679941 17684115 17688290 17692465 17696641 17700818 17704995 17709172 17713350
17717528 17721707 17725887 17730066 17734247 17738428 17742609 17746791 17750973
17755156 17759339 17763523 17767708 17771892 17776078 17780263 17784450 17788637
17792824 17797012 17801200 17805389 17809578 17813768 17817958 17822149 17826340
17830532 17834724 17838917 17843110 17847304 17851498 17855693 17859888 17864084
17868280 17872476 17876674 17880871 17885070 17889268 17893467 17897667 17901867
17906068 17910269 17914471 17918673 17922875 17927078 17931282 17935486 17939691
17943896 17948101 17952308 17956514 17960721 17964929 17969137 17973345 17977554
17981764 17985974 17990185 17994396 17998607 18002819 18007032 18011245 18015458
18019672 18023887 18028102 18032317 18036533 18040750 18044967 18049184 18053402
18057621 18061839 18066059 18070279 18074499 18078720 18082942 18087164 18091386
18095609 18099832 18104056 18108281 18112505 18116731 18120957 18125183 18129410
18133637 18137865 18142093 18146322 18150552 18154781 18159012 18163243 18167474
18171706 18175938 18180171 18184404 18188638 18192872 18197107 18201342 18205578
18209814 18214051 18218288 18222526 18226764 18231003 18235242 18239482 18243722
18247963 18252204 18256446 18260688 18264931 18269174 18273417 18277662 18281906
18286151 18290397 18294643 18298890 18303137 18307385 18311633 18315881 18320131
18324380 18328630 18332881 18337132 18341384 18345636 18349888 18354141 18358395
18362649 18366903 18371159 18375414 18379670 18383927 18388184 18392441 18396699
18400958 18405217 18409476 18413736 18417997 18422258 18426519 18430781 18435044
18439307 18443570 18447834 18452099 18456363 18460629 18464895 18469161 18473428
18477696 18481964 18486232 18490501 18494770 18499040 18503310 18507581 18511853
18516125 18520397 18524670 18528943 18533217 18537491 18541766 18546042 18550317
18554594 18558871 18563148 18567426 18571704 18575983 18580262 18584542 18588822
18593103 18597384 18601666 18605948 18610231 18614514 18618798 18623082 18627367
18631652 18635938 18640224 18644511 18648798 18653086 18657374 18661662 18665952
18670241 18674531 18678822 18683113 18687405 18691697 18695990 18700283 18704577
18708871 18713165 18717460 18721756 18726052 18730349 18734646 18738943 18743241
18747540 18751839 18756139 18760439 18764739 18769040 18773342 18777644 18781946
18786250 18790553 18794857 18799162 18803467 18807772 18812078 18816385 18820692
18824999 18829307 18833615 18837924 18842234 18846544 18850854 18855165 18859477
18863789 18868101 18872414 18876727 18881041 18885356 18889671 18893986 18898302
18902618 18906935 18911253 18915570 18919889 18924208 18928527 18932847 18937167
18941488 18945809 18950131 18954453 18958776 18963100 18967423 18971748 18976073
18980398 18984724 18989050 18993377 18997704 19002032 19006360 19010689 19015018

19019348 19023678 19028009 19032340 19036672 19041004 19045336 19049670 19054003
19058338 19062672 19067007 19071343 19075679 19080016 19084353 19088691 19093029
19097368 19101707 19106046 19110386 19114727 19119068 19123410 19127752 19132095
19136438 19140781 19145125 19149470 19153815 19158161 19162507 19166853 19171200
19175548 19179896 19184244 19188593 19192943 19197293 19201643 19205994 19210346
19214698 19219050 19223403 19227757 19232111 19236465 19240820 19245176 19249532
19253888 19258245 19262603 19266961 19271319 19275678 19280037 19284397 19288758
19293119 19297480 19301842 19306204 19310567 19314930 19319294 19323659 19328024
19332389 19336755 19341121 19345488 19349855 19354223 19358591 19362960 19367330
19371699 19376070 19380441 19384812 19389184 19393556 19397929 19402302 19406676
19411050 19415425 19419800 19424176 19428552 19432929 19437306 19441684 19446062
19450441 19454820 19459199 19463580 19467960 19472342 19476723 19481105 19485488
19489871 19494255 19498639 19503024 19507409 19511794 19516181 19520567 19524954
19529342 19533730 19538119 19542508 19546897 19551287 19555678 19560069 19564461
19568853 19573245 19577638 19582032 19586426 19590821 19595216 19599611 19604007
19608404 19612801 19617198 19621596 19625995 19630394 19634793 19639193 19643594
19647995 19652396 19656798 19661200 19665603 19670007 19674411 19678815 19683220
19687626 19692031 19696438 19700845 19705252 19709660 19714069 19718477 19722887
19727297 19731707 19736118 19740529 19744941 19749353 19753766 19758180 19762593
19767008 19771423 19775838 19780254 19784670 19789087 19793504 19797922 19802340
19806759 19811178 19815598 19820018 19824439 19828860 19833282 19837704 19842127
19846550 19850974 19855398 19859823 19864248 19868674 19873100 19877527 19881954
19886382 19890810 19895239 19899668 19904097 19908528 19912958 19917389 19921821
19926253 19930686 19935119 19939553 19943987 19948421 19952856 19957292 19961728
19966165 19970602 19975039 19979477 19983916 19988355 19992795 19997235 20001675
20006116 20010558 20015000 20019442 20023885 20028329 20032773 20037217 20041662
20046108 20050554 20055000 20059447 20063895 20068343 20072791 20077240 20081690
20086140 20090590 20095041 20099492 20103944 20108397 20112850 20117303 20121757
20126211 20130666 20135122 20139578 20144034 20148491 20152948 20157406 20161864
20166323 20170783 20175242 20179703 20184164 20188625 20193087 20197549 20202012
20206475 20210939 20215403 20219868 20224333 20228799 20233265 20237732 20242199
20246667 20251135 20255604 20260073 20264543 20269013 20273484 20277955 20282427
20286899 20291372 20295845 20300319 20304793 20309268 20313743 20318219 20322695
20327171 20331649 20336126 20340604 20345083 20349562 20354042 20358522 20363003
20367484 20371965 20376447 20380930 20385413 20389897 20394381 20398865 20403350
20407836 20412322 20416809 20421296 20425783 20430271 20434760 20439249 20443738
20448228 20452719 20457210 20461701 20466193 20470686 20475179 20479672 20484166
20488661 20493156 20497651 20502147 20506644 20511141 20515638 20520136 20524634
20529133 20533633 20538133 20542633 20547134 20551635 20556137 20560640 20565143
20569646 20574150 20578654 20583159 20587664 20592170 20596677 20601184 20605691
20610199 20614707 20619216 20623725 20628235 20632745 20637256 20641767 20646279
20650791 20655304 20659817 20664331 20668846 20673360 20677876 20682391 20686908
20691424 20695942 20700459 20704977 20709496 20714015 20718535 20723055 20727576
20732097 20736619 20741141 20745664 20750187 20754711 20759235 20763759 20768285
20772810 20777336 20781863 20786390 20790918 20795446 20799974 20804504 20809033
20813563 20818094 20822625 20827156 20831689 20836221 20840754 20845288 20849822
20854356 20858891 20863427 20867963 20872499 20877037 20881574 20886112 20890651
20895190 20899729 20904269 20908810 20913351 20917892 20922434 20926976 20931519

20936063 20940607 20945151 20949696 20954242 20958788 20963334 20967881 20972428
20976976 20981525 20986073 20990623 20995173 20999723 21004274 21008825 21013377
21017930 21022482 21027036 21031590 21036144 21040699 21045254 21049810 21054366
21058923 21063480 21068038 21072597 21077155 21081715 21086274 21090835 21095395
21099957 21104518 21109081 21113644 21118207 21122770 21127335 21131899 21136465
21141030 21145597 21150163 21154731 21159298 21163866 21168435 21173004 21177574
21182144 21186715 21191286 21195858 21200430 21205002 21209576 21214149 21218723
21223298 21227873 21232449 21237025 21241601 21246179 21250756 21255334 21259913
21264492 21269071 21273652 21278232 21282813 21287395 21291977 21296559 21301142
21305726 21310310 21314894 21319479 21324065 21328651 21333237 21337824 21342412
21347000 21351588 21356177 21360767 21365357 21369947 21374538 21379130 21383722
21388314 21392907 21397500 21402094 21406689 21411284 21415879 21420475 21425071
21429668 21434266 21438864 21443462 21448061 21452660 21457260 21461860 21466461
21471063 21475664 21480267 21484870 21489473 21494077 21498681 21503286 21507891
21512497 21517103 21521710 21526317 21530925 21535533 21540142 21544751 21549361
21553971 21558582 21563193 21567805 21572417 21577030 21581643 21586257 21590871
21595486 21600101 21604717 21609333 21613950 21618567 21623185 21627803 21632421
21637041 21641660 21646280 21650901 21655522 21660144 21664766 21669389 21674012
21678635 21683259 21687884 21692509 21697135 21701761 21706387 21711014 21715642
21720270 21724899 21729528 21734157 21738787 21743418 21748049 21752680 21757312
21761945 21766578 21771211 21775845 21780480 21785115 21789750 21794386 21799023
21803660 21808297 21812935 21817574 21822213 21826852 21831492 21836132 21840773
21845415 21850057 21854699 21859342 21863985 21868629 21873274 21877919 21882564
21887210 21891856 21896503 21901151 21905798 21910447 21915096 21919745 21924395
21929045 21933696 21938347 21942999 21947651 21952304 21956957 21961611 21966266
21970920 21975576 21980231 21984888 21989544 21994202 21998859 22003518 22008176
22012836 22017495 22022156 22026816 22031478 22036139 22040802 22045464 22050127
22054791 22059455 22064120 22068785 22073451 22078117 22082784 22087451 22092118
22096787 22101455 22106124 22110794 22115464 22120135 22124806 22129477 22134150
22138822 22143495 22148169 22152843 22157518 22162193 22166868 22171544 22176221
22180898 22185575 22190253 22194932 22199611 22204291 22208971 22213651 22218332
22223014 22227696 22232378 22237061 22241745 22246429 22251113 22255798 22260483
22265169 22269856 22274543 22279230 22283918 22288607 22293296 22297985 22302675
22307365 22312056 22316748 22321440 22326132 22330825 22335518 22340212 22344906
22349601 22354297 22358993 22363689 22368386 22373083 22377781 22382479 22387178
22391877 22396577 22401278 22405978 22410680 22415381 22420084 22424787 22429490
22434194 22438898 22443603 22448308 22453014 22457720 22462427 22467134 22471842
22476550 22481259 22485968 22490678 22495388 22500098 22504810 22509521 22514234
22518946 22523659 22528373 22533087 22537802 22542517 22547233 22551949 22556665
22561383 22566100 22570818 22575537 22580256 22584976 22589696 22594416 22599138
22603859 22608581 22613304 22618027 22622750 22627474 22632199 22636924 22641650
22646376 22651102 22655829 22660557 22665285 22670013 22674742 22679472 22684202
22688932 22693663 22698395 22703127 22707859 22712592 22717326 22722060 22726794
22731529 22736265 22741001 22745737 22750474 22755211 22759949 22764688 22769427
22774166 22778906 22783646 22788387 22793129 22797870 22802613 22807356 22812099
22816843 22821587 22826332 22831077 22835823 22840570 22845316 22850064 22854812
22859560 22864309 22869058 22873808 22878558 22883309 22888060 22892812 22897564
22902317 22907070 22911824 22916578 22921333 22926088 22930844 22935600 22940357

```
22945114 22949872 22954630 22959389 22964148 22968908 22973668 22978429 22983190
22987951 22992714 22997476 23002239 23007003 23011767 23016532 23021297 23026063
23030829 23035595 23040363 23045130 23049898 23054667 23059436 23064206 23068976
23073746 23078517 23083289 23088061 23092834 23097607 23102380 23107154 23111929
23116704 23121479 23126255 23131032 23135809 23140586 23145364 23150143 23154922
23159701 23164481 23169262 23174043 23178824 23183606 23188389 23193172 23197955
23202739 23207524 23212308 23217094 23221880 23226666 23231453 23236241 23241029
23245817 23250606 23255395 23260185 23264975 23269766 23274558 23279350 23284142
23288935 23293728 23298522 23303316 23308111 23312906 23317702 23322499 23327295
23332093 23336891 23341689 23346488 23351287 23356087 23360887 23365688 23370489
23375291 23380093 23384896 23389699 23394503 23399307 23404112 23408917 23413723
23418529 23423336 23428143 23432951 23437759 23442567 23447377 23452186 23456996
23461807 23466618 23471430 23476242 23481055 23485868 23490682 23495496 23500310
23505125 23509941 23514757 23519574 23524391 23529209 23534027 23538845 23543664
23548484 23553304 23558125 23562946 23567767 23572589 23577412 23582235 23587058
23591882 23596707 23601532 23606357 23611183 23616010 23620837 23625664 23630492
23635321 23640150 23644979 23649809 23654640 23659471 23664302 23669134 23673967
23678800 23683633 23688467 23693301 23698136 23702972 23707808 23712644 23717481
23722318 23727156 23731995 23736834 23741673 23746513 23751353 23756194 23761035
23765877 23770720 23775562 23780406 23785250 23790094 23794939 23799784 23804630
23809476 23814323 23819170 23824018 23828866 23833715 23838564 23843414 23848264
23853115 23857966 23862818 23867670 23872523 23877376 23882230 23887084 23891939
23896794 23901650 23906506 23911363 23916220 23921078 23925936 23930795 23935654
23940514 23945374 23950234 23955096 23959957 23964819 23969682 23974545 23979409
23984273 23989137 23994003 23998868 24003734 24008601 24013468 24018336 24023204
24028072 24032941 24037811 24042681 24047552 24052423 24057294 24062166 24067039
24071912 24076785 24081659 24086534 24091409 24096284 24101160 24106037 24110914
24115791 24120669 24125548 24130427 24135306 24140186 24145067 24149948 24154829
24159711 24164593 24169476 24174360 24179244 24184128 24189013 24193898 24198784
24203671 24208558 24213445 24218333 24223221 24228110 24233000 24237889 24242780
24247671 24252562 24257454 24262346 24267239 24272132 24277026 24281920 24286815
24291711 24296606 24301503 24306399 24311297 24316194 24321093 24325992 24330891
;

/*clean log*/

log/

/* feed random trees to the test*/

p mpt.tre;

set randmpttree getrandom [0 ntrees];

tsave* temp.tre;

save 'randmpttree';

keep 0;

p cons.tre;
```

```
set randcontree getrandom [0 ntrees];

save 'randcontree';

tsave/;

keep 0;

p temp.tre;


/* get character number */

set charnumber nchar ; /* note it gives number of chars -1! */


/* get number of trees */

set howmanytrees ntrees ; /* note it gives number of trees -1! */


    /* y-loop through characters */


    set nonzerodiffs 0 ;

    loop = y 0 'charnumber'


        /* compare changes of char y on tree 1 vs tree 0 */

        /* save result and absolute result in arrays */


        set thisdiff (score[1 (#y)] - score[0 (#y)]) ;

        if ('thisdiff' !=0)

            set diffs['nonzerodiffs'] 'thisdiff' ;

            if ('thisdiff' >= 0)

                set absdiffs['nonzerodiffs'] 'thisdiff' ;

            else

                set absdiffs['nonzerodiffs'] (-1*'thisdiff') ;

                end ;

            set nonzerodiffs ('nonzerodiffs'+1) ;

            end;

        stop ;
```

```
/* sort arrays */

if ('nonzerodiffs' > 1)
	loop =z 0 ('nonzerodiffs'-2)
		loop =i (#z+1) ('nonzerodiffs'-1)
			if ('absdiffs[#i]' < 'absdiffs[#z]')
				set swapbuffer 'diffs[#z]' ;
				set diffs[#z] 'diffs[#i]' ;
				set diffs[#i] 'swapbuffer' ;
				set swapbuffer 'absdiffs[#z]' ;
				set absdiffs[#z] 'absdiffs[#i]' ;
				set absdiffs[#i] 'swapbuffer' ;
			else
				end ;
			stop ;
		stop ;
	end ;

/* assign ranks; avg rank for ties */

set startrankblock 0 ;
set rankblocksum 1;
loop =r 0 ('nonzerodiffs'-1)
	if ((#r) == ('nonzerodiffs' -1))
		loop =s 'startrankblock' (#r)
			if ('diffs[#s]' < 0)
				set diffsrank[(#s)] ('rankblocksum' / (-1*(1 + (#r) -
'startrankblock'))) ;
			else
				set diffsrank[(#s)] ('rankblocksum' / (1 + (#r) -
'startrankblock')) ;
```

```
                        end;
                    stop ;
            else
                if ('absdiffs[(#r)]' == 'absdiffs[((#r)+1)]')
                    set rankblocksum ('rankblocksum' + #r + 2) ;
                else
                    loop =s 'startrankblock' (#r)
                        if ('diffs[(#s)]' < 0)
                            set diffsrank[(#s)] ('rankblocksum' / (-1*(1 +
(#r) - 'startrankblock'))) ;
                        else
                            set diffsrank[(#s)] ('rankblocksum' / (1 + (#r) -
'startrankblock')) ;
                            end;
                        stop ;
                    set startrankblock ((#r) + 1) ;
                    set rankblocksum ((#r) + 2);
                    end ;
                end ;
            stop ;

        /* obtain test statistic */

        set lookupwilc 0 ;
        set teststat 0 ;
        loop =t 0 ('nonzerodiffs'-1)
            if ('diffsrank[#t]'<0)
                set teststat ('teststat' - 'diffsrank[#t]') ;
                end ;
            if ('diffs[#t]' !=0)
                set lookupwilc ('lookupwilc' + 1) ;
                end ;
```

```
			stop;

		/* output sorted list of differences */

		quote Templeton test of most parsimonipus tree number 'randmpttree' against
constrained tree number 'randcontree' ;
		quote Rank Difference Rankscore ;
		loop =u 0 ('nonzerodiffs'-1)
			if ('diffs[#u]' != 0)
				set swapbuffer (#u+1) ;
				quote 'swapbuffer' 'diffs[#u]' 'diffsrank[#u]' ;
				end ;
			stop ;
		quote Sum of negative ranks 'teststat' ;
		quote Number of non-zero scores 'lookupwilc' ;

		/* compare against Wilcoxon table and output test result */
if ('lookupwilc' < 5)
			quote Too few differences between the trees ;
		else
			quote  'wilc5[('lookupwilc'-1)]' 'wilc2_5[('lookupwilc'-1)]' 'wilc1[('lookupwilc'-
1)]';
			if ('teststat' <= 'wilc5[('lookupwilc'-1)]')
				if ('teststat' <= 'wilc2_5[('lookupwilc'-1)]')
					if ('teststat' <= 'wilc1[('lookupwilc'-1)]')
						quote Significant at the 1 percent level ;
						set results 1;
						stop;
					else
						quote Significant at the 2.5 percent level ;
						set results 2.5;
stop;
```

```
                    end ;
            else
                quote Significant at the 5 percent level ;
                set results 5;
                stop;
                end ;
        else
            quote Not significant ;
            set results 0;
            end;
        end;
    ;
/*generate results table*/
log +results.out
quote   'randmpttree', 'randcontree', 'teststat', 'lookupwilc', 'wilc5[('lookupwilc'-1)]',
'wilc2_5[('lookupwilc'-1)]', 'wilc1[('lookupwilc'-1)]', 'results';



log/

procedure / ;
```

Supp C5. Tab delimited text file with the results of the Templeton tests for the three datasets.

Random MPT Random constrained tree Sum Negative ranks Non zero scores Rank for significance at 0.05 Rank for significance at 0.025 Rank for significance at 0.001 Result

2906 5758 26 12 " 17" 13 9 Non significant
821 1963 26 12 " 17" 13 9 Non significant
3175 5627 16.500.000 10 " 10" 8 5 Non significant
4426 7419 84 20 " 60" 52 43 Non significant
2274 4086 26 12 " 17" 13 9 Non significant
2749 4991 37.500.000 14 " 25" 21 15 Non significant
4438 4284 201.500.000 30 " 151" 137 120 Non significant
4349 1991 148.500.000 26 " 110" 98 84 Non significant
1859 5631 125 24 " 91" 81 69 Non significant
1907 2238 26 12 " 17" 13 9 Non significant
3736 4978 103.500.000 22 " 75" 65 55 Non significant
1911 2535 37.500.000 14 " 25" 21 15 Non significant
2075 6726 66.500.000 18 " 47" 40 32 Non significant
3093 5369 9 8 " 5" 3 1 Non significant
1298 2408 115 23 " 83" 73 62 Non significant
2648 5177 26 12 " 17" 13 9 Non significant
518 6550 84 20 " 60" 52 43 Non significant
1621 3515 84 20 " 60" 52 43 Non significant
3462 3877 76 19 " 53" 46 37 Non significant
1915 3768 9 8 " 5" 3 1 Non significant
4336 7412 103.500.000 22 " 75" 65 55 Non significant
4209 4143 16.500.000 10 " 10" 8 5 Non significant
2321 302 45 15 " 30" 25 19 Non significant
935 5627 51 16 " 35" 29 23 Non significant
2332 1506 84 20 " 60" 52 43 Non significant
3477 920 26 12 " 17" 13 9 Non significant
1152 1475 103.500.000 22 " 75" 65 55 Non significant
3316 3766 115 23 " 83" 73 62 Non significant
1422 223 51 16 " 35" 29 23 Non significant
1165 1945 84 20 " 60" 52 43 Non significant
541 6655 103.500.000 22 " 75" 65 55 Non significant
167 3894 51 16 " 35" 29 23 Non significant
86 483 84 20 " 60" 52 43 Non significant
2310 7757 66.500.000 18 " 47" 40 32 Non significant
1691 5530 188.500.000 29 " 140" 126 110 Non significant
730 6214 125 24 " 91" 81 69 Non significant
2446 4740 76 19 " 53" 46 37 Non significant
1414 5262 84 20 " 60" 52 43 Non significant
3178 7273 84 20 " 60" 52 43 Non significant
3181 5358 9 8 " 5" 3 1 Non significant
2113 246 76 19 " 53" 46 37 Non significant
1941 7536 37.500.000 14 " 25" 21 15 Non significant
468 2203 66.500.000 18 " 47" 40 32 Non significant
1195 1201 84 20 " 60" 52 43 Non significant
2752 5285 84 20 " 60" 52 43 Non significant
3576 677 3.500.000 6 " 2" 0 0 Non significant
2853 7644 76 19 " 53" 46 37 Non significant
4245 589 86 20 " 60" 52 43 Non significant
2284 6363 51 16 " 35" 29 23 Non significant
3504 3193 66.500.000 18 " 47" 40 32 Non significant
556 1395 37.500.000 14 " 25" 21 15 Non significant
4434 4989 51 16 " 35" 29 23 Non significant
1352 6815 137.500.000 25 " 100" 89 76 Non significant
4586 1877 103.500.000 22 " 75" 65 55 Non significant
3076 7174 66.500.000 18 " 47" 40 32 Non significant

4513	120	37.500.000	14	"	25"	21	15	Non significant
1827	1330	66.500.000	18	"	47"	40	32	Non significant
3547	6334	37.500.000	14	"	25"	21	15	Non significant
1518	4882	84	20	"	60"	52	43	Non significant
1444	945	103.500.000	22	"	75"	65	55	Non significant
3664	6386	174	28	"	130"	116	101	Non significant
4166	7221	26	12	"	17"	13	9	Non significant
3147	1206	84	20	"	60"	52	43	Non significant
2807	4247	37.500.000	14	"	25"	21	15	Non significant
3280	7129	16.500.000	10	"	10"	8	5	Non significant
2655	2582	84	20	"	60"	52	43	Non significant
1672	5000	84	20	"	60"	52	43	Non significant
3406	7637	137.500.000	25	"	100"	89	76	Non significant
1536	1276	37.500.000	14	"	25"	21	15	Non significant
1701	2876	13.500.000	9	"	8"	5	3	Non significant
1155	4699	16.500.000	10	"	10"	8	5	Non significant
1787	1713	37.500.000	14	"	25"	21	15	Non significant
3214	3919	103.500.000	22	"	75"	65	55	Non significant
2084	4697	37.500.000	14	"	25"	21	15	Non significant
2313	7745	66.500.000	18	"	47"	40	32	Non significant
681	7535	148.500.000	26	"	110"	98	84	Non significant
3123	445	51	16	"	35"	29	23	Non significant
4127	928	162	27	"	119"	107	92	Non significant
389	5349	16.500.000	10	"	10"	8	5	Non significant
2383	6157	37.500.000	14	"	25"	21	15	Non significant
607	1491	84	20	"	60"	52	43	Non significant
3829	1822	66.500.000	18	"	47"	40	32	Non significant
4588	5935	103.500.000	22	"	75"	65	55	Non significant
754	6790	66.500.000	18	"	47"	40	32	Non significant
4598	436	66.500.000	18	"	47"	40	32	Non significant
1294	5850	3.500.000	6	"	2"	0	0	Non significant
4273	611	59.500.000	17	"	41"	34	27	Non significant
1171	913	94.500.000	21	"	67"	58	49	Non significant
3752	4612	66.500.000	18	"	47"	40	32	Non significant
2607	3707	37.500.000	14	"	25"	21	15	Non significant
4184	3968	26	12	"	17"	13	9	Non significant
4590	33	16.500.000	10	"	10"	8	5	Non significant
717	4607	66.500.000	18	"	47"	40	32	Non significant
995	2624	26	12	"	17"	13	9	Non significant
283	5250	37.500.000	14	"	25"	21	15	Non significant
4133	4357	66.500.000	18	"	47"	40	32	Non significant
1468	3937	103.500.000	22	"	75"	65	55	Non significant
3775	5511	37.500.000	14	"	25"	21	15	Non significant
2877	7074	150.500.000	26	"	110"	98	84	Non significant
3999	3496	66.500.000	18	"	47"	40	32	Non significant
3473	6270	137.500.000	25	"	100"	89	76	Non significant
4578	3753	16.500.000	10	"	10"	8	5	Non significant
4269	4500	37.500.000	14	"	25"	21	15	Non significant
3961	5808	162	27	"	119"	107	92	Non significant
1787	4681	37.500.000	14	"	25"	21	15	Non significant
2604	922	76	19	"	53"	46	37	Non significant
2239	1670	66.500.000	18	"	47"	40	32	Non significant
3952	1472	37.500.000	14	"	25"	21	15	Non significant
1310	687	103.500.000	22	"	75"	65	55	Non significant
4309	3500	37.500.000	14	"	25"	21	15	Non significant
3048	3813	103.500.000	22	"	75"	65	55	Non significant
4100	6424	148.500.000	26	"	110"	98	84	Non significant

4154	2523	37.500.000	14	"	25"	21	15	Non significant
1212	5835	103.500.000	22	"	75"	65	55	Non significant
2231	2905	148.500.000	26	"	110"	98	84	Non significant
1856	5554	125	24	"	91"	81	69	Non significant
765	2098	125	24	"	91"	81	69	Non significant
802	1568	59.500.000	17	"	41"	34	27	Non significant
4359	2022	148.500.000	26	"	110"	98	84	Non significant
1191	6789	51	16	"	35"	29	23	Non significant
695	3959	9	8	"	5"	3	1	Non significant
1780	1488	51	16	"	35"	29	23	Non significant
3954	5035	115	23	"	83"	73	62	Non significant
3749	1092	37.500.000	14	"	25"	21	15	Non significant
850	4614	51	16	"	35"	29	23	Non significant
1611	668	76	19	"	53"	46	37	Non significant
1947	6661	26	12	"	17"	13	9	Non significant
2989	574	26	12	"	17"	13	9	Non significant
3678	1082	103.500.000	22	"	75"	65	55	Non significant
987	3680	115	23	"	83"	73	62	Non significant
2615	2859	66.500.000	18	"	47"	40	32	Non significant
653	862	59.500.000	17	"	41"	34	27	Non significant
446	905	94.500.000	21	"	67"	58	49	Non significant
4416	4087	148.500.000	26	"	110"	98	84	Non significant
2035	6001	66.500.000	18	"	47"	40	32	Non significant
2499	3786	26	12	"	17"	13	9	Non significant
568	2501	51	16	"	35"	29	23	Non significant
1322	2579	137.500.000	25	"	100"	89	76	Non significant
1044	5416	66.500.000	18	"	47"	40	32	Non significant
2859	5990	26	12	"	17"	13	9	Non significant
1545	3099	37.500.000	14	"	25"	21	15	Non significant
1245	487	26	12	"	17"	13	9	Non significant
3096	6042	26	12	"	17"	13	9	Non significant
541	1775	103.500.000	22	"	75"	65	55	Non significant
73	342	32.500.000	13	"	21"	17	12	Non significant
1118	288	37.500.000	14	"	25"	21	15	Non significant
1925	209	37.500.000	14	"	25"	21	15	Non significant
2093	1282	37.500.000	14	"	25"	21	15	Non significant
2219	4795	84	20	"	60"	52	43	Non significant
3474	6469	59.500.000	17	"	41"	34	27	Non significant
2750	879	51	16	"	35"	29	23	Non significant
3113	6806	84	20	"	60"	52	43	Non significant
1876	5036	103.500.000	22	"	75"	65	55	Non significant
234	5629	137.500.000	25	"	100"	89	76	Non significant
2235	5780	174	28	"	130"	116	101	Non significant
915	3356	66.500.000	18	"	47"	40	32	Non significant
597	3079	9	8	"	5"	3	1	Non significant
3988	807	188.500.000	29	"	140"	126	110	Non significant
1749	253	37.500.000	14	"	25"	21	15	Non significant
914	5635	103.500.000	22	"	75"	65	55	Non significant
3540	6246	103.500.000	22	"	75"	65	55	Non significant
857	609	32.500.000	13	"	21"	17	12	Non significant
1133	1505	103.500.000	22	"	75"	65	55	Non significant
2758	5432	148.500.000	26	"	110"	98	84	Non significant
1769	5535	148.500.000	26	"	110"	98	84	Non significant
2431	5348	37.500.000	14	"	25"	21	15	Non significant
847	7701	201.500.000	30	"	151"	137	120	Non significant
1691	4288	59.500.000	17	"	41"	34	27	Non significant
827	963	37.500.000	14	"	25"	21	15	Non significant

3645	1316	51	16	"	35"	29	23	Non significant
1233	712	51	16	"	35"	29	23	Non significant
740	6015	125	24	"	91"	81	69	Non significant
1462	5601	84	20	"	60"	52	43	Non significant
3546	1491	37.500.000	14	"	25"	21	15	Non significant
383	325	103.500.000	22	"	75"	65	55	Non significant
3391	82	66.500.000	18	"	47"	40	32	Non significant
1676	1090	16.500.000	10	"	10"	8	5	Non significant
2165	3836	37.500.000	14	"	25"	21	15	Non significant
4280	6582	66.500.000	18	"	47"	40	32	Non significant
3401	7574	103.500.000	22	"	75"	65	55	Non significant
50	685	103.500.000	22	"	75"	65	55	Non significant
2332	7722	137.500.000	25	"	100"	89	76	Non significant
2538	6365	103.500.000	22	"	75"	65	55	Non significant
2542	4193	9	8	"	5"	3	1	Non significant
4035	3235	66.500.000	18	"	47"	40	32	Non significant
4474	1249	174	28	"	130"	116	101	Non significant
3371	1403	3.500.000	6	"	2"	0	0	Non significant
774	5222	51	16	"	35"	29	23	Non significant
3334	5281	37.500.000	14	"	25"	21	15	Non significant
4638	2	188.500.000	29	"	140"	126	110	Non significant
394	3339	51	16	"	35"	29	23	Non significant
1645	340	37.500.000	14	"	25"	21	15	Non significant
1532	163	59.500.000	17	"	41"	34	27	Non significant
3347	650	137.500.000	25	"	100"	89	76	Non significant
142	3558	66.500.000	18	"	47"	40	32	Non significant
4058	7062	59.500.000	17	"	41"	34	27	Non significant
3784	4125	51	16	"	35"	29	23	Non significant
2038	6513	37.500.000	14	"	25"	21	15	Non significant
3911	1150	148.500.000	26	"	110"	98	84	Non significant
3623	5682	174	28	"	130"	116	101	Non significant
652	35	16.500.000	10	"	10"	8	5	Non significant
2503	3500	84	20	"	60"	52	43	Non significant
3359	7333	162	27	"	119"	107	92	Non significant
3137	3979	103.500.000	22	"	75"	65	55	Non significant
1383	288	59.500.000	17	"	41"	34	27	Non significant
4177	6381	148.500.000	26	"	110"	98	84	Non significant
1112	5222	201.500.000	30	"	151"	137	120	Non significant
3550	3393	148.500.000	26	"	110"	98	84	Non significant
1002	3424	66.500.000	18	"	47"	40	32	Non significant
525	6529	103.500.000	22	"	75"	65	55	Non significant
3305	6601	16.500.000	10	"	10"	8	5	Non significant
378	7214	59.500.000	17	"	41"	34	27	Non significant
1731	6089	66.500.000	18	"	47"	40	32	Non significant
2139	3887	66.500.000	18	"	47"	40	32	Non significant
3384	177	125	24	"	91"	81	69	Non significant
2911	3561	37.500.000	14	"	25"	21	15	Non significant
2699	7547	9	8	"	5"	3	1	Non significant
3885	3741	103.500.000	22	"	75"	65	55	Non significant
3629	5151	51	16	"	35"	29	23	Non significant
674	2597	16.500.000	10	"	10"	8	5	Non significant
947	4565	103.500.000	22	"	75"	65	55	Non significant
1289	3821	162	27	"	119"	107	92	Non significant
785	6287	174	28	"	130"	116	101	Non significant
1830	5201	9	8	"	5"	3	1	Non significant
4236	7730	9	8	"	5"	3	1	Non significant
1084	5658	51	16	"	35"	29	23	Non significant

4289	4280	162	27	"	119"	107	92	Non significant
496	6999	9	8	"	5"	3	1	Non significant
1865	6470	37.500.000	14	"	25"	21	15	Non significant
54	2941	51	16	"	35"	29	23	Non significant
3189	398	203.500.000	30	"	151"	137	120	Non significant
2800	2719	125	24	"	91"	81	69	Non significant
1071	3300	94.500.000	21	"	67"	58	49	Non significant
3055	2444	148.500.000	26	"	110"	98	84	Non significant
636	7009	37.500.000	14	"	25"	21	15	Non significant
4341	2028	51	16	"	35"	29	23	Non significant
3341	3961	37.500.000	14	"	25"	21	15	Non significant
1967	4760	66.500.000	18	"	47"	40	32	Non significant
488	7357	66.500.000	18	"	47"	40	32	Non significant
3410	700	94.500.000	21	"	67"	58	49	Non significant
1826	2384	51	16	"	35"	29	23	Non significant
4353	2029	84	20	"	60"	52	43	Non significant
349	4554	9	8	"	5"	3	1	Non significant
4006	6025	103.500.000	22	"	75"	65	55	Non significant
3492	3726	51	16	"	35"	29	23	Non significant
318	5460	37.500.000	14	"	25"	21	15	Non significant
3489	7051	125	24	"	91"	81	69	Non significant
318	3692	66.500.000	18	"	47"	40	32	Non significant
4640	5589	84	20	"	60"	52	43	Non significant
327	4337	37.500.000	14	"	25"	21	15	Non significant
2709	6113	3.500.000	6	"	2"	0	0	Non significant
900	4983	125	24	"	91"	81	69	Non significant
1462	6211	16.500.000	10	"	10"	8	5	Non significant
4144	7559	51	16	"	35"	29	23	Non significant
4120	1416	51	16	"	35"	29	23	Non significant
559	1017	37.500.000	14	"	25"	21	15	Non significant
3329	243	45	15	"	30"	25	19	Non significant
3077	2770	103.500.000	22	"	75"	65	55	Non significant
1594	887	188.500.000	29	"	140"	126	110	Non significant
4155	1213	84	20	"	60"	52	43	Non significant
1896	4312	51	16	"	35"	29	23	Non significant
4295	4714	9	8	"	5"	3	1	Non significant
2629	3106	94.500.000	21	"	67"	58	49	Non significant
1556	1545	66.500.000	18	"	47"	40	32	Non significant
416	4615	103.500.000	22	"	75"	65	55	Non significant
3371	4516	26	12	"	17"	13	9	Non significant
2908	3962	66.500.000	18	"	47"	40	32	Non significant
603	4256	84	20	"	60"	52	43	Non significant
2768	940	76	19	"	53"	46	37	Non significant
3819	946	203.500.000	30	"	151"	137	120	Non significant
68	1961	66.500.000	18	"	47"	40	32	Non significant
3408	5154	16.500.000	10	"	10"	8	5	Non significant
834	1618	94.500.000	21	"	67"	58	49	Non significant
530	3713	103.500.000	22	"	75"	65	55	Non significant
2868	5016	59.500.000	17	"	41"	34	27	Non significant
3990	4009	125	24	"	91"	81	69	Non significant
3021	2141	37.500.000	14	"	25"	21	15	Non significant
1878	6552	103.500.000	22	"	75"	65	55	Non significant
2515	7019	84	20	"	60"	52	43	Non significant
2595	4204	37.500.000	14	"	25"	21	15	Non significant
4400	4689	51	16	"	35"	29	23	Non significant
2260	1213	84	20	"	60"	52	43	Non significant
2015	321	51	16	"	35"	29	23	Non significant

3447	510	76	19	"	53"	46	37	Non significant
1409	6891	45	15	"	30"	25	19	Non significant
2378	2897	125	24	"	91"	81	69	Non significant
3517	6871	125	24	"	91"	81	69	Non significant
3525	6637	37.500.000	14	"	25"	21	15	Non significant
995	933	76	19	"	53"	46	37	Non significant
2200	6609	66.500.000	18	"	47"	40	32	Non significant
1595	5861	84	20	"	60"	52	43	Non significant
1440	4838	37.500.000	14	"	25"	21	15	Non significant
3649	4117	174	28	"	130"	116	101	Non significant
2531	3862	103.500.000	22	"	75"	65	55	Non significant
3237	3325	137.500.000	25	"	100"	89	76	Non significant
3932	4686	51	16	"	35"	29	23	Non significant
4606	5900	103.500.000	22	"	75"	65	55	Non significant
3471	1364	3.500.000	6	"	2"	0	0	Non significant
3634	4913	51	16	"	35"	29	23	Non significant
4553	1704	84	20	"	60"	52	43	Non significant
1696	3033	115	23	"	83"	73	62	Non significant
4163	92	9	8	"	5"	3	1	Non significant
4350	651	32.500.000	13	"	21"	17	12	Non significant
1291	4084	137.500.000	25	"	100"	89	76	Non significant
1800	613	26	12	"	17"	13	9	Non significant
1612	5244	16.500.000	10	"	10"	8	5	Non significant
3163	4500	94.500.000	21	"	67"	58	49	Non significant
2513	3660	103.500.000	22	"	75"	65	55	Non significant
1192	1270	125	24	"	91"	81	69	Non significant
4244	444	84	20	"	60"	52	43	Non significant
3062	7601	84	20	"	60"	52	43	Non significant
947	7207	115	23	"	83"	73	62	Non significant
4350	1903	162	27	"	119"	107	92	Non significant
1513	2817	115	23	"	83"	73	62	Non significant
753	3519	84	20	"	60"	52	43	Non significant
3463	6455	76	19	"	53"	46	37	Non significant
4167	781	51	16	"	35"	29	23	Non significant
1057	2347	37.500.000	14	"	25"	21	15	Non significant
172	4412	37.500.000	14	"	25"	21	15	Non significant
773	2861	26	12	"	17"	13	9	Non significant
2109	6089	125	24	"	91"	81	69	Non significant
3061	985	125	24	"	91"	81	69	Non significant
1603	2528	76	19	"	53"	46	37	Non significant
4435	6066	37.500.000	14	"	25"	21	15	Non significant
3716	5402	84	20	"	60"	52	43	Non significant
2332	2690	66.500.000	18	"	47"	40	32	Non significant
3822	6493	26	12	"	17"	13	9	Non significant
2726	2734	37.500.000	14	"	25"	21	15	Non significant
2683	7122	84	20	"	60"	52	43	Non significant
1916	1019	26	12	"	17"	13	9	Non significant
3367	4645	16.500.000	10	"	10"	8	5	Non significant
3640	132	125	24	"	91"	81	69	Non significant
448	7327	127	24	"	91"	81	69	Non significant
609	2070	51	16	"	35"	29	23	Non significant
952	3107	94.500.000	21	"	67"	58	49	Non significant
1651	1201	125	24	"	91"	81	69	Non significant
1409	1354	26	12	"	17"	13	9	Non significant
3524	1119	162	27	"	119"	107	92	Non significant
4046	1470	125	24	"	91"	81	69	Non significant
3219	2194	103.500.000	22	"	75"	65	55	Non significant

1081	1881	59.500.000	17	"	41"	34	27	Non significant
3129	2912	51	16	"	35"	29	23	Non significant
883	1596	125	24	"	91"	81	69	Non significant
3453	2877	9	8	"	5"	3	1	Non significant
3925	4558	37.500.000	14	"	25"	21	15	Non significant
2393	7324	84	20	"	60"	52	43	Non significant
577	635	26	12	"	17"	13	9	Non significant
3433	1509	26	12	"	17"	13	9	Non significant
1867	1768	37.500.000	14	"	25"	21	15	Non significant
3046	5365	16.500.000	10	"	10"	8	5	Non significant
1391	2743	45	15	"	30"	25	19	Non significant
2888	4089	115	23	"	83"	73	62	Non significant
57	63	37.500.000	14	"	25"	21	15	Non significant
2687	6632	103.500.000	22	"	75"	65	55	Non significant
1374	41	51	16	"	35"	29	23	Non significant
4436	35	84	20	"	60"	52	43	Non significant
2114	2410	9	8	"	5"	3	1	Non significant
759	1006	26	12	"	17"	13	9	Non significant
1484	3332	66.500.000	18	"	47"	40	32	Non significant
1978	5995	125	24	"	91"	81	69	Non significant
4472	5755	188.500.000	29	"	140"	126	110	Non significant
3127	2907	16.500.000	10	"	10"	8	5	Non significant
4367	440	51	16	"	35"	29	23	Non significant
958	19	84	20	"	60"	52	43	Non significant
1303	2303	26	12	"	17"	13	9	Non significant
1524	928	94.500.000	21	"	67"	58	49	Non significant
3417	944	16.500.000	10	"	10"	8	5	Non significant
2726	4379	84	20	"	60"	52	43	Non significant
3683	6657	125	24	"	91"	81	69	Non significant
731	5546	201.500.000	30	"	151"	137	120	Non significant
1971	4662	51	16	"	35"	29	23	Non significant
3152	6557	9	8	"	5"	3	1	Non significant
2822	2246	125	24	"	91"	81	69	Non significant
606	2787	37.500.000	14	"	25"	21	15	Non significant
2734	2637	148.500.000	26	"	110"	98	84	Non significant
1319	2768	115	23	"	83"	73	62	Non significant
851	1936	26	12	"	17"	13	9	Non significant
1376	1519	66.500.000	18	"	47"	40	32	Non significant
937	1300	51	16	"	35"	29	23	Non significant
2906	2950	37.500.000	14	"	25"	21	15	Non significant
2289	4029	66.500.000	18	"	47"	40	32	Non significant
4613	5583	188.500.000	29	"	140"	126	110	Non significant
3476	6951	137.500.000	25	"	100"	89	76	Non significant
4071	5008	32.500.000	13	"	21"	17	12	Non significant
4605	807	188.500.000	29	"	140"	126	110	Non significant
795	2911	174	28	"	130"	116	101	Non significant
3109	607	125	24	"	91"	81	69	Non significant
3988	4375	16.500.000	10	"	10"	8	5	Non significant
82	2436	45	15	"	30"	25	19	Non significant
983	2777	84	20	"	60"	52	43	Non significant
4076	943	115	23	"	83"	73	62	Non significant
3776	5059	66.500.000	18	"	47"	40	32	Non significant
4154	101	125	24	"	91"	81	69	Non significant
1019	5617	26	12	"	17"	13	9	Non significant
3032	5960	66.500.000	18	"	47"	40	32	Non significant
2293	5462	32.500.000	13	"	21"	17	12	Non significant
4093	601	103.500.000	22	"	75"	65	55	Non significant

4273	5977	76	19	"	53"	46	37	Non significant
4503	1620	26	12	"	17"	13	9	Non significant
62	6224	125	24	"	91"	81	69	Non significant
413	2953	94.500.000	21	"	67"	58	49	Non significant
3097	3581	26	12	"	17"	13	9	Non significant
241	1980	51	16	"	35"	29	23	Non significant
1555	4523	26	12	"	17"	13	9	Non significant
198	3132	45	15	"	30"	25	19	Non significant
1681	6407	76	19	"	53"	46	37	Non significant
4120	1860	84	20	"	60"	52	43	Non significant
2377	4830	150.500.000	26	"	110"	98	84	Non significant
3608	5811	162	27	"	119"	107	92	Non significant
2522	5242	84	20	"	60"	52	43	Non significant
2994	5995	26	12	"	17"	13	9	Non significant
4614	6684	9	8	"	5"	3	1	Non significant
1963	5899	37.500.000	14	"	25"	21	15	Non significant
3730	4120	125	24	"	91"	81	69	Non significant
4440	750	66.500.000	18	"	47"	40	32	Non significant
1211	2803	26	12	"	17"	13	9	Non significant
3999	3776	76	19	"	53"	46	37	Non significant
43	2782	51	16	"	35"	29	23	Non significant
1919	2276	125	24	"	91"	81	69	Non significant
4172	4783	37.500.000	14	"	25"	21	15	Non significant
1792	4167	66.500.000	18	"	47"	40	32	Non significant
4466	36	84	20	"	60"	52	43	Non significant
3808	5601	66.500.000	18	"	47"	40	32	Non significant
3118	791	26	12	"	17"	13	9	Non significant
2755	3185	51	16	"	35"	29	23	Non significant
14	800	148.500.000	26	"	110"	98	84	Non significant
1375	2933	51	16	"	35"	29	23	Non significant
1692	1389	22	11	"	13"	10	7	Non significant
1557	2924	201.500.000	30	"	151"	137	120	Non significant
3532	1826	16.500.000	10	"	10"	8	5	Non significant
3567	1197	84	20	"	60"	52	43	Non significant
450	6833	84	20	"	60"	52	43	Non significant
514	6948	125	24	"	91"	81	69	Non significant
194	7768	125	24	"	91"	81	69	Non significant
203	495	9	8	"	5"	3	1	Non significant
365	253	76	19	"	53"	46	37	Non significant
3129	4476	125	24	"	91"	81	69	Non significant
3779	938	76	19	"	53"	46	37	Non significant
2998	6784	37.500.000	14	"	25"	21	15	Non significant
2724	1736	103.500.000	22	"	75"	65	55	Non significant
875	1015	76	19	"	53"	46	37	Non significant
2553	3612	37.500.000	14	"	25"	21	15	Non significant
1554	7136	174	28	"	130"	116	101	Non significant
3213	6660	125	24	"	91"	81	69	Non significant
4514	7668	125	24	"	91"	81	69	Non significant
1064	4049	103.500.000	22	"	75"	65	55	Non significant
833	6442	66.500.000	18	"	47"	40	32	Non significant
2695	1602	103.500.000	22	"	75"	65	55	Non significant
3609	1931	84	20	"	60"	52	43	Non significant
4301	7268	26	12	"	17"	13	9	Non significant
6	7254	37.500.000	14	"	25"	21	15	Non significant
2396	5003	84	20	"	60"	52	43	Non significant
4327	1012	51	16	"	35"	29	23	Non significant
685	1199	84	20	"	60"	52	43	Non significant

4132	1080	148.500.000	26	"	110"	98	84	Non significant
1844	4327	37.500.000	14	"	25"	21	15	Non significant
3009	5534	66.500.000	18	"	47"	40	32	Non significant
3072	4262	125	24	"	91"	81	69	Non significant
441	1697	103.500.000	22	"	75"	65	55	Non significant
296	7448	148.500.000	26	"	110"	98	84	Non significant
3559	250	16.500.000	10	"	10"	8	5	Non significant
1065	7227	37.500.000	14	"	25"	21	15	Non significant
2661	1020	201.500.000	30	"	151"	137	120	Non significant
4329	6590	37.500.000	14	"	25"	21	15	Non significant
178	5072	51	16	"	35"	29	23	Non significant
1886	6490	103.500.000	22	"	75"	65	55	Non significant
22	336	16.500.000	10	"	10"	8	5	Non significant
3226	1522	84	20	"	60"	52	43	Non significant
504	5382	84	20	"	60"	52	43	Non significant
2370	2156	45	15	"	30"	25	19	Non significant
3921	6882	148.500.000	26	"	110"	98	84	Non significant
901	2541	103.500.000	22	"	75"	65	55	Non significant
887	990	22	11	"	13"	10	7	Non significant
882	4246	51	16	"	35"	29	23	Non significant
2914	6866	9	8	"	5"	3	1	Non significant
1435	6561	16.500.000	10	"	10"	8	5	Non significant
1582	7670	66.500.000	18	"	47"	40	32	Non significant
4618	3162	66.500.000	18	"	47"	40	32	Non significant
4605	1627	66.500.000	18	"	47"	40	32	Non significant
521	4701	125	24	"	91"	81	69	Non significant
971	6280	125	24	"	91"	81	69	Non significant
2042	4338	51	16	"	35"	29	23	Non significant
1772	1366	32.500.000	13	"	21"	17	12	Non significant
4386	171	103.500.000	22	"	75"	65	55	Non significant
2323	1098	9	8	"	5"	3	1	Non significant
149	1626	66.500.000	18	"	47"	40	32	Non significant
1903	1430	51	16	"	35"	29	23	Non significant
4489	3763	125	24	"	91"	81	69	Non significant
1295	6286	45	15	"	30"	25	19	Non significant
1415	7653	103.500.000	22	"	75"	65	55	Non significant
2736	2858	148.500.000	26	"	110"	98	84	Non significant
1445	791	51	16	"	35"	29	23	Non significant
2591	2721	45	15	"	30"	25	19	Non significant
1085	6788	32.500.000	13	"	21"	17	12	Non significant
894	480	37.500.000	14	"	25"	21	15	Non significant
152	5590	51	16	"	35"	29	23	Non significant
3427	141	16.500.000	10	"	10"	8	5	Non significant
2144	1188	66.500.000	18	"	47"	40	32	Non significant
800	2463	51	16	"	35"	29	23	Non significant
2535	3052	37.500.000	14	"	25"	21	15	Non significant
1999	7449	103.500.000	22	"	75"	65	55	Non significant
16	6671	103.500.000	22	"	75"	65	55	Non significant
196	1898	84	20	"	60"	52	43	Non significant
2298	1583	66.500.000	18	"	47"	40	32	Non significant
1616	2790	16.500.000	10	"	10"	8	5	Non significant
3278	4489	51	16	"	35"	29	23	Non significant
1635	3714	103.500.000	22	"	75"	65	55	Non significant
3473	4785	150.500.000	26	"	110"	98	84	Non significant
1682	1746	59.500.000	17	"	41"	34	27	Non significant
629	82	26	12	"	17"	13	9	Non significant
2767	3619	84	20	"	60"	52	43	Non significant

2828	2339	66.500.000	18	"	47"	40	32	Non significant
1208	343	84	20	"	60"	52	43	Non significant
2029	8	125	24	"	91"	81	69	Non significant
372	2371	32.500.000	13	"	21"	17	12	Non significant
3395	6254	201.500.000	30	"	151"	137	120	Non significant
2400	619	26	12	"	17"	13	9	Non significant
1628	1956	84	20	"	60"	52	43	Non significant
328	7640	76	19	"	53"	46	37	Non significant
3111	2458	103.500.000	22	"	75"	65	55	Non significant
841	415	37.500.000	14	"	25"	21	15	Non significant
1910	1786	66.500.000	18	"	47"	40	32	Non significant
3920	7314	137.500.000	25	"	100"	89	76	Non significant
1633	1065	26	12	"	17"	13	9	Non significant
3623	7426	26	12	"	17"	13	9	Non significant
712	380	37.500.000	14	"	25"	21	15	Non significant
565	5371	103.500.000	22	"	75"	65	55	Non significant
2297	5882	115	23	"	83"	73	62	Non significant
2298	253	37.500.000	14	"	25"	21	15	Non significant
2279	3574	174	28	"	130"	116	101	Non significant
1439	891	125	24	"	91"	81	69	Non significant
2430	393	86	20	"	60"	52	43	Non significant
1393	2006	16.500.000	10	"	10"	8	5	Non significant
1232	3601	148.500.000	26	"	110"	98	84	Non significant
1270	184	84	20	"	60"	52	43	Non significant
809	2924	125	24	"	91"	81	69	Non significant
2741	4304	103.500.000	22	"	75"	65	55	Non significant
4508	7506	125	24	"	91"	81	69	Non significant
3769	1488	37.500.000	14	"	25"	21	15	Non significant
2522	2229	9	8	"	5"	3	1	Non significant
609	2120	51	16	"	35"	29	23	Non significant
1501	6490	59.500.000	17	"	41"	34	27	Non significant
2624	1120	32.500.000	13	"	21"	17	12	Non significant
1862	5589	103.500.000	22	"	75"	65	55	Non significant
2104	4567	105.500.000	22	"	75"	65	55	Non significant
1782	2246	66.500.000	18	"	47"	40	32	Non significant
4609	543	45	15	"	30"	25	19	Non significant
4413	4501	188.500.000	29	"	140"	126	110	Non significant
3593	4917	45	15	"	30"	25	19	Non significant
1938	3662	37.500.000	14	"	25"	21	15	Non significant
1154	993	103.500.000	22	"	75"	65	55	Non significant
2490	1277	125	24	"	91"	81	69	Non significant
2424	4274	51	16	"	35"	29	23	Non significant
672	1640	51	16	"	35"	29	23	Non significant
2344	6230	84	20	"	60"	52	43	Non significant
4199	975	174	28	"	130"	116	101	Non significant
4043	1180	127	24	"	91"	81	69	Non significant
4157	2596	103.500.000	22	"	75"	65	55	Non significant
1031	7307	125	24	"	91"	81	69	Non significant
3862	4234	66.500.000	18	"	47"	40	32	Non significant
814	2110	103.500.000	22	"	75"	65	55	Non significant
663	2864	37.500.000	14	"	25"	21	15	Non significant
2795	4358	51	16	"	35"	29	23	Non significant
4353	3818	103.500.000	22	"	75"	65	55	Non significant
3963	4541	51	16	"	35"	29	23	Non significant
3935	5107	66.500.000	18	"	47"	40	32	Non significant
204	5932	26	12	"	17"	13	9	Non significant
1577	2565	84	20	"	60"	52	43	Non significant

1006	4252	66.500.000	18	"	47"	40	32	Non significant
1296	1204	51	16	"	35"	29	23	Non significant
4122	380	66.500.000	18	"	47"	40	32	Non significant
3021	3405	84	20	"	60"	52	43	Non significant
2479	4631	51	16	"	35"	29	23	Non significant
3254	5458	59.500.000	17	"	41"	34	27	Non significant
726	5331	84	20	"	60"	52	43	Non significant
1361	4691	26	12	"	17"	13	9	Non significant
4134	2474	148.500.000	26	"	110"	98	84	Non significant
1017	7627	148.500.000	26	"	110"	98	84	Non significant
2747	5864	125	24	"	91"	81	69	Non significant
1183	3104	51	16	"	35"	29	23	Non significant
3231	2409	66.500.000	18	"	47"	40	32	Non significant
4494	1272	148.500.000	26	"	110"	98	84	Non significant
2943	4143	45	15	"	30"	25	19	Non significant
4467	4700	125	24	"	91"	81	69	Non significant
4627	614	66.500.000	18	"	47"	40	32	Non significant
169	7469	148.500.000	26	"	110"	98	84	Non significant
2715	5497	66.500.000	18	"	47"	40	32	Non significant
746	6281	66.500.000	18	"	47"	40	32	Non significant
3927	6065	76	19	"	53"	46	37	Non significant
3895	540	76	19	"	53"	46	37	Non significant
4341	4304	103.500.000	22	"	75"	65	55	Non significant
394	6022	94.500.000	21	"	67"	58	49	Non significant
59	4369	84	20	"	60"	52	43	Non significant
2759	1329	51	16	"	35"	29	23	Non significant
3523	6878	115	23	"	83"	73	62	Non significant
3148	1103	125	24	"	91"	81	69	Non significant
2998	6382	103.500.000	22	"	75"	65	55	Non significant
3349	5301	37.500.000	14	"	25"	21	15	Non significant
1800	4165	66.500.000	18	"	47"	40	32	Non significant
3144	6233	37.500.000	14	"	25"	21	15	Non significant
1473	521	51	16	"	35"	29	23	Non significant
2100	2637	103.500.000	22	"	75"	65	55	Non significant
4100	869	16.500.000	10	"	10"	8	5	Non significant
2566	5881	66.500.000	18	"	47"	40	32	Non significant
3332	2624	26	12	"	17"	13	9	Non significant
4108	2554	103.500.000	22	"	75"	65	55	Non significant
4377	3291	103.500.000	22	"	75"	65	55	Non significant
2370	2940	137.500.000	25	"	100"	89	76	Non significant
438	6733	37.500.000	14	"	25"	21	15	Non significant
2145	5785	137.500.000	25	"	100"	89	76	Non significant
3834	4159	26	12	"	17"	13	9	Non significant
3342	4974	16.500.000	10	"	10"	8	5	Non significant
842	1222	51	16	"	35"	29	23	Non significant
1222	706	16.500.000	10	"	10"	8	5	Non significant
2549	36	37.500.000	14	"	25"	21	15	Non significant
3793	1366	127	24	"	91"	81	69	Non significant
3583	797	148.500.000	26	"	110"	98	84	Non significant
423	3147	26	12	"	17"	13	9	Non significant
3139	729	51	16	"	35"	29	23	Non significant
4152	246	137.500.000	25	"	100"	89	76	Non significant
908	7231	66.500.000	18	"	47"	40	32	Non significant
3892	1028	37.500.000	14	"	25"	21	15	Non significant
316	3532	174	28	"	130"	116	101	Non significant
3577	6350	16.500.000	10	"	10"	8	5	Non significant
3809	3734	37.500.000	14	"	25"	21	15	Non significant

2540	2074	125	24	"	91"	81	69	Non significant
80	7748	66.500.000	18	"	47"	40	32	Non significant
261	6967	26	12	"	17"	13	9	Non significant
4017	1353	66.500.000	18	"	47"	40	32	Non significant
1544	3575	84	20	"	60"	52	43	Non significant
4010	740	37.500.000	14	"	25"	21	15	Non significant
1851	2154	66.500.000	18	"	47"	40	32	Non significant
885	7385	84	20	"	60"	52	43	Non significant
3889	4702	84	20	"	60"	52	43	Non significant
2908	1485	162	27	"	119"	107	92	Non significant
949	1944	103.500.000	22	"	75"	65	55	Non significant
2391	4707	37.500.000	14	"	25"	21	15	Non significant
82	849	103.500.000	22	"	75"	65	55	Non significant
342	4538	9	8	"	5"	3	1	Non significant
4228	4466	103.500.000	22	"	75"	65	55	Non significant
536	867	125	24	"	91"	81	69	Non significant
4391	3853	84	20	"	60"	52	43	Non significant
1026	2084	51	16	"	35"	29	23	Non significant
4634	6081	84	20	"	60"	52	43	Non significant
2554	4206	26	12	"	17"	13	9	Non significant
182	7595	51	16	"	35"	29	23	Non significant
3426	5213	76	19	"	53"	46	37	Non significant
2310	6860	76	19	"	53"	46	37	Non significant
2517	3125	103.500.000	22	"	75"	65	55	Non significant
4639	2815	188.500.000	29	"	140"	126	110	Non significant
4414	3927	125	24	"	91"	81	69	Non significant
1619	2519	94.500.000	21	"	67"	58	49	Non significant
4356	1766	103.500.000	22	"	75"	65	55	Non significant
1268	603	37.500.000	14	"	25"	21	15	Non significant
544	6333	137.500.000	25	"	100"	89	76	Non significant
4310	4481	66.500.000	18	"	47"	40	32	Non significant
4347	525	9	8	"	5"	3	1	Non significant
824	1259	115	23	"	83"	73	62	Non significant
168	7095	13.500.000	9	"	8"	5	3	Non significant
4472	3075	125	24	"	91"	81	69	Non significant
311	4109	103.500.000	22	"	75"	65	55	Non significant
3773	1816	16.500.000	10	"	10"	8	5	Non significant
1851	4009	125	24	"	91"	81	69	Non significant
641	2166	45	15	"	30"	25	19	Non significant
4059	3850	137.500.000	25	"	100"	89	76	Non significant
1185	3911	84	20	"	60"	52	43	Non significant
2445	1586	45	15	"	30"	25	19	Non significant
411	6379	66.500.000	18	"	47"	40	32	Non significant
343	2560	37.500.000	14	"	25"	21	15	Non significant
3581	1465	66.500.000	18	"	47"	40	32	Non significant
1064	3886	103.500.000	22	"	75"	65	55	Non significant
3581	6451	51	16	"	35"	29	23	Non significant
2458	5323	66.500.000	18	"	47"	40	32	Non significant
875	906	45	15	"	30"	25	19	Non significant
3124	4342	103.500.000	22	"	75"	65	55	Non significant
757	6007	125	24	"	91"	81	69	Non significant
3002	6719	103.500.000	22	"	75"	65	55	Non significant
2836	4532	9	8	"	5"	3	1	Non significant
2427	2621	9	8	"	5"	3	1	Non significant
151	4830	26	12	"	17"	13	9	Non significant
2210	6735	16.500.000	10	"	10"	8	5	Non significant
3548	7564	125	24	"	91"	81	69	Non significant

4153	4595	84	20	"	60"	52	43	Non significant
886	5804	103.500.000	22	"	75"	65	55	Non significant
2611	3144	37.500.000	14	"	25"	21	15	Non significant
234	4418	76	19	"	53"	46	37	Non significant
4382	7108	84	20	"	60"	52	43	Non significant
832	1632	68.500.000	18	"	47"	40	32	Non significant
804	5333	26	12	"	17"	13	9	Non significant
1367	323	84	20	"	60"	52	43	Non significant
243	7544	201.500.000	30	"	151"	137	120	Non significant
3288	7475	26	12	"	17"	13	9	Non significant
2046	6811	84	20	"	60"	52	43	Non significant
3061	2745	16.500.000	10	"	10"	8	5	Non significant
2814	4275	103.500.000	22	"	75"	65	55	Non significant
10	2270	137.500.000	25	"	100"	89	76	Non significant
3585	7516	137.500.000	25	"	100"	89	76	Non significant
247	935	26	12	"	17"	13	9	Non significant
1425	2484	137.500.000	25	"	100"	89	76	Non significant
992	6482	26	12	"	17"	13	9	Non significant
1058	1463	148.500.000	26	"	110"	98	84	Non significant
841	5808	103.500.000	22	"	75"	65	55	Non significant
2601	6424	66.500.000	18	"	47"	40	32	Non significant
3921	3584	125	24	"	91"	81	69	Non significant
1790	3620	148.500.000	26	"	110"	98	84	Non significant
605	7407	51	16	"	35"	29	23	Non significant
3285	374	66.500.000	18	"	47"	40	32	Non significant
3546	2385	37.500.000	14	"	25"	21	15	Non significant
4161	5498	125	24	"	91"	81	69	Non significant
3064	3261	66.500.000	18	"	47"	40	32	Non significant
3007	3810	125	24	"	91"	81	69	Non significant
3559	3084	125	24	"	91"	81	69	Non significant
1241	2154	3.500.000	6	"	2"	0	0	Non significant
110	6544	103.500.000	22	"	75"	65	55	Non significant
3155	845	9	8	"	5"	3	1	Non significant
3340	309	125	24	"	91"	81	69	Non significant
150	5123	103.500.000	22	"	75"	65	55	Non significant
2557	2905	26	12	"	17"	13	9	Non significant
4343	1089	103.500.000	22	"	75"	65	55	Non significant
2135	3178	86	20	"	60"	52	43	Non significant
3802	4591	37.500.000	14	"	25"	21	15	Non significant
4495	6532	16.500.000	10	"	10"	8	5	Non significant
3852	3111	103.500.000	22	"	75"	65	55	Non significant
1091	6279	137.500.000	25	"	100"	89	76	Non significant
2511	787	51	16	"	35"	29	23	Non significant
3678	6586	37.500.000	14	"	25"	21	15	Non significant
2163	6563	76	19	"	53"	46	37	Non significant
3638	2081	37.500.000	14	"	25"	21	15	Non significant
3541	6833	103.500.000	22	"	75"	65	55	Non significant
4644	3392	37.500.000	14	"	25"	21	15	Non significant
1991	1877	3.500.000	6	"	2"	0	0	Non significant
285	4813	86	20	"	60"	52	43	Non significant
576	7527	115	23	"	83"	73	62	Non significant
111	5558	125	24	"	91"	81	69	Non significant
3211	5304	201.500.000	30	"	151"	137	120	Non significant
2139	2488	66.500.000	18	"	47"	40	32	Non significant
1206	6767	26	12	"	17"	13	9	Non significant
1100	3547	115	23	"	83"	73	62	Non significant
1018	996	66.500.000	18	"	47"	40	32	Non significant

4546	2574	3.500.000	6	"	2"	0	0	Non significant
421	3678	51	16	"	35"	29	23	Non significant
2326	3590	203.500.000	30	"	151"	137	120	Non significant
1774	4494	51	16	"	35"	29	23	Non significant
2884	4150	16.500.000	10	"	10"	8	5	Non significant
392	744	86	20	"	60"	52	43	Non significant
3392	2509	137.500.000	25	"	100"	89	76	Non significant
3926	5508	9	8	"	5"	3	1	Non significant
2783	402	26	12	"	17"	13	9	Non significant
3898	3960	84	20	"	60"	52	43	Non significant
3157	2880	103.500.000	22	"	75"	65	55	Non significant
31	7668	37.500.000	14	"	25"	21	15	Non significant
1565	5889	174	28	"	130"	116	101	Non significant
3179	5563	26	12	"	17"	13	9	Non significant
4425	4108	66.500.000	18	"	47"	40	32	Non significant
2314	6980	174	28	"	130"	116	101	Non significant
3725	318	84	20	"	60"	52	43	Non significant
2335	3188	9	8	"	5"	3	1	Non significant
3540	6618	51	16	"	35"	29	23	Non significant
2496	4505	84	20	"	60"	52	43	Non significant
2728	2049	84	20	"	60"	52	43	Non significant
1407	519	9	8	"	5"	3	1	Non significant
2052	2512	32.500.000	13	"	21"	17	12	Non significant
3104	7731	16.500.000	10	"	10"	8	5	Non significant
2486	4219	103.500.000	22	"	75"	65	55	Non significant
2766	5489	84	20	"	60"	52	43	Non significant
1321	7117	45	15	"	30"	25	19	Non significant
3843	3949	201.500.000	30	"	151"	137	120	Non significant
1974	7293	59.500.000	17	"	41"	34	27	Non significant
937	7723	125	24	"	91"	81	69	Non significant
2077	5903	37.500.000	14	"	25"	21	15	Non significant
3419	6752	32.500.000	13	"	21"	17	12	Non significant
1901	4207	103.500.000	22	"	75"	65	55	Non significant
3294	5773	51	16	"	35"	29	23	Non significant
4039	6158	9	8	"	5"	3	1	Non significant
2427	6782	66.500.000	18	"	47"	40	32	Non significant
4614	6395	26	12	"	17"	13	9	Non significant
2887	4135	162	27	"	119"	107	92	Non significant
2664	160	94.500.000	21	"	67"	58	49	Non significant
2126	5593	66.500.000	18	"	47"	40	32	Non significant
355	5730	51	16	"	35"	29	23	Non significant
241	4519	26	12	"	17"	13	9	Non significant
3800	6998	115	23	"	83"	73	62	Non significant
2788	4860	51	16	"	35"	29	23	Non significant
4206	7363	66.500.000	18	"	47"	40	32	Non significant
510	5784	115	23	"	83"	73	62	Non significant
1976	4764	26	12	"	17"	13	9	Non significant
3823	943	176	28	"	130"	116	101	Non significant
1387	2362	68.500.000	18	"	47"	40	32	Non significant
4592	5344	66.500.000	18	"	47"	40	32	Non significant
2762	4730	84	20	"	60"	52	43	Non significant
3722	3125	125	24	"	91"	81	69	Non significant
223	124	9	8	"	5"	3	1	Non significant
1757	351	66.500.000	18	"	47"	40	32	Non significant
685	5911	201.500.000	30	"	151"	137	120	Non significant
1770	4975	16.500.000	10	"	10"	8	5	Non significant
1033	5597	174	28	"	130"	116	101	Non significant

3915	1460	51	16	"	35"	29	23	Non significant
3181	4947	137.500.000	25	"	100"	89	76	Non significant
2510	4952	45	15	"	30"	25	19	Non significant
4472	5741	188.500.000	29	"	140"	126	110	Non significant
3659	1946	51	16	"	35"	29	23	Non significant
564	4106	51	16	"	35"	29	23	Non significant
950	1801	37.500.000	14	"	25"	21	15	Non significant
355	6869	32.500.000	13	"	21"	17	12	Non significant
2523	1029	51	16	"	35"	29	23	Non significant
373	6327	66.500.000	18	"	47"	40	32	Non significant
1576	6389	37.500.000	14	"	25"	21	15	Non significant
3745	2908	103.500.000	22	"	75"	65	55	Non significant
215	6472	51	16	"	35"	29	23	Non significant
2592	6700	105.500.000	22	"	75"	65	55	Non significant
998	44	137.500.000	25	"	100"	89	76	Non significant
4287	4006	9	8	"	5"	3	1	Non significant
256	3056	66.500.000	18	"	47"	40	32	Non significant
3805	4860	37.500.000	14	"	25"	21	15	Non significant
2433	4282	51	16	"	35"	29	23	Non significant
3157	1041	84	20	"	60"	52	43	Non significant
3726	4744	66.500.000	18	"	47"	40	32	Non significant
412	5491	84	20	"	60"	52	43	Non significant
4588	4295	26	12	"	17"	13	9	Non significant
414	5464	125	24	"	91"	81	69	Non significant
126	7189	51	16	"	35"	29	23	Non significant
3661	3643	16.500.000	10	"	10"	8	5	Non significant
3794	2167	84	20	"	60"	52	43	Non significant
1511	83	37.500.000	14	"	25"	21	15	Non significant
1577	6713	37.500.000	14	"	25"	21	15	Non significant
2648	4823	3.500.000	6	"	2"	0	0	Non significant
1841	1577	115	23	"	83"	73	62	Non significant
120	7347	66.500.000	18	"	47"	40	32	Non significant
3001	6834	37.500.000	14	"	25"	21	15	Non significant
1711	7113	148.500.000	26	"	110"	98	84	Non significant
162	3961	9	8	"	5"	3	1	Non significant
4324	1960	84	20	"	60"	52	43	Non significant
64	7386	66.500.000	18	"	47"	40	32	Non significant
1661	2510	115	23	"	83"	73	62	Non significant
214	5568	66.500.000	18	"	47"	40	32	Non significant
3190	6525	26	12	"	17"	13	9	Non significant
83	4519	32.500.000	13	"	21"	17	12	Non significant
3789	4734	103.500.000	22	"	75"	65	55	Non significant
2919	5425	103.500.000	22	"	75"	65	55	Non significant
2364	1497	76	19	"	53"	46	37	Non significant
248	7722	76	19	"	53"	46	37	Non significant
1245	6831	37.500.000	14	"	25"	21	15	Non significant
357	2525	32.500.000	13	"	21"	17	12	Non significant
1536	2654	103.500.000	22	"	75"	65	55	Non significant
4269	1106	16.500.000	10	"	10"	8	5	Non significant
293	4114	51	16	"	35"	29	23	Non significant
1264	1166	103.500.000	22	"	75"	65	55	Non significant
3011	1739	148.500.000	26	"	110"	98	84	Non significant
957	6089	66.500.000	18	"	47"	40	32	Non significant
796	787	26	12	"	17"	13	9	Non significant
3513	6605	125	24	"	91"	81	69	Non significant
50	3515	125	24	"	91"	81	69	Non significant
1549	1715	26	12	"	17"	13	9	Non significant

1707	1914	3.500.000	6	"	2"	0	0	Non significant
2868	2206	32.500.000	13	"	21"	17	12	Non significant
734	1542	84	20	"	60"	52	43	Non significant
794	276	26	12	"	17"	13	9	Non significant
1726	6862	84	20	"	60"	52	43	Non significant
77	1344	59.500.000	17	"	41"	34	27	Non significant
1989	5373	66.500.000	18	"	47"	40	32	Non significant
956	1065	37.500.000	14	"	25"	21	15	Non significant
4425	1967	127	24	"	91"	81	69	Non significant
4594	3568	125	24	"	91"	81	69	Non significant
2824	912	37.500.000	14	"	25"	21	15	Non significant
2119	620	94.500.000	21	"	67"	58	49	Non significant
2149	2969	125	24	"	91"	81	69	Non significant
967	6661	37.500.000	14	"	25"	21	15	Non significant
1211	4426	84	20	"	60"	52	43	Non significant
4302	2706	32.500.000	13	"	21"	17	12	Non significant
1566	2854	37.500.000	14	"	25"	21	15	Non significant
2818	7380	103.500.000	22	"	75"	65	55	Non significant
4514	4689	103.500.000	22	"	75"	65	55	Non significant
3765	5359	84	20	"	60"	52	43	Non significant
3782	7755	94.500.000	21	"	67"	58	49	Non significant
3201	6987	137.500.000	25	"	100"	89	76	Non significant
88	489	51	16	"	35"	29	23	Non significant
372	6030	115	23	"	83"	73	62	Non significant
2122	471	16.500.000	10	"	10"	8	5	Non significant
3037	7127	51	16	"	35"	29	23	Non significant
2005	6224	66.500.000	18	"	47"	40	32	Non significant
4533	3195	84	20	"	60"	52	43	Non significant
1747	3194	26	12	"	17"	13	9	Non significant
1206	7169	66.500.000	18	"	47"	40	32	Non significant
2230	674	9	8	"	5"	3	1	Non significant
4596	2748	37.500.000	14	"	25"	21	15	Non significant
4108	3930	125	24	"	91"	81	69	Non significant
3542	7435	103.500.000	22	"	75"	65	55	Non significant
3033	4948	94.500.000	21	"	67"	58	49	Non significant
1288	4753	16.500.000	10	"	10"	8	5	Non significant
25	3261	51	16	"	35"	29	23	Non significant
575	1141	148.500.000	26	"	110"	98	84	Non significant
2278	1402	16.500.000	10	"	10"	8	5	Non significant
1157	3322	68.500.000	18	"	47"	40	32	Non significant
2449	3684	125	24	"	91"	81	69	Non significant
2082	7196	59.500.000	17	"	41"	34	27	Non significant
3462	6723	59.500.000	17	"	41"	34	27	Non significant
763	1263	125	24	"	91"	81	69	Non significant
2237	396	103.500.000	22	"	75"	65	55	Non significant
1256	2840	37.500.000	14	"	25"	21	15	Non significant
2347	5978	137.500.000	25	"	100"	89	76	Non significant
3216	2888	201.500.000	30	"	151"	137	120	Non significant
2258	2990	9	8	"	5"	3	1	Non significant
329	475	76	19	"	53"	46	37	Non significant
2991	3078	94.500.000	21	"	67"	58	49	Non significant
55	5461	115	23	"	83"	73	62	Non significant
3380	1949	84	20	"	60"	52	43	Non significant
544	3767	26	12	"	17"	13	9	Non significant
1083	4936	9	8	"	5"	3	1	Non significant
2827	2148	37.500.000	14	"	25"	21	15	Non significant
1766	7693	76	19	"	53"	46	37	Non significant

510	6295	125	24	"	91"	81	69	Non significant
87	993	16.500.000	10	"	10"	8	5	Non significant
2583	4160	137.500.000	25	"	100"	89	76	Non significant
3837	5544	26	12	"	17"	13	9	Non significant
1461	2	188.500.000	29	"	140"	126	110	Non significant
1062	6380	125	24	"	91"	81	69	Non significant
2804	3278	51	16	"	35"	29	23	Non significant
949	2137	84	20	"	60"	52	43	Non significant
833	1214	66.500.000	18	"	47"	40	32	Non significant
2305	478	103.500.000	22	"	75"	65	55	Non significant
2985	6928	76	19	"	53"	46	37	Non significant
1113	1636	115	23	"	83"	73	62	Non significant
342	4776	37.500.000	14	"	25"	21	15	Non significant
2490	6891	94.500.000	21	"	67"	58	49	Non significant
384	2501	103.500.000	22	"	75"	65	55	Non significant
3542	617	94.500.000	21	"	67"	58	49	Non significant
666	223	59.500.000	17	"	41"	34	27	Non significant
3131	1026	125	24	"	91"	81	69	Non significant
3564	1445	51	16	"	35"	29	23	Non significant
3599	7751	137.500.000	25	"	100"	89	76	Non significant
346	7704	115	23	"	83"	73	62	Non significant
1803	1326	66.500.000	18	"	47"	40	32	Non significant
752	3751	125	24	"	91"	81	69	Non significant
2189	4949	103.500.000	22	"	75"	65	55	Non significant
1350	425	51	16	"	35"	29	23	Non significant
2082	5302	103.500.000	22	"	75"	65	55	Non significant
4082	5501	51	16	"	35"	29	23	Non significant
554	2728	37.500.000	14	"	25"	21	15	Non significant
29	7361	26	12	"	17"	13	9	Non significant
3214	5371	16.500.000	10	"	10"	8	5	Non significant
4376	4596	125	24	"	91"	81	69	Non significant
3436	7602	76	19	"	53"	46	37	Non significant
2827	4172	148.500.000	26	"	110"	98	84	Non significant
4539	30	76	19	"	53"	46	37	Non significant
3038	5686	103.500.000	22	"	75"	65	55	Non significant
1038	6495	103.500.000	22	"	75"	65	55	Non significant
244	7730	66.500.000	18	"	47"	40	32	Non significant
3126	7578	16.500.000	10	"	10"	8	5	Non significant
1200	3242	26	12	"	17"	13	9	Non significant
4577	3704	9	8	"	5"	3	1	Non significant
1987	502	84	20	"	60"	52	43	Non significant
3938	7460	66.500.000	18	"	47"	40	32	Non significant
2872	7185	137.500.000	25	"	100"	89	76	Non significant
1712	994	16.500.000	10	"	10"	8	5	Non significant
2984	3001	59.500.000	17	"	41"	34	27	Non significant
3157	6352	125	24	"	91"	81	69	Non significant
2013	3881	84	20	"	60"	52	43	Non significant
3970	6942	84	20	"	60"	52	43	Non significant
1051	3287	125	24	"	91"	81	69	Non significant
676	1837	37.500.000	14	"	25"	21	15	Non significant
1213	3564	125	24	"	91"	81	69	Non significant
1797	2729	84	20	"	60"	52	43	Non significant
685	6246	66.500.000	18	"	47"	40	32	Non significant
2863	3654	16.500.000	10	"	10"	8	5	Non significant
3531	6480	66.500.000	18	"	47"	40	32	Non significant
129	1596	84	20	"	60"	52	43	Non significant
536	4892	103.500.000	22	"	75"	65	55	Non significant

3631	5281	51	16	"	35"	29	23	Non significant
4642	3782	174	28	"	130"	116	101	Non significant
2415	2571	9	8	"	5"	3	1	Non significant
1717	3113	37.500.000	14	"	25"	21	15	Non significant
2976	7554	59.500.000	17	"	41"	34	27	Non significant
449	7133	16.500.000	10	"	10"	8	5	Non significant
4254	6183	9	8	"	5"	3	1	Non significant
2006	3767	103.500.000	22	"	75"	65	55	Non significant
1370	935	76	19	"	53"	46	37	Non significant
3074	318	103.500.000	22	"	75"	65	55	Non significant
3952	6653	53	16	"	35"	29	23	Non significant
855	1039	66.500.000	18	"	47"	40	32	Non significant
3266	7595	16.500.000	10	"	10"	8	5	Non significant
3106	1744	125	24	"	91"	81	69	Non significant
678	6071	84	20	"	60"	52	43	Non significant
2757	884	76	19	"	53"	46	37	Non significant
1828	518	66.500.000	18	"	47"	40	32	Non significant
876	1748	51	16	"	35"	29	23	Non significant
1235	1177	84	20	"	60"	52	43	Non significant
860	1288	66.500.000	18	"	47"	40	32	Non significant
1626	6793	9	8	"	5"	3	1	Non significant
2576	2471	51	16	"	35"	29	23	Non significant
1461	3235	37.500.000	14	"	25"	21	15	Non significant
1494	3428	66.500.000	18	"	47"	40	32	Non significant
2283	228	127	24	"	91"	81	69	Non significant
787	1413	37.500.000	14	"	25"	21	15	Non significant
3396	7731	115	23	"	83"	73	62	Non significant
2029	1506	51	16	"	35"	29	23	Non significant
1223	322	51	16	"	35"	29	23	Non significant
1297	3650	32.500.000	13	"	21"	17	12	Non significant
2185	5506	51	16	"	35"	29	23	Non significant
3295	6262	51	16	"	35"	29	23	Non significant
2632	553	125	24	"	91"	81	69	Non significant
